# A Family of Superhelicenes: Easily Tunable, Chiral Nanographenes by Merging Helicity with Planar π Systems

**DOI:** 10.1002/anie.202103253

**Published:** 2021-07-09

**Authors:** David Reger, Philipp Haines, Konstantin Y. Amsharov, Julia A. Schmidt, Tobias Ullrich, Simon Bönisch, Frank Hampel, Andreas Görling, Jenny Nelson, Kim E. Jelfs, Dirk M. Guldi, Norbert Jux

**Affiliations:** ^1^ Department of Chemistry and Pharmacy & Interdisciplinary Center for Molecular Materials (ICMM) Friedrich-Alexander-University Erlangen-Nuremberg Nikolaus-Fiebiger-Straße 10 91058 Erlangen Germany; ^2^ Department of Chemistry and Pharmacy & Interdisciplinary Center for Molecular Materials (ICMM) Friedrich-Alexander-University Erlangen-Nuremberg Egerlandstraße 3 91058 Erlangen Germany; ^3^ Institute for Organic Chemistry Martin Luther University Halle-Wittenberg Kurt-Mothes-Straße 2 06120 Halle Germany; ^4^ Department of Chemistry Molecular Sciences Research Hub Imperial College London White City Campus, Wood Lane London W12 0BZ UK; ^5^ Department of Physics Imperial College London South Kensington Campus London SW7 2AZ UK

**Keywords:** circular dichroism, helicenes, luminescence, nanographene, polycyclic aromatic hydrocarbons

## Abstract

We designed a straightforward synthetic route towards a full‐fledged family of π‐extended helicenes: superhelicenes. They have two hexa‐peri‐hexabenzocoronenes (HBCs) in common that are connected via a central five‐membered ring. By means of structurally altering this 5‐membered ring, we realized a versatile library of molecular building blocks. Not only the superhelicene structure, but also their features are tuned with ease. In‐depth physico‐chemical characterizations served as a proof of concept thereof. The superhelicene enantiomers were separated, their circular dichroism was measured in preliminary studies and concluded with an enantiomeric assignment. Our work was rounded‐off by crystal structure analyses. Mixed stacks of M‐ and P‐isomers led to twisted molecular wires. Using such stacks, charge‐carrier mobilities were calculated, giving reason to expect outstanding hole transporting properties.

## Graphical abstract

We present the synthesis and in‐depth physico‐chemical characterization of a family of π‐extended helicenes that consist of HBCs fused via different five‐membered rings. Despite being only a small part of the compounds, the nature of the five‐membered rings has a huge impact on the properties of the whole molecule. We show the enantiomeric separation and circular dichroism properties together with X‐ray analysis and charge transport calculations.

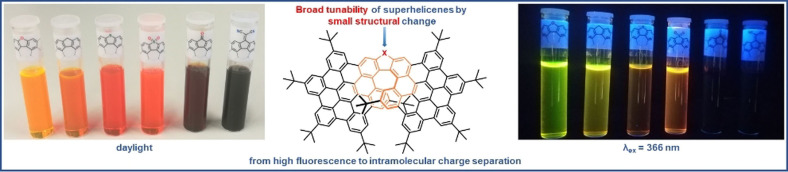

## Introduction

PAHs have attracted tremendous interest in recent decades both in terms of potential novel materials,^[1]^ as well as a benchmark for carbon allotropes (e.g. graphene, carbon nanotubes or fullerenes).^[2]^ To this end, many different planar and non‐planar PAHs (e.g. buckybowls, helicenes or other curved structures) have been synthesized, studied, and applied. Among these, conformationally stable π‐extended [*n*]‐helicenes (with *n*≥5) have lately received increased attention.^[3]^ Such helicenes commonly consist of large planar segments, that is, the “π‐extension” and one or more twisted parts, that is, the “helicene”. [*n*]‐helicenes feature unique characteristics that differ strongly both from planar PAHs as well as from non π‐extended helicenes. Their properties are paired with their inherent chirality, a feature that many PAHs lack.

Apart from potential applications, which are well‐documented for PAHs in general,^[1]^ chirality opens up additional possibilities ranging from enantioselective catalysis^[4]^ to applications based on circular polarized light absorption and emission such as advanced display technologies,^[5]^ chiral molecular probes and sensors,^[6]^ improved security inks,[^6a^, ^7^] and chiroptical switches.^[8]^

Spin‐filtering properties of chiral compounds, known as the chirality induced spin selectivity effect (CISS‐effect),^[9]^ render helicenes intriguing targets for spin dependent applications^[10]^ like spintronics. Finally, helicity is a useful tool to fine‐tune the parameters for organic materials, as mixtures of enantiomers in different ratios lead to varying solid‐state packings and, therefore, different properties.^[11]^ For many of these purposes, such as optical and electronic applications, the π‐extension of helicenes is beneficial due to the strong visible light absorption and fluorescence, tunable semiconducting properties, and aggregation of large π‐systems.

In the context of future developments, the synthesis of π‐extended helical compounds might also pave the way towards the design of novel helical carbon allotropes or so‐called graphene spirals, for which outstanding optical and electronic properties have been predicted.^[12]^

## Results and Discussion

**Figure 1:**
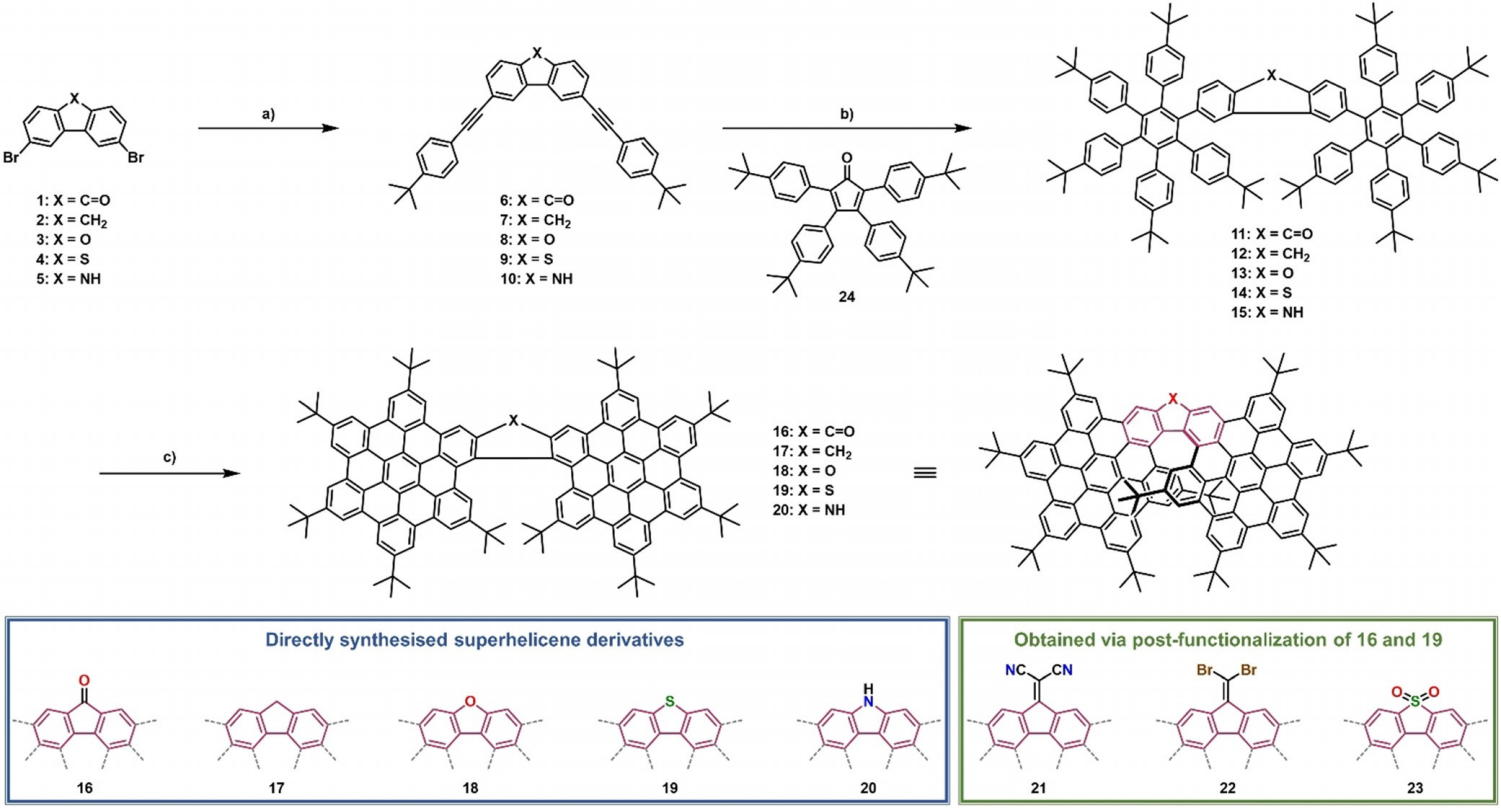
Synthesis of superhelicene derivatives. **16**–**20** (blue box) were synthesized directly from precursors **1**–**5** via a reaction sequence including a double Sonogashira coupling to obtain **6**–**10** followed by a Diels–Alder reaction with tetraphenylcyclopentadienone **24** to obtain **11**–**15**. Those double HAB derivatives were finally closed to the helicenes **16**–**20** under oxidative cyclodehydrogenation conditions. **21**–**23** (green box) were synthesized via post‐functionalization of **16** and **19**. Conditions: a) Pd(PPh_3_)_4_, (5 mol %), CuI (5–10 mol %), 4‐*tert*‐butylphenylacetylene (2.2–3.0 equiv.), DMF/NEt_3_, N_2_, 16–19.5 h, 90–100 °C; 29–82 %; b) **24** (2.5 equiv.), toluene, N_2_, 26–131 h, 220 °C (pressure flask), 67–93 %; c) FeCl_3_ (30 equiv.) in MeNO_2_ (ca. 300 mg(FeCl_3_) mL^−1^), dichloromethane, N_2_, 2–4.5 h, 0 °C to rt., 64–96 % (for **16**–**19**), DDQ (12 equiv.), triflic acid (28 equiv.), dichloromethane, N_2_, 3 h, 0 °C to rt., 79 % (for **20**); conditions for **21**: **16**, malononitrile (15 equiv.), pyridine, 1.5 h, 80 °C, 96 %; conditions for **22**: **16**, CBr_4_ (2 equiv.), PPh_3_ (4 equiv.), toluene, N_2_, 24 h, reflux, 58 %; conditions for **23**: **19**, *m*‐CPBA (5 equiv.), dichloromethane, 68 h, 0 °C to rt., 52 %.

A novel structural motif, that is, π‐extended helicenes was recently published by our group:^[13]^ Oxa‐[7]‐superhelicene. Inspired by its unique structure together with its striking features, we aimed at, on one hand, enlarging this family of superhelicenes and, on the other hand, gathering a deeper understanding of π‐extended helicenes. Consequently, the next step was to vary the nature of the heteroatoms in the central five‐membered ring. To this end, we synthesized eight new superhelicenes either directly or indirectly via post‐functionalization (Figure 1). Such a versatility is rather uncommon and renders our superhelicenes to stand out from the vast majority of published π‐extended helicenes.^[3]^ In the original synthesis of oxa‐[7]‐superhelicene,^[13]^ we started from a diphenyl ether precursor. In the final step of the synthesis, it was necessary not only to close the hexaarylbenzenes, but also the furan ring to obtain the extended helicene. The ring closure in the last step towards oxa‐[7]‐superhelicene proved to be difficult. It only proceeded cleanly under certain Scholl conditions. Thus, we focused in our current work on employing precursors that have already a closed, central, five‐membered ring. Precursors 2,8‐dibromofluorenone **1**,^[14]^ 2,8‐dibromofluorene **2**,^[14]^ 2,8‐dibromodibenzofuran **3**,^[15]^ 2,8‐dibromodibenzothiophene **4** ^[16]^ and 2,8‐dibromocarbazole **5** ^[17]^ were synthesized following adapted literature procedures starting from 9,10‐phenanthrenquionene, dibenzofuran, dibenzothiophene, and carbazole, respectively. The brominated precursors were then converted into the corresponding helicenes in an efficient three‐step synthesis. First, a double Sonogashira reaction with 4‐*tert*‐butylphenylacetylene was performed to provide the necessary triple bonds for the subsequent Diels–Alder reactions. These were conducted with 2.5 equiv. of 2,3,4,5‐tetrakis[4‐(*tert*‐butyl)phenyl]cyclopenta‐2,4‐diene‐1‐one **24** in minimal amounts of toluene in pressure vials at high temperatures of 220 °C. Bis‐hexaarylbenzenes **11**–**15** were reacted under classical Scholl conditions with FeCl_3_/MeNO_2_or DDQ/triflic acid in dichloromethane to afford superhelicenes **16**–**20**. Despite the closure of 12 bonds in a single step, the reaction proceeds cleanly. It should be noted that **20** decomposes fast (within several hours) under ambient conditions. This might be attributed to the strong +M effect of the N−H making the HBC subunits very electron rich and reactive. A similar instability was observed by Müllen and co‐workers for amino‐HBC.^[18]^

Fluorenone‐based **16** and thiophene‐based **19** were used for post‐functionalizations. The ketone functionality of **16** was utilized for the conversion to **21** via a Knoevenagel condensation with malononitrile or to **22** with tetrabromomethane and triphenylphosphine, which is known to be the first step in the Corey–Fuchs–Ramirez reaction. Notably, **22** slowly decomposes (within several weeks) under ambient conditions. Except for **20** and **22**, all helicenes are long time stable under ambient conditions. **19** was oxidized to sulfone‐derivative **23** with *meta*‐chloroperoxybenzoic acid in dichloromethane.^[19]^

Detailed physico‐chemical investigations corroborated the tunability of our superhelicenes. As such, this is remarkable as only the five‐membered, central ring was altered. In the following, detailed information is given for **16**, **17**, **19**, **21** and **23. 20** and **22** are excluded due to the low stability of **20** and due to the fact that **22** was only synthesized for later post‐functionalization. For **18**, detailed information is given in our previous publication.^[13]^

The absorptions of **16**, **17**, **19**, **21**, and **23** resemble the absorption features of the recently published oxa‐[7]‐superhelicene **18**.^[13]^ In particular, PAH‐centered β‐ and p‐bands are discernable between 300 and 440 nm, while the α‐bands are observed between 440 and 550 nm (Supporting Information, Table S1). Similar to our previous results, the α‐bands are intense due to symmetry lowering in the bent HBC structure. The same holds for **17**, **19**, and **23**. In toluene, **16** exhibits an additional absorption, which is red‐shifted relative to the α‐bands to 584 nm. Finally, **21** features a 571 nm maximum, a shoulder at 624 nm, and a weak, red‐shifted absorption at around 750 nm.

**Figure 2:**
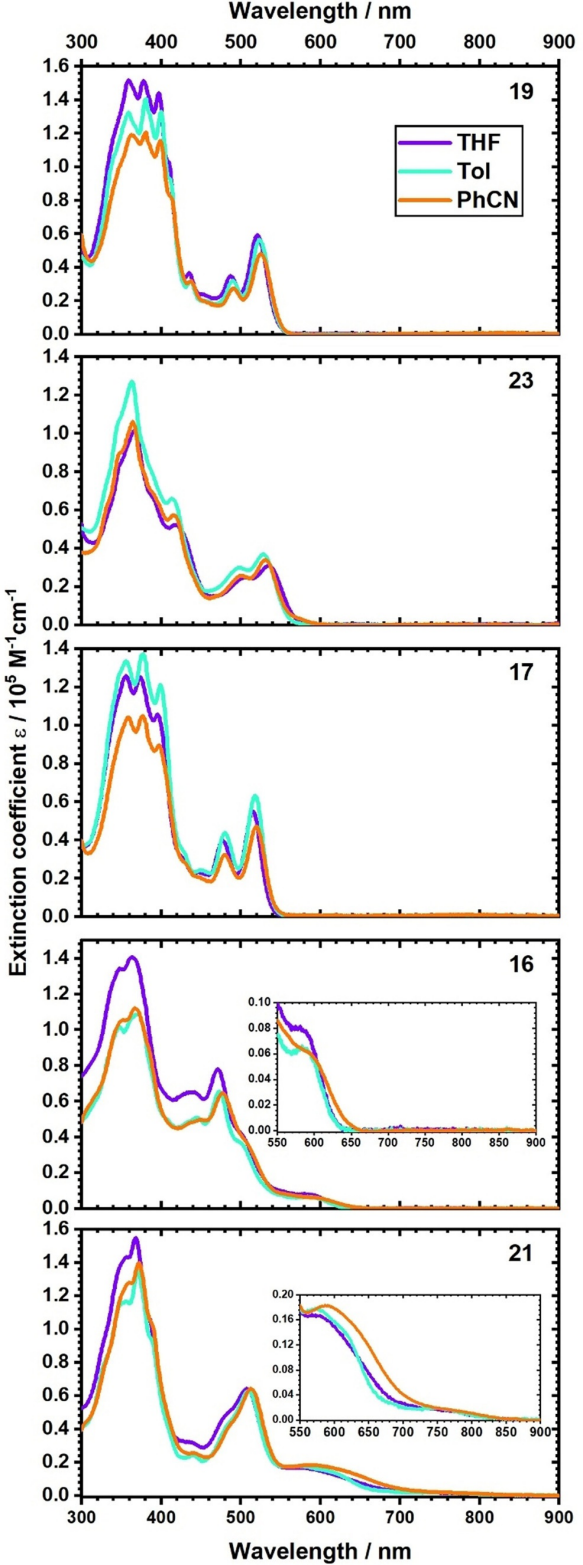
Absorption spectra of **19**, **23**, **17**, **16**, and **21** in toluene (turquoise), THF (purple), PhCN (orange). The charge transfer (CT) absorption bands of **16** and **21** are highlighted by the insets.

In general, the absorption spectra reveal in solvents of different polarities only a minor solvatochromism (Figure 2). For **23** in PhCN, an additional low‐intense absorption band appears red‐shifted at 588 nm, which indicates an additional transition in the low energy regime. More drastic are, however, the changes for **16** and **21**. For example, in PhCN rather than toluene, the absorptions of **16** red‐shift by 15 nm. For **21**, overlaying absorptions render it, nevertheless, much harder to discern any underlying shifts. Therefore, multipeak analyses were applied to dissect the different features (Figure S1). In toluene, **21** exhibits absorptions at 571, 623, and 738 nm. When changing to PhCN, red‐shifts of 16, 13, and 27 nm, respectively, evolved. Compared to the most intense α‐band, which 4.5 nm shifts, the strong solvatochromic effects substantiates an underlying charge‐transfer character.^[20]^

The fluorescence features support the conclusions from absorption spectroscopy (Figure S2). **17**, **19**, and **23** show fluorescence, which is similar to that reported previously for **18**. For example, **17** exhibits intense fluorescence at 537 and 570 nm. In toluene, the fluorescence quantum yield is 86 %. By switching to PhCN, the quantum yield is somewhat lower with 69 %. **19** and **23** both possess very similar fluorescence features. For **23** in PhCN, the fluorescence red‐shifts moderately by 13 nm (Figure S2). This points to a weak CT character of **23**. Their quantum yields are moderate with 36 % and 61 % in toluene. As aforementioned, the quantum yields decrease with increasing solvent polarity (Table 1). In stark contrast, the fluorescence of **16** is shifted and broadened. Here, the maximum is located at 631 nm and is subject to a polarity‐dependent red‐shift of 35 nm, when, for example, switching from toluene to PhCN. The quantum yields are distinctly low with 6.6 %, 6.9 %, and 1.8 % in toluene, THF, and PhCN, respectively. **21** reveals fairly weak fluorescence, which resembles that of **17** and lacks polarity‐dependence. A closer look at the fluorescence in PhCN (Figure S4) reveals the absence of any charge‐transfer features. Instead, **21** fluoresces at around 540 nm. The corresponding quantum yields were far less than 0.1 %. Hereby, the choice of the solvent is irrelevant, as the quantum yields remained low in both toluene and PhCN. By means of 3D fluorescence spectroscopy, the fluorescence of **16**, **17**, **19**, **21**, and **23** was found to be independent of the excitation (Figures S3–S7).

**Table 1:** Fluorescence quantum yields of all superhelicenes in toluene, THF, and PhCN.^[a]^

	Toluene [%]	THF [%]	PhCN [%]
19	36	20	18
23	61	38	38
17	86	60	69
16	6.6	6.9	1.8
21	<0.1	0.2	<0.1

[a] Rhodamine 6G was used as a reference.

The broad tunability of π‐extended helicenes is a great asset. On one hand, strongly fluorescent helicenes are realized with quantum yields as high as 86 % in, for example, **17**. Such a value is exceptionally high for helicenes and/or nanographenes. On the other hand, broad absorptions all the way up to 850 nm were recorded for **21**. Here, the combination of a large π‐extended helicene and an electron acceptor evokes panchromic absorptions. Panchromaticity is uncommon for helicenes and, in turn, only scarcely investigated.

By means of time‐correlated single photon counting (TCSPC), the radiative lifetimes were determined upon photo‐excitation at, for example, 480 nm (Table 2 and Figure S39). Measurements with **19** reveal lifetimes in the range from 1.3 to 1.4 ns in toluene, THF, and PhCN. Compared to **23** and **17**, which exhibit lifetimes of 2.8–3.2 ns and 3.2–3.8 ns, respectively, the fluorescence of **19** is, however, rather short‐lived. The charge transfer state of **16** radiates with substantially lower quantum yields, but longer lifetimes all the way up to 4.4 ns.^[21]^

**Table 2:** Fluorescence lifetimes of all superhelicenes in toluene, THF, and PhCN obtained by TCSPC measurements.^[a]^

TCSPC lifetimes	Toluene [ns]	THF [ns]	PhCN [ns]
19	1.31	1.34	1.35
23	2.82	3.21	3.17
17	3.23	3.78	3.41
16	4.19	4.42	3.87
21	–	–	–

[a] Excitation wavelength was set to 480 nm and emission wavelength to the corresponding emission maximum.

Next, the oxidations and reductions, which were determined using a three‐electrode electrochemical setup, are considered. In dichloromethane, the first and second oxidations evolved for all superhelicenes at around +1.0 and +1.2 V, respectively (Table 3). Assignments of the reductions are far more complex. Except for **21**, all superhelicenes undergo reductions at around −1.5 V. In **17** and **19**, this is, however, the first reduction, while in **16** and **23** the first reduction occurs at −1.1 V. In the case of **21**, the lowest reduction occurs at −0.7 V. The shift in reduction for **16**, **21**, and **23** leads to a lowering of the band gap. This matches the trend seen in the steady‐state absorption measurements, in which the absorption features are red‐shifted. The bands at 583 nm for **16** and 572–743 nm for **21** are too far red‐shifted for HBC typical α‐bands and point to the presence of a charge‐transfer state stemming from a redistribution of charge‐density between the π‐extended helicenes and the functional groups at the five‐membered central ring. DPVs and CVs are depicted in Figures S9–S11.

**Table 3:** Electrochemical oxidation and reduction of all superhelicenes in dichloromethane.^[a]^

	E^Red^_3_ [V]	E^Red^_2_ [V]	E^Red^_1_ [V]	E^Ox^_1_ [V]	E^Ox^_2_ [V]
19	−1.5			1.0	1.2
23	−1.5	−1.1		1.2	1.4
17	−1.5			0.9	1.2
16	−1.5	−1.1		1.0	1.3
21		−1.1	−0.7	1.0	1.2

[a] The internal reference was Fc/Fc^+^ and the electrolyte was TBAPF_6_.

Spectroelectrochemical measurements were carried out to oxidize **16**, **17**, **19**, **21**, and **23** at a potential of 1.0 V. Hand‐in‐hand with the oxidation is a decrease of the α‐, β‐ and p‐absorptions and the concomitant formation of new characteristics between 400 and 500 nm and between 550 and 800 nm. Here, local maxima are seen at 560, 700, and 750 nm. For **17**, the broad absorption with a maximum around 700 nm is missing. All differential absorption spectra are shown in Figure S38.

Transient absorption experiments were carried out on the femtosecond (fs‐TAS) and nanosecond (ns‐TAS) timescales in toluene, THF, and PhCN. Upon fs‐TAS photo‐excitation at 550 nm, **19** exhibits in toluene a characteristic ground‐state bleaching with minima at 492 and 545 nm, together with a stimulated emission minimum at 600 nm. All of the aforementioned is rounded off by maxima at 435, 630, 800, and 958 nm. At longer time delays, the minima at 492 and 545 nm and the transient absorption at 958 nm blue‐shift. At the same time, the stimulated emission minimum at 600 nm disappears and is replaced by a maximum around 550 nm. In THF and PhCN, the excited state characteristics and dynamics are very similar (Figures S20–S25). By employing global analysis, we identified two excited state species in the fs‐TAS. The first state, for which the lifetime is 1.22 ns, is assigned to the singlet excited state, which deactivates to the ground state by fluorescence and to the triplet excited state by intersystem crossing. This is the second species and it deactivates in 11.4 μs to the ground state.^[22]^

Immediately after 550 nm fs‐TAS photo‐excitation, **16** shows in toluene a strong ground‐state bleaching at 475 nm, positive transients at 575, 659, 737, and 757 nm, and a broad shoulder between 800 and 900 nm. On the time scale of fs‐TAS, neither complete transformation nor deactivation of the excited state species was noted. Therefore, we turned to ns‐TAS to monitor the complete deactivation. Immediately after ns‐TAS photo‐excitation, excited state species are observed, which agree well with those in the fs‐TAS experiments, but with a poorer fine structure. Within the first nanoseconds, the excited state features are replaced by maxima at 550 and 640 nm, which then decay to the ground state. We employed global analysis to identify two different excited state species in fs‐TAS with lifetimes of 1.04 ps and >5 ns (Figure S12). In ns‐TAS, two different excited state species were also concluded (Figure S15). Important is hereby that the first ns‐TAS species is identical to the second fs‐TAS species and lives for 5.11 ns. The second, long‐lived excited state species decays with 21.03 μs. We assign the 1.04 ps‐lived species to a higher unrelaxed singlet excited state. In line with the results from steady‐state and time‐resolved fluorescence measurements, the 5.11 ns‐lived species, whose ground‐state bleaching slightly blue‐shifts with time, is ascribed to a charge‐transfer state. It deactivates in parallel to either the ground‐state as a major pathway or to the triplet excited state as a minor pathway. From the latter, the ground‐state is recovered within 21.03 μs. In THF, the same trend is observed. Negligible are the changes associated with the three different states (Figures S13 and S16). Quite different is, however, the picture in PhCN. Here, the first lifetime increases to 18.49 ps, while the second lifetime is as short as 2.42 ns. The third species deactivates in 53.31 μs (Figures S14 and S17).

**Figure 3:**
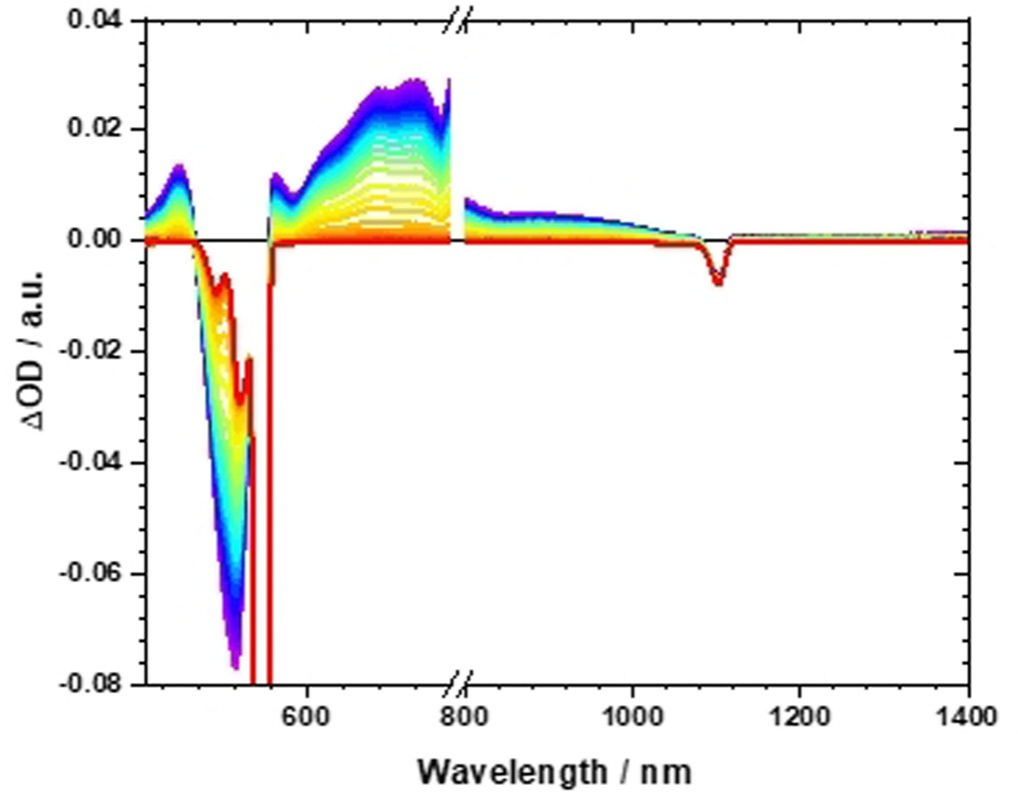
Femtosecond transient absorption spectra of **21** in PhCN with different time delays between 0 and 200 ps.

**Figure 4:**
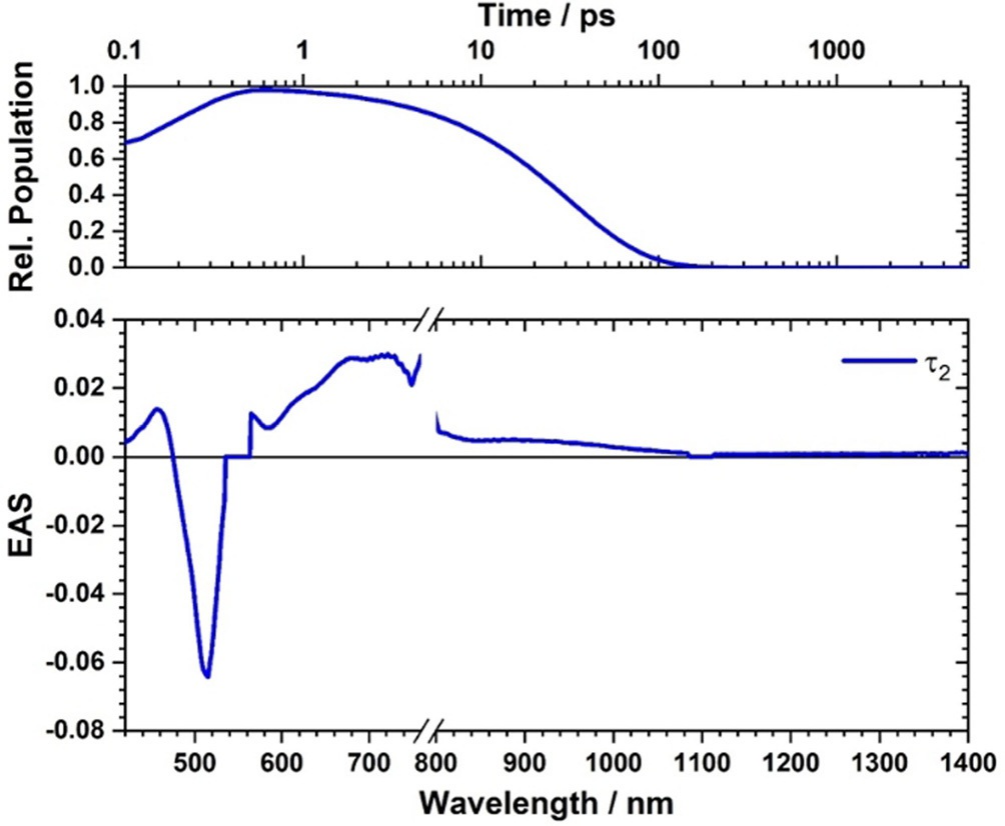
Evolution associated spectrum of **21** in PhCN with time delays from 0–5500 ps upon excitation at 550 nm.

**21** contrasts the excited‐state deactivation eluded for **19** and **16**. Immediately after fs‐TAS photo‐excitation, a strong ground‐state bleaching develops at 500 nm together with transients at 430, 450, 720, 757, and 870 nm. It is within a few picoseconds that the transients decay to the ground state. No notable changes and no appreciable population of any triplet excited state are, however, seen (Figure S18). By means of global analysis, a mono‐exponential decay was found, by which the ground state is recovered. In toluene, the lifetime is 53 ps. In THF (Figure S19) and PhCN (Figure 3 and 4), the decays are even faster with 38 and 32 ps, respectively. The lifetimes found for **21** are assigned to charge recombination from a charge‐separated state rather than deactivation of the charge‐transfer state. Support for our assignment came from the fact that the transient maxima at 430, 450, and 720 nm match the fingerprints of the differential absorption spectra for **21** upon applying oxidative conditions (Figure S38).^[23]^ Accordingly, we postulate that the initially formed charge‐transfer state transforms in less than 200 fs into a charge‐separated state.

**Figure 5:**
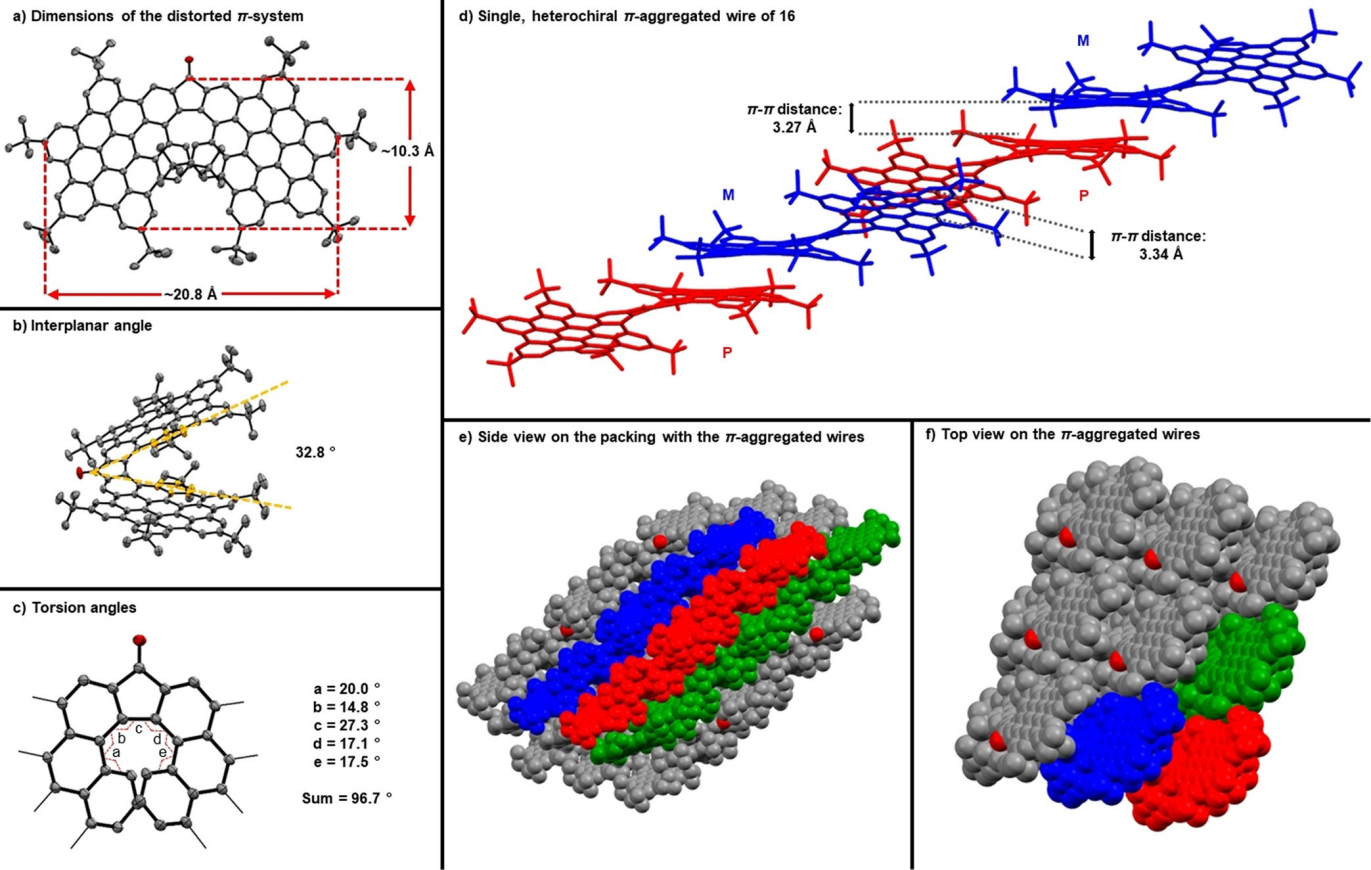
Crystal structure analysis of **16**.^[29]^ a) Dimensions of the π‐system; b) interplanar angle; c) torsion angles; d) single molecular wire formed by a mixed stack of M‐ and P‐enantiomers; e) side view of a bundle of those π‐aggregated wire; f) top view of the bundle of π‐aggregated wires.

For **16**, we present the first high quality crystal structure for one of our superhelicenes (Figure 5). The size of the distorted π‐extended helicene is without the carbonyl group approximately 20.8 Å×10.3 Å. This underlines the fact that **16** is a real helical nanographene. In **16**, the sum of the 5 torsion angles of the [7]helicene core is 96.8° with interplanar/dihedral angles of 32.8°. Overall, the central, helical part is far more twisted compared to its parent structure, that is, fluorenone[7]helicene. In the latter the sum of the 5 torsion angles is 82.8° and the dihedral angle is 26.8°.^[24]^ We postulate that this distortion is due to the bulky *tert*‐butyl groups, which point inwards and, in turn, push the HBC‐plates apart.

The crystal packing is dominated by mixed stacks of M‐ and P‐isomers forming twisted molecular wires. They consist of two different dimers based on π–π interactions between the HBC plates. The π–π distances are around 3.27 and 3.34 Å. The surrounding *tert*‐butyl groups prevent direct π‐π interactions between the single wires but keep them together presumably by weak dispersion interactions.

**Figure 6:**
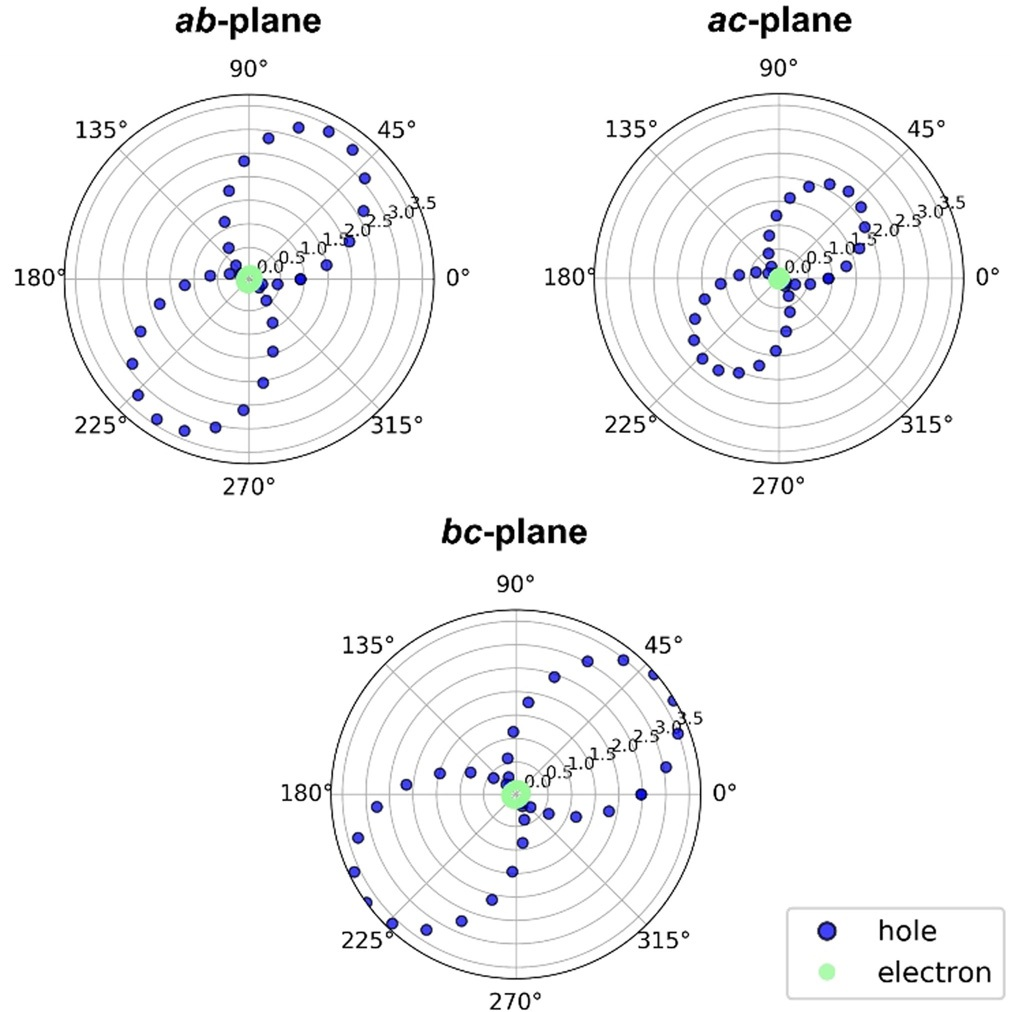
Hole (blue) and electron (green) charge carrier mobility (cm^2^ V^−1^ s^−1^) for the crystal structure of **16** in the *ab*, *ac* and *bc* plane.

To gain insight into the capability for charge transport of these wire‐like columns of **16**, we calculated electron and hole mobilities within the crystal structure, assuming a hopping regime of charge transport as described by semi‐classical Marcus Theory.^[25]^ **16** gives rise to a high hole mobility, but virtually no electron mobility in all directions. The negligibly small electron mobility of **16** with low maximum electron transfer integrals (*J*_max_=16 meV) could be related to electron being localized on the molecule, which is suggested by our visualization of the LUMO (Figure S59). If the electron wavefunction is strongly localized in a part of a large molecule, then wavefunction overlap between neighboring molecules can be reduced. The promising hole mobility of **16** is a combination of high hole transfer integrals between neighboring pairs of the molecules within the crystal (*J*_max_=95 meV) (Figure S42) and a relatively low inner reorganization energy *λ*_intra_(hole)=100 meV. Taking the outer reorganization energy *λ*_outer_=0.30 eV, this leads to an average hole mobility of 1.56 cm^2^ V^−1^ s^−1^ and a maximum hole mobility of 3.75 cm^2^ V^−1^ s^−1^ (Figure 6) along the *bc* plane. For comparison, from equivalent mobility calculations with higher outer reorganization energies (*λ*_outer_=0.5 eV), hole mobilities for TIPS‐pentacene (0.33 cm^2^ V^−1^ s^−1^) and TES‐pentacene (0.28 cm^2^ V^−1^ s^−1^) are lower than for **16**.^[26]^ In TIPS‐Pn the lower hole mobility can be attributed to the lower hole transfer integral (*J*_max_=65 meV) and the increased inner and outer *λ*. In TES‐Pn, *J*_max_ is equal to *J*_max_ in **16**; the lower hole mobility is largely related to a higher *λ* (*λ*_inner_=142 meV). The maximum hole mobility of 3.75 cm^2^ V^−1^ s^−1^ in **16** exceeds all hole mobilities for helicenes previously computed (i) aza[6]helicene regioisomers (*λ*_outer_=0.30 eV) 0.02–0.26 cm^2^ V^−1^ s^−1^;^[27]^ (ii) carbo[6]helicene (*λ*_outer_=0.14 eV) 0.00–2.00 cm^2^ V^−1^ s^−1^;[^11b^, ^11c^] (iii) (rac)‐aza[6]helicene 0.06 cm^2^ V^−1^ s^−1^ and (+)‐aza[6]helicene 0.03 cm^2^ V^−1^ s^−1^.^[11a]^ Those helicenes generally show smaller hole transfer integrals and larger *λ*_intra_ *(hole)* as would be expected for smaller molecules. Therefore, we have reason to believe that the crystal structure of **16** could be the basis for good hole‐transporting materials.

It is worth highlighting that the high hole mobility is relatively isotropic, meaning that the average mobility is the same in all 3D directions and, thus, the material is not limited to a 2D plane of good transport. The calculated poor electron mobility (*μ*_max_=0.031 cm^2^ V^−1^ s^−1^) of **16** is a result of a high inner reorganization energy *λ*_intra_(electron)=213 meV and a low *J*_max_=16 meV.

The charge carrier mobilities for the previously reported **18** ^[13]^ were calculated as well but are unreliable due to a highly disordered crystal structure. For further details see the Supporting Information, Section 5.

Enantiomeric separation via analytical HPLC (Figures S47–S48) was possible and **16**, **17**, **18**, as well as **19** were separated successfully. Circular dichroism (CD) spectra of the separated isomers were measured. By comparison with the spectra obtained from time‐dependent density‐functional (TDDFT)^[28]^ calculations, the M‐ and the P‐isomers could be assigned (Figures S50–S57; Table S10).

**Figure 7:**
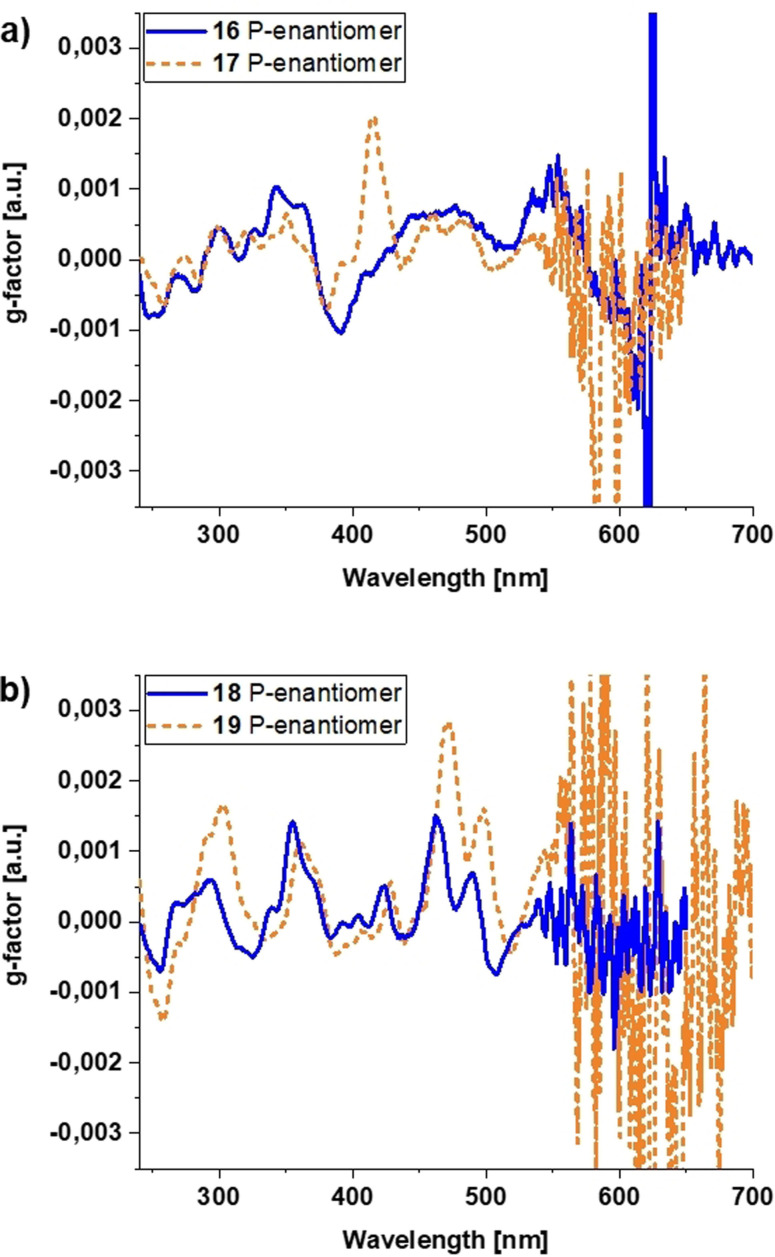
Determined g_Abs_ for P‐enantiomers of a) **16**, **17** and b) **18**, **19**.

From the CD spectra, the g‐factors for the isomers were determined (Figure 7). For clarity reasons only the g‐factor of the P‐enantiomers are displayed. The mirror image spectra of the respective M‐enantiomers are shown in the supporting information (Figure S58). The maximum g‐factors (Table 4) are around 10^−3^, which is relatively common for small organic molecules. Interestingly, the spectra of **18** and **19** are quite similar with the peaks of **19** slightly red shifted. However, g_max_ of **19** is approximately twice as high as g_max_ of **18**. As the only difference is that in **19** sulfur is present compared to oxygen in **18** (both chalcogenides) it could be argued that the presence of the heavier atoms increases the g‐factor.

**Table 4:** Maximum g‐factors (g_max_) for the **16**, **17**, **18** and **19** and the respective wavelength.

Compound	Wavelength [nm]	|g_max_|
16	342, 390	1.0×10^−3^
17	416	2.0×10^−3^
18	462	1.5×10^−3^
19	470	2.8×10^−3^

## Conclusion

We have presented a full‐fledged family of superhelicenes, whose structures and, in turn, physico‐chemical characteristics are freely tunable. Please note that this has never been shown in such a versatile way for π‐extended helicenes. Particularly noteworthy are the very high fluorescence quantum yields of up to 80 % in **17**, **18**, **19**, and **23**. Remarkable is also the fact that in **16** and **21** the absorption covers nearly the entire visible region. All of the aforementioned was independently supported by excited‐state deactivation pathways that differed as a function of the functionalization at the 5‐membered ring. We did document that the superhelicenes are tolerant to post‐functionalization reactions to gather an even larger library of superhelicenes. A detailed solid‐state crystallographic analysis was presented for **16**. Based on this analysis, we used calculations to demonstrate the unique potential of **16** as a hole transporting material. In light of the latter, further research, which, for example, will include thin‐film and/or single‐crystal measurements needs to be carried out to verify the results experimentally. We succeeded in separating the enantiomers of **16**, **17**, **18**, and **19** via analytical HPLC over a chiral stationary phase and the features were assigned to the respective enantiomers. After separation, first insights into their chiroptical properties were derived from the CD spectra with respectable g_Abs_‐values of around 10^−3^. Even though many more aspects of superhelicenes, on which we did not touch in the current work, but are subject to future studies, we did show that these superhelicenes are a versatile, easily tunable platform to obtain a great variety of π‐extended helicenes. We like to mention here detailed chiroptical properties, more efficient enantiomeric separation, and spintronic applications. We are confident that our work will help to unleash the full potential of chiral, helical nanographenes and bring them a step closer to applications via their on‐demand tunability.

## Conflict of interest

The authors declare no conflict of interest.

## Supporting information

As a service to our authors and readers, this journal provides supporting information supplied by the authors. Such materials are peer reviewed and may be re‐organized for online delivery, but are not copy‐edited or typeset. Technical support issues arising from supporting information (other than missing files) should be addressed to the authors.

Supporting Information
