# Supplementary material for: A Family of Superhelicenes: Easily Tunable, Chiral Nanographenes by Merging Helicity with Planar π Systems
Source: Angew Chem Int Ed Engl. 2021 Jul 9;60(33):18073–81. doi: 10.1002/anie.202103253 (PMC8456895; doi:10.1002/anie.202103253)
Supplement: Supplementary file 1 — Supporting Information [file ANIE-60-18073-s001.pdf]

Angewandte Chemie
Eine Zeitschrift der Gesellschaft Deutscher Chemiker

Supporting Information

A Family of Superhelicenes: Easily Tunable, Chiral Nanographenes by Merging Helicity with Planar π Systems

David Reger[+], Philipp Haines[+], Konstantin Y. Amsharov, Julia A. Schmidt, Tobias Ullrich, Simon Bönisch, Frank Hampel, Andreas Görling, Jenny Nelson, Kim E. Jelfs, Dirk M. Guldi, and Norbert Jux**

anie_202103253_sm_miscellaneous_information.pdf

Table of contents

1 General information **2**

2 Synthesis **4**

3 Physicochemical data **27**

4 X-Ray data **44**

4.1 Data for fluorenone-superhelicene 16 *44*

5 Charge carrier mobilities **46**

5.1 Methods *46*

5.2 Mobility calculation procedure *47*

5.3 Results *48*

6 Enantiomeric separation **53**

7 Assignment of the enantiomers **54**

7.1 Computational details *54*

7.2 Calculated structural features *56*

7.3 Comparison and assignment of measured and calculated spectra *58*

8 Calculations of frontier molecular orbitals (FMOs) **63**

9 Spectra appendix for NMR spectroscopy and MS spectrometry **66**

10 Literature **130**

1 General information

The general information for synthesis and physicochemical investigations can be found here. The general information on theoretical chemical calculations can be found within the respective chapters. All chemicals were purchased from Sigma-Aldrich and used without any further purification. Solvents were distilled prior to usage. CH_2Cl_2, $CHCl_3$ and EtOAc were distilled from K_2CO_3 prior to usage. Thin layer chromatography (TLC) was performed on Merck silica gel 60 F254, detected by UV-light (254 nm, 366 nm). Column chromatography and flash column chromatography were performed on Macherey Nagel silica gel 60 M (230–400 mesh, 0.04–0.063 mm). NMR spectroscopy was performed on a JEOL JNM EX 400 (^{1}H: 400 MHz, ^{13}C: 100 MHz), a JEOL JNM GX 2 400 (^{1}H: 400 MHz, ^{13}C: 100 MHz), a Bruker Avance 400 (^{1}H: 400 MHz, ^{13}C: 100 MHz) or a Bruker Avance 300 (^{1}H: 300 MHz, ^{13}C: 75 MHz) instrument. Deuterated solvents were purchased from Sigma Aldrich and used as received. Chemical shifts are referenced to residual protic residues in the solvents ($CHCl_3$: ^{1}H: 7.26 ppm; $C_2D_2Cl_4$: 5.91 ppm, DMSO-d_6: 2.50 ppm) or the deuterated solvent itself ($CDCl_3$: ^{13}C; 77.0 ppm). The resonance multiplicities are indicated as “s“ (singlet), “d” (doublet), “t” (triplet), “q” (quartet) and “m” (multiplet). Signals referred to bs (broad singlet) are not clearly resolved or significantly broadened. *Para*-substituted phenylrings with an AA’BB’ spin system are termed as duplets for simplicity even though we are aware that these spin systems give spectra of higher order. HPLC separations were performed on a Shimadzu Prominence Liquid ChromatographLC-20AT with communication bus module CBM-20A, diode array detector SPDM20A, degassing unit DGU-20A5 R, column oven CTO-20AC or CTO-20A, respectively and with auto sampler SIL-20A HT. A Chiralpak IA column from Chiral Technologies (Daicel) was used. LDI/MALDI-ToF mass spectrometry was performed on a Shimazu AXIMA Confidence (nitrogen UV-laser, 337 nm) or a Bruker ultrafleXtreme (nitrogen UV-laser, 337 nm) instrument. In case of MALDI, the following matrix were used: 2,5-dihydroxybenzoic acid (DHB), sinapic acid (SIN) or trans-2-[3-(4-tert-butylphenyl)-2-methyl-2-propenylidene]malononitrile (DCTB). High resolution mass spectrometry was performed on an ESI-ToF mass spectrometer Bruker maXis 4G UHR MS/MS, a Bruker micrOTOF II focus TOF MS or a Bruker ultrafleXtreme (nitrogen UV-laser, 337 nm) instrument. X-ray diffraction was carried out on an Agilent SuperNova Dual Wavelength Platform with an Atlas CCD Detector or an Agilent Xcalibur E system with

a Ruby CCD Detector. Circular dichroism spectroscopy was carried out on a Jasco J-815 spectrometer. The samples were measured in CH_2Cl_2 at room temperature. Steady-state absorption experiments were measured using a Lambda 2 spectrophotometer from Perkin Elmer. The samples were measured in a 10mm×10mm quartz cuvette. Steady state fluorescence measurements were also carried out in a 10mm×10mm quartz cuvette using a FluoroMax®-3 fluorometer from Horiba Jovin Yvon equipped with a R928P photomultiplier tube from Hamamatsu (185 –850 nm). Time correlated single-photon counting (TCSPC) experiments were recorded with a FluoroLog®-3 lifetime spectrofluorometer from Horiba Jobin Yvon with integrated TCSPC software. The samples were excited at 400 nm by a SuperK Extreme EXB-6 laser from NKT Photonics. The signal was detected by a Hamamatsu MCP photomultiplier (R33809U-50). Each measurement was performed using a 10mm×10mm quartz cuvette. Femtosecond transient absorption spectroscopy measurements were performed using transient absorption pump/probe system EOS from Ultrafast Systems. Laser pulses were generated with an amplified titanium:sapphire CPA-2110 fs laser system (775 nm output, 1 kHz repetition rate, 150 fs pulse width; 200 nJ excitation laser energy) from Clark-MXR Inc. The desired 3excitation at 387 nm was tuned by frequency doubling using a calcium fluoride non-linear optical crystal. The spectral width is detected from 400 to 915 nm by two CCD cameras. The measurements were carried out using a 2mm×10mm quartz cuvette. The transient absorption spectra were recorded with the software EOS from Newport/Ultrafast Systems. Electrochemical measurements, as cyclic voltammetry and differential pulse voltammetry were executed with a three electrode setup. A glassy carbon disk was used as working electrode, an Ag/AgCl was used as reference electrode and a Pt-plate was used as counter electrode. Voltage was controlled with an EG&G Princeton Applied Research potentiostat. The data was recorded with NOVA 1.10 software. Spectroelectrochemistry was conducted with a home-made three neck cell in a Cary 5000 UV/Vis/NIR spectrometer from Varian and the respective processing software. We used a three electrode setup comprising a Pt-gauze as working electrode, a Pt-wire as counter electrode and a silver wire as pseudo reference electrode.

2 Synthesis

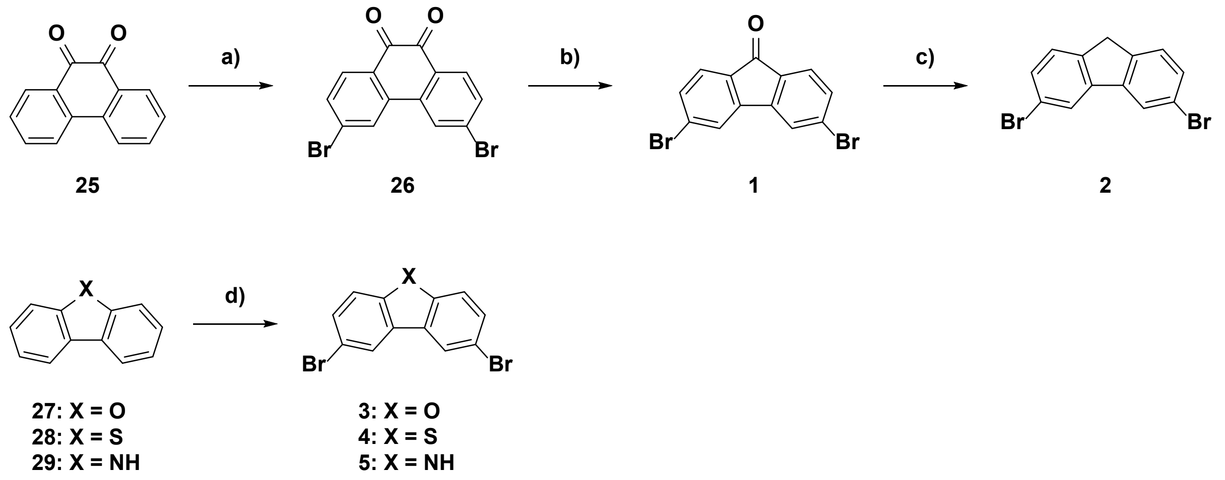

Scheme S1. Synthesis of the dibrominated precursors either by direct bromination (**3**, **4**, **5**) or indirectly via utilization of phenanthrene-9,10-dione (**1**, **2**).

The syntheses of precursors **1**, **2**, **3**, **4**, **5** and **26** were adapted from literature procedures.[S1,S2,S3,S4]

3,6-Dibromophenanthrene-9,10-dione 26

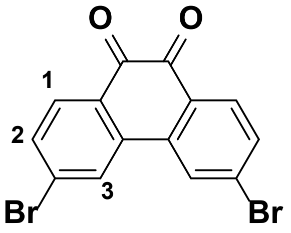

26

A 2 neck round-bottom Schlenk-flask (500 mL) equipped with a magnetic stirring bar a dropping funnel and a reflux condenser was charged with 9,10-phenanthrenquinone (12.5 g, 60.0 mmol) and nitrobenzene (120 mL). The dropping funnel was charged with 2.15 eq. Br_2 (20.5 g, 6.57 mL, 129 mmol) in nitrobenzene (30 mL). The 9,10-phenanthrenquinone suspension was degassed by bubbling N_2 through it for 15 min. 10 mol% dibenzoyl peroxide (moistened with water 25 wt%) (1.95 g, 6.00 mmol) was added in one portion. The Br_2 solution was added dropwise over 25 min at rt. After complete addition the reaction mixture was heated in steps of 20 °C to 110 °C. The dark mixture was stirred for 17 h at 110 °C. After cooling to rt. ethanol (250 mL) was added and an orange solid precipitated. The suspension was cooled to 4 °C in the fridge for 30 min. It was filtered off through a glass-frit (P4) and the solid was washed with an excess of ethanol until the filtrate remained colorless. The solid was dried under vacuum. The product was obtained as an orange solid with a yield of 65 % (14.2 g, 38.8 mmol). REMARK: When the reaction was carried out on a smaller scale the yields were generally higher (80 % yield on a scale of 4.00 mmol of 9,10-phenanthrenquinone).

^{1}H NMR (CDCl$_3$, 400 MHz, rt.): δ [ppm] = 8.12 (d, ^{4}J = 1.7 Hz, 2H, 3); 8.07 (d, ^{3}J = 8.3 Hz, 2H, 1), 7.67 (dd, ^{3}J = 8.3 Hz, ^{4}J = 1.7 Hz, 2H, 2).

^{13}C NMR (CDCl$_3$, 100 MHz, rt.): δ [ppm] = 178.8, 135.9, 133.4, 132.10, 132.09, 129.8, 127.4.

3,6-Dibromofluorenone 1

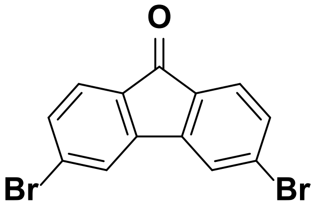

1

A round-bottom flask (250 mL) equipped with a magnetic stirring bar and a reflux condenser was charged with KOH (8.75 g, 156 mmol) and water (20 mL). The KOH solution was heated to reflux and 3,6-dibromophenanthrene-9,10-dione **26** (4.39 g, 12.0 mmol) was added in one portion. It was heated for 30 min and turned dark during that time. 5.5 eq. $KMnO_4$ (10.4 g, 66.0 mmol) were added in 4 portions over 40 min at reflux. Subsequently the reaction mixture was refluxed for further 1 h. It was allowed to cool to rt. and acidified with H_2SO_4. $Na_2S_2O_5$ was added until the reaction mixture turned yellow. The aqueous phase which contained a fast amount of inorganic salts was extracted with portions of CH_2Cl_2 (a large amount of CH_2Cl_2 necessary) until the CH_2Cl_2 portions did not contain solids anymore. The combined organic phases were dried over Na_2SO_4. The amount of CH_2Cl_2 was reduced to approximately 20 mL and a yellow solid precipitated. The solid was filtered off through a glass-frit (P4) and the solid was washed with an excess of hexanes. The product was obtained as a yellow solid in a yield of 33 % (1.34 g, 3.96 mmol). The product was almost insoluble in all common organic solvents. Therefore, the product was not analyzed by NMR spectroscopy or mass spectrometry.

3,6-Dibromofluorene 2

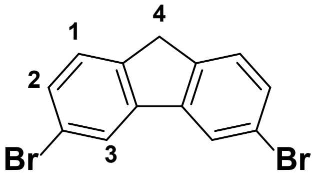

2

A round-bottom Schlenk-flask equipped with a magnetic stirring bar and a reflux condenser was charged with 3,6-dibromofluorenone **1** (1.95 g, 5.77 mmol) and ethylene glycol (80 mL). The suspension was degassed by N_2-bubbling for 30 min. 5 eq. Hydrazine-monohydrate (1.45 g, 1.41 mL, 28.9 mmol) was added and the suspension was stirred for 16.5 h at 100 °C. A solution of 5.5 eq. KOH (1.78 g, 31.7 mmol) in water (4.50 mL) was at 100 °C and the reaction mixture was heated further to 130 °C for 2 h. During that time it turned into an orange solution. It was allowed to cool to rt. and water (100 mL) was added. An orange solid precipitated which was filtered off through a glass-frit (P4). The solid was taken up in

CH_2Cl_2 (100 mL) and water (50 mL) was added. The phases were separated and the aqueous phase was extracted with CH_2Cl_2 (2x50 mL). The combined organic phases were washed brine (50 mL) and dried over Na_2SO_4. The crude product was purified via column chromatography over silica gel with CH_2Cl_2. The product was obtained as a pale yellow solid in a yield of 82 % (1.53 g, 4.72 mmol).

^{1}H NMR ($CDCl_3$, 400 MHz, rt.): δ [ppm] = 7.82 (d, ^4J = 1.7 Hz, 2H, 3); 7.42 (dd, ^3J = 8.0 Hz, ^4J = 1.8 Hz, 2H, 2), 7.37 (d, ^4J = 8.0 Hz, 2H, 1), 3.75 (s, 2H, 4).

^{13}C NMR ($CDCl_3$, 100 MHz, rt.): δ [ppm] = 142.5, 142.1, 130.2, 126.5, 123.3, 120.9, 36.2.

2,8-Dibromodibenzofuran 3

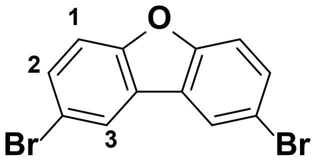

3

A round-bottom flask (100 mL) equipped with a magnetic stirring bar and a dropping funnel was charged with dibenzofuran (3.36 g, 20.0 mmol) with was dissolved in glacial acetic acid (40 mL). The dropping funnel was charged with 2.1 eq. Br_2 (6.71 g, 2.15 mL, 42.0 mmol) in glacial acetic acid (12 mL). The Br_2 solution was added dropwise over 30 min at rt. The reaction mixture was stirred for 18.5 h at rt. and for additional 1.5 h at 80 °C. Meanwhile a white solid precipitated. After cooling to rt. ethanol (50 mL) was added and the solid was filtered off through a glass-frit (P4). It was washed with an excess of water, ethanol (30 mL) and hexanes (30 mL) and was dried under vacuum. The product was obtained as a white solid with a yield of 34 % (2.23 g, 6.84 mmol).

^{1}H NMR ($CDCl_3$, 400 MHz, rt.): δ [ppm] = 8.01 (d, ^4J = 1.9 Hz, 2H, 3), 7.57 (dd, ^3J = 8.7 Hz, ^4J = 2.0 Hz, 2H, 2), 7.44 (d, ^4J = 8.7 Hz, 2H, 1).

^{13}C NMR ($CDCl_3$, 100 MHz, rt.): δ [ppm] = 155.3, 130.7, 125.1, 123.7, 115.9, 113.4.

2,8-Dibromodibenzothiophene 4

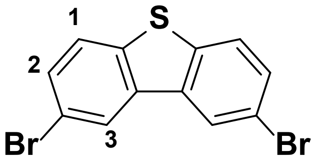

4

A round-bottom flask (250 mL) equipped with a magnetic stirring bar and a dropping funnel was charged with dibenzofuran (9.21 g, 50.0 mmol) which was dissolved in $CHCl_3$ (50 mL). The clear solution was cooled to 0 °C in an ice-bath. The dropping funnel was charged with 2.2 eq. Br_2 (17.6 g, 5.64 mL, 110 mmol) in $CHCl_3$ (50 mL). The Br_2 solution was added dropwise

over 30 min at 0 °C. The ice-bath was removed and the dark solution was stirred for 20 h at rt. A solid precipitated during that time. Methanol (25 mL) was added and the suspension was cooled to 4 °C in the fridge. The solid was filtered off through a glass-frit (P4) and was washed with an excess of methanol until the filtrate remained colorless. The solid was dried under vacuum. The product was obtained as a white solid with a yield of 57 % (9.64 g, 28.2 mmol).

^{1}H NMR ($CDCl_3$, 400 MHz, rt.): δ [ppm] = 8.24 (d, ^4J = 1.8 Hz, 2H, 3), 7.71 (d, ^3J = 8.5 Hz, 2H, 1), 7.58 (dd, ^3J = 8.5 Hz, ^4J = 1.9 Hz, 2H, 2).

^{13}C NMR ($CDCl_3$, 100 MHz, rt.): δ [ppm] = 138.6, 136.1, 130.3, 124.7, 124.2, 118.6.

3,6-Dibromocarbazole 5

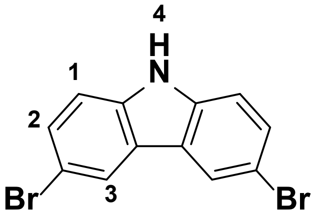

5

A round-bottom flask (50 mL) equipped with a magnetic stirring bar and a dropping funnel was charged with carbazole (0.50 g, 3.00 mmol) which was dissolved in DMF (3 mL).The solution was cooled to 0 °C in an ice-bath. The dropping funnel was charged with a solution of 2.05 eq. *N*-bromosuccinimide (1.09 g, 6.15 mmol) in DMF (6 mL). The *N*-bromosuccinimide solution was added dropwise over 15 min at 0 °C. The reaction mixture was stirred for 1.5 hat 0 °C and for additional 1 h at rt. The reaction mixture was poured onto water and a white solid precipitated immediately. The solid was filtered off through a glass-frit (P4) and was washed with an excess of water. The solid was dried under vacuum. The product was obtained as a white solid with a yield of 97 % (0.94 g, 2.89 mmol).

^{1}H NMR ($CDCl_3$, 400 MHz, rt.): δ [ppm] = 11.58 (s, 1H, 4), 8.42 (d, ^4J = 1.7 Hz, 2H, 3); 7.53 (dd, ^3J = 8.6 Hz, ^4J = 1.9 Hz, 2H, 2), 7.47 (d, ^4J = 8.6 Hz, 2H, 1).

^{13}C NMR ($CDCl_3$, 100 MHz, rt.): δ [ppm] = 138.8, 128.7, 123.4, 123.3, 113.2, 111.0.

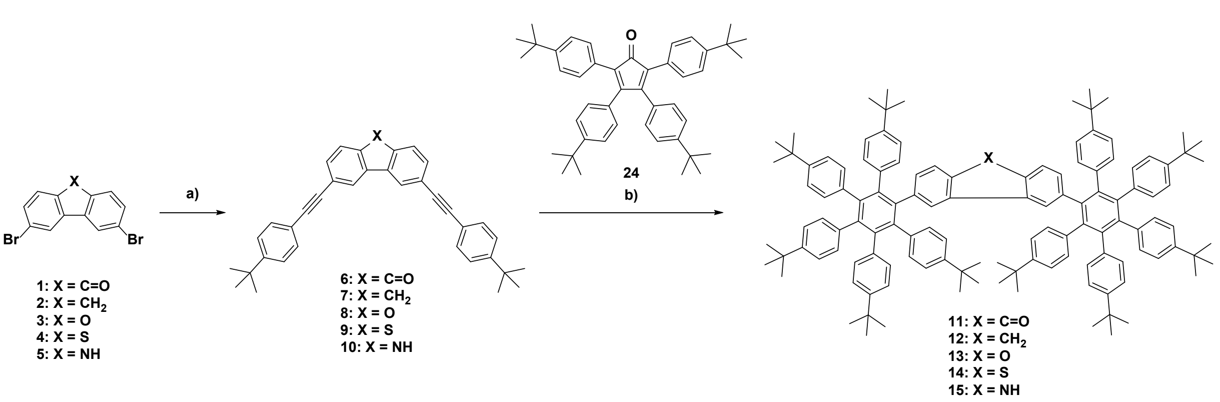

Scheme S2. Synthesis of the bis-acetylene precursor **6-10** and utilization of the latter for Diels-Alder reaction with **24** to give bis-HABs **11-16**.

Tetracyclone **24** which was used for the Diels-Alder reactions was synthesised according to literature procedures.[S5,S6,S7]

3,6-Bis((4-(*tert*-butyl)phenyl)ethynyl)fluorenone 6

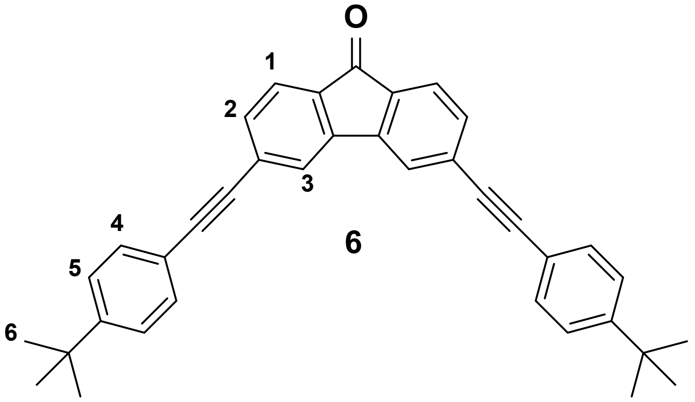

A pressure-vial (25 mL) equipped with a magnetic stirring bar was charged with 3,6-dibromofluorenone **1** (0.34 g, 1.00 mmol), 5 mol% $Pd(PPh_3)_4$ (57.8 mg, 0.05 mmol), 5 mol% CuI (9.52 mg, 0.05 mmol), DMF (8.30 mL) and triethylamine (1.70 mL). The suspension was degassed by vacuum-ultrasound (3x1 min)/N_2-cycles. 2.2 eq. 4-*tert*-butylphenylacetylene (0.35 g, 0.39 mL, 2.20 mmol) was added and the vial was closed. The reaction mixture was stirred for 19 h at 90 °C. After cooling to rt. the reaction mixture was diluted with CH_2Cl_2 and filtered over a pad of silica gel with CH_2Cl_2. The brown solid which was obtained was suspended in CH_2Cl_2 and ethanol (30 mL) was added. The suspension was cooled to 4 °C in the fridge and then filtered off through a glass-frit (P4). It was washed with an excess of ethanol until the filtrate remained colorless. The product was dried under vacuum and was obtained as a bright yellow solid in a yield of 75 % (0.37 g, 0.75 mmol). Due to the low solubility of the compound in common organic solvents it was only analyzed by ^{1}H NMR and mass spectrometry.

^{1}H NMR ($C_2D_2Cl_4$ 400 MHz, rt.): δ [ppm] = 7.64 (s, 2H, 3), 7.57 (d, ^3J = 7.7 Hz, 2H, 1), 7.45 (d, ^3J = 8.5 Hz 4H, 4), 7.42 (dd, ^3J = 7.7 Hz, ^4J = 1.2 Hz, 2H, 2), 7.34 (d, ^3J = 8.5 Hz, 4H, 5), 1.26 (s, 18H, 6).

MS (MALDI-ToF, dhb): m/z = 493 $[M+H]^+$

HRMS (APPI, toluene/CH_2Cl_2): m/z (calculated for $C_{37}H_{32}O$) = 492.2448 $[M]^+$

m/z (found) = 492.2455 $[M]^+$

error [ppm] = -1.5

3,6-Bis((4-(*tert*-butyl)phenyl)ethynyl)fluorene 7

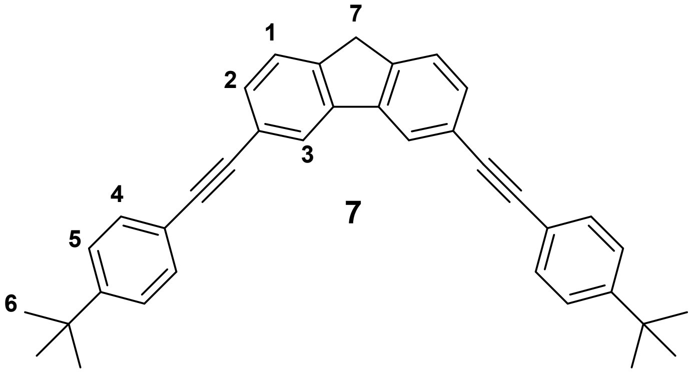

A round-bottom pressure-flask (100 mL) equipped with a magnetic stirring bar was charged with 3,6-dibromofluorene **2** (0.65 g, 2.00 mmol)), 5 mol% $Pd(PPh_3)_4$ (0.12 g, 0.10 mmol), 5 mol% CuI (19.0 mg, 0.10 mmol), DMF (16.6 mL) and triethylamine (3.40 mL). The suspension was degassed by vacuum-ultrasound (5x1 min)/N_2-cycles. 2.2 eq. 4-*tert*-butylphenylacetylene (0.70 g, 0.80 mL, 4.40 mmol) was added and the flask was closed. The reaction mixture was stirred for 19 h at 90 °C. After cooling to rt. the reaction mixture was diluted with CH_2Cl_2 and filtered over a pad of silica gel with CH_2Cl_2. Further purification was achieved by column chromatography over silica gel with hexanes/CH_2Cl_2 2/1. The remaining solid was dried under vacuum and the final product was obtained as a pale orange solid in a yield of 75 % (0.37 g, 0.75 mmol).

^{1}H NMR ($CDCl_3$, 400 MHz, rt.): δ [ppm] = 7.97 (s, 2H, 3), 7.53-7.51 (m, 8H, 1/2/4), 7.40 (d, ^3J = 8.5 Hz, 4H, 5), 3.93 (s, 2H, 7), 1.35 (s, 18H, 6).

^{13}C NMR ($CDCl_3$, 100 MHz, rt.): δ [ppm] = 151.5, 143.3, 141.2, 131.4, 130.5, 125.4, 125.0, 123.2, 122.1, 120.3, 89.2, 89.1, 37.1, 34.8, 31.2.

MS (MALDI-ToF, dhb): m/z = 478 $[M]^+$

HRMS (APPI, toluene): m/z (calculated for $C_{37}H_{34}$) = 478.2655 $[M]^+$

m/z (found) = 478.2659 $[M]^+$

error [ppm] = -0.8

2,8-Bis((4-(*tert*-butyl)phenyl)ethynyl)dibenzofuran 8

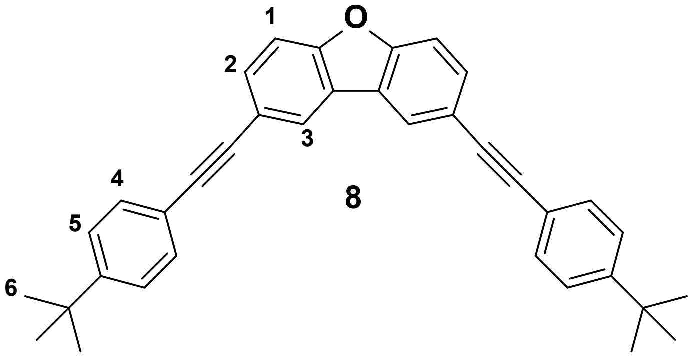

A pressure-vial (25 mL) equipped with a magnetic stirring bar was charged with 2,8-dibromodibenzofuran **3** (0.65 g, 2.00 mmol), 5 mol% $Pd(PPh_3)_4$ (0.12 g, 0.10 mmol), 5 mol% CuI (19.0 mg, 0.10 mmol), DMF (13.3 mL) and triethylamine (2.70 mL). The suspension was degassed by N_2-bubbling for 20 min. 2.2 eq. 4-*tert*-butylphenylacetylene (0.70 g, 0.80 mL, 4.40 mmol) was added and the vial was closed. The dark reaction mixture was stirred for 16 h at 90 °C. After cooling to rt. the mixture was diluted with CH_2Cl_2 and filtered over a pad of silica gel with CH_2Cl_2. The crude product was purified by column chromatography over silica gel with hexanes/CH_2Cl_2 4/1. All product containing fractions were collected and the solvents were evaporated. The remaining solid was dissolved in CH_2Cl_2 and reprecipitated by slow addition of methanol (20 mL). After cooling the suspension in the fridge to 4 °C the white solid was filtered off through a glass-frit (P4) and washed with an excess of methanol. The solid was dried under vacuum. The product was obtained as a white solid in a yield of 59 % (0.56 g, 1.17 mmol).

^{1}H NMR ($CDCl_3$, 400 MHz, rt.): δ [ppm] = 8.12 (d, ^4J = 1.3 Hz, 2H, 3); 7.65 (dd, ^3J = 8.5 Hz, ^4J = 1.7 Hz, 2H, 2), 7.55-7.50 (m, 6H, 1/4), 7.40 (d, ^3J = 8.5 Hz, 4H, 5), 1.35 (s, 18H, 6).

^{13}C NMR ($CDCl_3$, 100 MHz, rt.): δ [ppm] = 156.1, 151.5, 131.3, 131.2, 125.4, 124.1, 123.9, 120.2, 118.5, 111.9, 88.9, 88.6, 34.8, 31.2.

MS (MALDI-ToF, dhb): m/z = 480 $[M]^+$ (100 %), 960 $[2M]^+$ (15 %)

HRMS (MALDI-ToF, dctb): m/z (calculated for $C_{36}H_{32}O$) = 480.2448 $[M]^+$

m/z (found) = 480.2435 $[M]^+$

error [ppm] = 2.66

2,8-Bis((4-(*tert*-butyl)phenyl)ethynyl)dibenzothiophene 9

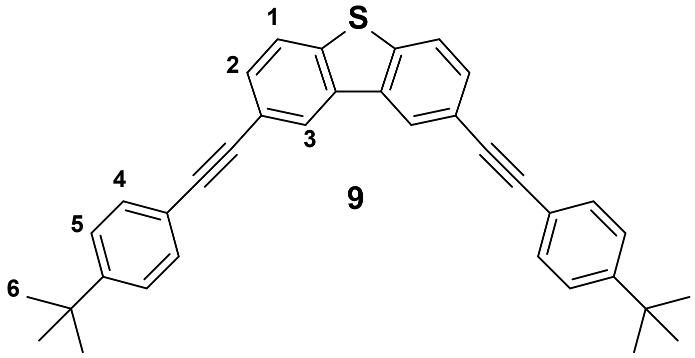

9

A round-bottom pressure-flask (100 mL) equipped with a magnetic stirring bar was charged with 2,8-dibromodibenzothiophene **4** (1.37 g, 4.00 mmol), 5 mol% $Pd(PPh_3)_4$ (0.23 g, 0.20 mmol), 5 mol% CuI (38.1 mg, 0.20 mmol), DMF (33.3 mL) and triethylamine (6.70 mL). The suspension was degassed by vacuum-ultrasound (6x1 min)/N_2-cycles. 2.2 eq. 4-*tert*-butylphenylacetylene (1.39 g, 1.58 mL, 8.80 mmol) was added and the flask was closed. The reaction mixture was stirred for 19.5 h at 90 °C. After cooling to rt. the reaction mixture was diluted with CH_2Cl_2 and filtered over a pad of silica gel with CH_2Cl_2. All solvents were evaporated and the remaining solid was taken up in CH_2Cl_2 (30 mL) again. Methanol (60 mL) was added and the obtained suspension was cooled to 4 °C in the fridge. The solid was filtered off through a glass-frit (P4) and washed with an excess of methanol until the filtrate remained colorless. The solid was dried under vacuum and the product was obtained as an off white solid in a yield of 82 % (1.63 g, 3.28 mmol).

^{1}H NMR ($CDCl_3$, 400 MHz, rt.): δ [ppm] = 8.34 (d, ^4J = 1.3 Hz, 2H, 3), 7.81 (d, ^3J = 8.3 Hz, 2H, 1), 7.62 (dd, ^3J = 8.3 Hz, ^4J = 1.4 Hz, 2H, 2), 7.53 (d, ^3J = 8.4 Hz, 4H, 4), 7.41 (d, ^3J = 8.4 Hz, 4H, 5), 1.35 (s, 18H, 6).

^{13}C NMR ($CDCl_3$, 100 MHz, rt.): δ [ppm] = 151.6, 139.5, 135.1, 131.4, 130.1, 125.4, 124.9, 122.7, 120.2, 120.0, 89.7, 88.7, 34.8, 31.2.

MS (LDI-ToF): m/z = 496 $[M]^+$ (100 %); 992 $[2M]^+$ (55 %)

HRMS (APPI, CH_3CN): m/z (calculated for $C_{36}H_{32}S$) = 496.2219 $[M]^+$

m/z (found) = 496.2228 $[M]^+$

error [ppm] = -1.8

3,6-Bis((4-(*tert*-butyl)phenyl)ethynyl)carbazole 10

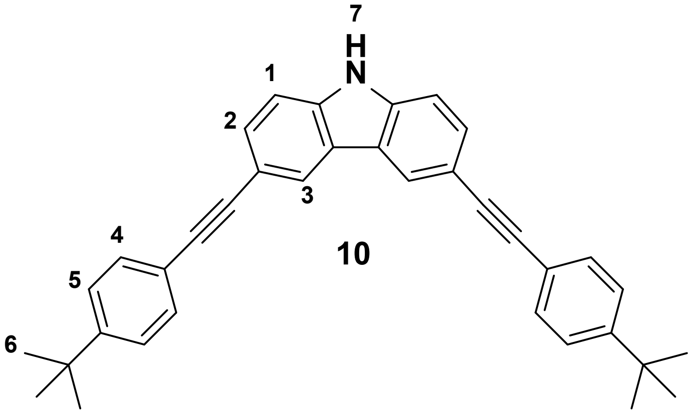

10

A round-bottom Schlenk-flask (50 mL) equipped with a magnetic stirring bar and a reflux condenser was charged with 3,6-dibromocarbazole **5** (0.15 g, 0.46 mmol), 5 mol% $Pd(PPh_3)_4$ (16.1 mg, 23.0 µmol), 10 mol% CuI (8.76 mg, 46.0 µmol), DMF (5.00 mL) and triethylamine (5.00 mL). The suspension was degassed by vacuum-ultrasound (3x1 min)/N_2-cycles. 3 eq. 4-*tert*-butylphenylacetylene (0.22 g, 0.23 mL, 1.38 mmol) was added and the reaction mixture was stirred for 20.5 h at 100 °C. After cooling to rt. all solvents were evaporated and the crude product was filtered over a pad of silica gel with CH_2Cl_2. Final purification was achieved via flash chromatography eluting with a solvent gradient from hexanes to CH_2Cl_2. After drying under vacuum, the product was obtained as a white solid white solid in a yield of 29 % (63.0 mg, 0.13 mmol).

^{1}H NMR ($CDCl_3$, 300 MHz, rt.): δ [ppm] = 8.26 (s, 2H, 3), 8.13 (s, 1H, 7), 7.61 (dd, ^3J = 8.4 Hz, ^4J = 1.4 Hz, 2H, 2), 7.54 (d, ^3J = 8.4 Hz, 4H, 4/5), 7.41 (d, ^3J = 8.4 Hz, 4H, 4/5), 7.35 (d, ^3J = 8.4 Hz, 2H, 1), 1.36 (s, 18H, 6).

^{13}C NMR ($CDCl_3$, 75 MHz, rt.): δ [ppm] = 151.1, 139.3, 131.2, 129.9, 125.3, 124.0, 123.0, 120.7, 114.8, 110.8, 89.7, 88.0, 34.8, 31.2.

MS: No assignable product peak was detected by mass spectrometry.

Bis-HAB-fluorenone 11

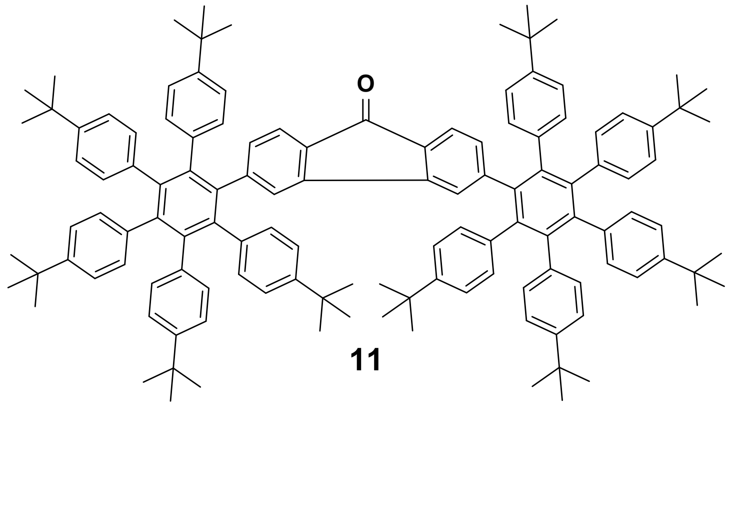

11

A round-bottom pressure-flask (100 mL) equipped with a magnetic stirring bar was charged with 3,6-bis((4-(*tert*-butyl)phenyl)ethynyl)fluorenone **6** (0.15 g, 0.30 mmol), 2.5 eq. 2,3,4,5-tetrakis(4-(*tert*-butyl)phenyl)cyclopenta-2,4-

diene-1-one **24** (0.46 g, 0.75 mmol) and toluene (1.5 mL). The flask was purged with N_2 and closed. The reaction mixture was stirred for 42.5 h at 220 °C. After cooling to rt. CH_2Cl_2 (5 mL) was added to dissolve everything and methanol (20 mL) was added slowly upon stirring. A yellow solid precipitated and the suspension was cooled to 4 °C in the fridge. The solid was filtered off through a glass-frit (P4) and was washed with an excess of methanol until the filtrate remained colourless. The remaining solid was dried under vacuum. The product was obtained as a bright yellow solid with a yield of 87 % (0.43 g, 0.26 mmol).

^{1}H NMR ($CDCl_3$, 400 MHz, rt.): δ [ppm] = 6.99 (d, ^3J = 7.6 Hz, 2H), 6.85-6.9 (m, 20H), 6.70-6.61 (m, 24H), 1.12 (s, 36H), 1.11 (s, 36H), 1.10 (s, 18H).

^{13}C NMR ($CDCl_3$, 100 MHz, rt.): δ [ppm] = 194.4, 148.1, 148.0, 147.6, 147.5, 143.0, 141.3, 141.0, 139.9, 138.8, 137.6, 137.5, 137.1, 132.4, 131.5, 131.2, 131.1, 130.9, 130.7, 123.5, 123.4, 123.2, 123.1, 122.0, 34.1, 34.0, 31.23, 31.18, 31.17.

MS (MALDI-ToF, dctb): m/z = 1653 $[M]^+$

HRMS (APPI, toluene): m/z (calculated for $C_{125}H_{136}O$) = 1653.0586 $[M]^+$

m/z (found) = 1653.0590 $[M]^+$

error [ppm] = -0.2

Bis-HAB-fluorene 12

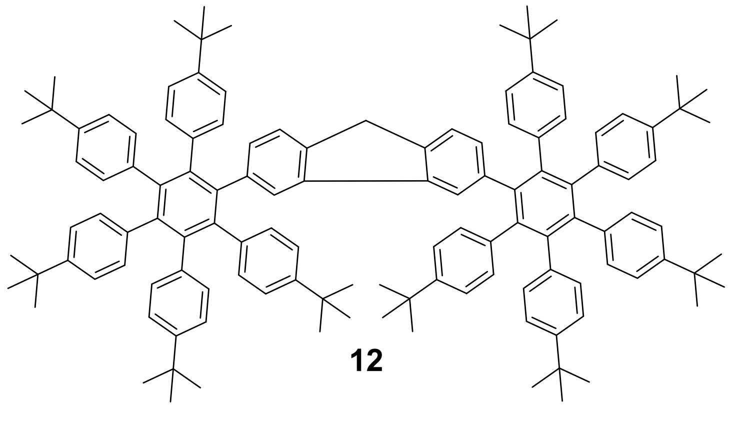

12

A pressure-vial (25 mL) equipped with a magnetic stirring bar was charged with 3,6-bis((4-(*tert*-butyl)phenyl)ethynyl)fluorene **7** (0.15 g, 0.30 mmol), 2.5 eq. 2,3,4,5-tetrakis(4-(*tert*-butyl)phenyl)cyclopenta-2,4-diene-1-one **24** (0.46 g, 0.75 mmol) and toluene (1.5 mL). The vial was purged with N_2 and closed. The reaction mixture was stirred for 43 h at 220 °C. After cooling to rt. methanol (20 mL) was added slowly upon stirring and a brown solid precipitated. The suspension was cooled in the fridge to 4 °C and filtered off through a glass-frit (P4). After washing the remaining feed with an excess of methanol there were still impurities

found in the solid. Therefor the product was further purified by column chromatography over silica gel with hexanes/CH_2Cl_2 2/1. The obtained solid was reprecipitated by addition of methanol (20 mL) to solution in CH_2Cl_2 (5 mL) and cooling to 4 °C. After filtration through a glass-frit (P4) the solid was washed with methanol (30 mL) and dried under vacuum. The product was obtained as a pale yellow solid in a yield of 67 % (0.32 g, 0.26 mmol).

^{1}H NMR ($CDCl_3$, 400 MHz, rt.): δ [ppm] = 6.97 (s, 2H), 6.85-6.64 (m, 44H), 3.41 (s, 2H), 1.11 (s, 54H), 1.09 (s, 36H).

^{13}C NMR ($CDCl_3$, 100 MHz, rt.): δ [ppm] = 147.4, 147.3, 147.2, 140.7, 140.44, 140.43, 140.39, 140.03, 140.02, 138.8, 138.0, 137.8, 131.10, 131.08, 130.2, 123.2, 123.0, 122.9, 122.8, 122.4, 34.03, 34.02, 31.22, 31.20.

MS (MALDI-ToF, dhb): m/z = 1639 $[M]^+$

HRMS (APPI, toluene): m/z (calculated for $C_{125}H_{138}$) = 1639.0793 $[M]^+$

m/z (found) = 1639.0783 $[M]^+$

error [ppm] = 0.6

Bis-HAB-dibenzofuran 13

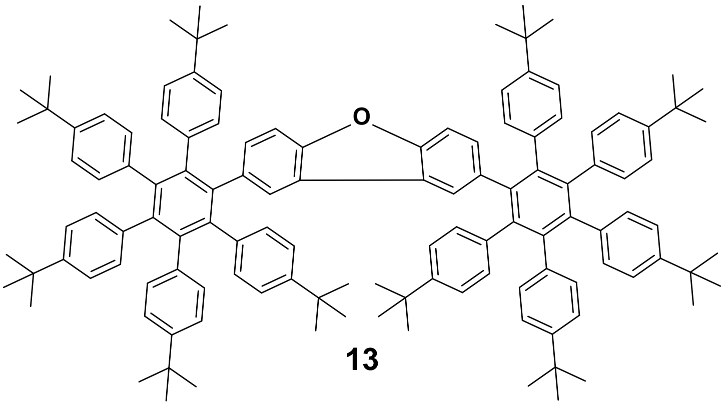

13

A round-bottom pressure-flask (100 mL) equipped with a magnetic stirring bar was charged with 2,8-bis((4-(*tert*-butyl)phenyl)ethynyl)-dibenzofuran **8** (0.19 g, 0.40 mmol), 2.5 eq. 2,3,4,5-tetrakis(4-(*tert*-butyl)phenyl)cyclopenta-2,4-diene-1-one **24** (0.61 g, 1.00 mmol) and toluene (2 mL). The flask was purged with N_2 and closed. The reaction mixture was stirred for 26 h at 220 °C. After cooling to rt. CH_2Cl_2 (5 mL) was added to dissolve everything and methanol (35 mL) was added slowly upon stirring. A grey solid precipitated and the suspension was cooled to 4 °C in the fridge. The solid was filtered off through a glass-frit (P4) and washed with an excess of methanol until the filtrate remained colorless.

The solid was dried under vacuum and the product was obtained as an off white solid with a yield of 93 % (0.61 g, 0.37 mmol).

^{1}H NMR ($CDCl_3$, 400 MHz, rt.): δ [ppm] = 7.14 (d, ^{4}J = 1.3 Hz, 2H), 6.91 (d, ^{3}J = 8.5 Hz, 2H), 6.84-6.60 (m, 42H), 1.11 (s, 54H), 1.08 (s, 36H).

^{13}C NMR ($CDCl_3$, 100 MHz, rt.): δ [ppm] = 154.0, 147.43, 174.41, 147.3, 140.8, 140.7, 140.5, 139.5, 137.9, 137.7, 135.2, 131.1, 131.0, 130.6, 123.3, 123.2, 123.02, 122.95, 109.2, 34.0, 31.2.

HRMS (MALDI-ToF, dctb): m/z (calculated for $C_{124}H_{136}O$) = 1641.0586 $[M]^+$

m/z (found) = 1641.0559$[M]^+$

error [ppm] = 1.65

Bis-HAB-dibenzothiophene 14

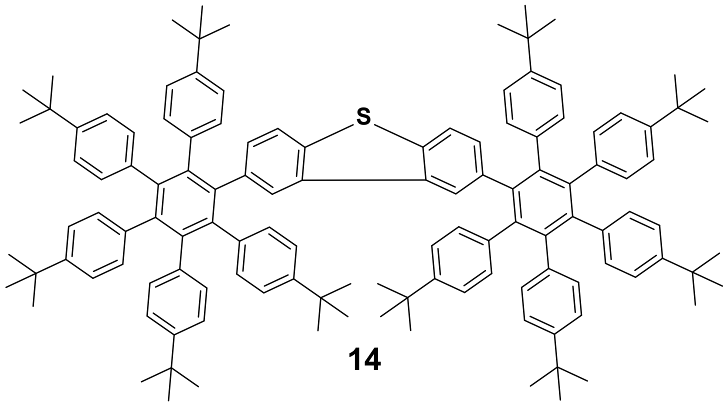

14

A round-bottom pressure-flask (100 mL) equipped with a magnetic stirring bar was charged with 2,8-bis((4-(*tert*-butyl)phenyl)ethynyl)-dibenzothiophene **9** (0.20 g, 0.40 mmol), 2.5 eq. 2,3,4,5-tetrakis(4-(*tert*-butyl)phenyl)cyclopenta-2,4-diene-1-one **24** (0.61 g, 1.00 mmol) and toluene (2 mL). The flask was purged with N_2 and closed. The reaction mixture was stirred for 41 h at 220 °C. After cooling to rt. the dark mixture was diluted with CH_2Cl_2 (5 mL) to dissolve everything. Methanol (40 mL) was added slowly upon stirring and a brownish solid precipitated. The suspension was cooled to approximately 4 °C in the fridge and was filtered off through a glass-frit (P4). The remaining solid was washed with an excess of methanol. As impurities were still found the crude product was dissolved in CH_2Cl_2, adsorbed on silica gel and purified via column chromatography over silica gel with hexanes/CH_2Cl_2 4/1. The obtained white solid was again reprecipitated by addition of methanol (15 mL) to solution in CH_2Cl_2 (5 mL) and cooling to 4 °C. After filtration through a glass-frit (P4) the solid was washed with methanol (30 mL) and dried under

vacuum. The product was obtained as a white solid in a yield of 73 % (0.48 g, 0.29 mmol).

^{1}H NMR ($CDCl_3$, 300 MHz, rt.): δ [ppm] = 7.28 (d, ^4J = 1.8 Hz, 2H), 7.14 (d, ^3J = 8.3 Hz, 2H), 6.84-6.61 (m, 42H), 1.11 (s, 54H), 1.07 (s, 36H).

^{13}C NMR ($CDCl_3$, 75 MHz, rt.): δ [ppm] = 147.52, 147.47, 147.3, 140.9, 140.6, 140.5, 139.3, 138.0, 137.8, 136.9, 136.1, 134.6, 131.2, 131.1, 130.4, 124.7, 123.3, 123.1, 123.0, 120.3, 34.1, 31.2.

MS (MALDI-ToF, dctb): m/z = 1657

HRMS (APPI, MeOH): m/z (calculated for $C_{124}H_{137}S$) = 1658.0436 [M+H]$^+$

m/z (found) = 1658.0435 [M+H]$^+$

error [ppm] = 0.1

Bis-HAB-carbazole 15

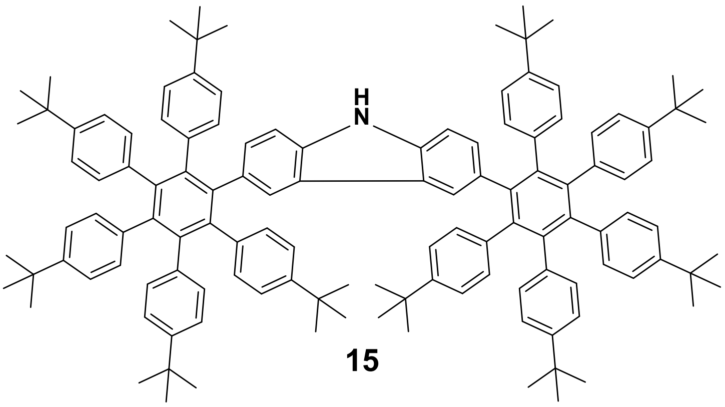

15

A pressure vial (5 mL) equipped with a magnetic stirring bar was charged with **10** (0.10 g, 0.21 mmol), 2.5 eq. 2,3,4,5-tetrakis(4-(*tert*-butyl)phenyl)cyclopenta-2,4-diene-1-one **24** (0.32 g, 0.53 mmol) and toluene. (1.00 mL). The reaction mixture was purged with N_2 and the vial was closed. The reaction mixture was stirred for 131 h at 220 °C. All solvents were evaporated and the crude was filtered over a pad of silica gel with CH_2Cl_2/hexanes 1/1. After removal of the solvents grey solid remained which was dissolved in CH_2Cl_2 (4 mL) and reprecipitated by addition of MeOH (20 mL). The suspension was cooled in the fridge to approx.. 6 °C. The solid was filtered off through a glass-frit (P4) and washed with a small MeOH until the filtrate remained colorless. The product was dried under vacuum and obtained as a white solid in a yield of 67 % (0.23 g, 0.14 mmol).

^{1}H NMR ($CDCl_3$, 400 MHz, rt.): δ [ppm] = 7.25 (s, 2H), 6.82-6.62 (m, 44H), 1.10 (s, 54H), 1.06 (s, 36H).

^{13}C NMR (CDCl$_3$, 100 MHz, rt.): δ [ppm] = 147.3, 147.2, 147.1, 140.71, 140.68, 140.4, 140.3, 138.1, 138.03, 138.01, 137.2, 131.5, 131.1, 129.5, 123.21,123.16, 123.0, 122.5, 108.2, 34.03, 34.01, 33.99, 31.2.

MS (MALDI-ToF, dhb): m/z = 1641 [M]$^+$

HRMS (APPI, toluene): m/z (calculated for $C_{124}H_{137}N$) = 1640.0746 [M]$^+$

m/z (found) = 1640.0756 [M]$^+$

error [ppm] = -0.6

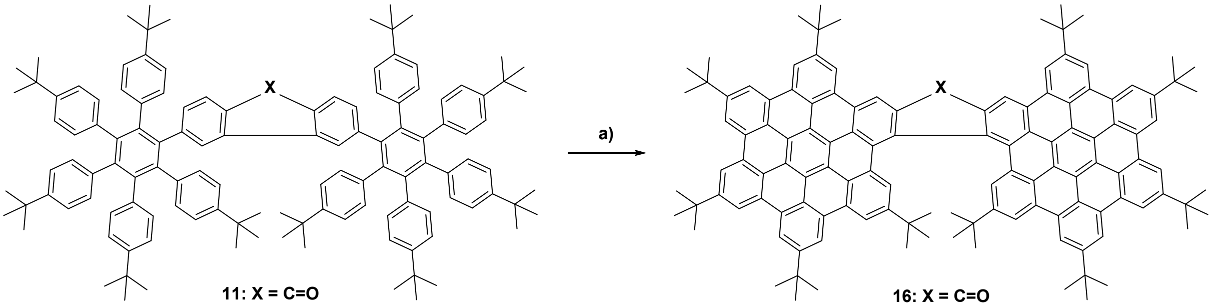

11: X = C=O
12: X = CH$_2$
13: X = O
14: X = S
15: X = NH

16: X = C=O
17: X = CH$_2$
18: X = O
19: X = S
20: X = NH

Scheme S3. Closure of bis-HAB derivative **11**-**15** under oxidative cyclodehydrogenation conditions to give superhelicene products **16**-**20**.

Fluorenone-superhelicene 16

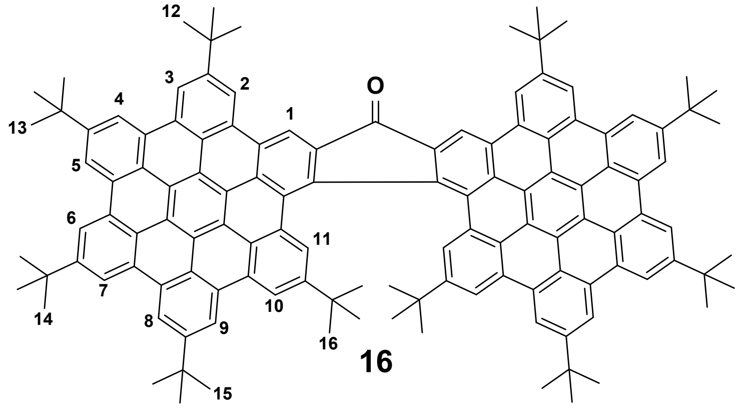

A round-bottom Schlenk-flask (500 mL) equipped with a magnetic stirring bar was charged with **11** (0.40 g, 0.24 mmol) and CH_2Cl_2 (240 mL). The solution was degassed via N_2-bubbling for 30 min and meanwhile cooled to 0 °C. 30 eq. of $FeCl_3$ (1.17 g, 7.20 mmol) dissolved in $MeNO_2$ (3.90 mL) was added portion wise over 20 min via a syringe. Cooling and N_2-bubbling were continued during that time and for further 30 min. Cooling and N_2-bubbling were stopped, the flask was closed under N_2 with a rubber septum and the reaction mixture was stirred for further 2 h at rt. MeOH (100 mL) was added, all solvents were evaporated and the crude was filtered over a pad of silica gel with hexanes/CH_2Cl_2 1/1. Final purification was achieved via column chromatography over silica gel with hexanes/CH_2Cl_2 2/1. The product was

dried under vacuum and obtained as a red-brownish solid in a yield of 96 % (0.37 g, 0.23 mmol).

^{1}H NMR ($CDCl_3$, 400 MHz, rt.): δ [ppm] = 9.84 (s, 2H, 1), 9.59 (s, 2H, 2), 9.48 (s, 2H, 3), 9.42-9.41 (m, 8H, 4/5/6/7), 9.35 (s, 2H, 8), 9.05 (s, 2H, 9), 8.58 (s, 2H, 10), 8.56 (s, 2H, 11), 1.97 (s, 18H, 12), 1.91 (s, 18H, 13/14), 1.90 (s, 18H, 13/14), 1.86 (s, 18H, 15), 0.14 (s, 18H, 16).

^{13}C NMR ($CDCl_3$, 100 MHz, rt.): δ [ppm] = 194.3, 150.0, 149.7, 149.5, 146.9, 143.0, 136.1, 131.8, 131.1, 131.0, 130.9, 130.61, 130.59, 130.56, 130.42, 130.37, 130.35, 129.8, 129.4, 129.3, 124.0, 123.9, 123.82, 123.77, 123.5, 122.5, 121.9, 121.8, 121.6, 121.4, 121.0, 120.4, 120.3, 119.8, 119.7, 119.3, 119.2, 119.1, 119.0, 118.6, 116.8, 36.0, 35.84, 35.80, 35.7, 34.1, 32.2, 32.04, 32.0, 30.0.

MS (MALDI-ToF, dctb): m/z = 1629 $[M]^+$

HRMS (APPI, toluene): m/z (calculated for $C_{125}H_{112}O$) = 1628.8708 $[M]^+$

m/z (found) = 1628.8707 $[M]^+$

error [ppm] = 0.1

Fluorene-superhelicene 17

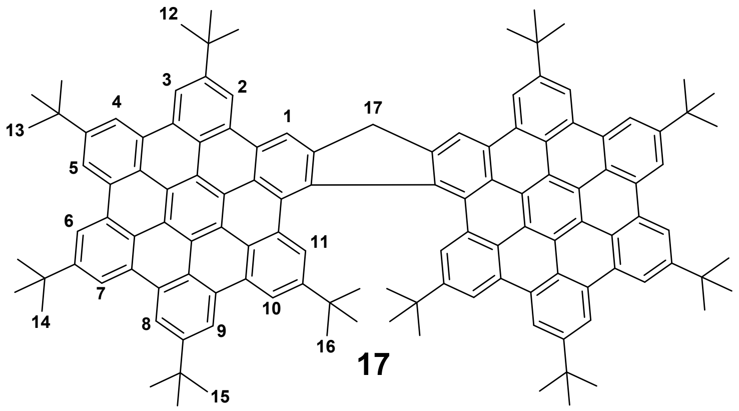

17

A round-bottom Schlenk-flask (250 mL) equipped with a magnetic stirring bar was charged with **12** (0.15 g, 91.4 µmol) and CH_2Cl_2 (90 mL). The solution was degassed via N_2-bubbling for 20 min and meanwhile cooled to 0 °C. 30 eq. of $FeCl_3$ (0.44 g, 2.70 mmol) dissolved in $MeNO_2$ (1.47 mL) was added portion wise over 5 min via a syringe. Cooling and N_2-bubbling were continued during that time and for further 20 min. Cooling and N_2-bubbling were stopped, the flask was closed under N_2 with a rubber septum and the reaction mixture was stirred for further 2 h at rt. MeOH (50 mL) was added, all solvents were evaporated and the crude was filtered over a pad of silica gel with hexanes/CH_2Cl_2 2/1. Final purification was achieved via flash chromatography over an Interchim Si-HP column (40 g, 50 µm) with a solvent

gradient from pure hexanes to hexanes/CH_2Cl_2 5/1. The product was dried under vacuum and obtained as an orange solid in a yield of 64 % (94.0 mg, 58.2 µmol).

^{1}H NMR ($CDCl_3$, 400 MHz, rt.): δ [ppm] = 9.70 (s, 2H, 1), 9.61 (s, 2H, 2), 9.46 (s, 2H, 3), 9.43-9.41 (m, 8H, 4/5/6/7), 9.35 (s, 2H, 8), 9.07 (s, 2H, 9), 8.59 (d, ^4J = 1.1 Hz, 2H, 10), 8.55 (s, 2H, 11), 5.26 (s, 2H, 17), 1.98 (s, 18H, 12), 1.91 (s, 18H, 13/14), 1.90 (s, 18H, 13/14), 1.86 (s, 18H, 15), 0.07 (s, 18H, 16).

^{13}C NMR ($CDCl_3$, 100 MHz, rt.): δ [ppm] = 149.3, 149.14, 149.12, 149.08, 146.8, 144.4, 137.8, 130.9, 130.8, 130.63, 130.60, 130.57, 130.5, 130.31, 130.29, 130.27, 129.9, 129.3, 128.0, 126.2, 124.3, 124.2, 123.9, 123.8, 123.4, 121.5, 121.2, 120.8, 120.6, 120.5, 120.4, 120.2, 119.4, 119.06, 119.02, 118.94, 118.92, 118.89, 118.2, 117.1, 35.9, 35.80, 35.79, 35.7, 34.0, 32.2, 32.09, 32.08, 32.0, 29.9.

HRMS (MALDI-ToF, dctb): m/z (calculated for $C_{125}H_{114}$) = 1614.8915 $[M]^+$

m/z (found) = 1614.8906 $[M]^+$

error [ppm] = 0.59

Oxa-[7]-superhelicene 18

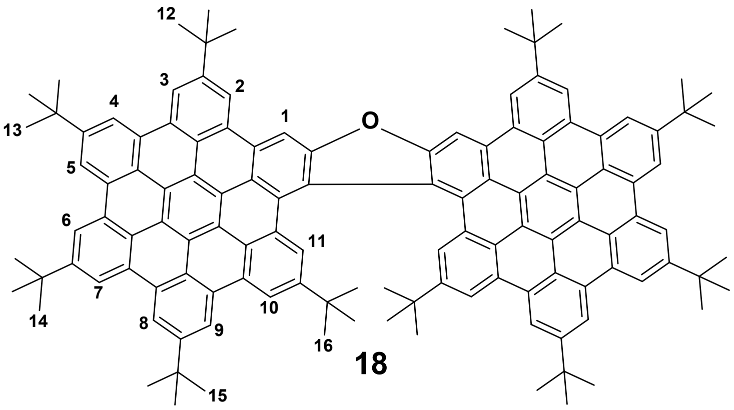

18

A round-bottom Schlenk-flask (500 mL) equipped with a magnetic stirring bar was charged with **13** (0.33 g, 0.20 mmol) and CH_2Cl_2 (200 mL). The solution was degassed via N_2-bubbling for 30 min and meanwhile cooled to 0 °C. 30 eq. $FeCl_3$ (0.97 g, 6.00 mmol) dissolved in $MeNO_2$ (3.23 mL) were added portion wise to the reaction mixture via a syringe over 20 min. Cooling and N_2-bubbling were continued during that time and for further 30 min. Subsequently it was stirred for further 4 h at rt. and finally quenched with MeOH (200 mL). All solvents were evaporated and the crude was filtered over a pad of silica gel with CH_2Cl_2/hexanes 1/1 and afterwards column chromatography over silica gel with CH_2Cl_2/hexanes 1/5. The obtained solid was dissolved in a minimal amount of CH_2Cl_2 and reprecipitated by addition of MeOH (30 mL). After cooling to 6 °C for complete precipitation the orange

solid was filtered off through a glass-frit (P4) and washed with a small amount of MeOH. The product was dried under vacuum and obtained as an orange solid in a yield of 85 % (0.27 g, 0.17 mmol)

Spectroscopical data for **18** are in agreement with the data obtained in our previous publication.[S5]

^{1}H NMR (CDCl$_3$, 400 MHz, rt.): δ [ppm] = 9.79 (s, 2H, 1), 9.63 (s, 2H, 2), 9.50 (s, 2H, 3), 9.44-9.43 (m, 8H, 4/5/6/7), 9.38 (s, 2H, 8), 9.20 (s, 2H, 9), 8.83 (s, 2H, 10), 8.56 (d, ^4J = 1.4 Hz, 2H, 11), 1.97 (s, 18H, 12), 1.91 (s, 36H, 13/14), 1.87 (s, 18H, 15), 0.22 (s, 18H, 16).

^{13}C NMR (CDCl$_3$, 100 MHz, rt.): δ [ppm] = 157.9, 149.5, 149.3, 149.24, 149.22, 147.4, 131.4, 130.8, 130.74, 130.69, 130.6, 130.5, 130.2, 129.9, 129.5, 127.9, 124.3, 124.2, 124.0, 123.9, 123.7, 123.2, 122.3, 121.0, 120.6, 120.5, 120.4, 120.1, 119.9, 119.7, 119.3, 119.2, 119.1, 119.0, 118.7, 103.8, 35.9, 35.82, 35.75, 34.3, 32.2, 32.10, 32.08, 32.0, 30.1.

MS (MALDI-ToF, dctb): m/z = 1617

HRMS (APPI, toluene): m/z (calculated for $C_{124}H_{112}O$) = 1616.8708 [M]$^+$

m/z (found) = 1616.8730 [M]$^+$

error [ppm] = -1.4

Thia-[7]-superhelicene 19

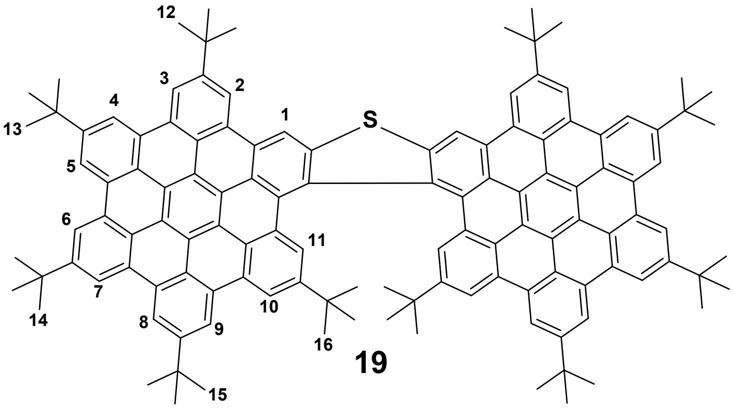

19

A Schlenk-tube (100 mL) equipped with a magnetic stirring bar was charged with bis-HAB-dibenzothiophene **14** (49.8 mg, 30.0 µmol). The precursor was dissolved in CH_2Cl_2. The solution was degassed by bubbling N_2 through it for 15 min. Meanwhile it was cooled to 0 °C in an ice bath. A solution of 30 eq. anhydrous $FeCl_3$ (0.15 g, 0.90 mmol) in $MeNO_2$ (0.5 mL) was added via a syringe in one portion. Cooling and N_2 bubbling were continued for 10 min. The tube

was closed with a rubber septum and the reaction mixture was stirred for further 2 h at rt. It was quenched by addition of methanol (30 mL). All solvents were evaporated and the crude product was filtered over pad over silica gel with CH_2Cl_2. Final purification was achieved via column chromatography over silica gel with hexanes/CH_2Cl_2 5/1. The product was dried under vacuum and obtained as a bright orange solid in a yield of 70 % (34.0 mg, 20.9 µmol).

^{1}H NMR ($CDCl_3$, 400 MHz, rt.): δ [ppm] = 9.92 (s, 2H, 1), 9.56 (s, 2H, 2), 9.45 (s, 2H, 3), 9.41-9.39 (m, 8H, 4/5/6/7), 9.32 (s, 2H, 8), 8.96 (s, 2H, 9), 8.64 (s, 2H, 10), 8.43 (s, 2H, 11), 1.95 (s, 18H, 12), 1.88 (s, 36H, 13/14), 1.80 (s, 18H, 15), -0.02 (s, 18H, 16).

^{13}C NMR ($CDCl_3$, 100 MHz, rt.): δ [ppm] = 149.5, 149.23, 149.18, 147.0, 140.7, 130.8, 130.7, 130.63, 130.58, 130.57, 130.44, 130.36, 130.3, 130.0, 129.8, 129.7, 129.4, 129.1, 124.5, 124.2, 124.1, 124.0, 123.7, 123.2, 120.9, 120.80, 120.78, 120.7, 120.51, 120.45, 120.4, 120.3, 119.7, 119.6, 119.1, 119.01, 118.98, 118.9, 118.7, 118.4, 115.2, 35.80, 35.79, 35.7, 33.9, 32.2, 32.1, 32.0, 29.9.

MS (MALDI-ToF, dctb): m/z = 1633 $[M]^+$

HRMS (MALDI-ToF, dctb): m/z (calculated for $C_{124}H_{112}S$) = 1632.8479 $[M]^+$

m/z (found) = 1632.8459 $[M]^+$

error [ppm] = 1.26

Aza-[7]-superhelicene 20

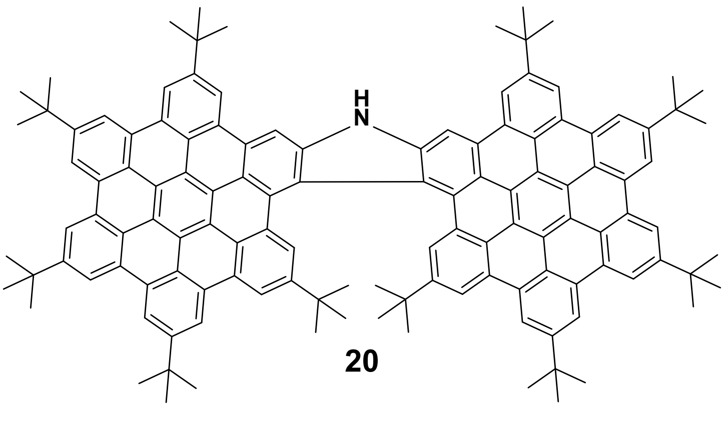

20

A Schlenk-tube (100 mL) equipped with a magnetic stirring bar was charged with **15** (0.10 g, 0.06 mmol), 12 eq. DDQ (0.16 g, 0.72 mmol) and CH_2Cl_2 (40 mL). The mixture was degassed via N_2-bubbling for 20 min and meanwhile cooled to 0 °C. 28 eq. of triflic acid (0.25 g, 0.15 mL, 1.68 mmol) were added in one portion and the tube was closed with a rubber septum under N_2. The reaction mixture was stirred for 30 min at 0 °C and for additional 2.5 h at rt. MeOH (30 mL) and NEt_3 (10 mL) were added. All solvents were evaporated and the crude was filtered over a pad of silica gel

with hexanes/CH_2Cl_2 1/1. Final purification was achieved by column chromatography over silica gel with hexanes/CH_2Cl_2 2/1. The product was dried under vacuum and obtained as an orange solid in a yield of 79 % (76.0 mg, 47.0 mmol). REMARK: The product is unstable under ambient conditions. It is assumed that the *π*-system becomes to electron rich with the strongly electron donating N-H unit and is therefore easily oxidized. Similar was observed already for amino-HBC[S8] Therefore, the obtained yield probably contains decomposition products already. No clean NMR could be obtained however the existence of the product was confirmed by mass spectrometry.

MS (MALDI-ToF, dctb): m/z = 1616 $[M]^+$

HRMS (APPI, toluene): m/z (calculated for $C_{124}H_{113}N$) = 1615.8868 $[M]^+$

m/z (found) = 1615.8851 $[M]^+$

error [ppm] = 1.0

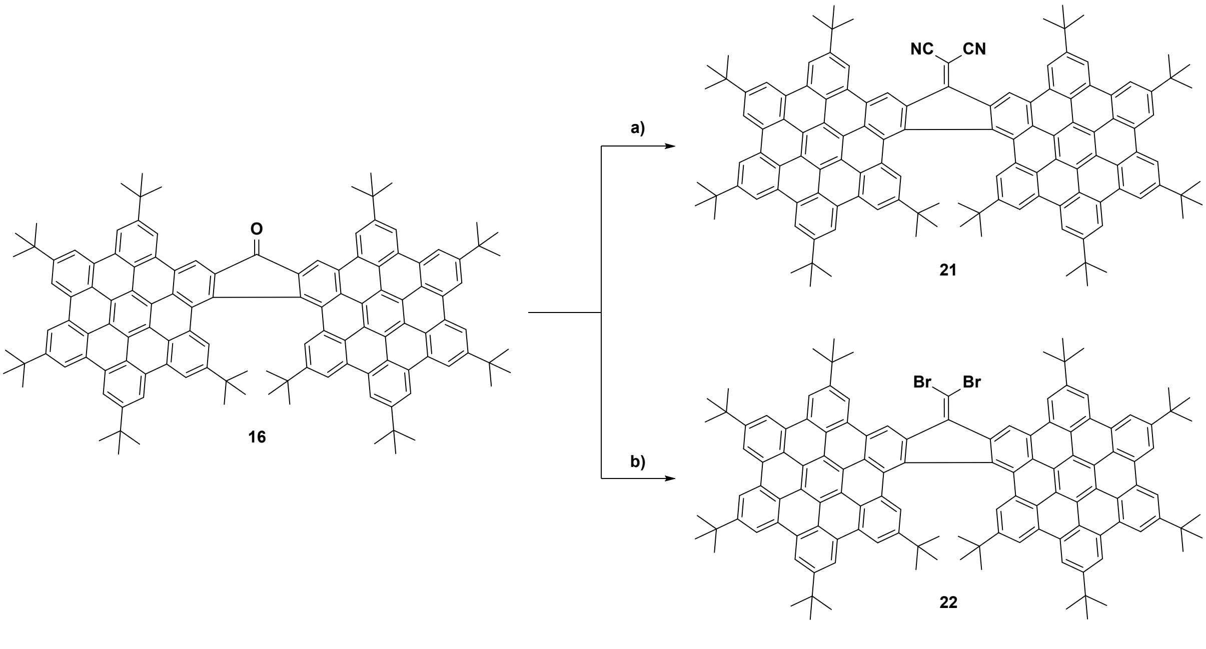

Scheme S4. Post-functionalization of superhelicene **16** to give di-cyano derivative **21** and di-bromo derivative **22**.

Dicyano-superhelicene 21

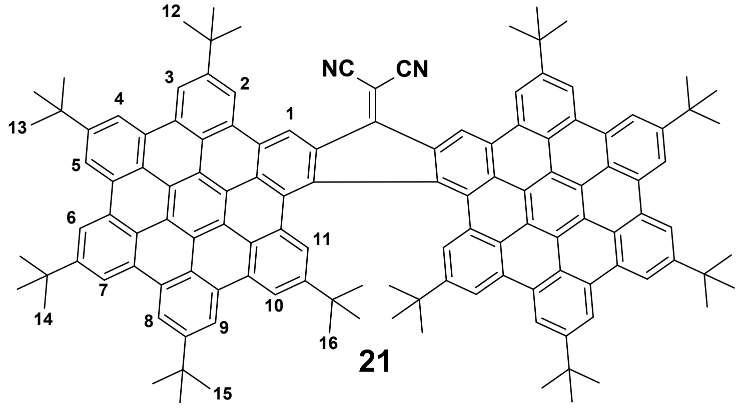

21

A reaction-tube (10 mL) equipped with a magnetic stirring bar and a reflux condenser was charged with **16** (48.9 mg, 30.0 µmol) pyridine (2 mL) and 15 eq. malononitrile (29.7 mg, 25.0 µL, 0.45 mmol). The suspension was stirred for 1.5 h at 80 °C. All solvents were evaporated and the crude product was purified by column chromatography over silica gel with hexanes/CH_2Cl_2 3/2. The product was dried under vacuum and obtained as a black solid in a yield of 96 % (48.0 mg, 28.6 µmol).

^{1}H NMR ($CDCl_3$, 400 MHz, rt.): δ [ppm] = 10.70 (s, 2H, 1), 9.54 (s, 2H, 2), 9.44 (s, 2H, 3), 9.39-9.36 (m, 8H, 4/5/6/7), 9.31 (s, 2H, 8), 8.98 (s, 2H, 9), 8.50 (s, 2H, 10), 8.47 (d, ^4J = 1.6 Hz, 2H, 11), 1.91 (s, 18H, 12), 1.87 (s, 18H, 13/14), 1.87 (s, 18H, 13/14), 1.82 (s, 18H, 15), 0.09 (s, 18H, 16).

^{13}C NMR ($CDCl_3$, 100 MHz, rt.): δ [ppm] = 162.2, 150.4, 149.9, 149.60, 149.57, 146.9, 141.0, 136.0, 131.4, 131.0, 130.7, 130.63, 130.61, 130.5, 130.4, 130.3, 129.8, 129.7, 129.6, 129.5, 124.0, 123.91, 123.87, 123.8, 123.4, 122.4, 122.2, 121.83, 121.81, 121.77, 121.1, 120.4, 119.92, 119.86, 119.6, 119.32, 119.28, 119.1, 119.0, 118.7, 115.6, 72.5, 36.0, 35.9, 35.8, 35.7, 34.1, 32.03, 31.99, 31.97, 29.9.

MS (MALDI-ToF, dctb): m/z = 1677 $[M]^+$

HRMS (MALDI-ToF, dctb): m/z (calculated for $C_{128}H_{112}N_2$) = 1676.8820 $[M]^+$

m/z (found) = 1676.8837 $[M]^+$

error [ppm] = 1.02

Dibromo-superhelicene 22

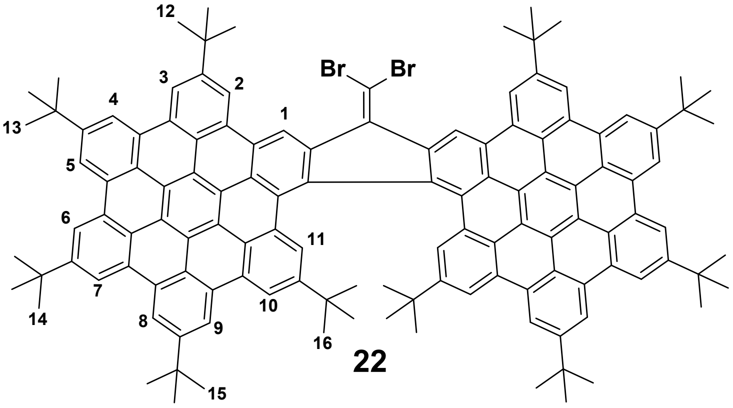

22

A Schlenk-tube equipped with a magnetic stirring bar and a reflux condenser was flame dried under vacuum and subsequently charged with **16** (48.9 mg, 30.0 µmol) and toluene (3 mL). The solution was degassed via N_2 bubbling for 5 min. 2 eq. CBr_4 (19.9 mg, 60.0 µmol) and 4 eq. PPh_3 (31.5 mg, 0.12 mmol) were added. The reaction mixture was stirred for 24 h under reflux (heat-on temperature set to 140 °C). All solvents were evaporated and the crude product was purified by column chromatography over silica gel with hexanes/CH_2Cl_2 1/1. The product was dried under vacuum and obtained as a red, brownish solid in a yield of 58 % (31.0 mg, 17.4 µmol). REMARK: 29 % (14.0 mg, 8.59 µmol) of precursor were recovered after column chromatography.

^{1}H NMR ($CDCl_3$, 400 MHz, rt.): δ [ppm] = 11.00 (s, 2H, 1), 9.53 (s, 2H, 2), 9.43 (s, 2H, 3/4/5/6/7), 9.41-9.38 (m, 8H, 3/4/5/6/7), 9.31 (s, 2H, 8), 8.97 (d, ^4J = 1.0 Hz, 2H, 9), 8.61 (d, ^4J = 1.7 Hz, 2H, 10), 8.45 (d, ^4J = 1.6 Hz, 2H, 11), 1.93 (s, 18H, 12), 1.88 (s, 36H, 13/14), 1.81 (s, 18H, 15), 0.04 (s, 18H, 16).

^{13}C NMR ($CDCl_3$, 100 MHz, rt.): δ [ppm] = 149.5, 149.4, 149.3, 147.0, 139.6, 139.1, 137.5, 130.8, 130.73, 130.66, 130.6, 130.5, 130.3, 130.0, 129.7, 129.4, 127.9, 127.7, 124.2, 124.1, 123.9, 123.64, 123.58, 121.7, 121.5, 121.1, 120.9, 120.8, 120.28, 120.27, 119.5, 119.3, 119.14, 119.09, 119.0, 118.8, 118.4, 35.9, 35.82, 35.80, 35.7, 34.0, 32.10, 32.07, 32.0, 29.9.

HRMS (MALDI-ToF, dctb): m/z (calculated for $C_{126}H_{112}Br_2$) = 1782.7125 $[M]^+$

m/z (found) = 1782.7164 $[M]^+$

error [ppm] = 2.19

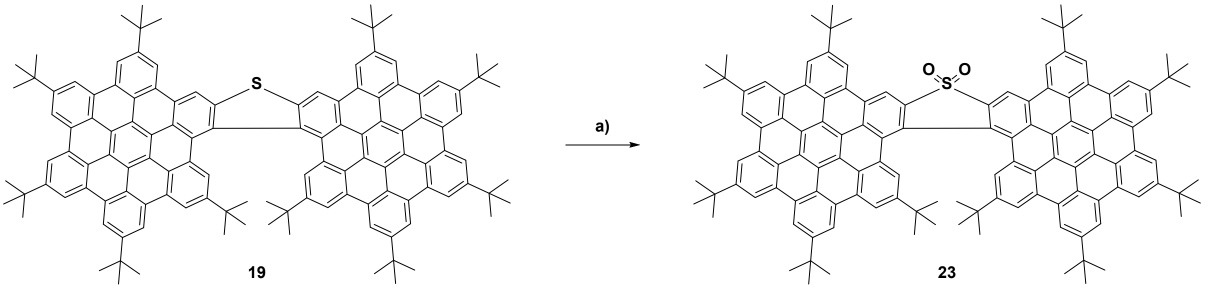

Scheme S5. Oxidation of **19** to sulfone-superhelicene **23**.

Sulfone-superhelicene 23

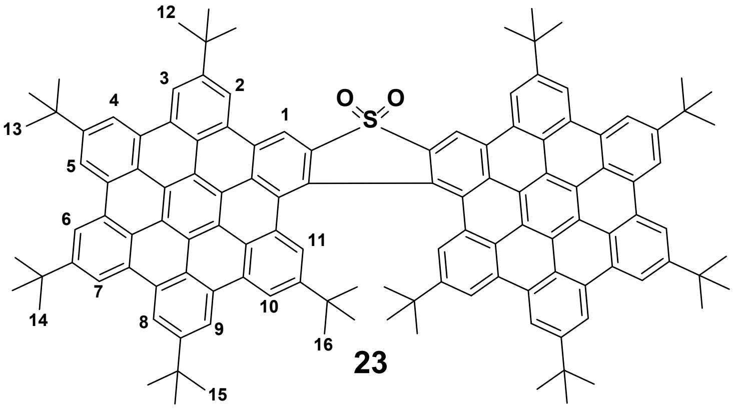

A reaction-tube (10 mL) equipped with a magnetic stirring bar was charged with **19** (30.0 mg, 18.4 µmol) and CH_2Cl_2 (4 mL). The solution was cooled to 0 °C and 5 eq. *m*-CPBA (15.9 mg, 92.0 µmol) were added. The reaction mixture was stirred for 2 h at 0 °C and for additional 66 h at rt. The reaction mixture was poured onto aq. sat. $NaHCO_3$ (50 mL). CH_2Cl_2 (50 mL) were added and the phases were separated. The organic phase was washed with aq. sat. $NaHCO_3$ (50 mL) and brine (50 mL) and was dried over Na_2SO_4. The crude product was purified by column chromatography over silica gel with hexanes/CH_2Cl_2 1/1. The product was dried under vacuum and obtained as an orange solid in a yield of 52 % (16.0 mg, 9.60 µmol).

^{1}H NMR ($CDCl_3$, 400 MHz, rt.): δ [ppm] = 9.89 (s, 2H, 1), 9.52 (s, 2H, 2/3), 9.50 (s, 2H, 2/3), 9.41-9.40 (m, 6H, 4/5/6/7), 9.38 (s, 2H, 4/5/6/7), 9.33 (s, 2H, 8), 8.90 (d, ^4J = 0.7 Hz, 2H, 9), 8.53 (d, ^4J = 1.6 Hz, 2H, 10), 8.43 (d, ^4J = 1.4 Hz, 2H, 11), 1.94 (s, 18H, 12), 1.88 (s, 36H, 13/14), 1.79 (s, 18H, 15), 0.01 (s, 18H, 16).

^{13}C NMR ($CDCl_3$, 100 MHz, rt.): δ [ppm] = 150.2, 149.9, 149.7, 149.6, 147.2, 138.1, 132.1, 130.87, 130.85, 130.64, 130.60, 130.55, 130.38, 130.36, 130.3, 130.2, 129.4, 129.18, 129.16, 128.3, 124.0, 123.9, 123.82, 123.78, 123.4, 122.2, 121.84, 121.81, 121.6, 121.19, 121.17, 120.6, 120.5, 120.1, 119.44, 119.37, 119.2, 119.1, 118.9, 118.7, 114.0, 36.0, 35.9, 35.8, 35.7, 34.0, 32.1, 32.03, 32.00, 29.9.

MS (MALDI-ToF, dctb): m/z = 1665 $[M]^+$

HRMS (MALDI-ToF, dctb): m/z (calculated for $C_{124}H_{112}SO_2$) = 1664.8378 $[M]^+$

m/z (found) = 1664.8416 $[M]^+$

error [ppm] = 2.30

3 Physicochemical data

Table S 1. Wavelength of the absorptions in toluene.

Absorption	β-bands [nm]	p-bands [nm]	α-bands [nm]	CT-bands [nm]
**19**	359, 381	399.5, 410, 437	457.5, 489.5, 523.5	-
**23**	347.5, 364.5, 378.5	390, 416.5	467, 499.5, 530.5	-
**17**	355.5, 377	399, 426.5	449.5, 480, 517.5	-
**16**	347.5, 370	386, 406	444, 473, 502	583
**21**	330, 354, 370.5	387.5, 437.5	483.5, 508.5	572.5, 609, 743

Table S 2. Extinction coefficients of the most intense absorption band of each compound in different solvents.

Extinction coefficients	toluene ε [10^5 $M^{-1}cm^{-1}$] (λ [nm])	THF ε [10^5 $M^{-1}cm^{-1}$] (λ [nm])	PhCN ε [10^5 $M^{-1}cm^{-1}$] (λ [nm])
**19**	1.40 (381)	1.51 (377.5)	1.41 (381)
**23**	1.06 (364.5)	1.27 (363)	1.01 (365.5)
**17**	1.37 (377)	1.25 (373.5)	1.05 (377)
**16**	1.18 (370)	1.44 (364)	1.13 (367)

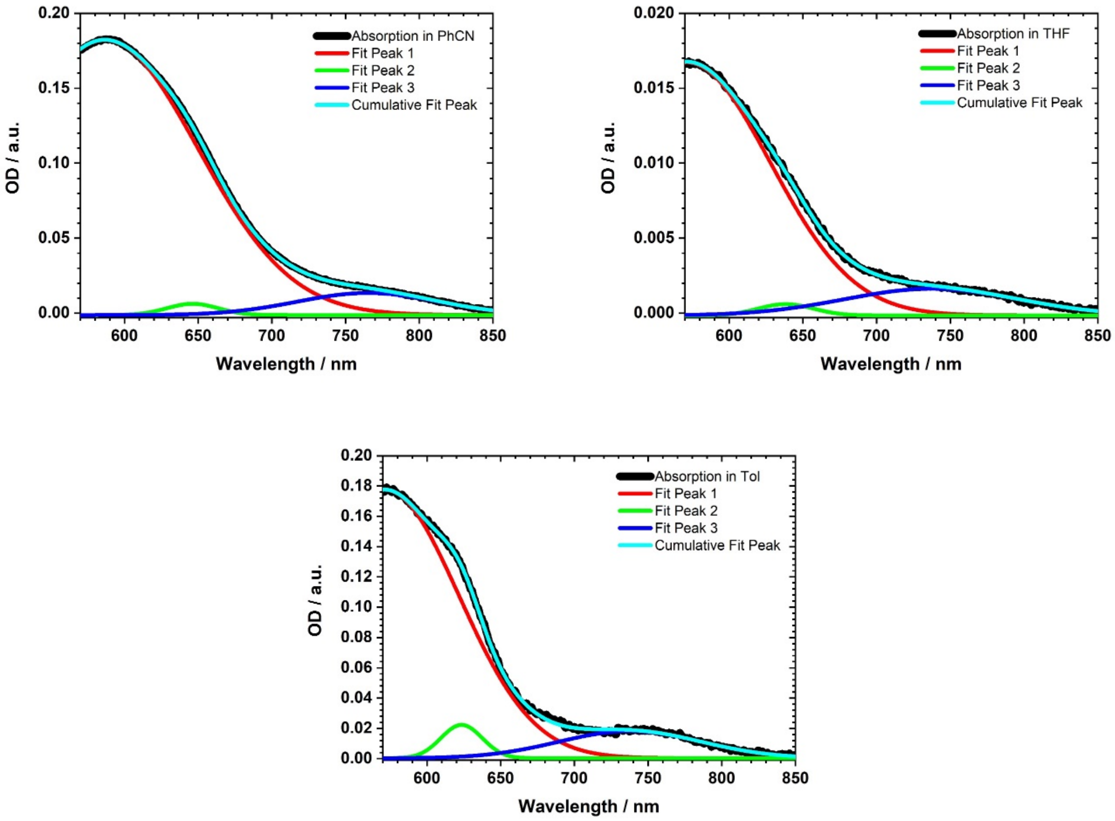

Figure S 1. Multipeak analysis of the absorption of **21** between 570 nm and 850 nm in PhCN (top left) and THF (top right) and toluene (bottom).

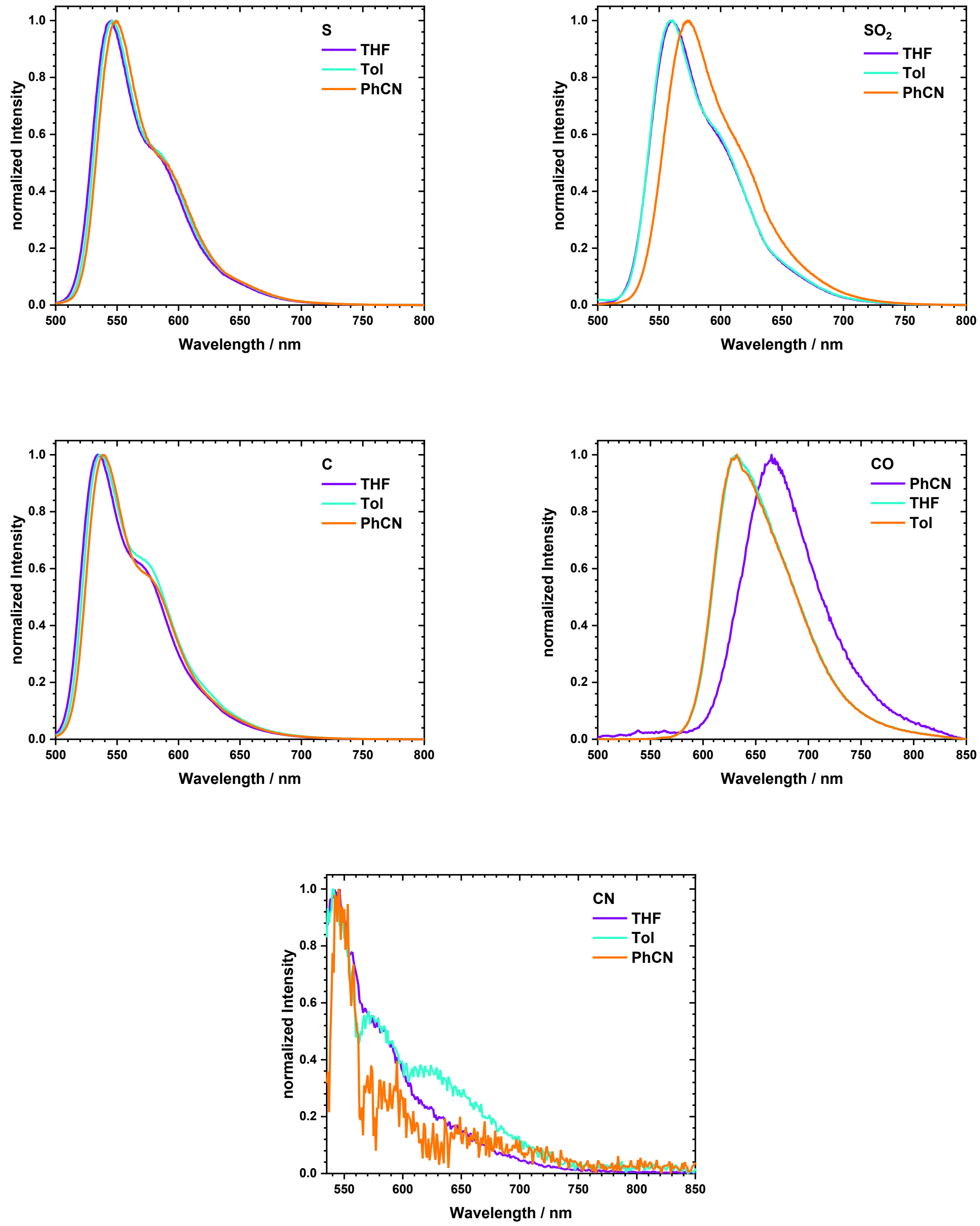

Figure S 2. Fluorescence spectra of **19** (top left), **23** (top right), **17** (middle left), **16** (middle right) and **21** (bottom) in toluene, THF and PhCN.

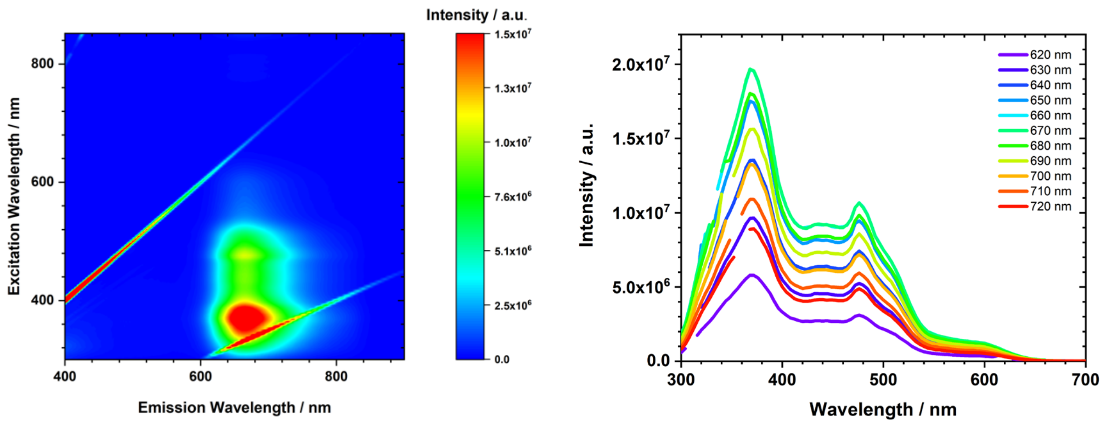

Figure S 3. 3D fluorescence map and excitation spectra of **16** in PhCN at various emission wavelengths showing no excitation dependence of the emission. The changes, occurring at lower and higher emission wavelength at low excitation wavelengths stem from PhCN related emission.

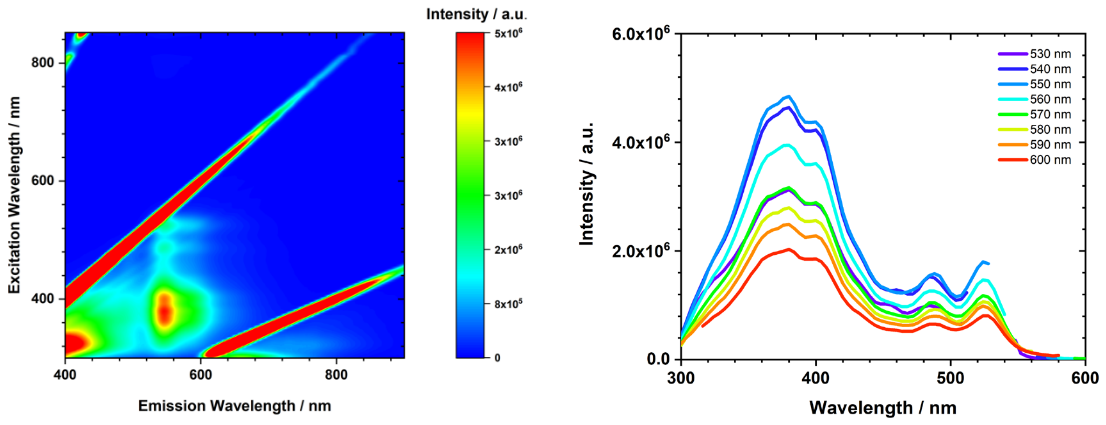

Figure S 4. 3D fluorescence map and excitation spectra of **21** in PhCN at various emission wavelengths showing no excitation dependence of the emission. The changes, occurring at lower and higher emission wavelengths at low excitation wavelength stem from PhCN related emission.

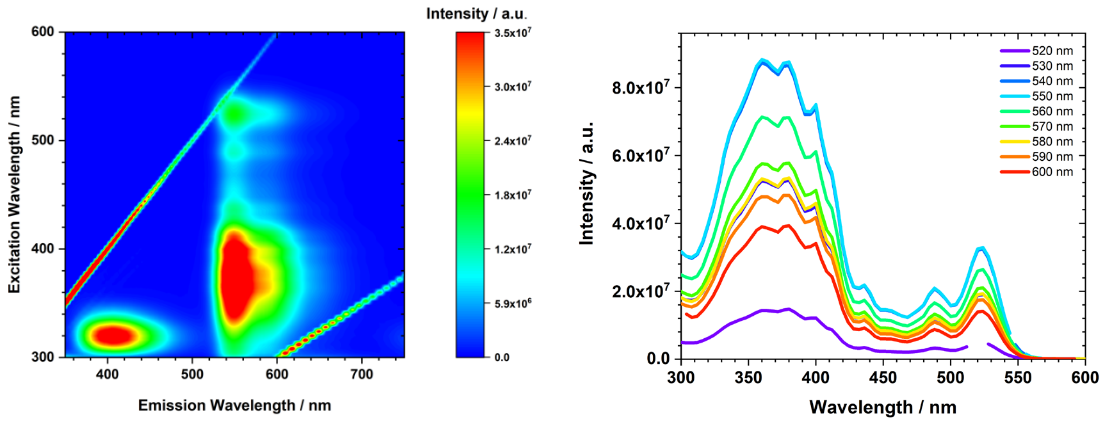

Figure S 5. 3D fluorescence map (left) and excitation spectra of **19** in PhCN at various emission wavelengths showing no excitation dependence of the emission. The changes, occurring at lower and higher emission wavelengths at low excitation wavelength stem from PhCN related emission.

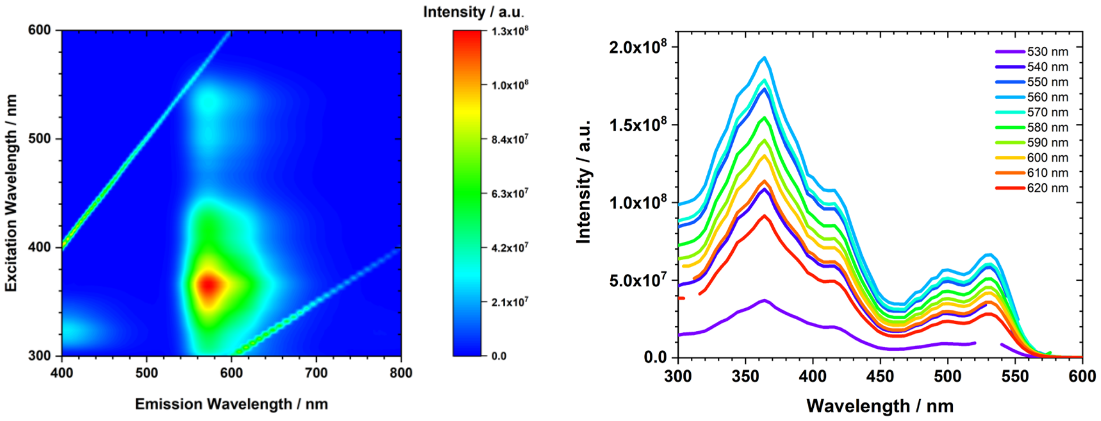

Figure S 6. 3D fluorescence map (left) and excitation spectra of **23** in PhCN at various emission wavelengths showing no excitation dependence of the emission. The changes, occurring at lower and higher emission wavelengths at low excitation wavelength stem from PhCN related emission.

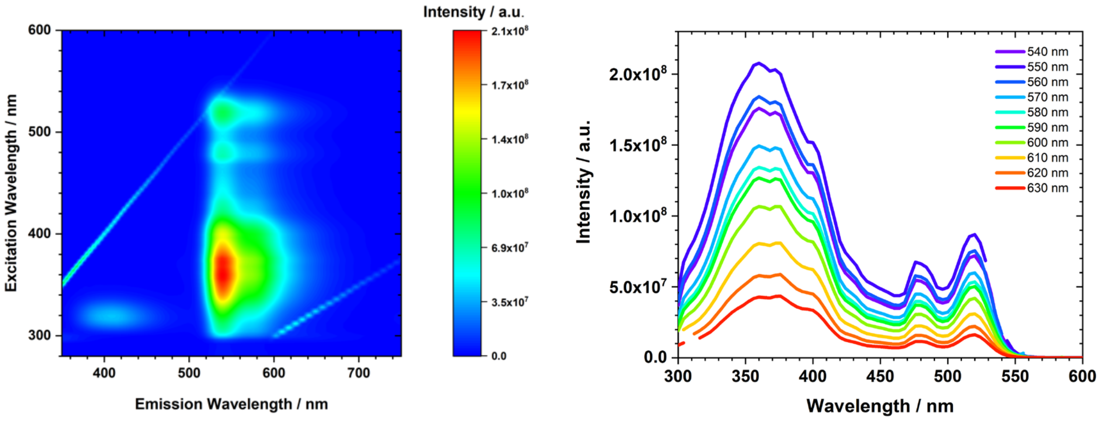

Figure S 7. 3D fluorescence map (left) and excitation spectra of **17** in PhCN at various emission wavelengths showing no excitation dependence of the emission. The changes, occurring at lower and higher emission wavelengths at low excitation wavelength stem from PhCN related emission.

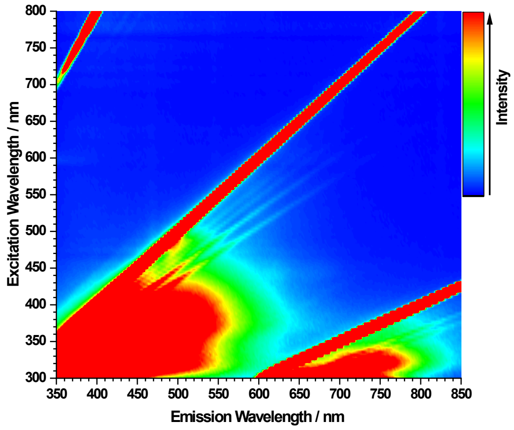

Figure S 8. 3D emission map of PhCN.

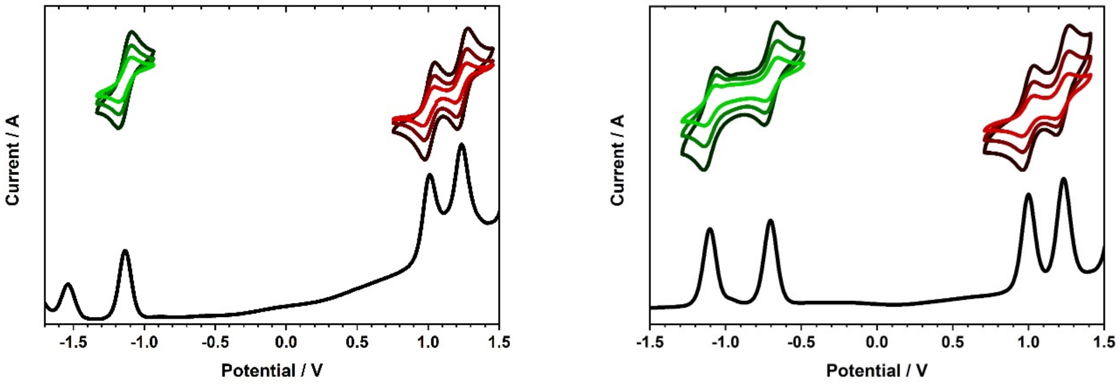

Figure S 9. Left: DPV and CV spectra of 16 in CH_2Cl_2. Right: DPV and CV spectra of 21 in CH_2Cl_2.

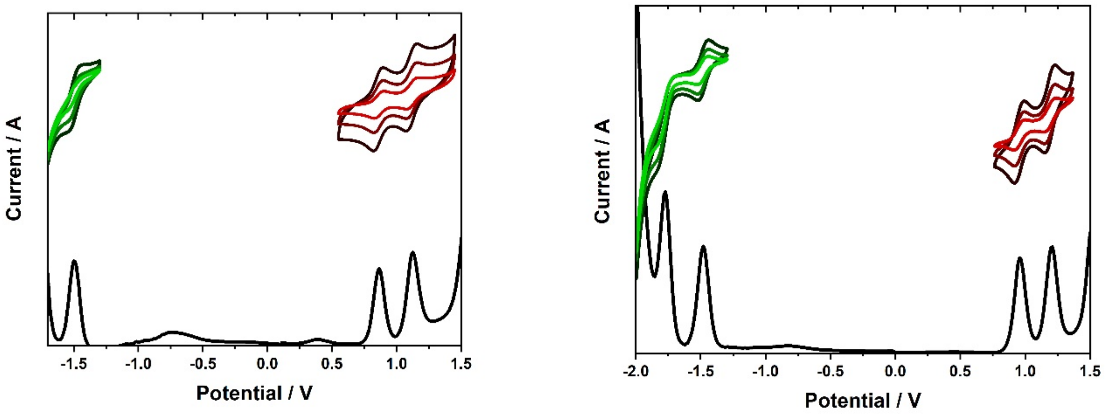

Figure S 10. Left: DPV and CV spectra of 17 in CH_2Cl_2. Right: DPV and CV spectra of 19 in CH_2Cl_2.

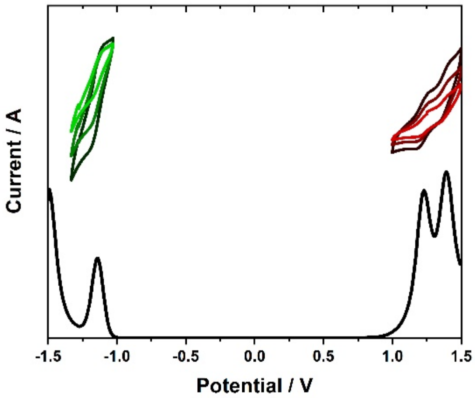

Figure S 11. DPV and CV spectra of 23 in CH_2Cl_2

Table S 3. Excited state lifetimes of all compounds in toluene, THF and PhCN obtained by global analysis of transient absorption spectra in femtosecond (Helios) and nanosecond (EOS) scale. The excitation wavelength was 550 nm.

**19**	**τ_1 [ps]**	**τ_2 [ps]**	**τ_3 [ns]**	**τ_4 [µs]**
	**Helios**		**EOS**	
**Toluene**	1470	-	1.22	11.4
**THF**	1190	-	2.43	21.3
**PhCN**	1200	-	1.94	38.5
**23**	**τ_1 [ps]**	**τ_2 [ps]**	**τ_3 [ns]**	**τ_4 [µs]**
	**Helios**		**EOS**	
**Toluene**	2030	-	2.64	24.4
**THF**	3270	-	3.13	12.4
**PhCN**	2240	-	2.84	51.2
**17**	**τ_1 [ps]**	**τ_2 [ps]**	**τ_3 [ns]**	**τ_4 [µs]**
	**Helios**		**EOS**	
**Toluene**	3440	-	3.29	17.8
**THF**	3330	-	2.85	51.6
**PhCN**	3510	-	3.75	91.1
**16**	**τ_1 [ps]**	**τ_2 [ps]**	**τ_3 [ns]**	**τ_4 [µs]**
	**Helios**		**EOS**	
**Toluene**	1.04	-	5.11	21.03
**THF**	1.53	-	5.10	27.25
**PhCN**	18.49	-	2.42	53.31
**21**	**τ_1 [ps]**	**τ_2 [ps]**	**τ_3 [ns]**	**τ_4 [µs]**
	**Helios**		**EOS**	
**Toluene**	-	52.99	-	-
**THF**	-	37.66	-	-
**PhCN**	-	31.63	-	-

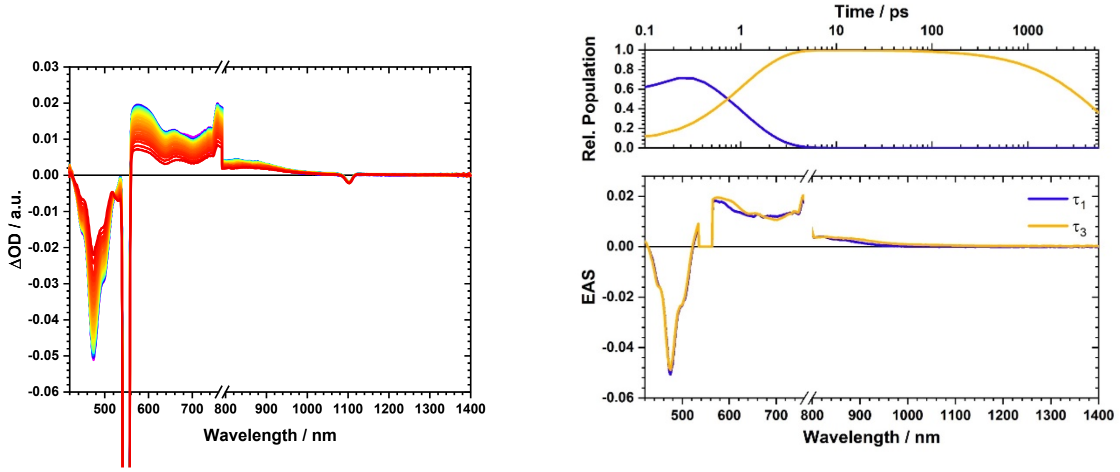

Figure S 12. Femtosecond transient absorption spectra of **16** in toluene (left) with time delays from 0 to 5500 ps and corresponding evolution associated spectra (right) obtained by global analysis upon excitation at 550 nm.

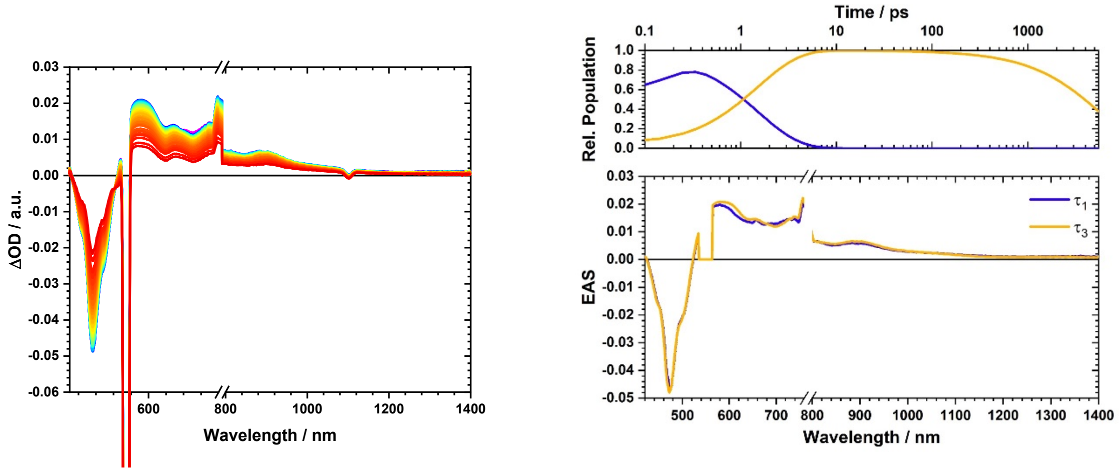

Figure S 13. Femtosecond transient absorption spectra of **16** in to THF (left) with time delays from 0 to 5500 ps and corresponding evolution associated spectra (right) obtained by global analysis upon excitation at 550 nm.

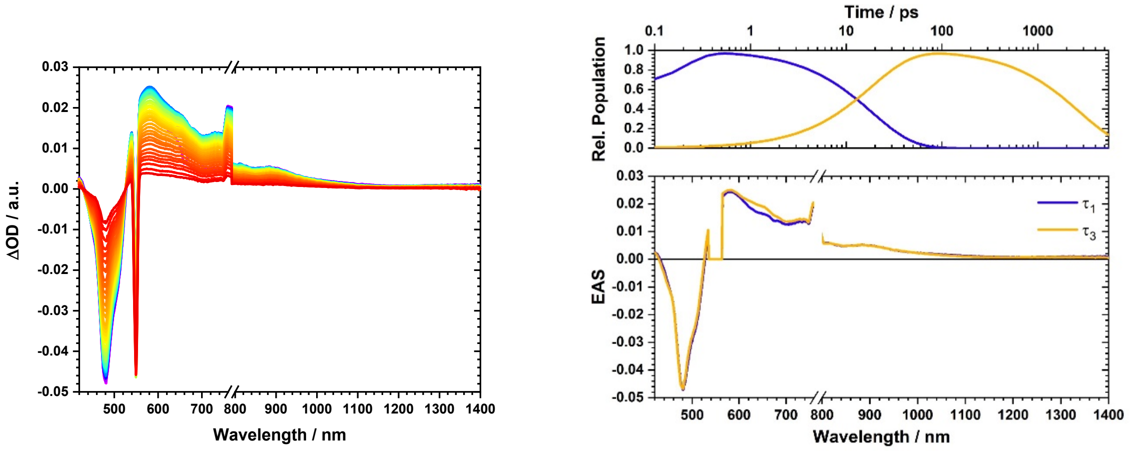

Figure S 14. Femtosecond transient absorption spectra of **16** in to PhCN (left) with time delays from 0 to 5500 ps and corresponding evolution associated spectra (right) obtained by global analysis upon excitation at 550 nm.

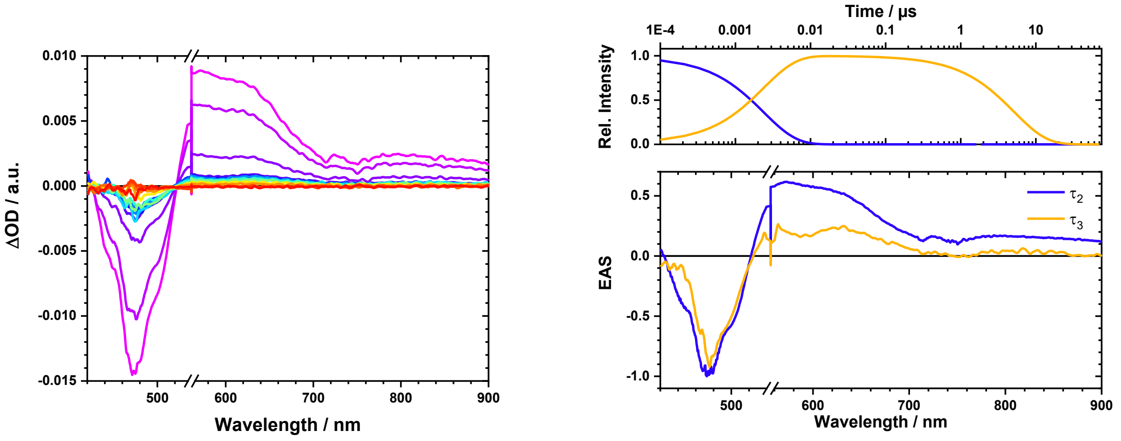

Figure S 15. Nanosecond transient absorption spectra of **16** in toluene (left) with time delays from 0 to 350 ns and corresponding evolution associated spectra (right) obtained by global analysis upon excitation at 550 nm.

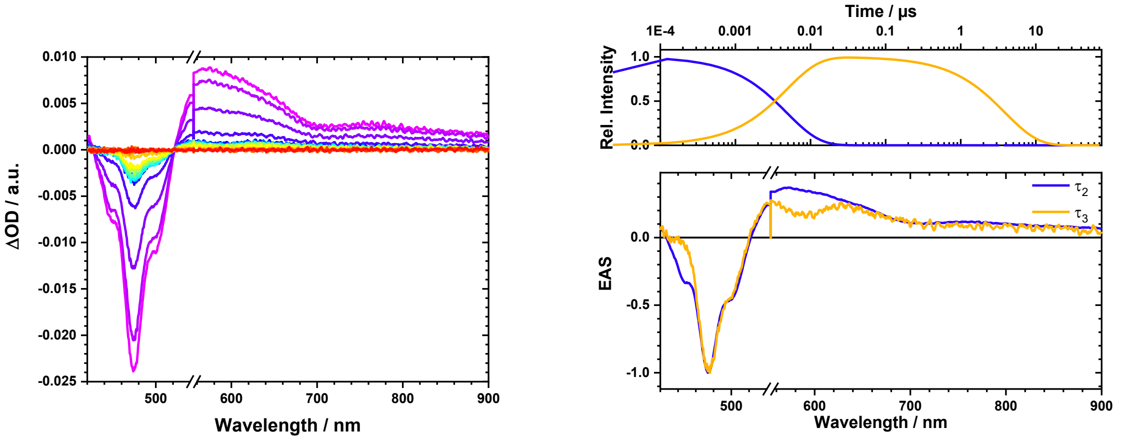

Figure S 16. Nanosecond transient absorption spectra of **16** in THF (left) with time delays from 0 to 350 ns and corresponding evolution associated spectra (right) obtained by global analysis upon excitation at 550 nm.

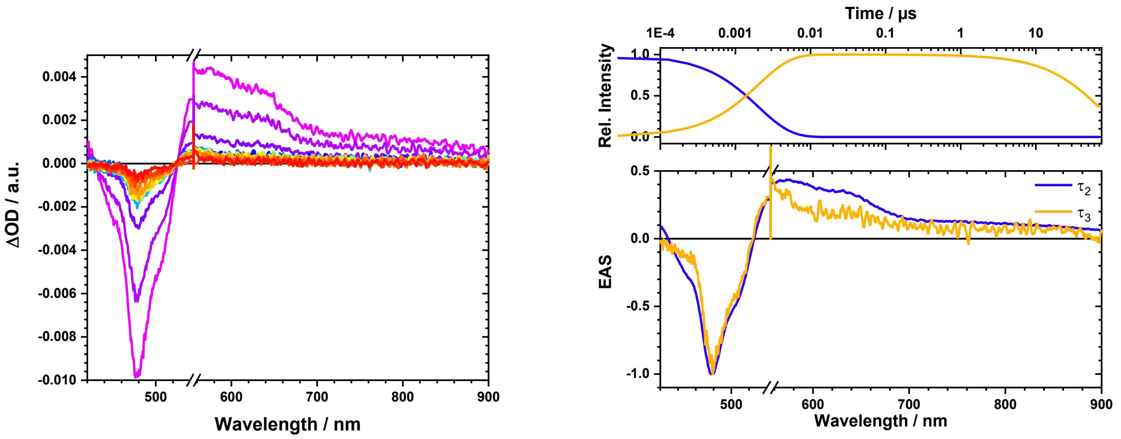

Figure S 17. Nanosecond transient absorption spectra of **16** in PhCN (left) with time delays from 0 to 350 ns and corresponding evolution associated spectra (right) obtained by global analysis upon excitation at 550 nm.

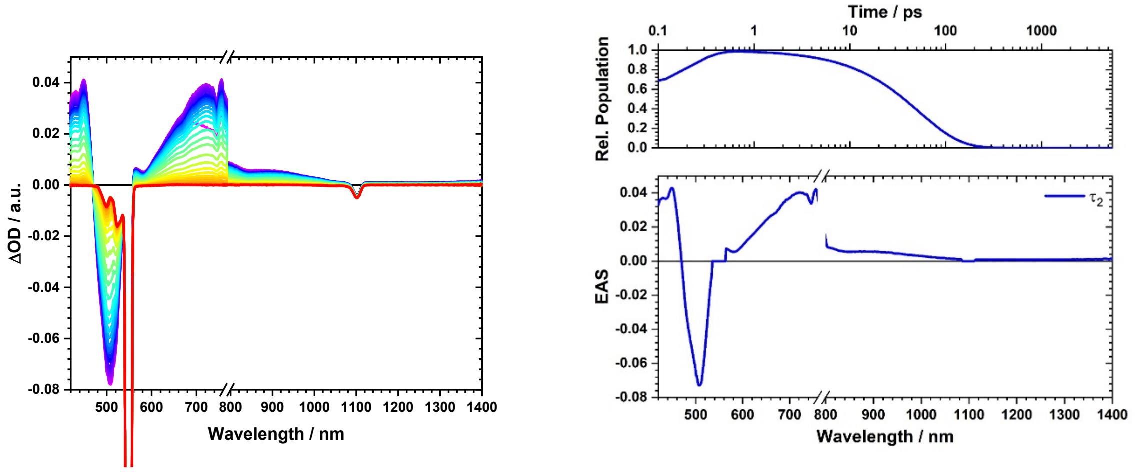

Figure S 18. Femtosecond transient absorption spectra of **21** in toluene (left) with time delays from 0 to 660 ps and corresponding evolution associated spectra (right) obtained by global analysis upon excitation at 550 nm.

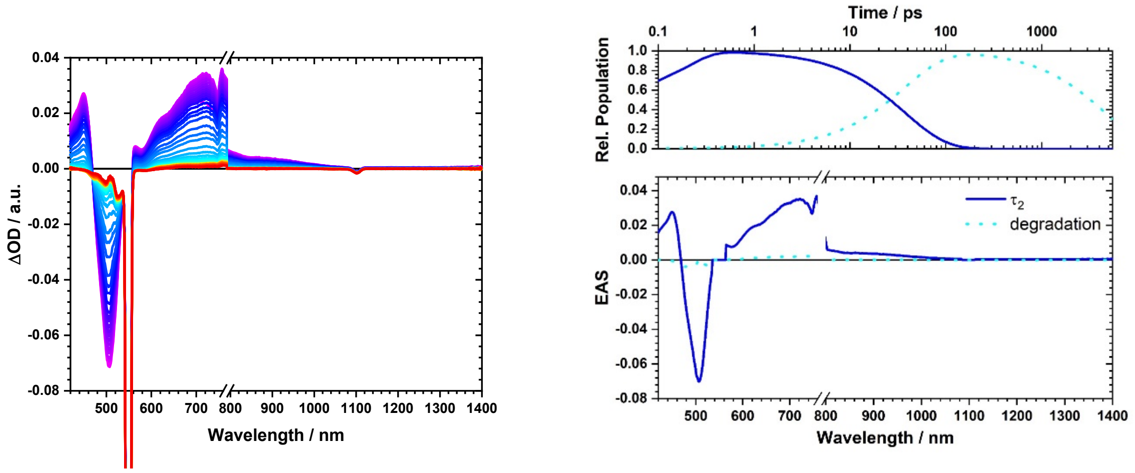

Figure S 19. Femtosecond transient absorption spectra of **21** in THF (left) with time delays from 0 to 5500 ps and corresponding evolution associated spectra (right) obtained by global analysis upon excitation at 550 nm.

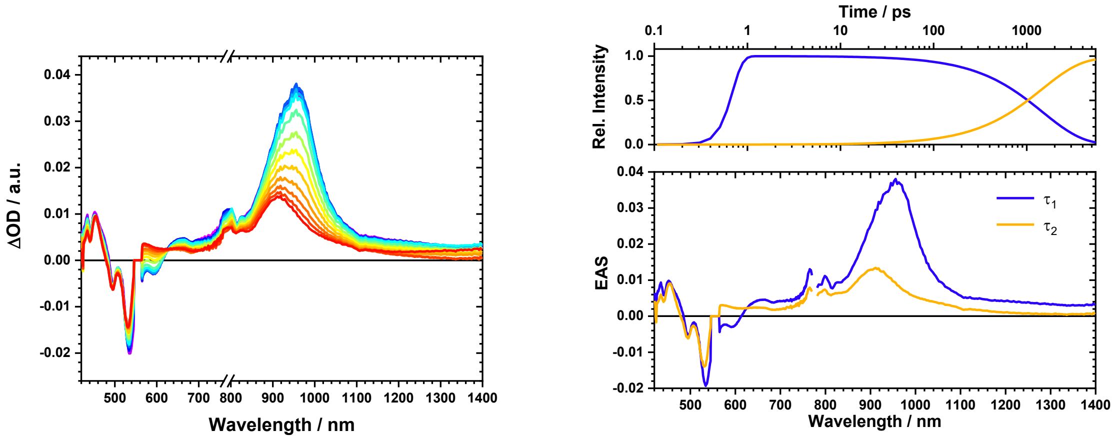

Figure S 20. Femtosecond transient absorption spectra of **19** in toluene (left) with time delays from 0 to 5500 ps and corresponding evolution associated spectra (right) obtained by global analysis upon excitation at 550 nm.

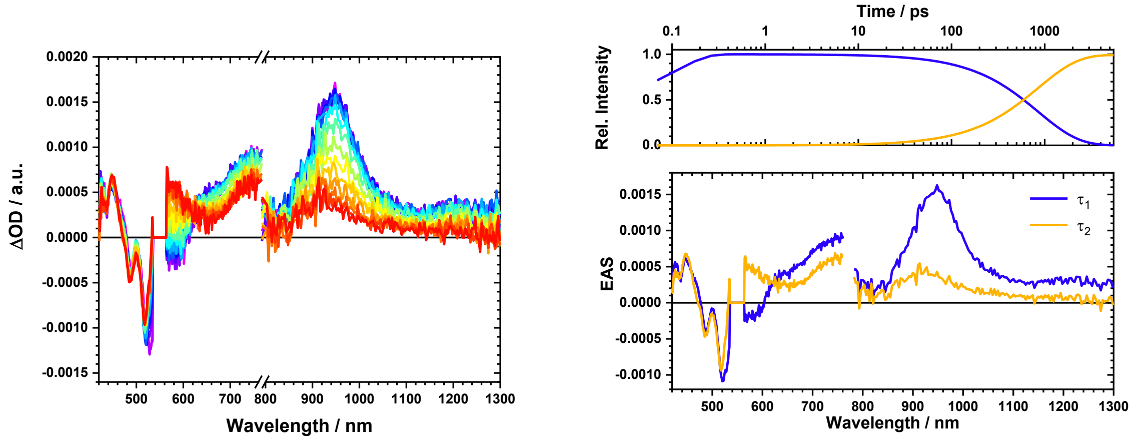

Figure S 21. Femtosecond transient absorption spectra of **19** in THF (left) with time delays from 0 to 5500 ps and corresponding evolution associated spectra (right) obtained by global analysis upon excitation at 550 nm.

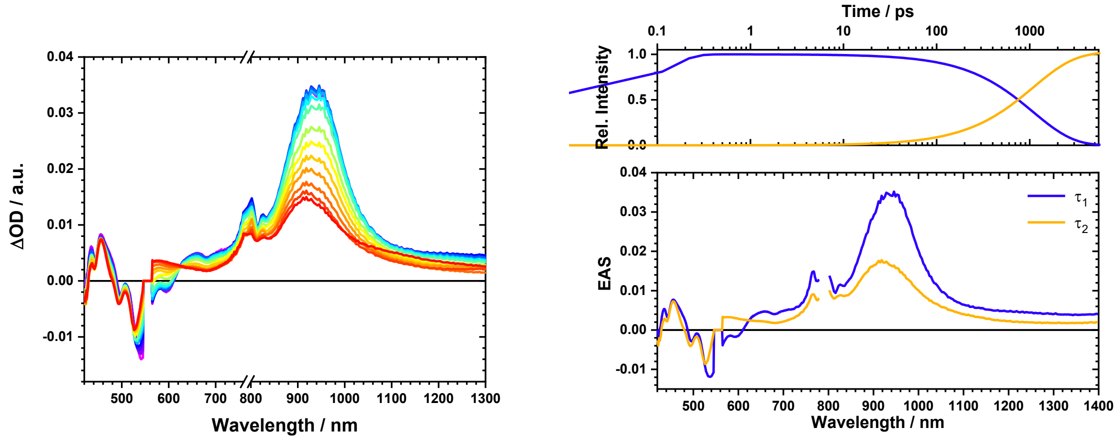

Figure S 22. Femtosecond transient absorption spectra of **19** in PhCN (left) with time delays from 0 to 5500 ps and corresponding evolution associated spectra (right) obtained by global analysis upon excitation at 550 nm.

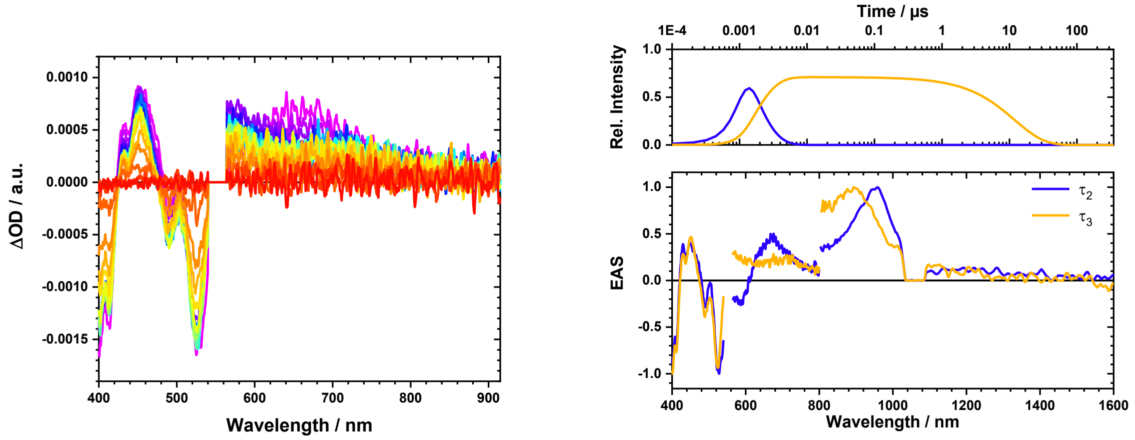

Figure S 23. Nanosecond transient absorption spectra of **19** in toluene (left) with time delays from 0 to 350 μs and corresponding normalized evolution associated spectra (right) obtained by global analysis upon excitation at 550 nm.

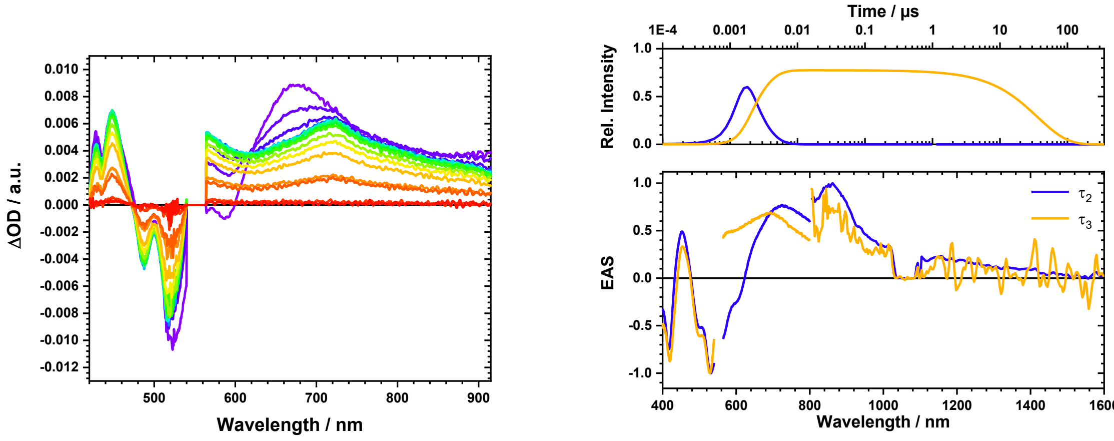

Figure S 24. Nanosecond transient absorption spectra of **19** in THF (left) with time delays from 0 to 350 µs and corresponding normalized evolution associated spectra (right) obtained by global analysis upon excitation at 550 nm.

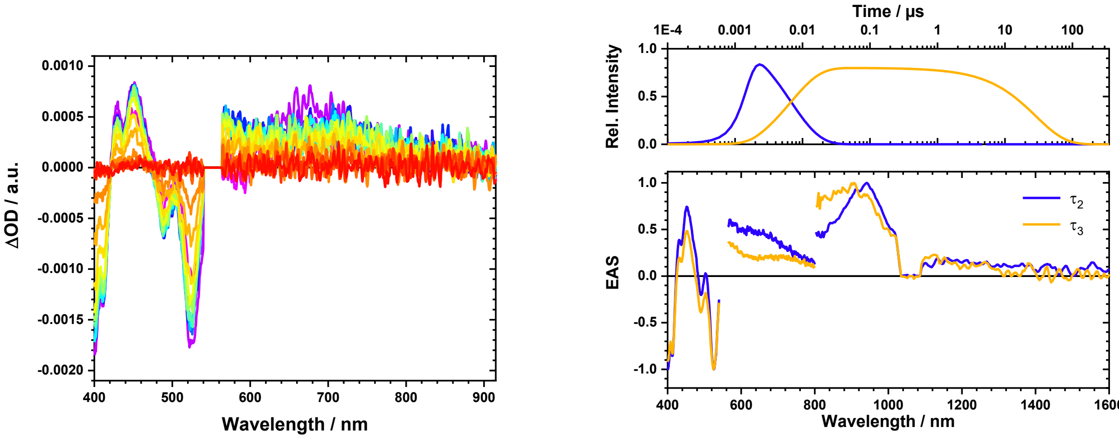

Figure S 25. Nanosecond transient absorption spectra of **19** in PhCN (left) with time delays from 0 to 350 µs and corresponding normalized evolution associated spectra (right) obtained by global analysis upon excitation at 550 nm.

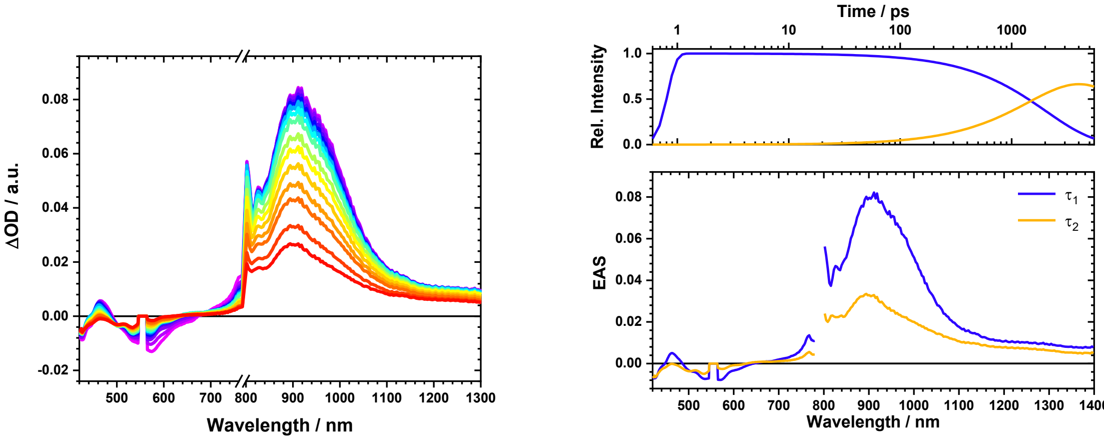

Figure S 26. Femtosecond transient absorption spectra of **23** in toluene (left) with time delays from 0 to 5500 ps and corresponding evolution associated spectra (right) obtained by global analysis upon excitation at 550 nm.

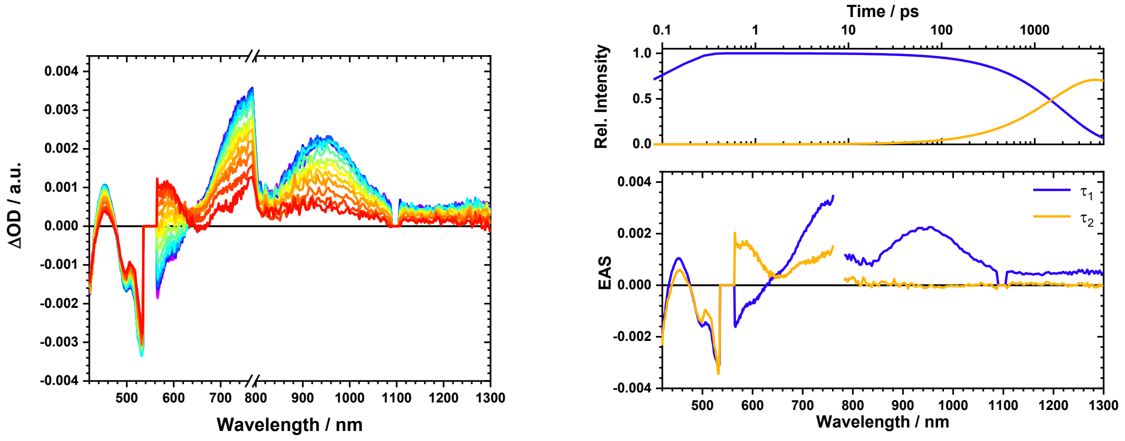

Figure S 27. Femtosecond transient absorption spectra of **23** in THF (left) with time delays from 0 to 5500 ps and corresponding evolution associated spectra (right) obtained by global analysis upon excitation at 550 nm.

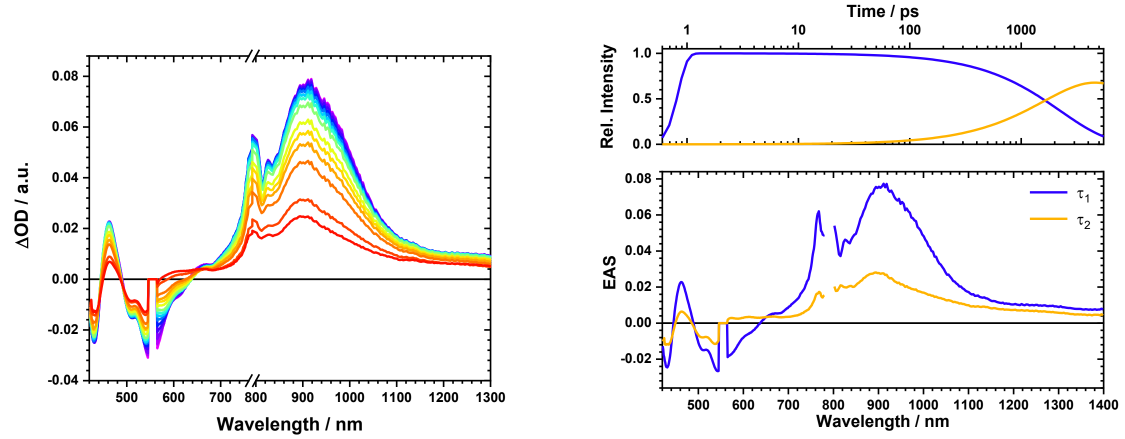

Figure S 28. Femtosecond transient absorption spectra of **23** in PhCN (left) with time delays from 0 to 5500 ps and corresponding evolution associated spectra (right) obtained by global analysis upon excitation at 550 nm.

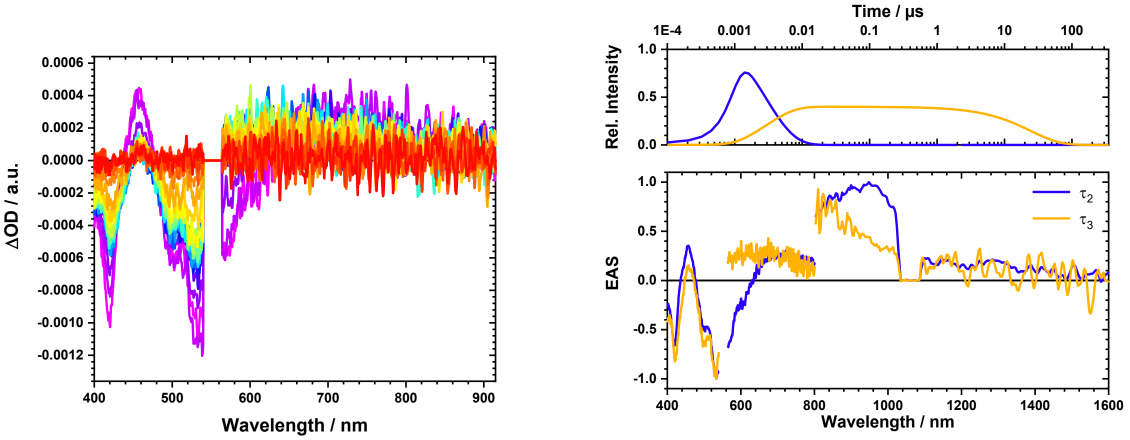

Figure S 29. Nanosecond transient absorption spectra of **23** in toluene (left) with time delays from 0 to 350 μs and corresponding normalized evolution associated spectra (right) obtained by global analysis upon excitation at 550 nm.

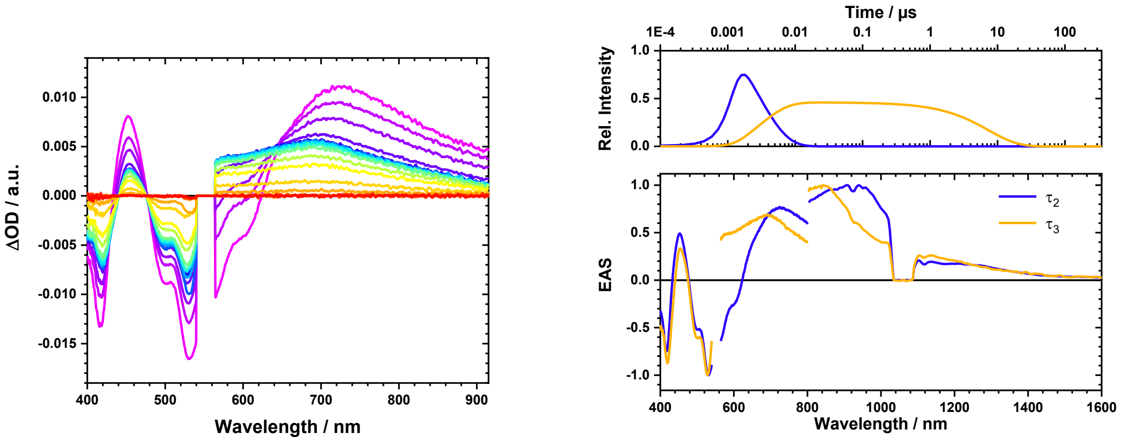

Figure S 30. Nanosecond transient absorption spectra of **23** in THF (left) with time delays from 0 to 350 µs and corresponding normalized evolution associated spectra (right) obtained by global analysis upon excitation at 550 nm.

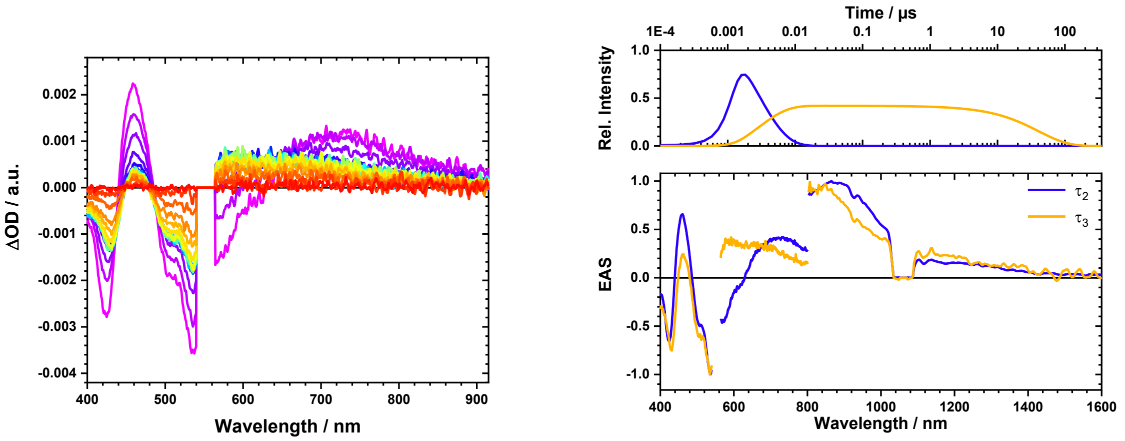

Figure S 31. Nanosecond transient absorption spectra of **23** in PhCN (left) with time delays from 0 to 350 µs and corresponding normalized evolution associated spectra (right) obtained by global analysis upon excitation at 550 nm.

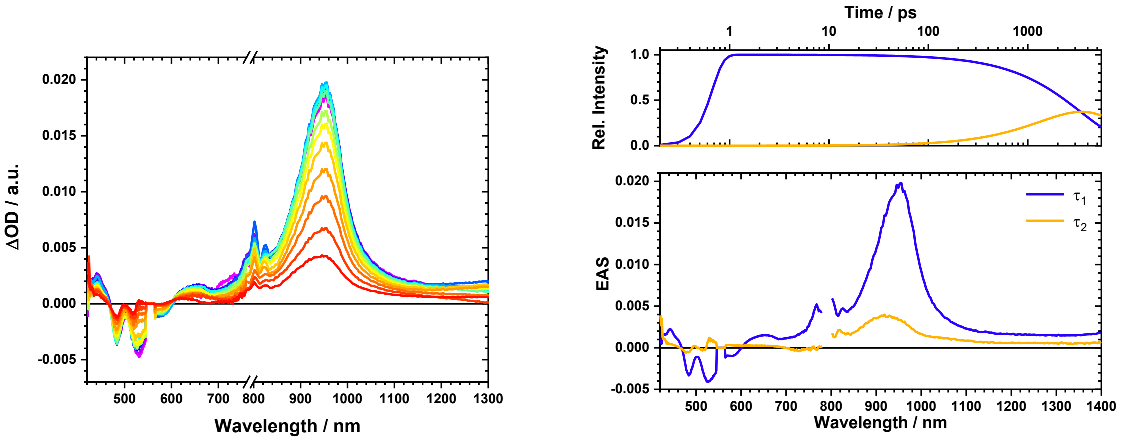

Figure S 32. Femtosecond transient absorption spectra of **17** in toluene (left) with time delays from 0 to 5500 ps and corresponding evolution associated spectra (right) obtained by global analysis upon excitation at 550 nm.

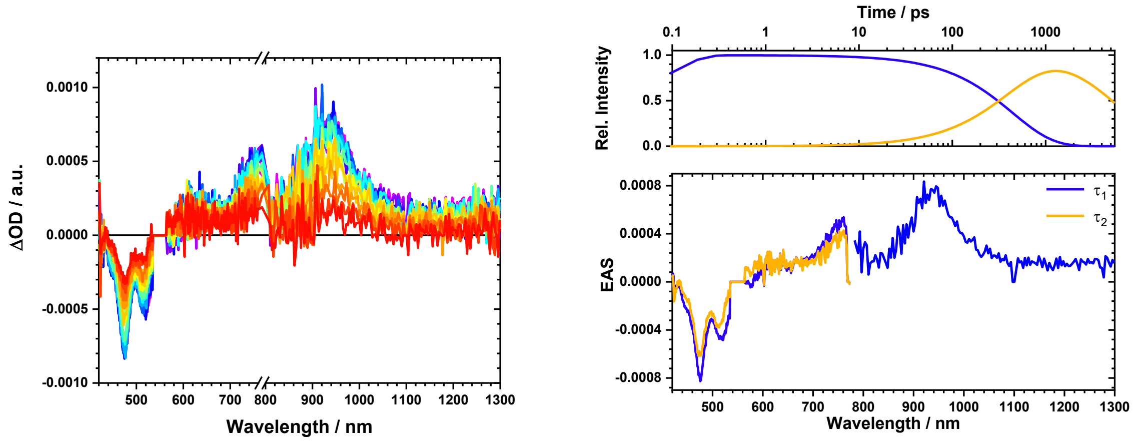

Figure S 33. Femtosecond transient absorption spectra of **17** in THF (left) with time delays from 0 to 5500 ps and corresponding evolution associated spectra (right) obtained by global analysis upon excitation at 550 nm.

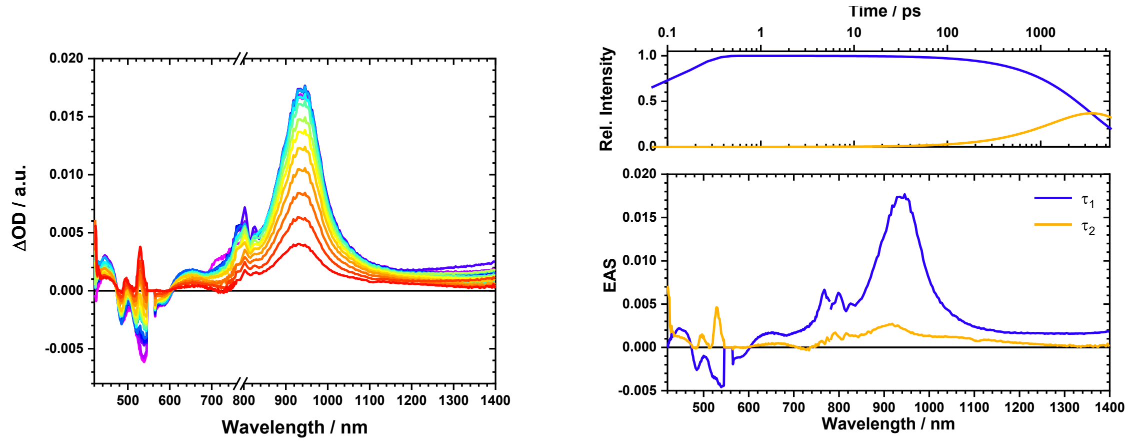

Figure S 34. Femtosecond transient absorption spectra of **17** in PhCN (left) with time delays from 0 to 5500 ps and corresponding evolution associated spectra (right) obtained by global analysis upon excitation at 550 nm.

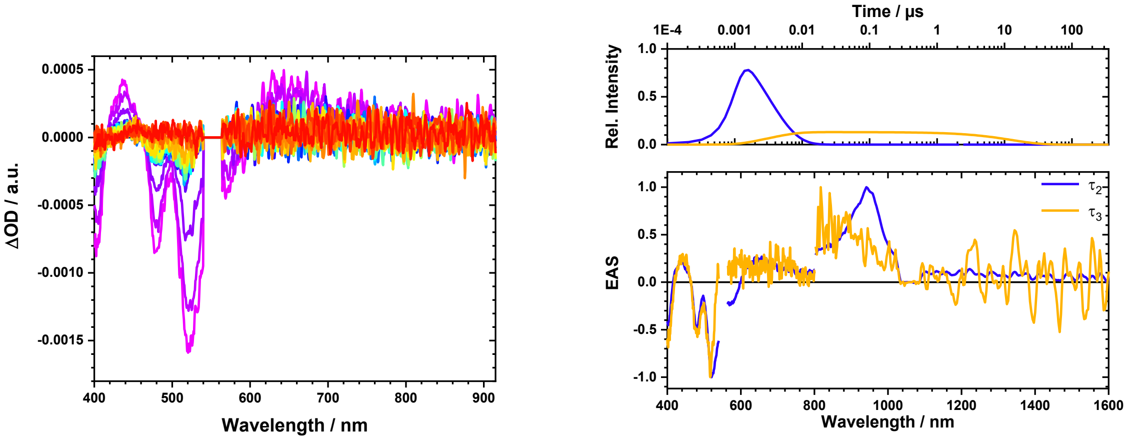

Figure S 35. Nanosecond transient absorption spectra of **17** in toluene (left) with time delays from 0 to 350 µs and corresponding normalized evolution associated spectra (right) obtained by global analysis upon excitation at 550 nm.

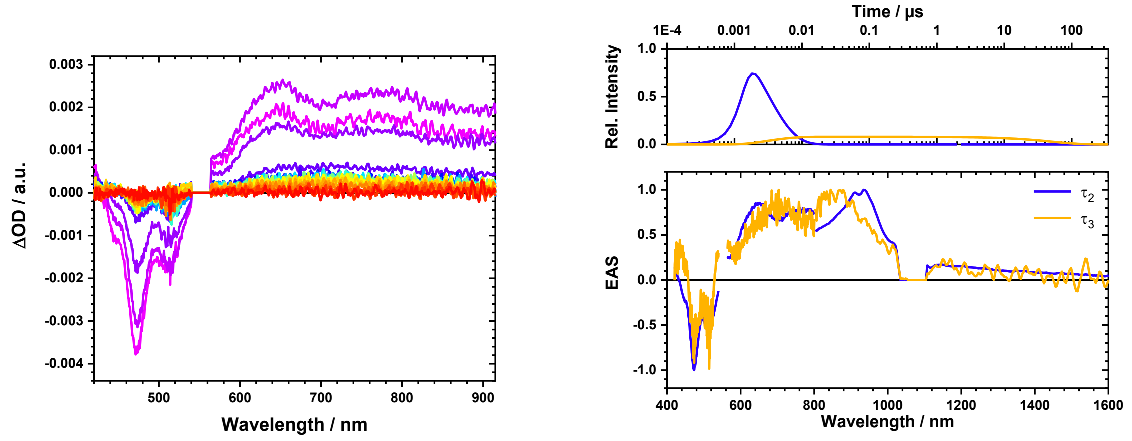

Figure S 36. Nanosecond transient absorption spectra of **17** in THF (left) with time delays from 0 to 350 μs and corresponding normalized evolution associated spectra (right) obtained by global analysis upon excitation at 550 nm.

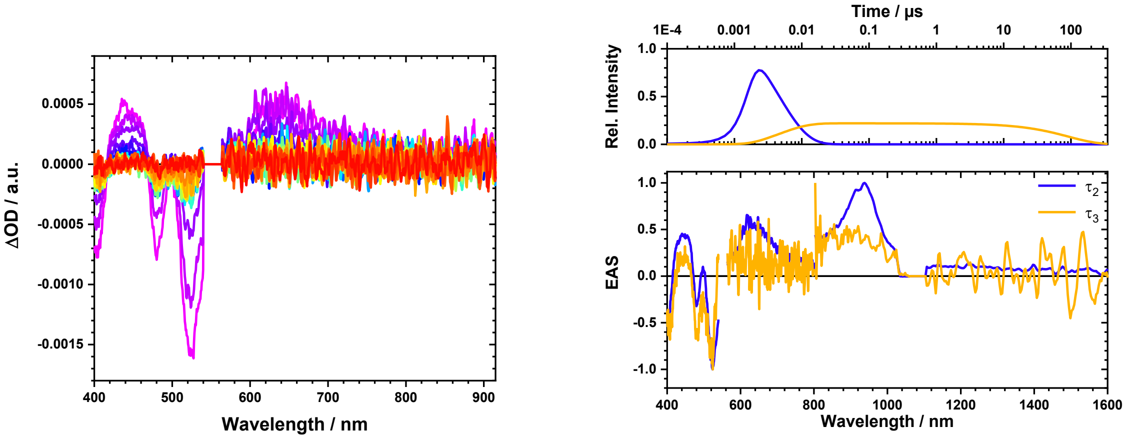

Figure S 37. Nanosecond transient absorption spectra of **17** in PhCN (left) with time delays from 0 to 350 μs and corresponding normalized evolution associated spectra (right) obtained by global analysis upon excitation at 550 nm.

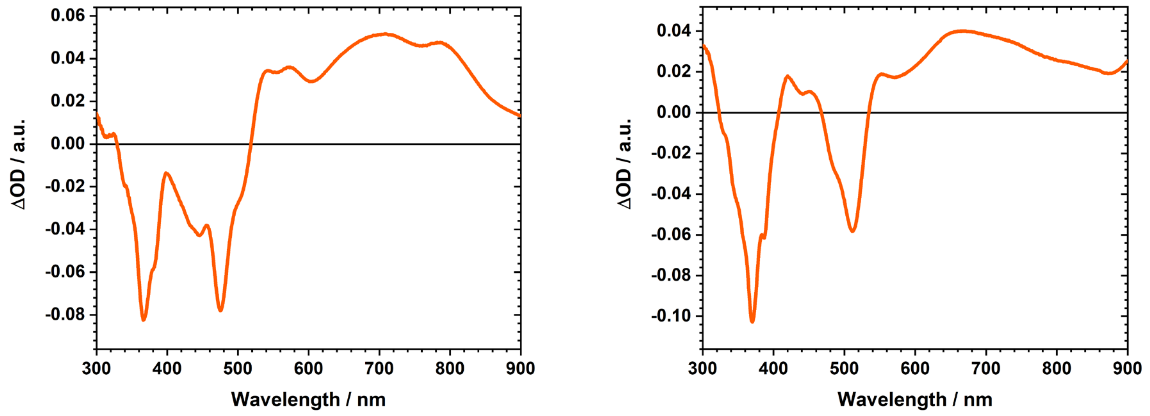

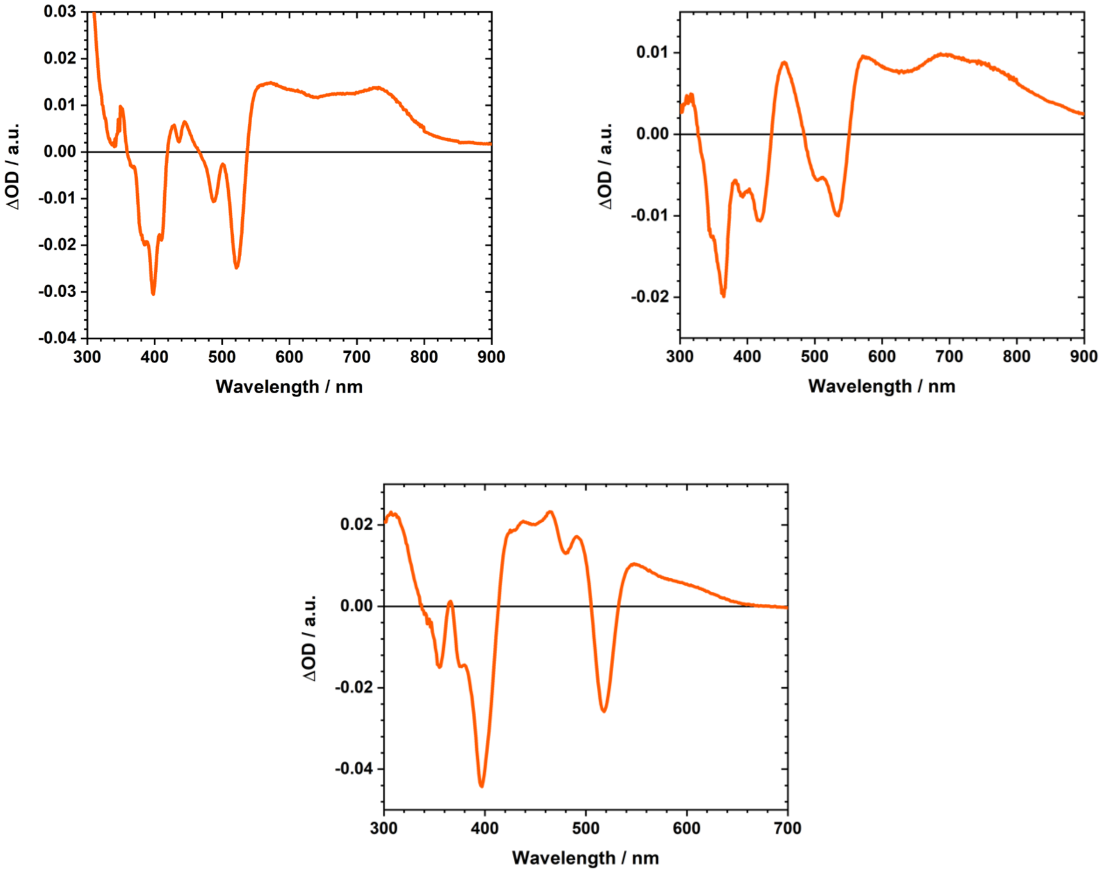

Figure S 38. Differential absorption spectra of **16** (upper left), **21** (upper right), **19** (middle left), **23** (middle right) and **17** (bottom) in argon saturated CH_2Cl_2 containing 0.2 M $TBAPF_6$ obtained by electrochemical oxidation with applied potential of 1.0 V *versus* Ag wire.

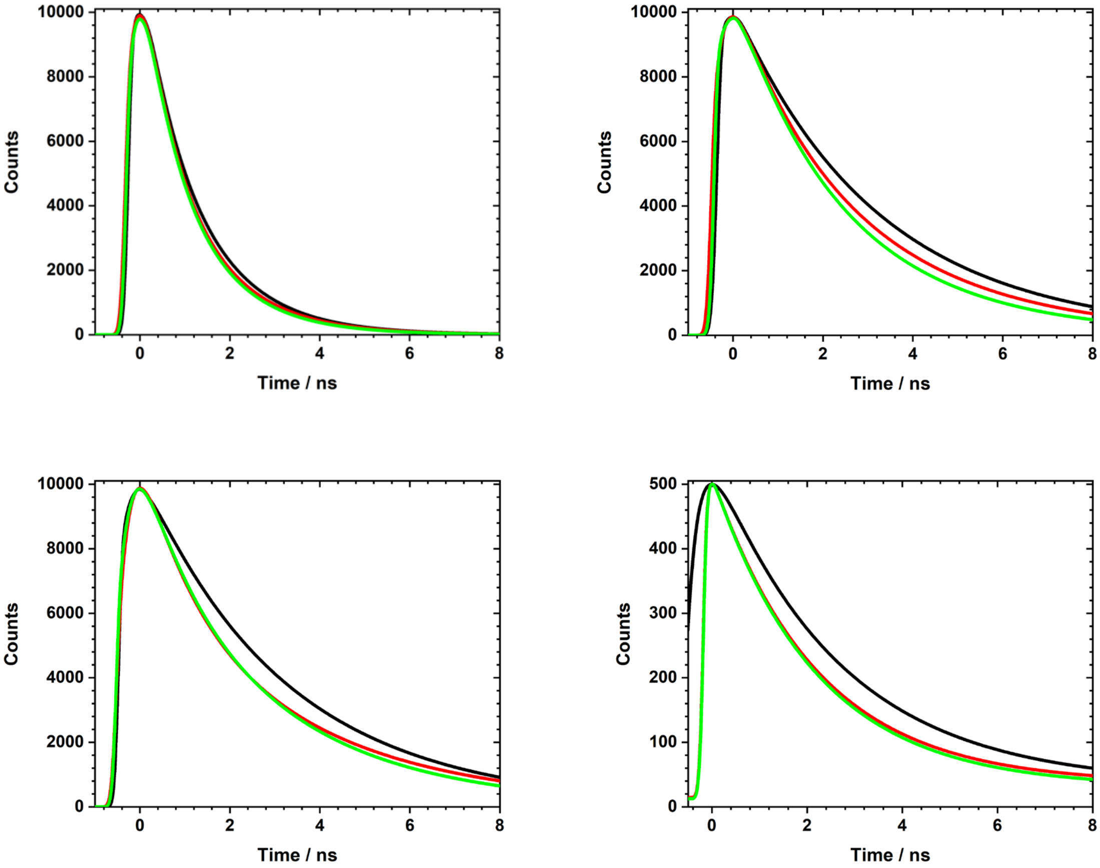

Figure S 39. Photoluminescence decay curves of **19** (upper left), **23** (upper right), **17** (bottom left) and **16** (bottom right) in argon saturated PhCl (black), THF (red) and toluene (green). Excitation wavelength was set to 480 nm and emission monochromator to the corresponding emission maximum.

4 X-Ray data

4.1 Data for fluorenone-superhelicene 16

Single crystals of **16** ($C_{125}H_{112}O$) were crystallized by solvent diffusion from CH_2Cl_2 with MeOH. A suitable crystal was selected and mounted on a loop on a SuperNova, Dual, Cu at zero, Atlas diffractometer. The crystal was kept at 153.00(10) K during data collection. Using Olex2[S9], the structure was solved with the ShelXS[S10] structure solution program using Direct Methods and refined with the ShelXL[S11] refinement package using Least Squares minimization.

Table S 4. Crystal data and structure refinement for **16**.

Identification code	17Jux_DR01
Empirical formula	$C_{125}H_{112}O$
Formula weight	1630.14
Temperature/K	153.00(10)
Crystal system	triclinic
Space group	P-1
a/Å	17.0104(11)
b/Å	17.0342(10)
c/Å	19.9546(13)
α/°	84.267(5)
β/°	66.094(6)
γ/°	73.888(5)
Volume/Å^3	5077.9(6)
Z	2
ρ_{calc}g/cm^3	1.066
μ/mm^{-1}	0.457
F(000)	1740.0
Crystal size/mm^3	0.155 × 0.088 × 0.033
Radiation	CuKα (λ = 1.54184)
2Θ range for data collection/°	6.998 to 129.73
Index ranges	-17 ≤ h ≤ 19, -19 ≤ k ≤ 19, -22 ≤ l ≤ 23
Reflections collected	28050
Independent reflections	16365 [R_{int} = 0.0480, R_{sigma} = 0.0540]
Data/restraints/parameters	16365/15/1166
Goodness-of-fit on F^2	1.021
Final R indexes [I>=2σ (I)]	R_1 = 0.0603, wR_2 = 0.1555
Final R indexes [all data]	R_1 = 0.0848, wR_2 = 0.1739
Largest diff. peak/hole / e Å^{-3}	0.27/-0.27
CCDC deposition number	2062652

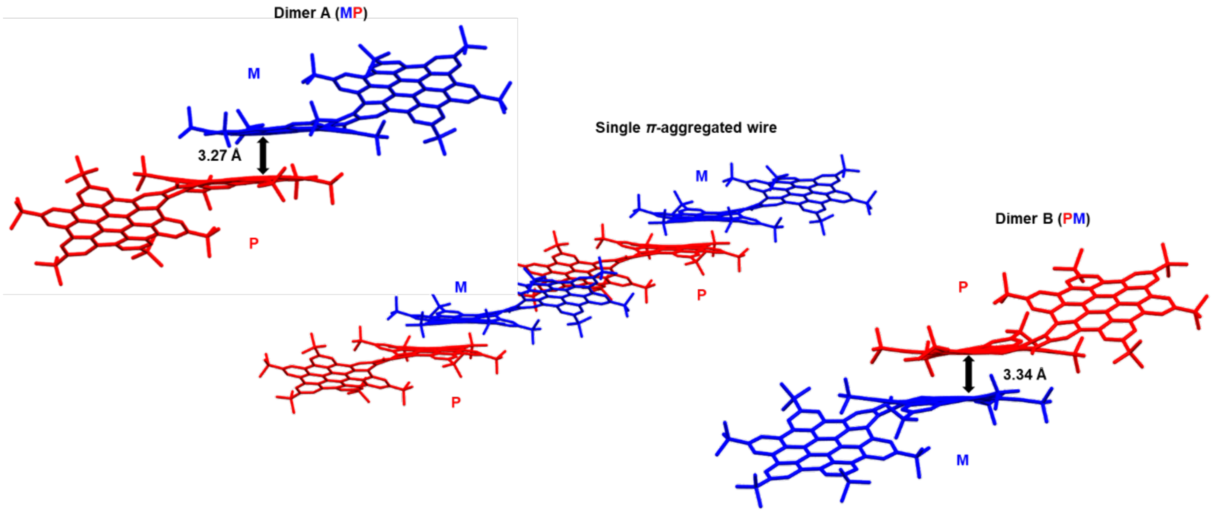

Figure S 40. Single heterochiral column (middle) of **16** which is formed by 2 alternating *π*-*π* aggregated dimers. Dimer A (MP) (top left) and dimer B (bottom right). Hydrogen atoms and disordered solvent molecules omitted for clarity.

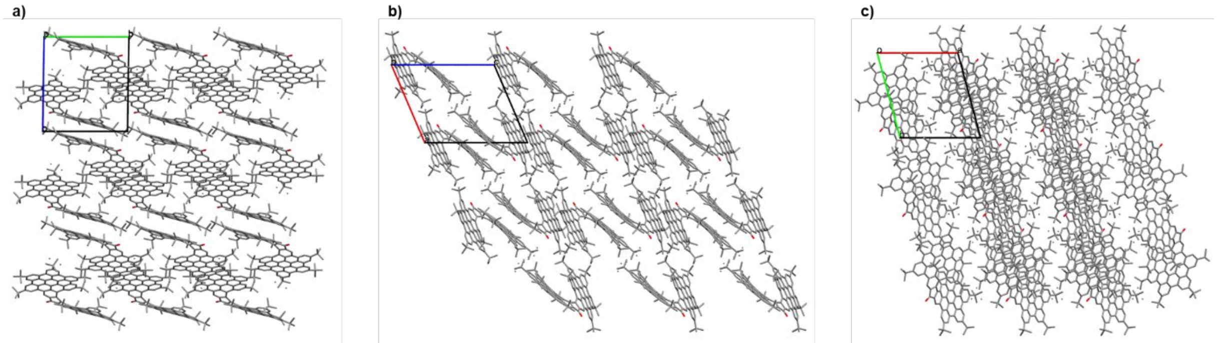

Figure S 41. Different perspectives on the crystal packing of **16**: a) View along the crystallographic a-axis; b) view along the crystallographic b-axis; c) view along the crystallographic c-axis. Hydrogen atoms and disordered solvent molecules omitted for clarity.

5 Charge carrier mobilities

5.1 Methods

The charge transport is modelled in the hopping regime (assuming weak-coupling).[S12,S13,S14,S15] Whilst this approximation is sufficient to uncover qualitative trends across calculated charge-carrier mobilities, we cannot expect the calculated mobilities to quantitatively match those of experimental mobility measurements, because our calculations assume charges to be fully localized at a given site and do not account for transport occurring somewhere in the intermediate regime between a hopping and band regime[S14], nor do we consider electron-phonon interactions.[S16] However, our hopping-based approach has proven to be successful for many molecular materials in indicating suitability as organic semiconductors, for example PCBM,[S17] spiro-OMeTADs[S18] (substituted) pentacenes[S17,S19,S20] and several [6]helicenes[S21,S22,S23,S24]

We used the DFTB Hamiltonian with self-consistent charges (SCCs) to periodically minimize the X-ray resolved crystal structures. The SCC convergence tolerance was set to 1xE^{-10} a.u. with a maximum of 1000 SCC iterations and Broyden mixing parameter was set to 0.99. The 3ob-3-1 general purpose Slater-Koster parameters were used together with Grimme's D3 dispersion correction. The Brillouin zone was homogeneously sampled using the 1x1x1 Monkhorst-Pack scheme using DFTB+.[S25]

The single molecule in the asymmetric unit was extracted from the DFTB-D3 relaxed crystal and projected according to the crystal's symmetry operations to give rise to a 3×3×3 supercell. The transfer integrals between all molecules in the unit cell with all surrounding molecules were calculated within a center-of-mass distance cut-off of 25 Å, given the large size of the superhelicenes. The transfer integrals were calculated at B3LYP/6-31G(d) level of theory and by means of the projective method in Gaussian16[S26], which involves projecting the orbitals of a pair of molecules onto a basis set defined by the unperturbed orbitals of the individual molecules.[S27] The charge transfer rates were calculated with non-adiabatic semi-classical Marcus theory[S28] with an outer reorganization energy of 0.30 eV and a master equation approach. The absolute mobilities depend on the reorganization energy, one component of which, the outer-sphere reorganization energy, cannot be computed explicitly and hence was given with a conservatively low value of λ_{outer} = 0.30 eV. The

outer-sphere reorganization energy does not vary strongly with morphology,[S29] but is dependent on the dielectric properties of the solvent and the size of the ions.[S30] For these very similar systems, we thus used a constant outer reorganization energy λ_{outer} across all three crystals.

5.2 Mobility calculation procedure

A 3x3x3 supercell was created from the DFTB relaxed crystal. The charge hopping rate from molecule *i* to *j* is given by the Marcus equation:

$$\Gamma_{ij} = \frac{|J_{ij}|^2}{\hbar}\sqrt{\frac{\pi}{\lambda k_b T}}\,exp\left(-\frac{(\Delta E_{ij} + \lambda)^2}{4\lambda k_b T}\right)$$

J_{ij} is the transfer integral, ΔE_{ij} is the change in energy between molecules *i* and *j*, λ is the reorganization energy. The reorganization energy is the sum of the energy cost associated with the rearrangement of the molecules involved in the hop (λ_{inner}) and their surroundings (λ_{outer}). λ_{inner} is calculated with the four-point method:[S31]

$$\lambda_{inner} = (E_n^{+/-} - E_c^{+/-} + E_c^0 - E_n^0)$$

The subscript n and c denote the single molecule being optimized at the charged or neutral geometry. The superscript indicates the single point energy computed for the charged (+/-) and neutral (0) state. The outer reorganization energy is harder to quantify, and thus was approximated to 0.3 eV for all superhelicenes studied. The maximum transfer integral J_{max} computed was J_{max} = 95 meV and λ was always higher than 0.39 eV, the weak coupling assumption (J << λ) was still valid. Across all superhelicenes, the maximum transfer integral was less than 100 meV.

The change in energy between the sites is:

$$\Delta E_{ik} = \varepsilon_i - \varepsilon_j - qF \cdot r_{ij}$$

The energies of the frontier orbitals for molecule *i* and *j* are given by ε_i and ε_j. The charge of an electron is denoted with q, the field vector F and r_{ij} distance vector

between the two sites *i* and j. Energetic disorder is discarded here, thus $\varepsilon_i = \varepsilon_j$ and ΔE_{ij} reduces down to:

$$\Delta E_{ij} = -qF \cdot r_{ij}$$

The angular dependent mobility was computed by applying a field strength of 10^4 Vcm^{-1} at 10° intervals in each of the 3 planes (ab, bc, ad) by solving the Master equation:

$$\frac{dP_i}{dt} = \sum_{i \neq j} \left[\Gamma_{ij} P_i(t) - \Gamma_{ji} P_j(t)\right]$$

Where Γ_{ij} is the hopping rate between sites i and j, P_i is the probability of site i being occupied and the Master equation is solved in the steady state: AP=0 with A defined as:

$$A_{ij} = \begin{cases} \Gamma_{ij}, i \neq j \\ -\sum_{i \neq j} \Gamma_{ij}, i = j \end{cases}$$

Using singular value decomposition, one can find the null space of the A matrix, which corresponds to the steady state probabilities. Then, therefrom the average velocity of charges is found according to:

$$\vec{v} = \sum_{i \neq j} \vec{r_{ij}}\, A_{ij} P_j$$

The mobility is computed from the velocities with $\mu = \frac{\langle v \rangle}{F}$ where $\langle v \rangle$ is the average velocity of charges in the direction of the field and F the applied electric field.

5.3 Results

The major results for fluorenone-superhelicene **16** are discussed in the paper itself yet some additional information is given here.

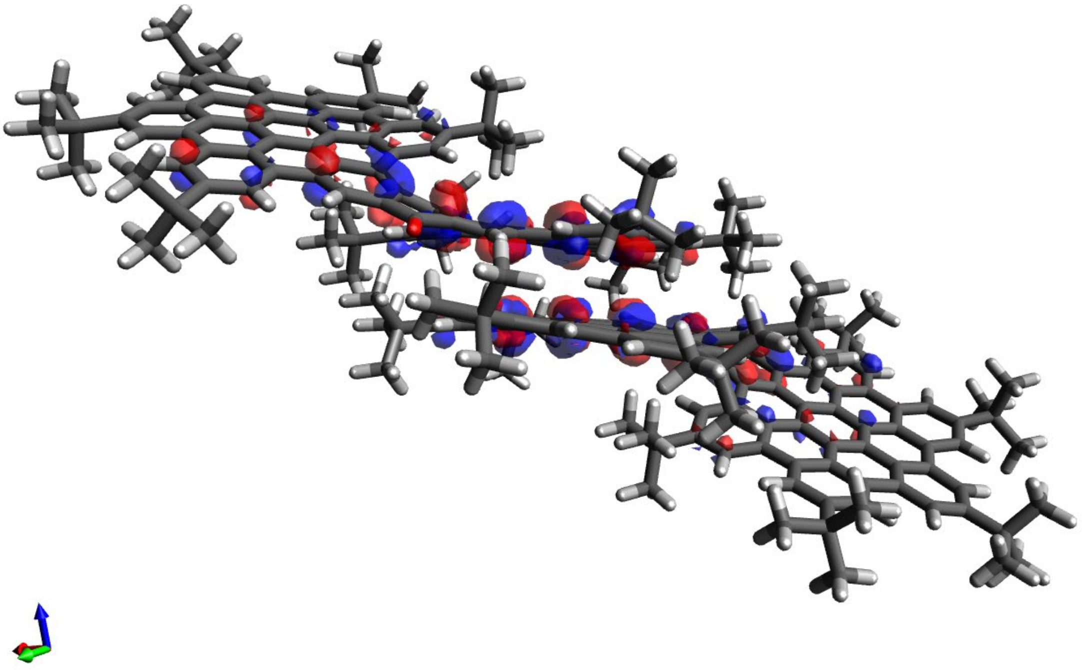

Figure S 42. Representation of the HOMO for the **16** J_{max} = 94 meV dimer (isovalue 0.02). The HOMO is delocalized over both units.

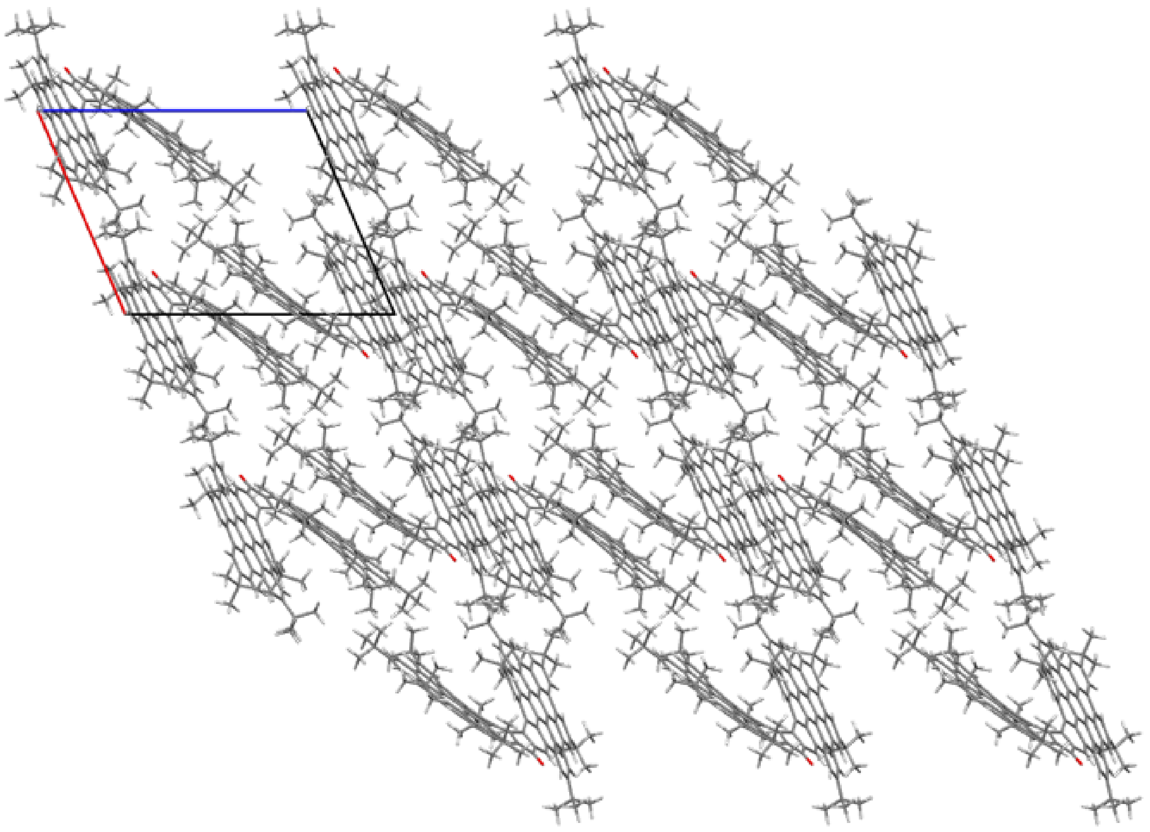

Figure S 43. *ac*-plane of the crystal structure of 16 – clearly showing the J_{max}*(hole)* dimer to propagate along the c-axis (blue).

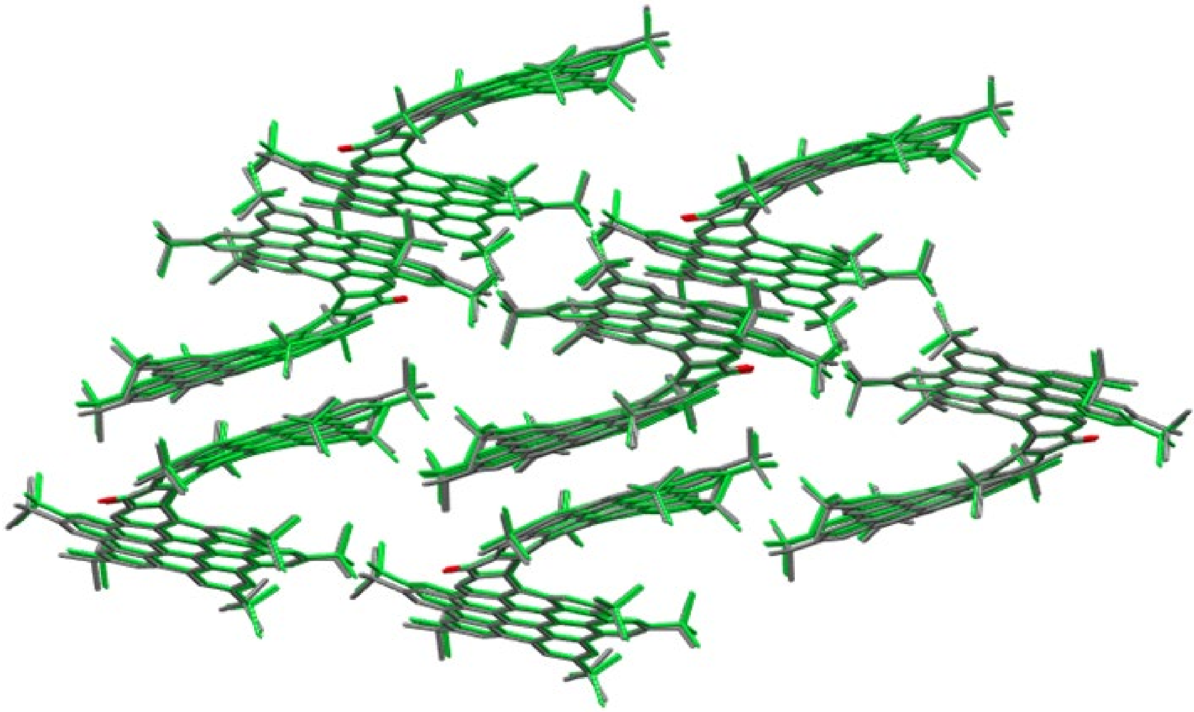

Figure S 44. Structure overlay of the DFTB-D3 relaxed crystal structure of **16** with DFT-D3 relaxed crystal structure (PBE-D3/TZV2P-MOLOPT-GTH using CP2K). RMSD = 0.279, matching 15/15 molecules in the neighboring shell. Hydrogen atoms are omitted for clarity.

The results for the previously reported crystal structure of **18**[S5] are discussed here. As the crystal structure of **18** had a high degree of disorder, only the highest occupancy conformation of the crystal was taken and results are therefore considered as merely preliminary. **18** has an even lower hole inner reorganization energy $\lambda_{intra}(hole)$ = 91 meV than **16**, thus the dibenzofuran stabilizes the positive charge better than the fluorenone. The lower inner reorganisation energy of **18** compared to **16**, can be rationalised by the increased π-system size of **18**. λ was experimentally proven to be inversely proportional to the inverse participation ratio (IPR) of the electron wavefunction, which quantifies charge delocalisation.[S32] For polymers, the inverse relationship between π-system size and inner-sphere reorganisation energy was confirmed for side-group functionalisation of the polymer.[S33] Hutchison et al. calculated the inner-sphere reorganisation energies of a large set of polymers, showing show that the reorganisation energy drops with the number of monomers (i.e. the number of double bonds added) to the polymer chain.[S34] Nonetheless, the hole mobility of **18** is extremely poor (μ_{max} = 0.114 $cm^2V^{-1}s^{-1}$) with an average hole mobility of 0.050 $cm^2V^{-1}s^{-1}$, due to the lack of high transfer integrals (J_{max} = 34 meV); this crystal is thus unsuitable for hole transport (Figure S45). The electron mobility of **18** reaches a maximum of 0.353 $cm^2V^{-1}s^{-1}$ in the *bc* plane. The average mobility however is only 0.120 $cm^2\,V^{-1}\,s^{-1}$.

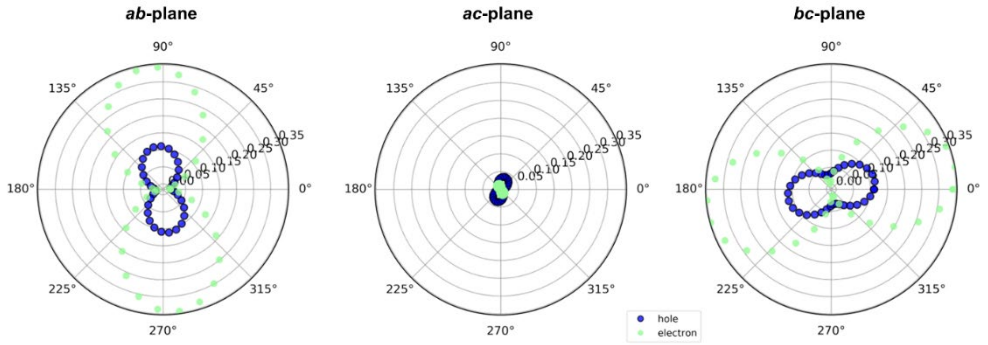

Figure S 45. Hole (blue) and electron (green) charge carrier mobility (cm^{2}V^{-1}s^{-1}) of the crystal of **18**.

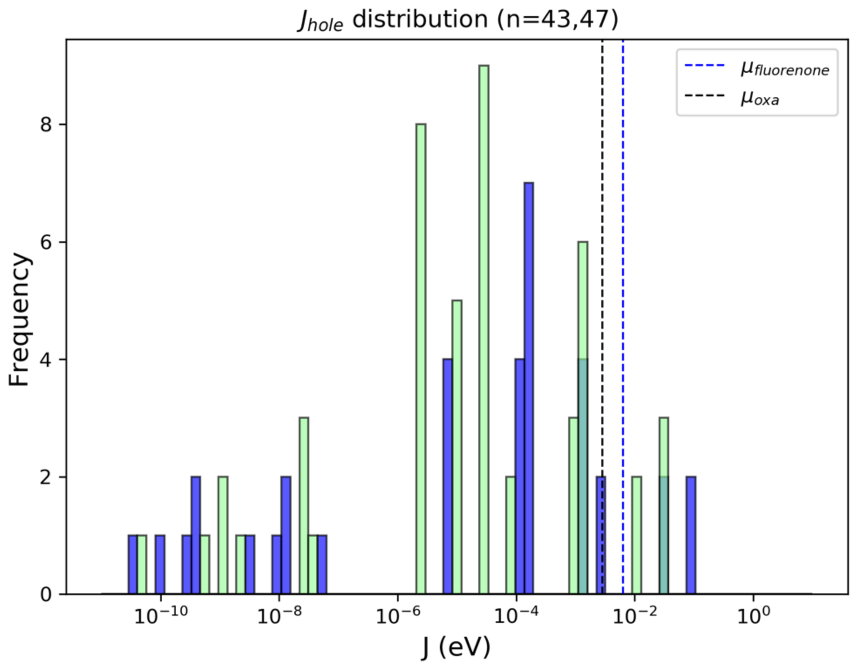

[S

Figure S 46. Distribution of **16** and **18** transfer integrals observed. The dashed lines indicate the average transfer integrals for **16** and **18**.

Table S 5. Inner reorganization energies (λ_{inner}) and maximum transfer integrals (J_{max}) observed for **16** and **18**.

	λ_{inner}**(hole)** [meV]	λ_{inner}(electron) [meV]	J_{max}**(hole)** [meV]	J_{max}(electron) [meV]
**16**	**100**	213	**95**	16
**18**	**91**	136	**34**	23

Table S 6. Hole mobility related properties.

	λ_{intra}**(hole)** [meV]	Min μ [$cm^2V^{-1}s^{-1}$]	Max μ [$cm^2V^{-1}s^{-1}$]	Average μ [$cm^2V^{-1}s^{-1}$]
**16**	100	0.006	3.741	1.561
**18**	91	0.004	0.114	0.050

Table S 7. Electron mobility related properties.

	λ_{intra}**(electron)** [meV]	Min μ [$cm^2V^{-1}s^{-1}$]	Max μ [$cm^2V^{-1}s^{-1}$]	Average μ [$cm^2V^{-1}s^{-1}$]
**16**	213	0.000	0.030	0.012
**18**	136	0.001	0.353	0.120

Best values per column are shown in green, worst values in red. "Best" meaning lowest possible inner reorganization energy and highest mobility and for "worst" vice versa.

Table S 8. A selection of publications with simulated charge carrier mobilities ($cm^2V^{-1}s^{-1}$) with their respective reorganization energies (eV).

λ_{intra} [eV]	$\lambda_{outer.}$ [eV]	λ $=\lambda_{intra}+\lambda_{outer}$ [eV]	μ_{hole} [$cm^2V^{-1}s^{-1}$]	$\mu_{electron}$ [$cm^2V^{-1}s^{-1}$]	Molecule	Related Publication
0.160	**0.140**	0.3	**0.0-2.0**	0.1-2.9	carbo[6]helicene	[S21]/[S22]
0.170	**0.130**	0.3	**0.058**	-	(*rac*)-aza[6]helicene	[S23]
0.170	**0.130**	0.3	**0.032**	-	(+)-aza[6]helicene	[S23]
0.138	**0.362**	0.5	**0.33**	-	TIPS-pentacene	[S20]
0.142	**0.358**	0.5	**0.28**	-	TES-pentacene	[S17]
0.200	**0.000**	0.2	**-**	4.73	PCBM	[S17]
0.200	**0.000**	0.2	**-**	0.92	Bis-PCBM	[S17]
0.200	**0.000**	0.2	**-**	0.29	Tris-PCBM	[S17]
0.147-0.216	**0.300**	0.4-0.5	**0.016-0.256**	0.031-0.297	X-aza[6]helicenes X=[1,16]	[S24]

6 Enantiomeric separation

The enantiomers of **16**, **17**, **18** and **19** were separated via HPLC chromatography over a Chiralpak IA column with *n*-hexane/*iso*-propanol/THF solvent mixtures.

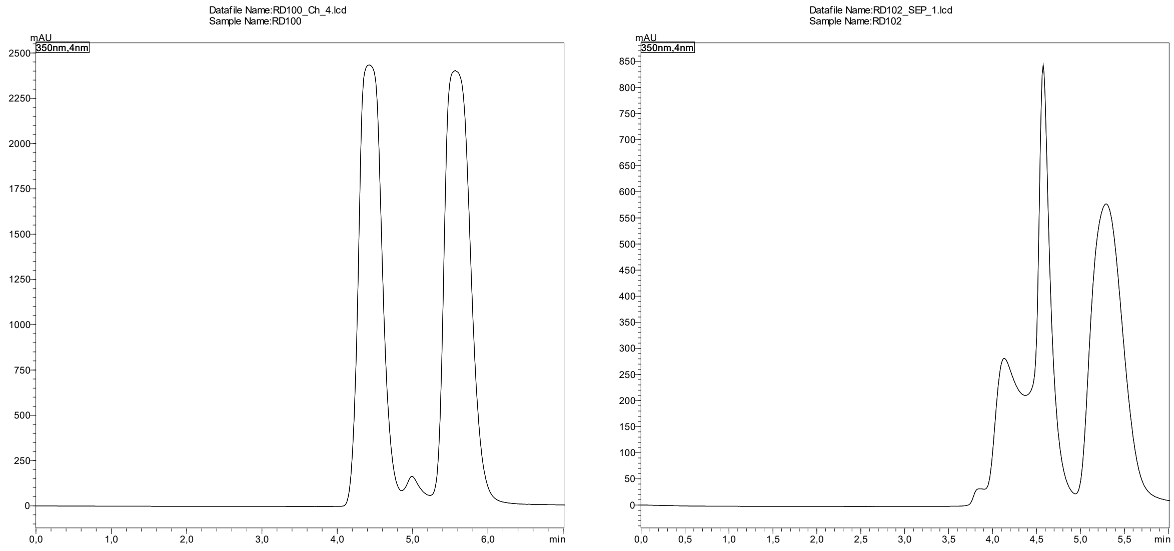

Figure S 47. Left: HPLC profile of the enantiomeric separation of fluorenone-superhelicene **16**. Chiralpak IA, *n*-hexane/*iso*-propanol/THF 95/3/2, 1mL/min, 30 °C. Right: HPLC profile of the enantiomeric separation of fluorene-superhelicene **17**. Chiralpak IA, *n*-hexane/*iso*-propanol/THF 95/2/3, 1mL/min, 30 °C. Note that the split of the first peak does not correspond to impurities but stems from the injection in toluene that washes down some of the first enantiomer earlier than the rest.

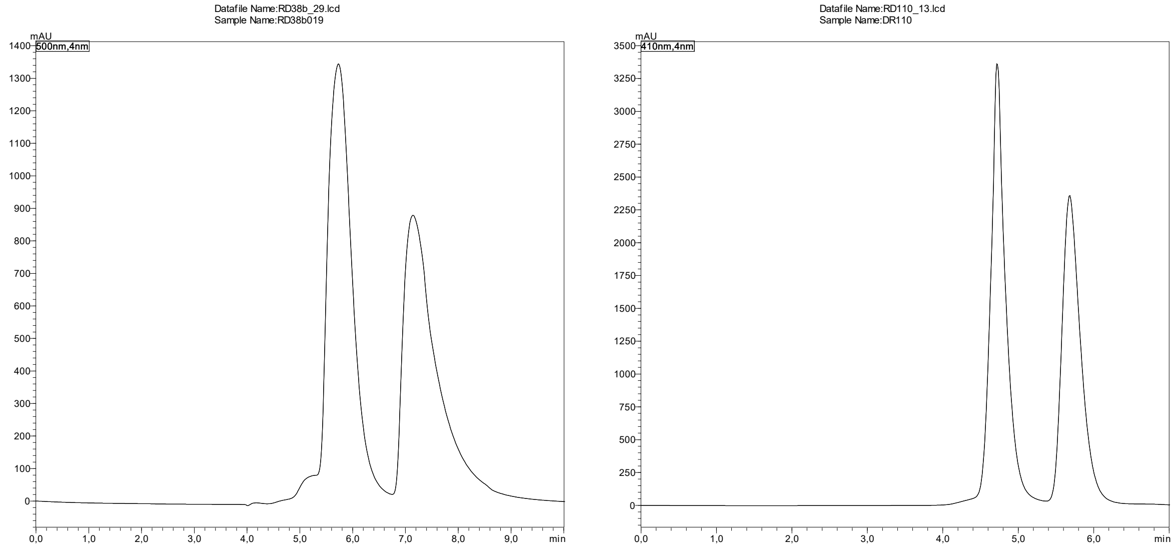

Figure S 48. Left: HPLC profile of the enantiomeric separation of oxa-[7]-superhelicene **18**. Chiralpak IA, *n*-hexane/*iso*-propanol/THF 98.3/0.2/1.5, 1mL/min, 30 °C. Right: HPLC profile of the enantiomeric separation of thia-[7].superhelicene **19**. Chiralpak IA, *n*-hexane/*iso*-propanol/THF 97/1/2, 1mL/min, 30 °C.

7 Assignment of the enantiomers

7.1 Computational details

The geometry optimizations were carried out with the Turbomole program package,[S35] while the spectra were calculated using the Orca program.[S36] In order to calculate the spectra more efficiently only the four *tert*-butyl groups closest to the helical part (see Figure S 49) were taken into account, as these influence the structure of the helical part. The other *tert*-butyl groups were replaced by hydrogen atoms. The structures were obtained using the following procedure: First several conformations of the four *tert*-butyl groups of **17** were calculated. The most stable conformation was then also used in the optimization of the other studied superhelicenes (**16**, **18**-**23**). Furthermore, a derivative containing a central benzene ring (**30**) instead of a five-membered ring was calculated for comparison. All geometry optimizations were performed using the PBE[S37] functional and the def2-TZVP[S38] basis set. The resolution of identity approximation[S39,S40] in conjunction with multipole approximation[S41] was used for these optimizations. The SCF-energies and densities were converged to 10^{-7} a.u., the energy change between geometry optimization steps to 10^{-6} a.u. and the maximum norm of the gradient with respect to change of atomic positions to 10^{-4} a.u. For all calculations dispersion interactions were included via the semi-empirical D3 dispersion interaction correction developed by Grimme and coworkers,[S42] including Becke-Johnson dampening.[S43]

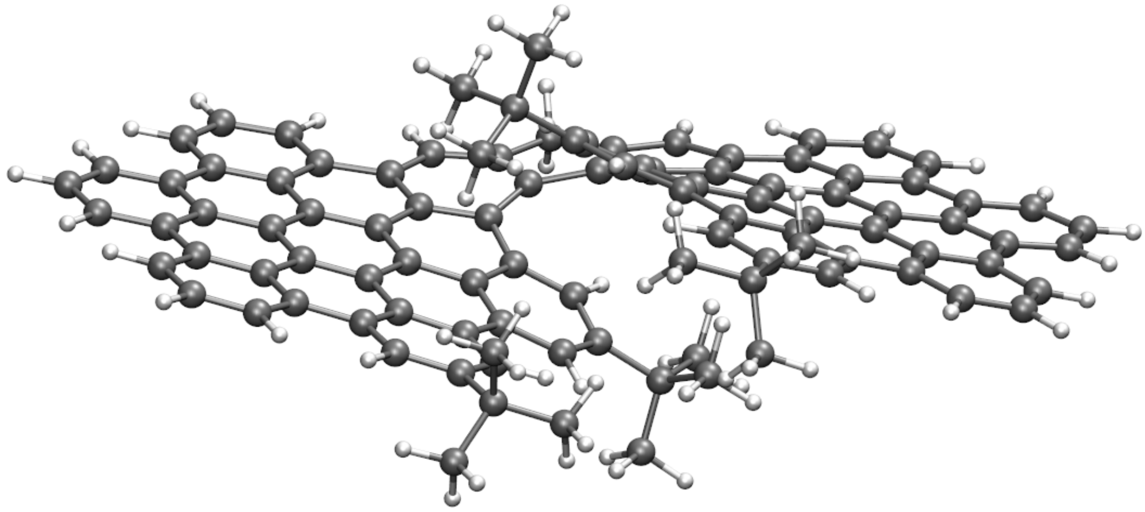

Figure S 49. Optimized structure of the used derivative of **17**. In order to increase the computational efficiency only the four inner *tert*-butyl groups were taken into consideration as they play an important role in the helical distortion of the π-system. The other six *tert*-butyl groups were replaced by hydrogen atoms.

Frequency calculations were performed using the NumForce[S44] module of Turbomole in order to confirm that the obtained structures are local minima (no imaginary frequencies) or reoptimize them if they were not. The HOMOs and LUMOs depicted in Figures S59 – S67 were obtained by single point calculations using the B3-LYP[S45] functional and def2-TZVP basis set. The UV/VIS and CD spectra of **16**, **17**, **18**, and **19** were then calculated using the range separated ωB97-D3[S46] functional. The spectra were calculated with two different methods/basis sets. First spectra using the simplified time-depended density-functional theory[S47,S48] (sTDDFT) approach developed by Grimme and coworkers in combination with the def2-TZVP basis set was used. The energy threshold for these calculations was set to 12 eV. The second method was using standard TDDFT[S49,S50] calculations of the first 250 excitations, which were carried out using the def2-SVP[S38] basis set. For both methods the resolution of identity approximation for the Coulomb part in combination with the "chain of spheres" (COSX) approximation[S51] for the exchange part (RIJCOSX method[S51]) was used. The DFT grids were "GRID5" for the SCF iterations and "FINALGRID6" for the final SCF calculation. GRIDX8 was used for the COSX part. The SCF convergence criteria "TightSCF" was used for both methods. The spectra were shifted by a constant shift (determined for each spectrum individually) in order to align the maximum of the calculated UV-Vis spectrum with the right peak of the most intense band at 364 nm in case of **16.** In case of **17, 18** and **19** the middle peak of the most intense band was used (374 nm, 375 nm and 378 nm, respectively). The individual shifts are given in the description of Figure S 50, S 52, S 54 and S56. The first method reproduces the experimental spectra better in the area below 400 nm while the second method reproduces the area above 400 nm better. Both methods lead to the same assignment though. Solvent effects (CH_2Cl_2: ε = 8.93,[S52] refractive index = 1.4242)[S53] were included via the conductor-like screening (COSMO) method[S54] for the calculations with Turbomole and via the Conductor-like Polarizable Continuum Model[S55] (CPCM) using the COSMO epsilon function, which is the COSMO implementation of ORCA for the spectra. Cartesian coordinates of the structures used for calculating the spectra can be found separately as xyz-files.

7.2 Calculated structural features

Based on the calculated structures several features were determined which are typical for analysing the structure of helicenes. If possible, those were compared to the corresponding values obtained by crystal structure analysis (Table S 9).

Table S 9. Structural parameters (interplanar angle and torsion angles) of the synthesised helicenes **16**-**23** and their comparison to an analogous helicene containing a central benzene ring instead of a five-membered ring (**30**). If available, the calculated values are compared to the corresponding values obtained by x-ray structure analysis.

Compound	Interplanar angle [°]	Torsion angles [°]<br>a / b / c / d / e<br>**Sum**
**16**	26.8<br><br>X-ray:<br>32.8	22.9 / 16.4 / 23.1 / 16.6 / 22.7<br>**101.7**<br><br>X-ray:<br>20.0 / 14.8 / 27.3 / 17.1 / 17.5<br>**96.7**
**17**	38.3	13.5 / 15.6 / 23.8 / 15.5 / 13.5<br>**81.9**
**18**	29.9<br><br>X-ray:<br>46.2	20.6 / 17.0 / 17.9 / 17.7 / 20.4<br>**93.6**<br><br>X-ray:<br>14.2 / 21.2 / 22.5 / 16.1 / 12.4<br>**86.4**
**19**	27.0	22.2 / 19.5 / 20.6 / 19.5 / 22.2<br>**104.0**
**20**	29.0	21.0 / 17.6 / 19.2 / 17.2 / 21.1<br>**96.1**
**21**	26.6	23.0 / 17.0 / 23.1 / 16.3 / 23.6<br>**103.0**
**22**	26.9	22.6 / 18.5 / 21.6 / 18.4 / 22.7<br>**103.8**
**23**	26.3	24.3 / 16.8 / 23.8 / 19.5 / 22.1<br>**106.5**
**30 (central benzene ring)**	18.9	25.7 / 25.8 / 25.6 / 25.5 / 25.9<br>**128.5**

It is observed that the central atom/functionality, which is exchanged, can have a significant influence on the structural parameters of the helicenes. **17** exhibits a large calculated interplanar angle of 38.3 ° and a sum of all torsion angles of 81.9 ° while for example 21 shows an interplanar angle of 26.6 ° and a sum of all torsion angles of 103.0 °. This might in parts be explained by the hybridization of the central atom in the five-membered ring. Generally, the interplanar angles and torsion angles behave

inverse, meaning that compounds with larger interplanar angles exhibit a smaller sum of all torsion angles. **30**, containing the central benzene ring differs significantly from the derivatives with the five membered rings, exhibiting the smallest interplanar angle (18.9 °) and the largest sum of all torsion angles (128.5 °).

Comparing calculated and measured structures it is apparent that the interplanar angles are significantly higher for the measured ones. In contrast to that, the sum of all torsion angles is smaller. Also, the torsion angles are much more symmetrical for the calculated structure, meaning that a ≈ e and b ≈ d. This shows that effects of crystal packing have significant influence on the structure of the compounds. However, it needs to be considered, that, as stated above, the structures were calculated without the six *tert*-butyl groups pointing outwards and only the four pointing inside the helix were taken into account. This might also have an influence on the structures despite of the *tert*-butyl groups not being close to the interior helix.

7.3 Comparison and assignment of measured and calculated spectra

The four enantiomerically separated compounds **16**, **17**, **18** and **19** were investigated by means of CD spectroscopy to get a first insight into their chiroptical properties. To assign the HPLC fractions to the respective enantiomers, CD spectra were calculated for comparison. Calculated spectra correspond to the M enantiomer.

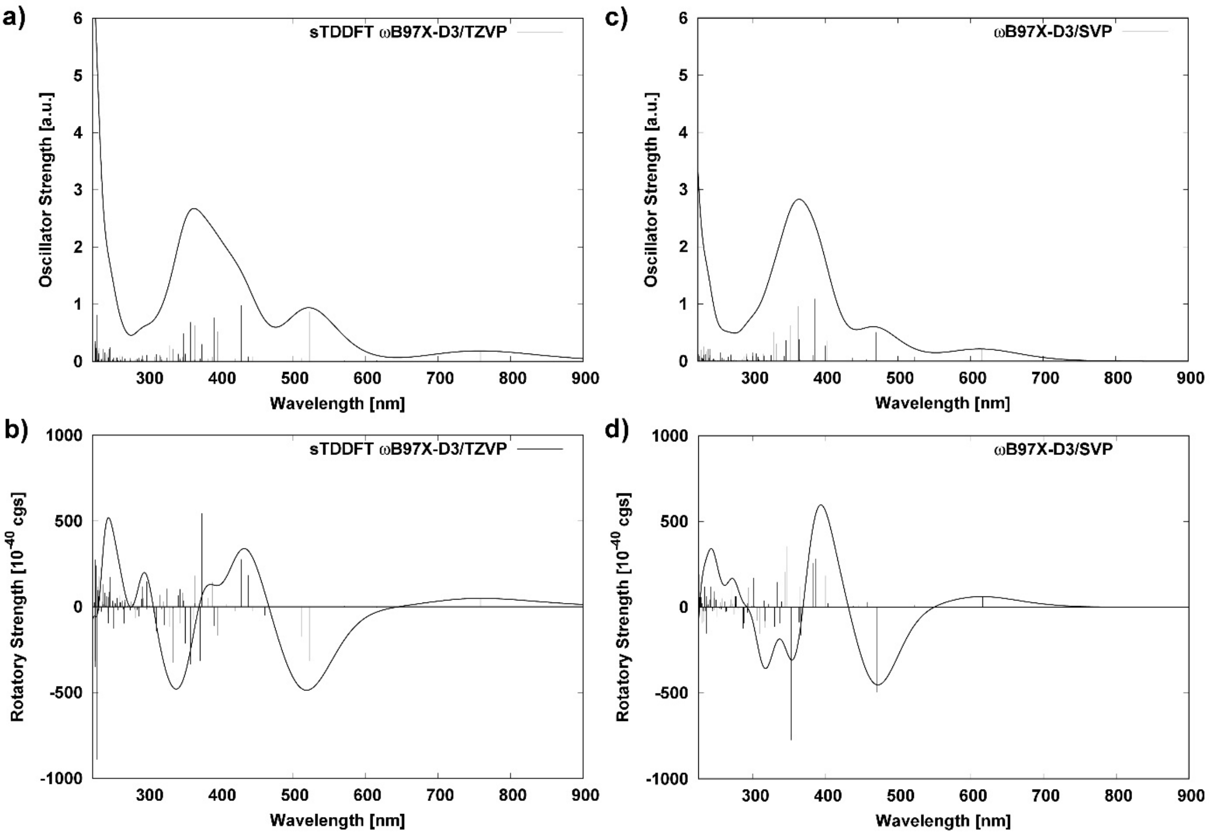

Figure S 50. Left: UV/Vis (a) and CD (b) spectra for **16** (M-enantiomer) calculated with the sTDDFT ωB97-D3/TZVP method. Both spectra corrected with a bathochromic shift of 7145 cm^{-1}. Right: UV/Vis (c) and CD (d) spectra for **16** calculated with the ωB97-D3/SVP method. Both spectra corrected with a bathochromic shift of 5301 cm^{-1}. Calculated stick spectra were broadened by a Gaussian shaped broadening (FWHM=3000 cm^{-1}). Spectrum corresponds to HPLC faction 1.

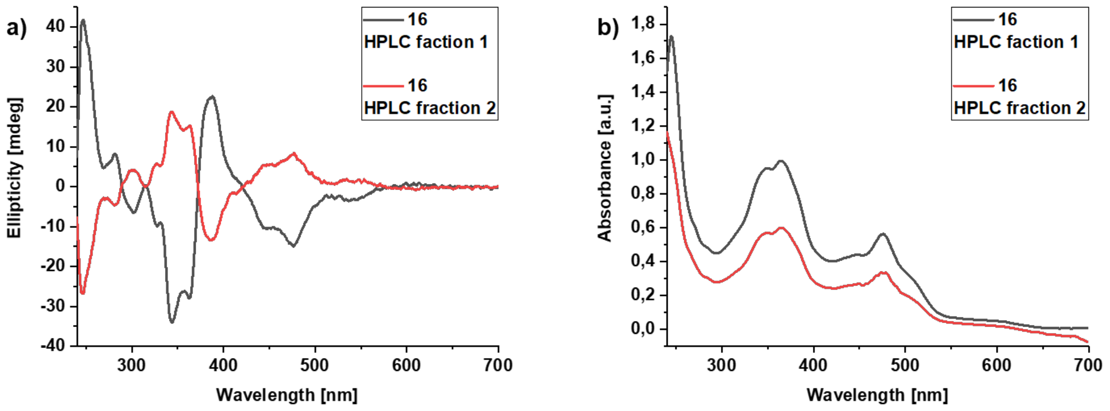

Figure S 51. Measured CD spectra in CH_2Cl_2 for the two enantiomers of **16** (a) separated via HPLC and respective UV/Vis spectra (b).

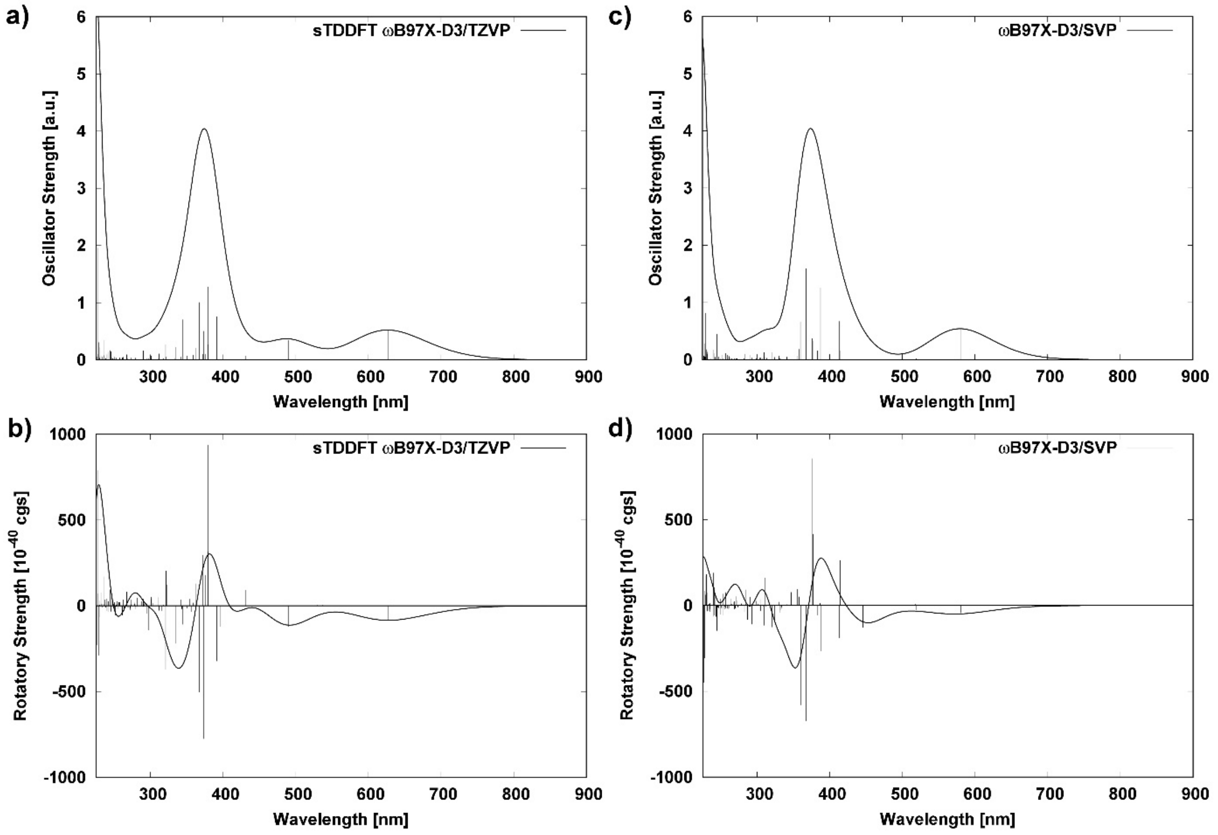

Figure S 52. Left: UV/Vis (a) and CD (b) spectra for **17** (M-enantiomer) calculated with the sTDDFT ωB97-D3/TZVP method. Both spectra corrected with a bathochromic shift of 6496 cm^{-1}. Right: UV/Vis (c) and CD (d) spectra for **17** calculated with the ωB97-D3/SVP method. Both spectra corrected with a bathochromic shift of 6588 cm^{-1}. Calculated stick spectra were broadened by a Gaussian shaped broadening (FWHM=3000 cm^{-1}). Spectrum corresponds to HPLC faction 1.

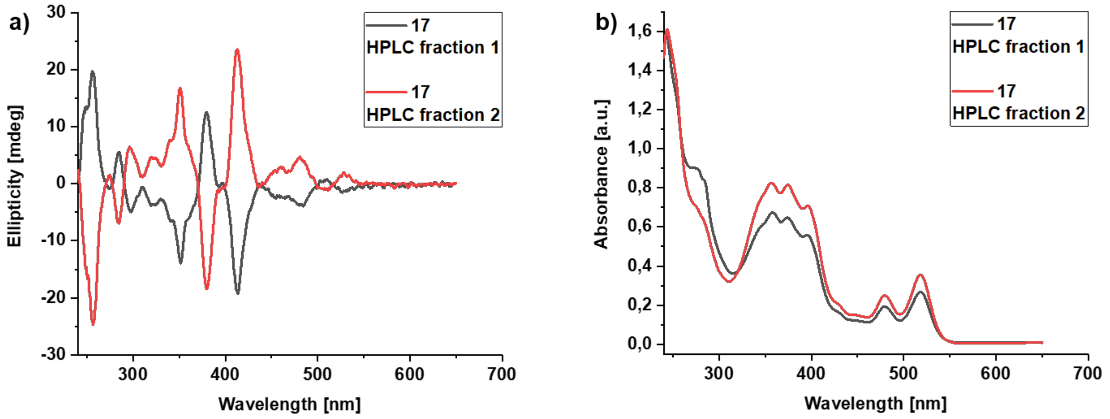

Figure S 53. Measured CD spectra in CH_2Cl_2 for the two enantiomers of **17** (a) separated via HPLC and respective UV/Vis spectra (b).

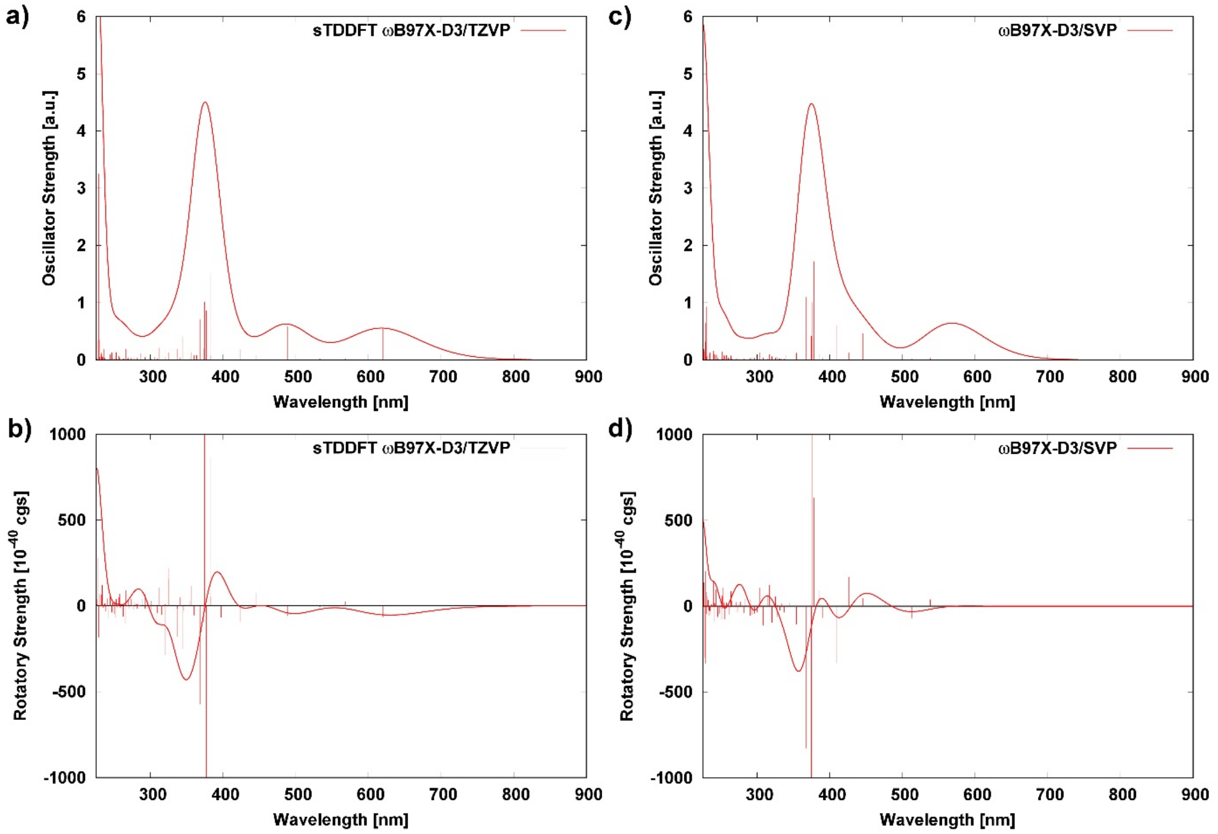

Figure S 54. Left: UV/Vis (a) and CD (b) spectra for **18** (M-enantiomer) calculated with the sTDDFT ωB97-D3/TZVP method. Both spectra corrected with a bathochromic shift of 6669 cm^{-1}. Right: UV/Vis (c) and CD (d) spectra for **18** calculated with the ωB97-D3/SVP method. Both spectra corrected with a bathochromic shift of 6716 cm^{-1}. Calculated stick spectra were broadened by a Gaussian shaped broadening (FWHM=3000 cm^{-1}). Spectrum corresponds to mirror image of HPLC faction 1.

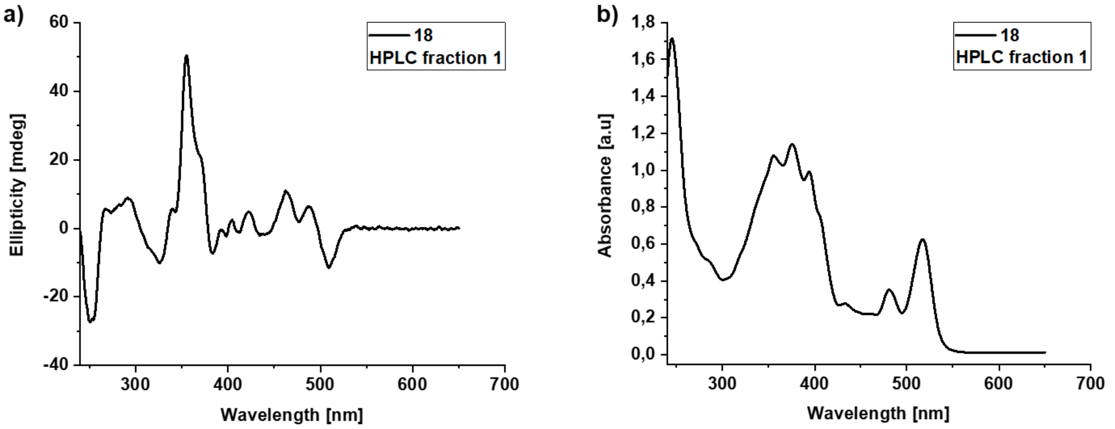

Figure S 55. Measured CD spectrum in CH_2Cl_2 for one enantiomer of **18** (a) separated via HPLC and respective UV/Vis spectrum (b).

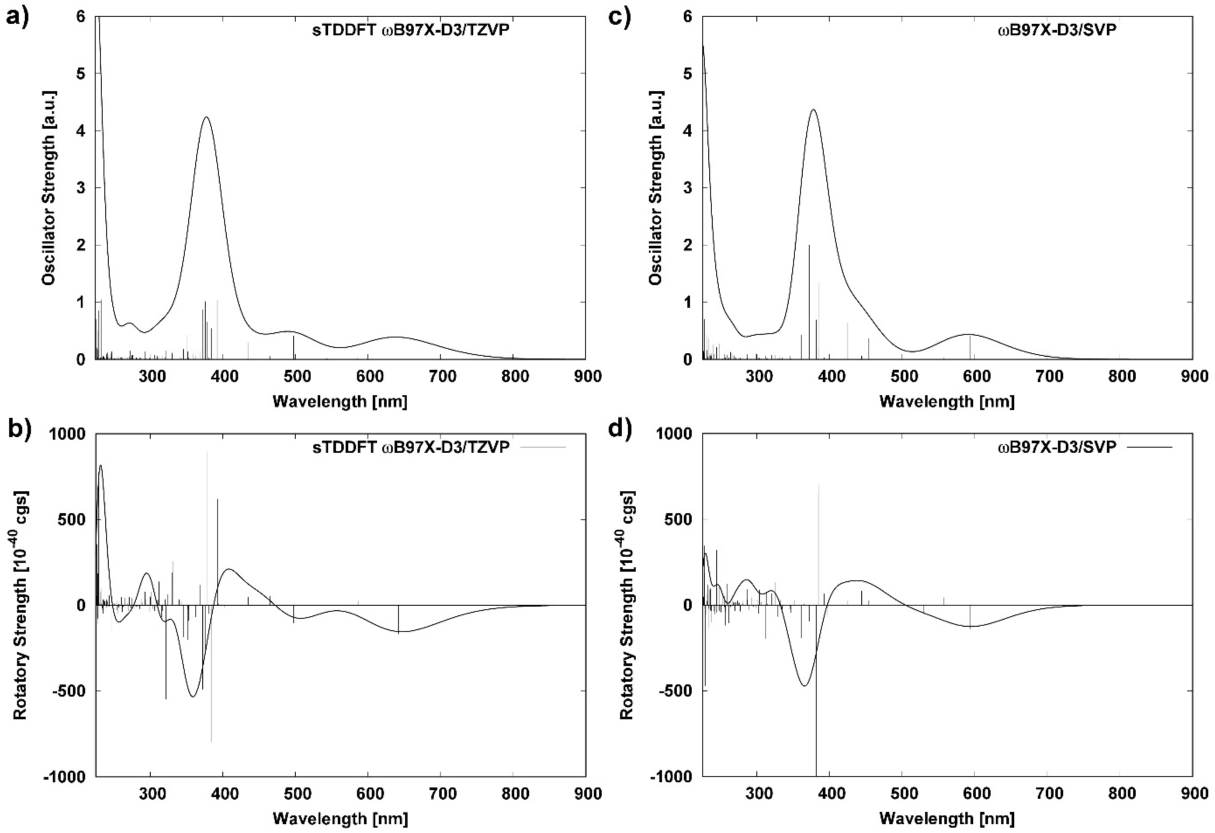

Figure S 56. Left: UV/Vis (a) and CD (b) spectra for **19** (M-enantiomer) calculated with the sTDDFT ωB97-D3//TZVP method. Both spectra corrected with a bathochromic shift of 6715 cm^{-1}. Right: UV/Vis (c) and CD (d) spectra for **19** calculated with the ωB97-D3/SVP method. Both spectra corrected with a bathochromic shift of 6840 cm^{-1}. Calculated stick spectra were broadened by a Gaussian shaped broadening (FWHM=3000 cm^{-1}). Spectrum corresponds to HPLC faction 1.

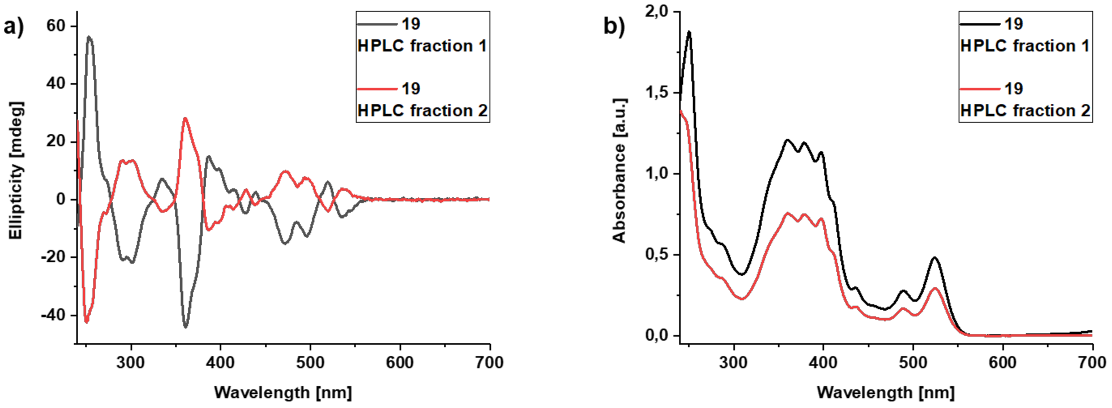

Figure S 57. Measured CD spectra in CH_2Cl_2 for the two enantiomers of **19** (a) separated via HPLC and respective UV/Vis spectra (b).

With the measured and calculated CD spectra at hand it was possible to assign the HPLC fractions to the respective enantiomers (see Table S 10).

Table S 10. Assignment of the HPLC fractions to the respective enantiomers.

Compound	HPLC fraction 1	HPLC fraction 2
**16**	**M**	**P**
**17**	**M**	**P**
**18**	**P**	**M**
**19**	**M**	**P**

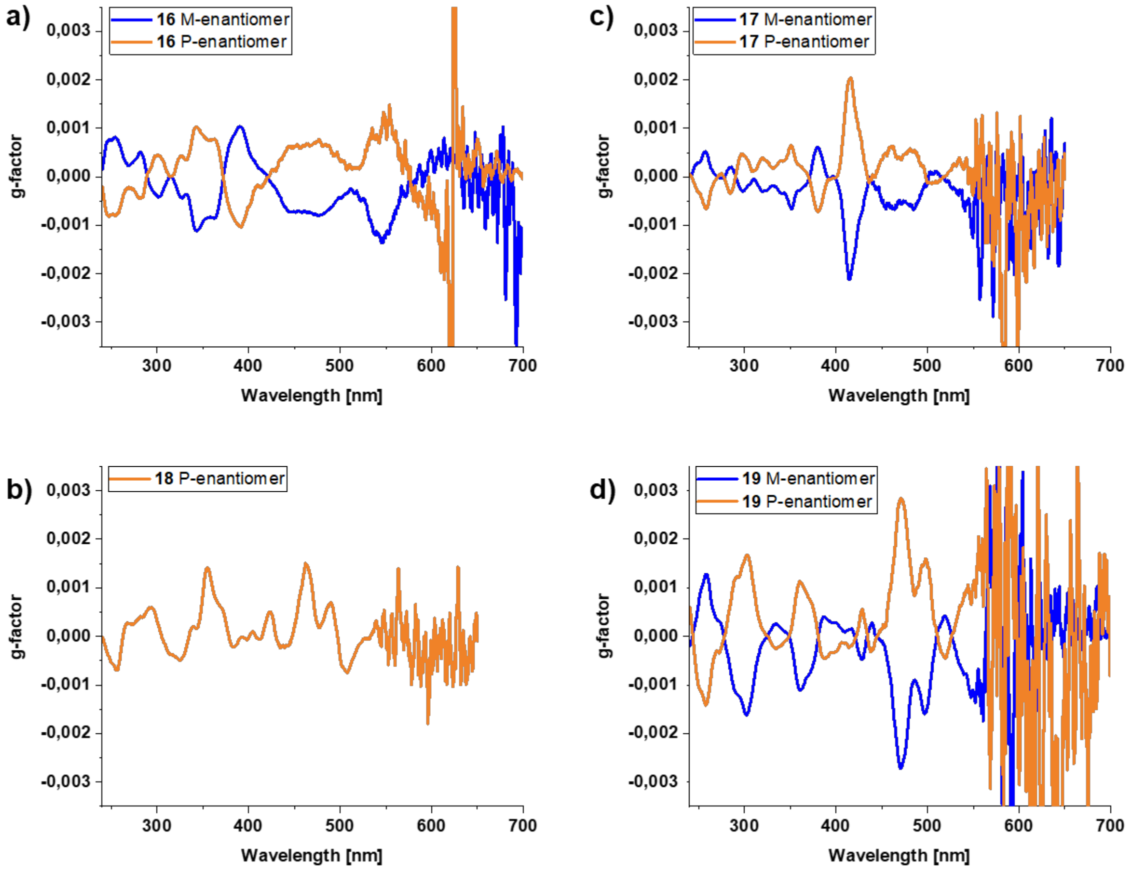

Figure S 58. Determined g-factors for the separated compounds **16**, **17**, **18** and **19**. For **18** only one enantiomer was measured.

8 Calculations of frontier molecular orbitals (FMOs)

The FMOs were calculated on the B3-LYP/def2-TZVP level of theory on the PBE/def2-TZVP geometries. See computational details in Section 7.1 for further information. The isovalues of the plotted MOs is 0.03.

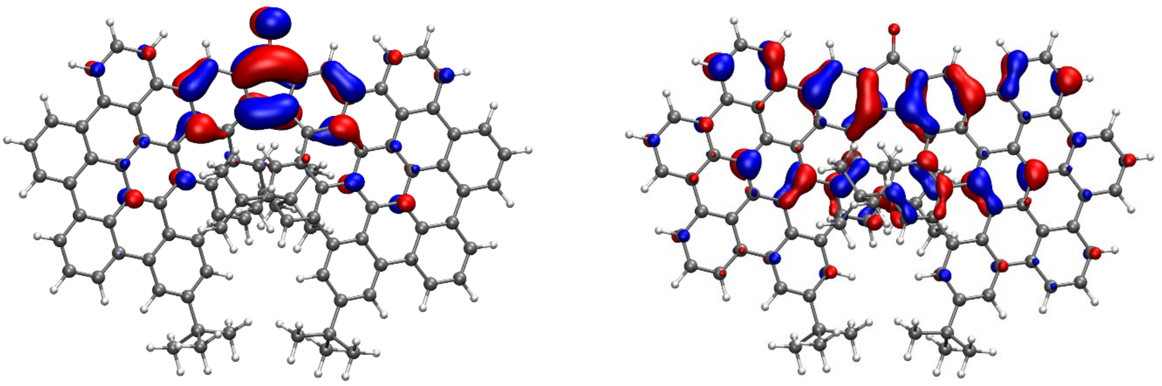

Figure S 59. FMOs of **16**. Left: LUMO (-2.82 eV); right: HOMO (-5.25 eV); gap: 2.4 eV.

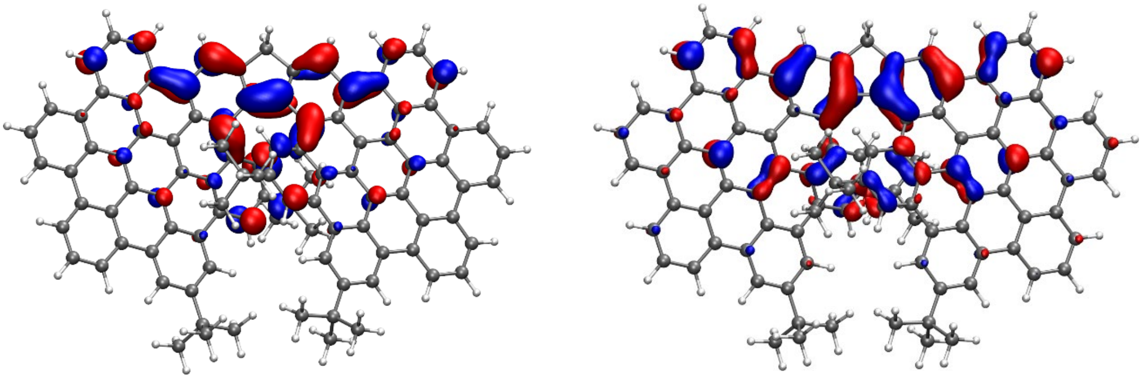

Figure S 60. FMOs of **17**. Left: LUMO (-2.39 eV); right: HOMO (-5.10 eV); gap: 2.7 eV.

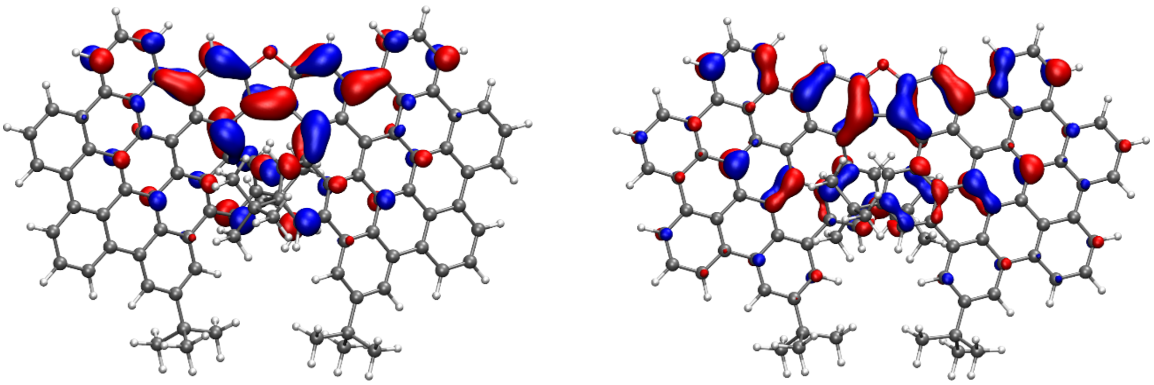

Figure S 61. FMOs of **18**. Left: LUMO (-2.47 eV); right: HOMO (-5.20 eV); gap: 2.7 eV.

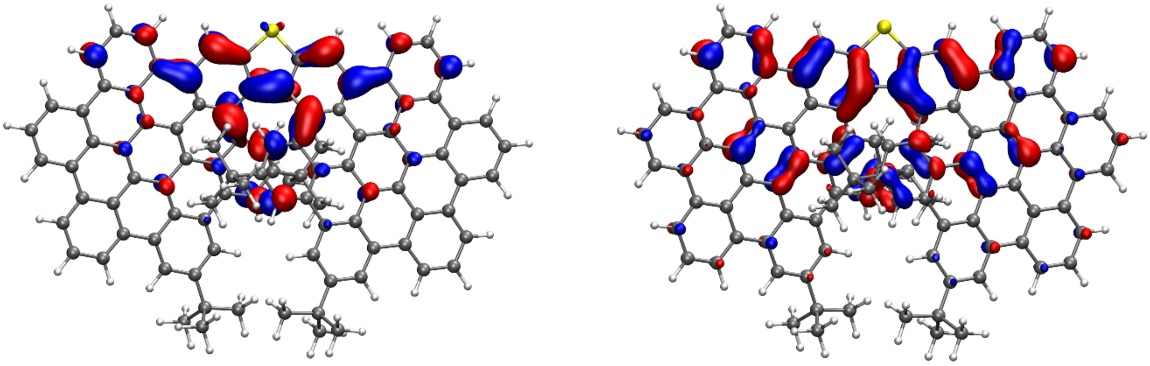

Figure S 62. FMOs of **19**. Left: LUMO (-2.50 eV); right: HOMO (-5.20 eV); gap: 2.7 eV.

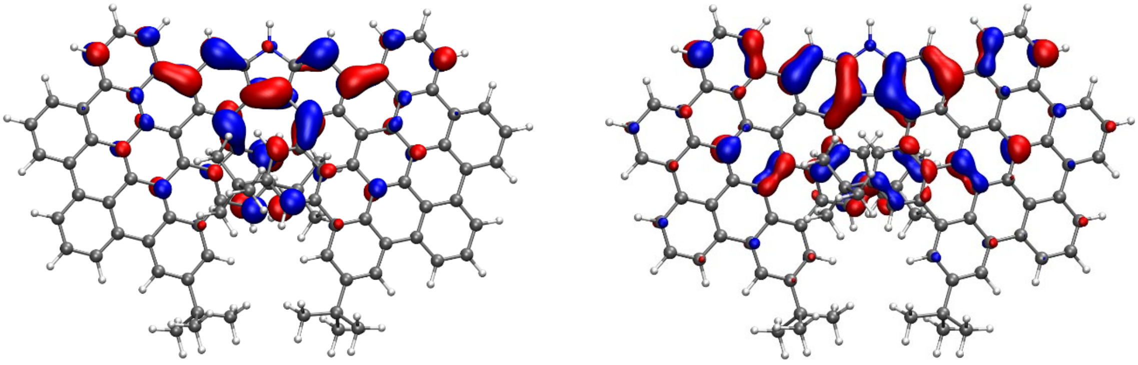

Figure S 63. FMOs of **20**. Left: LUMO (-2.37 eV); right: HOMO (-5.11 eV); gap: 2.7 eV.

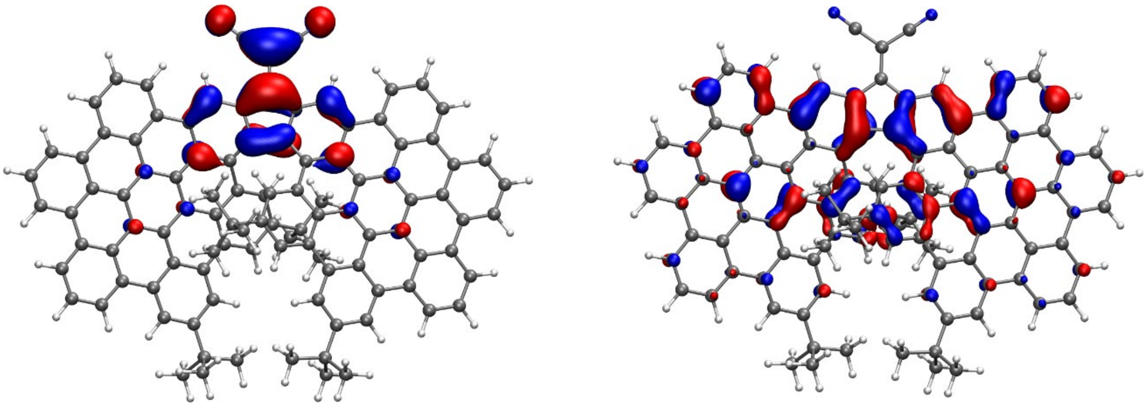

Figure S 64. FMOs of **21**. Left: LUMO (-3.42 eV); right: HOMO (-5.31 eV); gap: 1.9 eV.

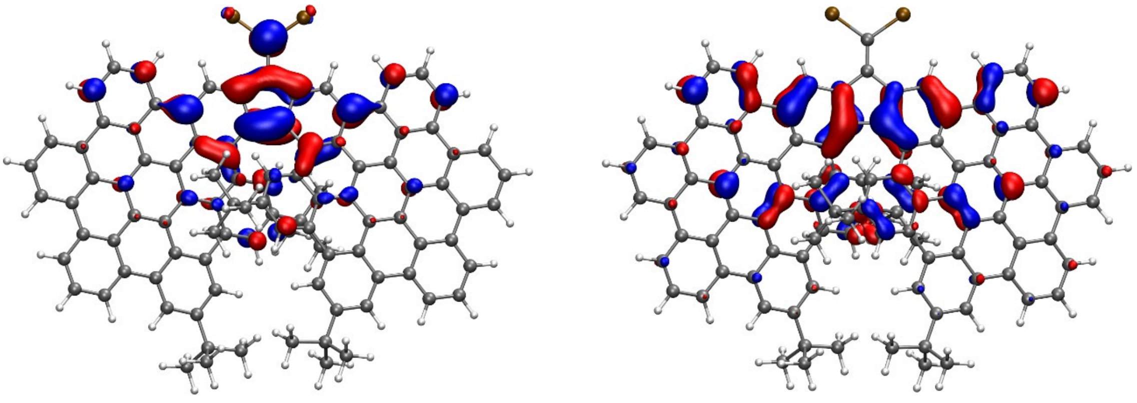

Figure S 65. FMOs of **22**. Left: LUMO (-2.59 eV); right: HOMO (-5.17 eV); gap: 2.6 eV

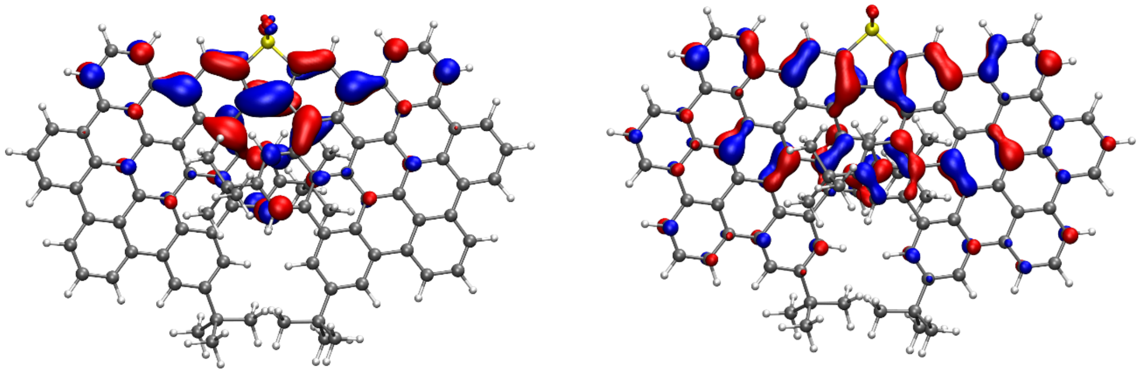

Figure S 66. FMOs of **23**. Left: LUMO (-2.71 eV); right: HOMO (-5.37 eV); gap: 2.7 eV.

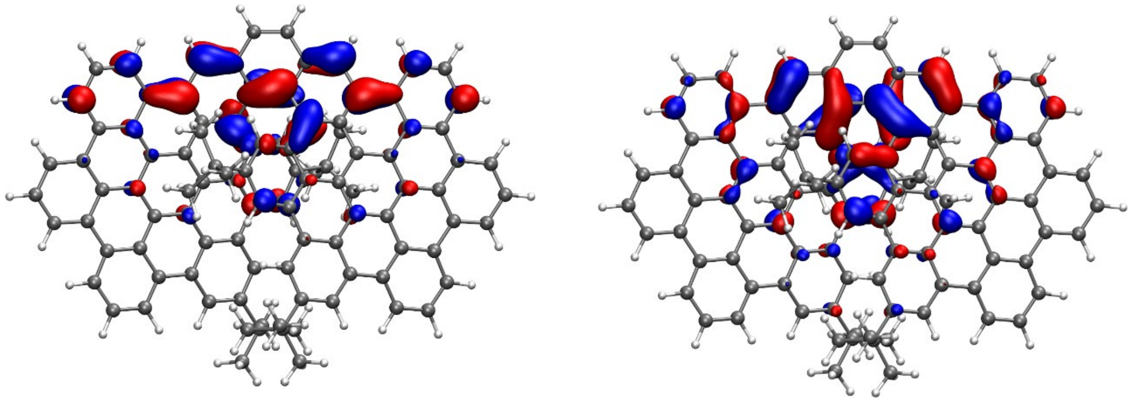

Figure S 67. FMOs of **30**. Left: LUMO (-2.37 eV); right: HOMO (-5.12 eV); gap: 2.8 eV.

9 Spectra appendix for NMR spectroscopy and MS spectrometry

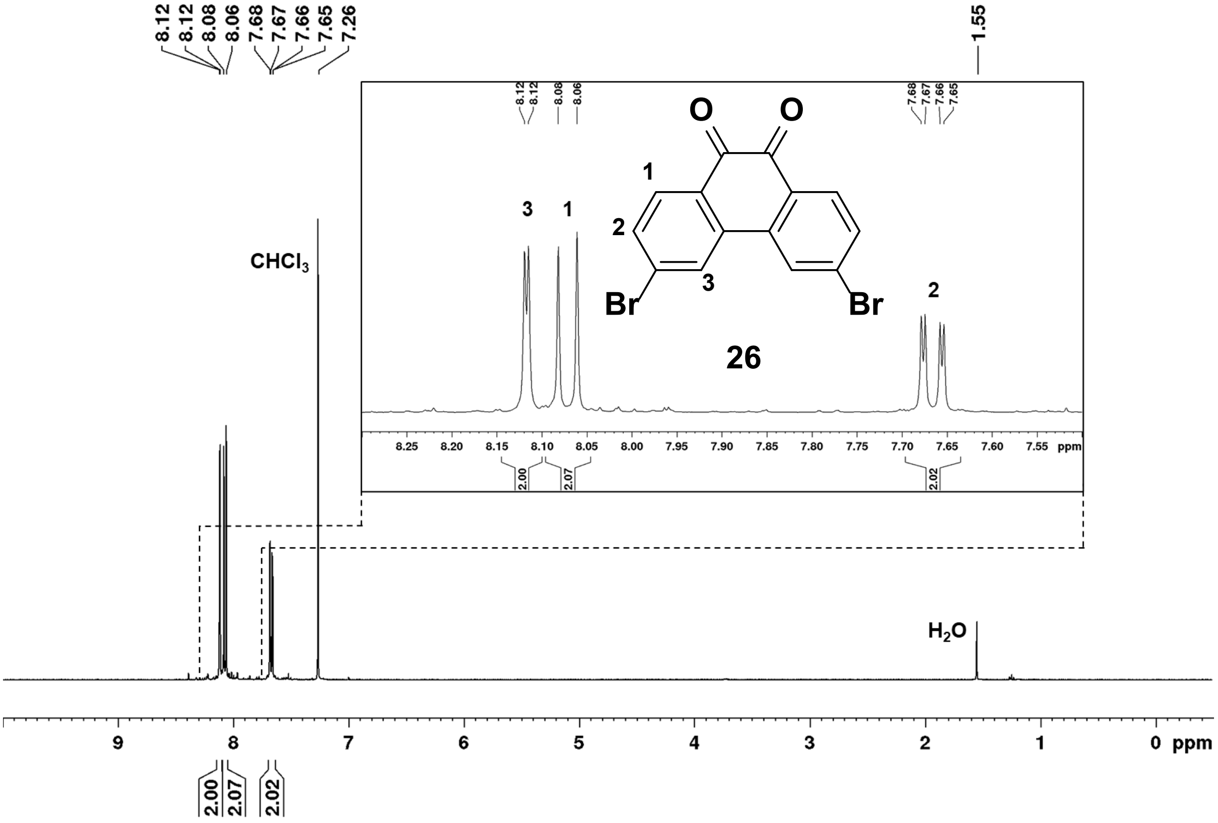

Figure S 68. ^{1}H NMR spectrum of **26** ($CDCl_3$, 400 MHz, rt.).

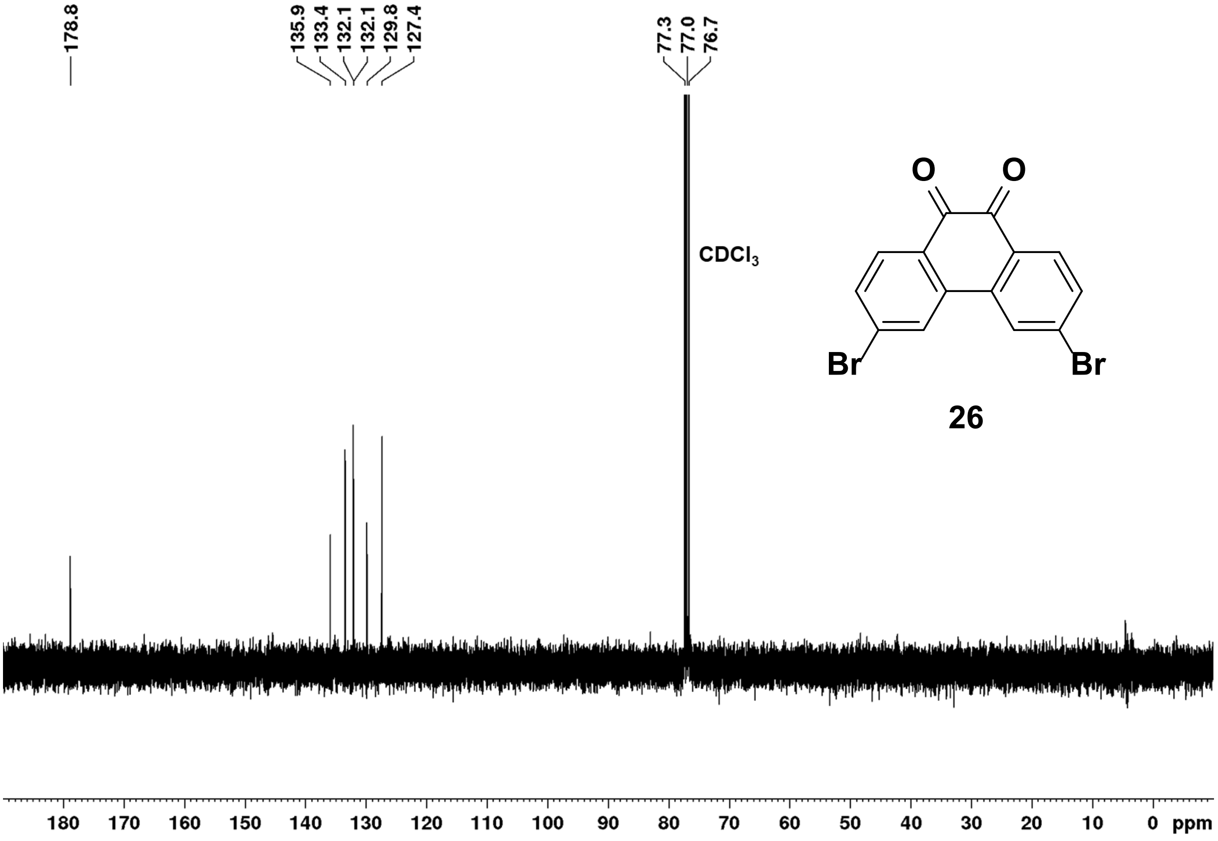

Figure S 69. ^{13}C NMR spectrum of **26** ($CDCl_3$, 100 MHz, rt.).

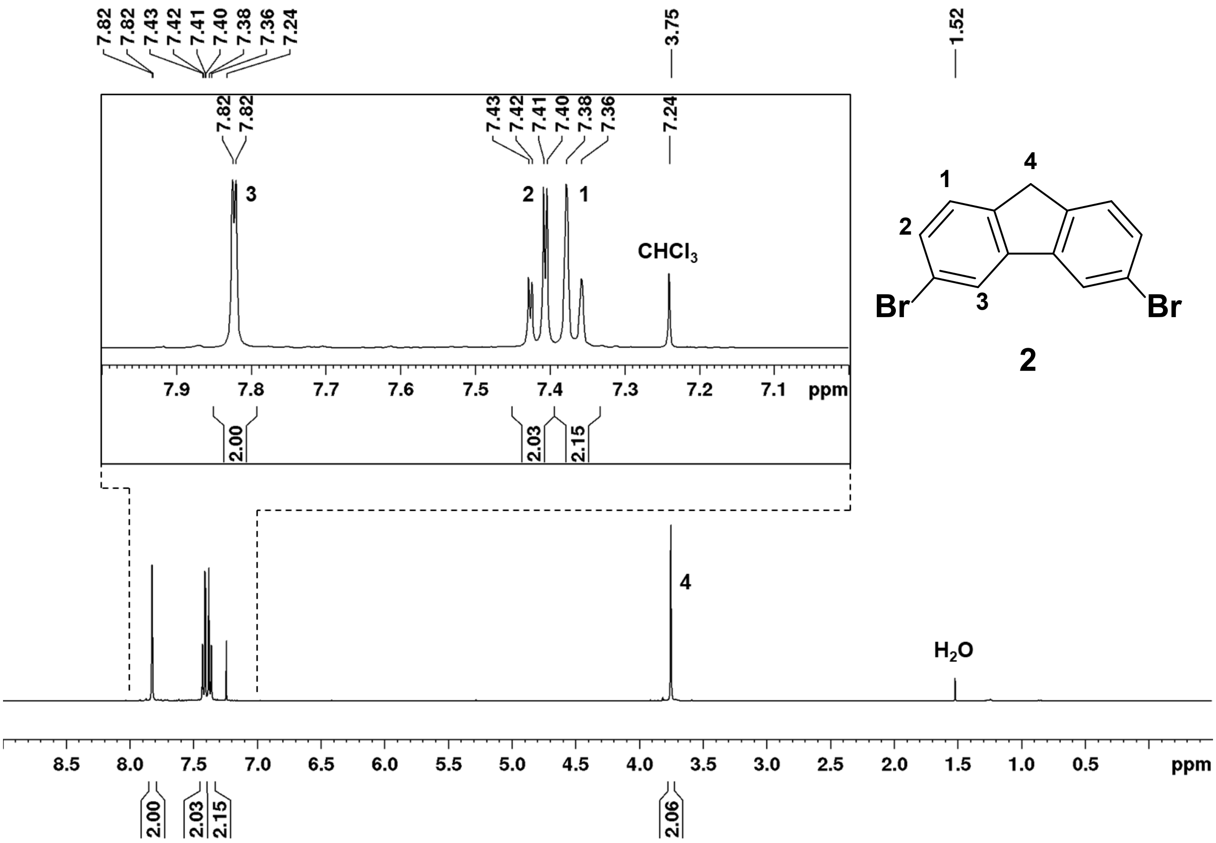

Figure S 70. ^{1}H NMR spectrum of **2** ($CDCl_3$, 400 MHz, rt.).

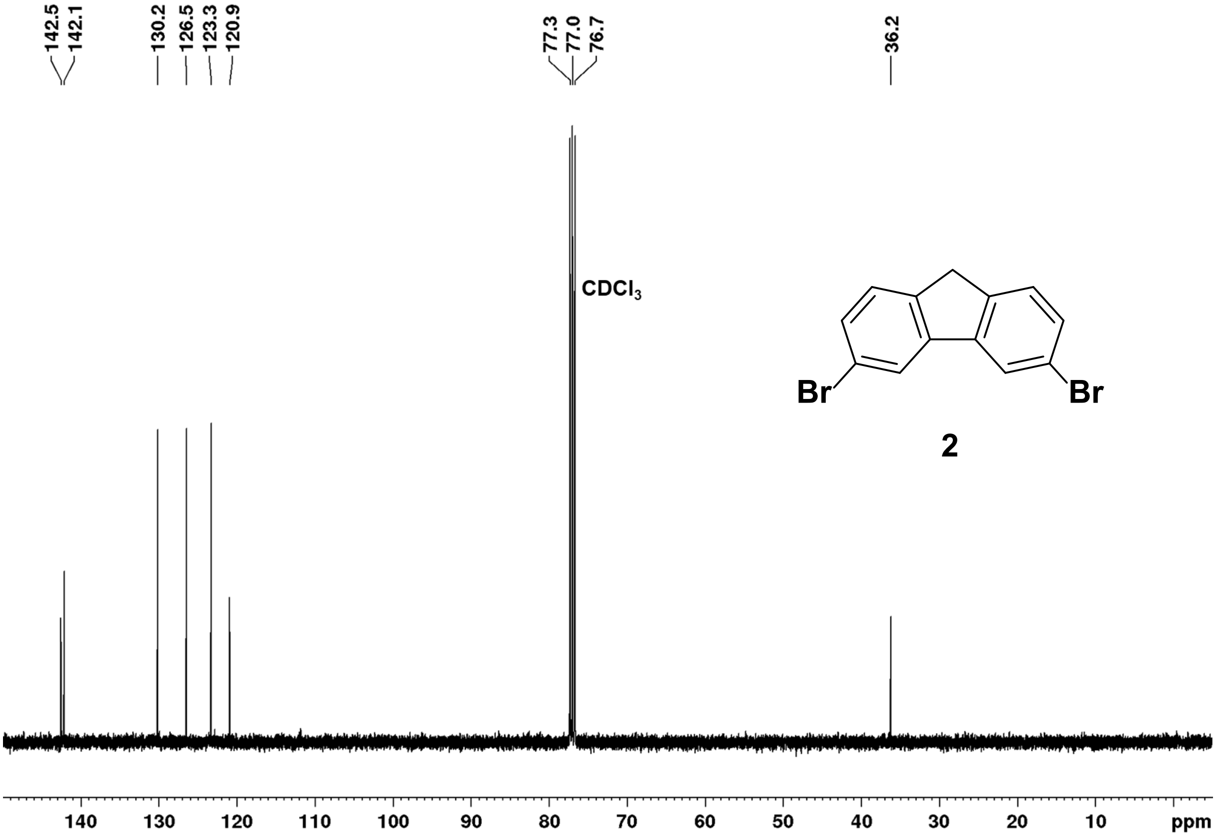

Figure S 71. ^{13}C NMR spectrum of **2** ($CDCl_3$, 100 MHz, rt.).

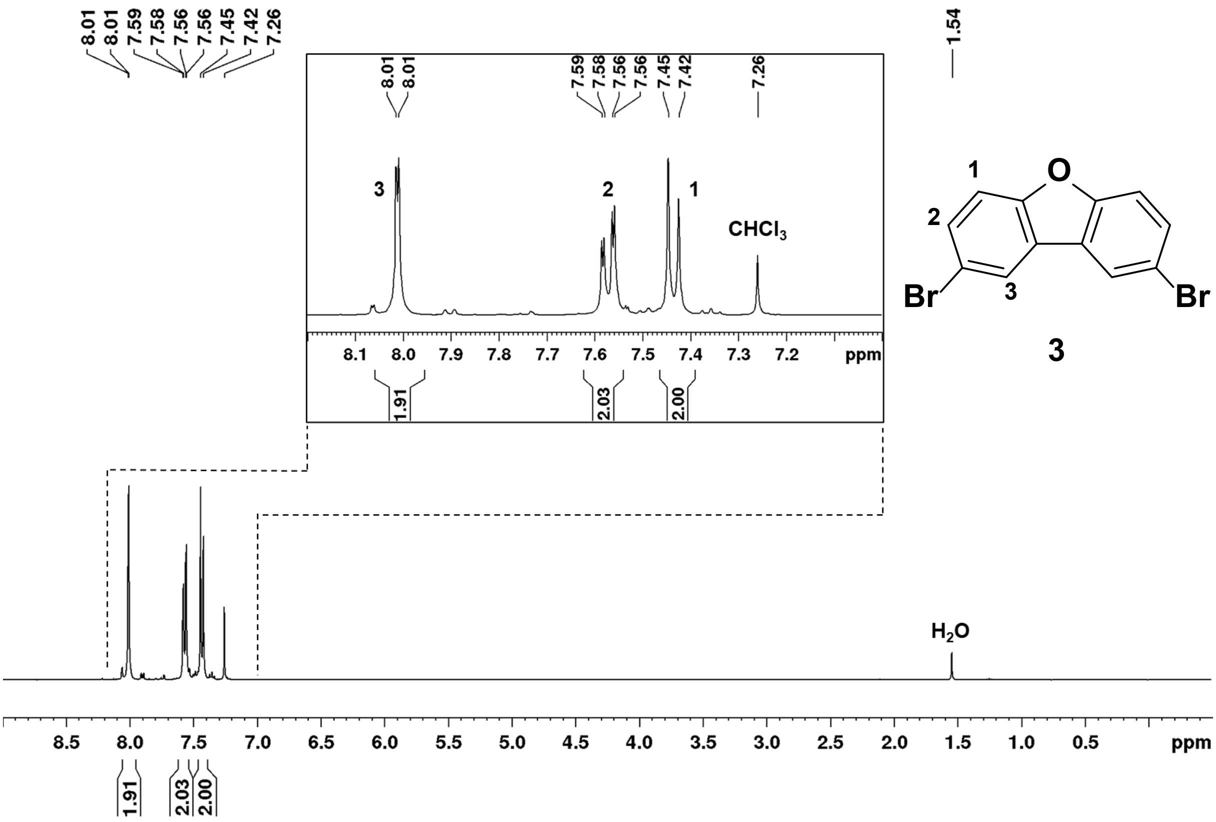

Figure S 72. ^{1}H NMR spectrum of **3** ($CDCl_3$, 400 MHz, rt.).

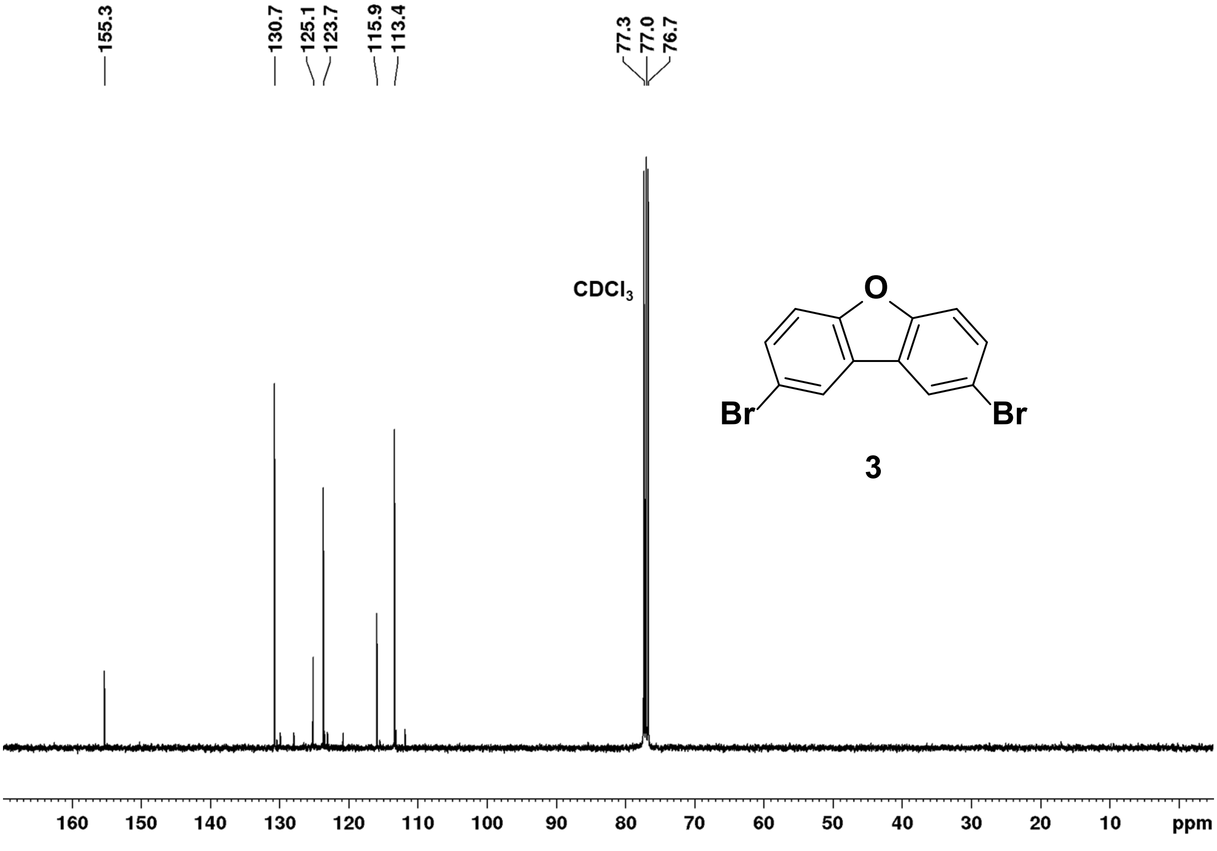

Figure S 73. ^{13}C NMR spectrum of **3** ($CDCl_3$, 100 MHz, rt.).

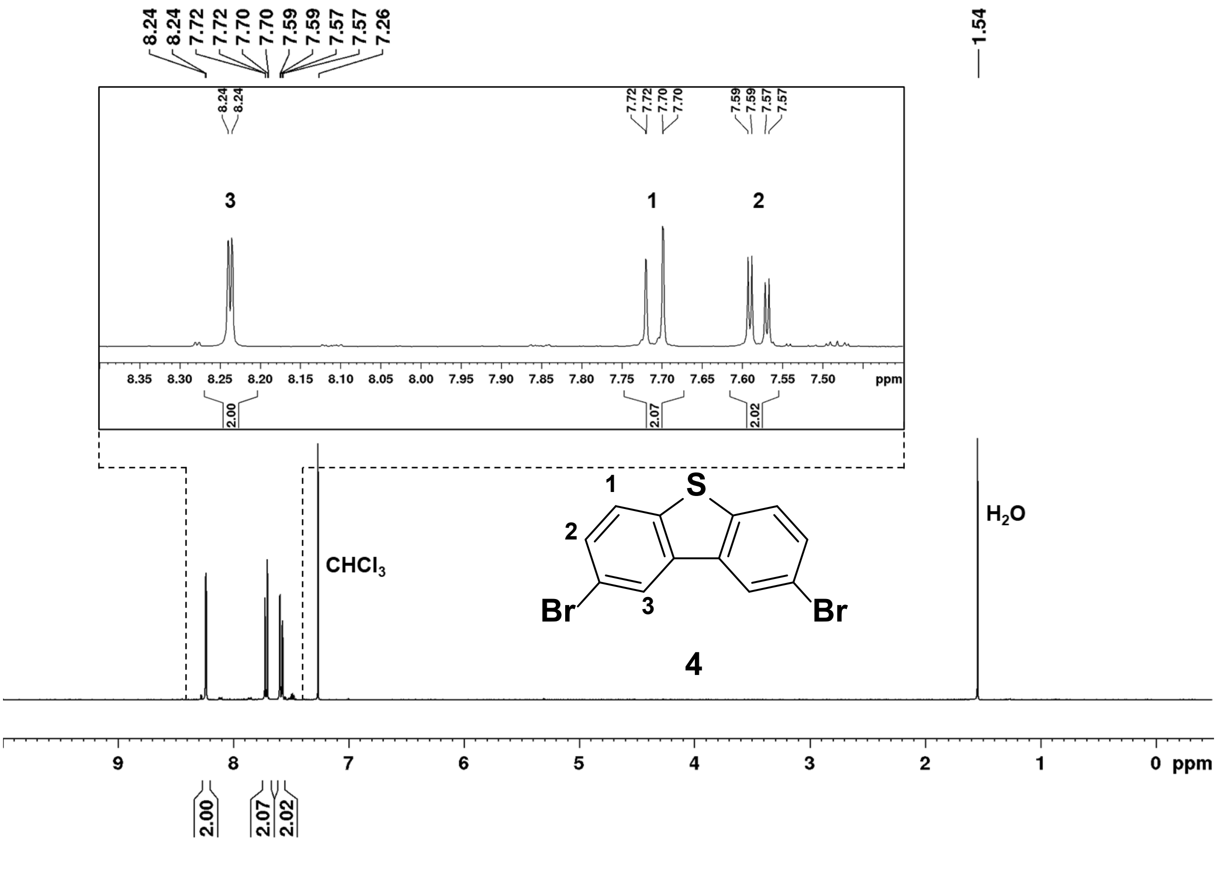

Figure S 74. ^{1}H NMR spectrum of **4** ($CDCl_3$, 400 MHz, rt.).

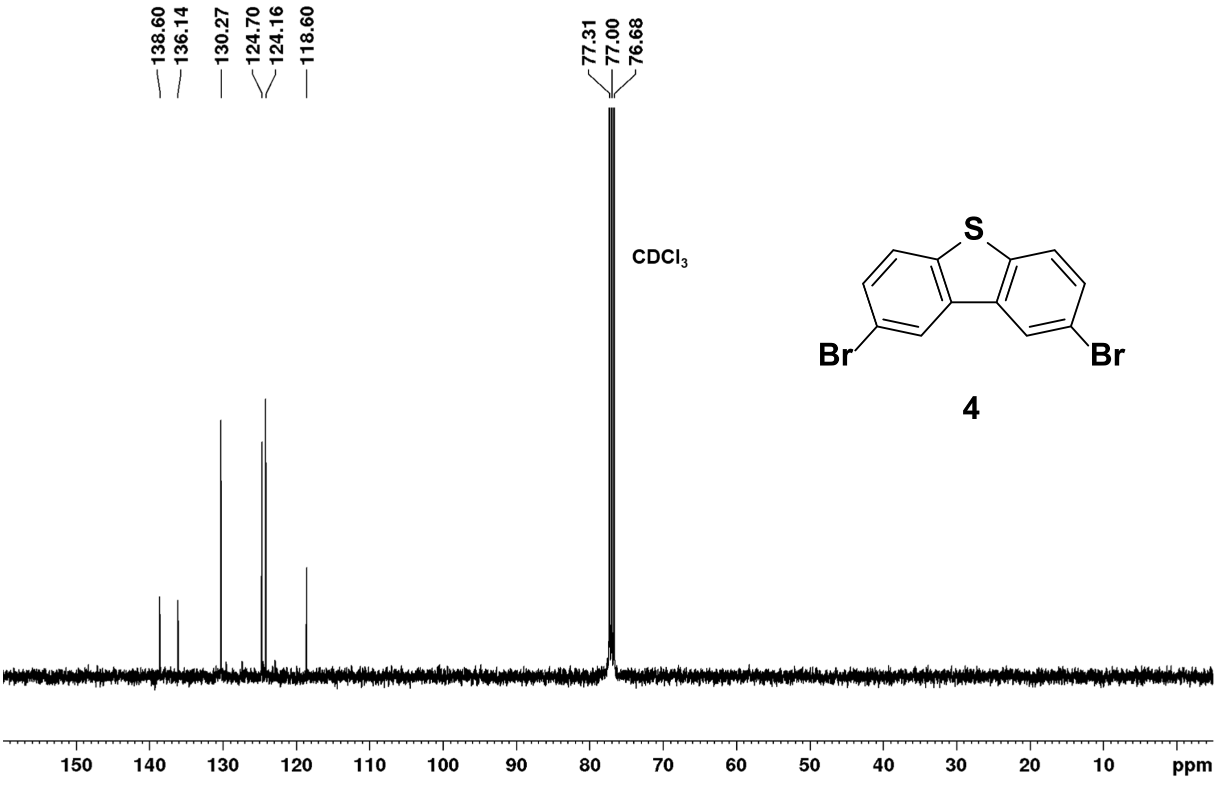

Figure S 75. ^{13}C NMR spectrum of **4** ($CDCl_3$, 100 MHz, rt.).

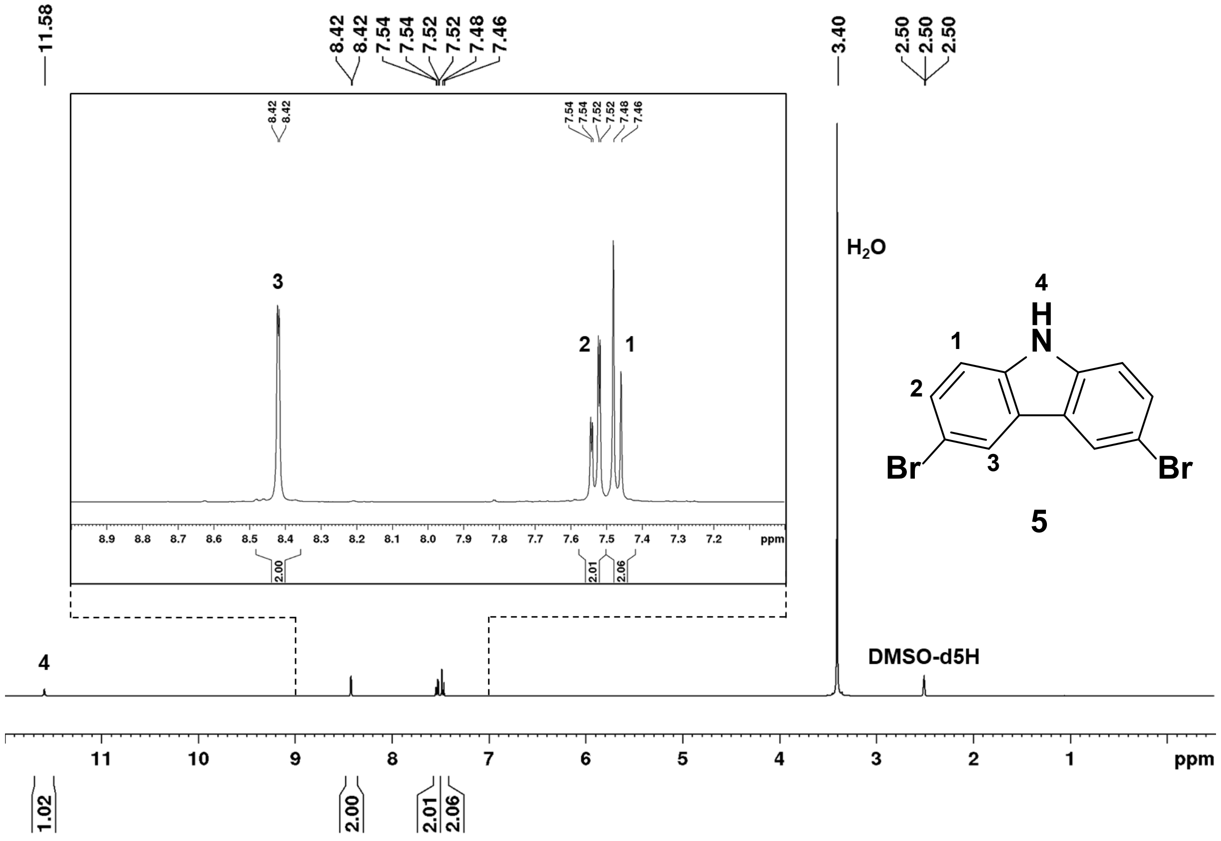

Figure S 76. ^{1}H NMR spectrum of **5** ($CDCl_3$, 400 MHz, rt.).

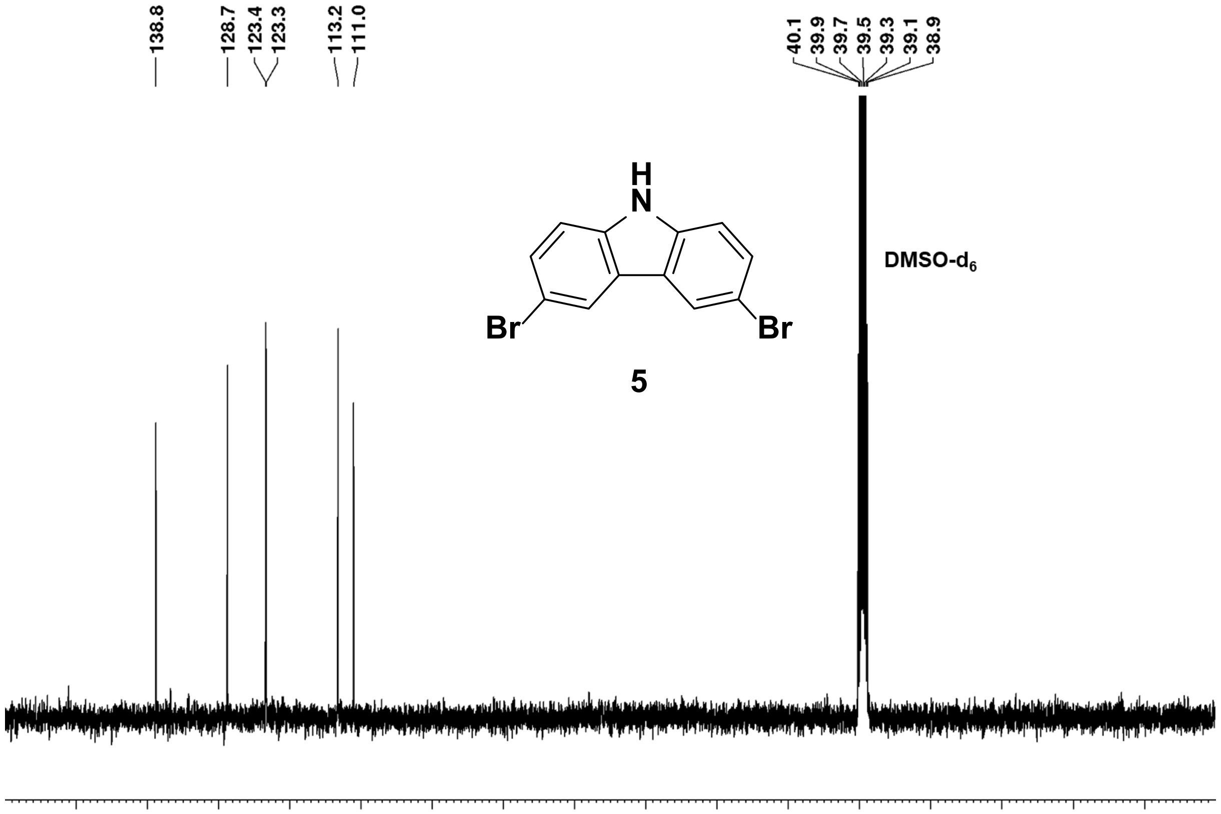

Figure S 77. ^{13}C NMR spectrum of **5** ($CDCl_3$, 100 MHz, rt.).

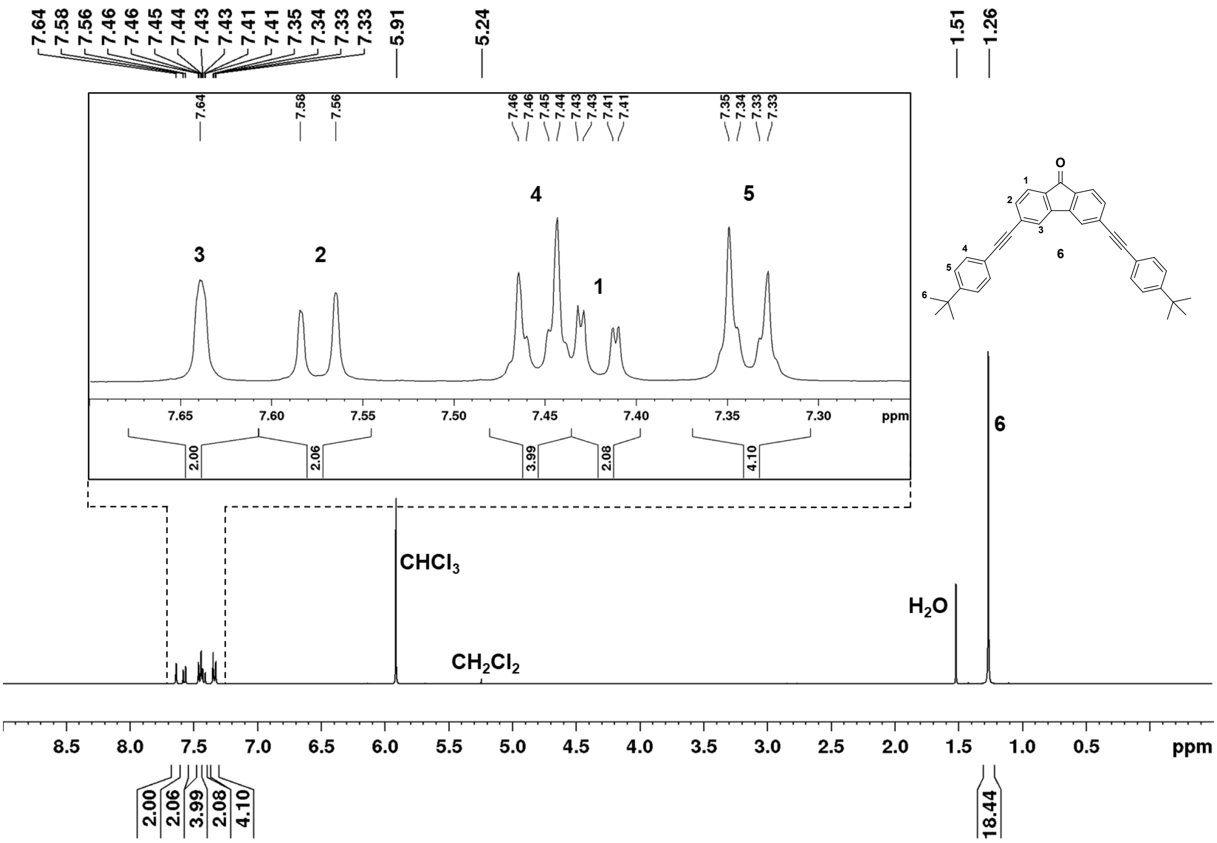

Figure S 78. ^{1}H NMR spectrum of **6** ($C_2D_2Cl_4$, 400 MHz, rt.).

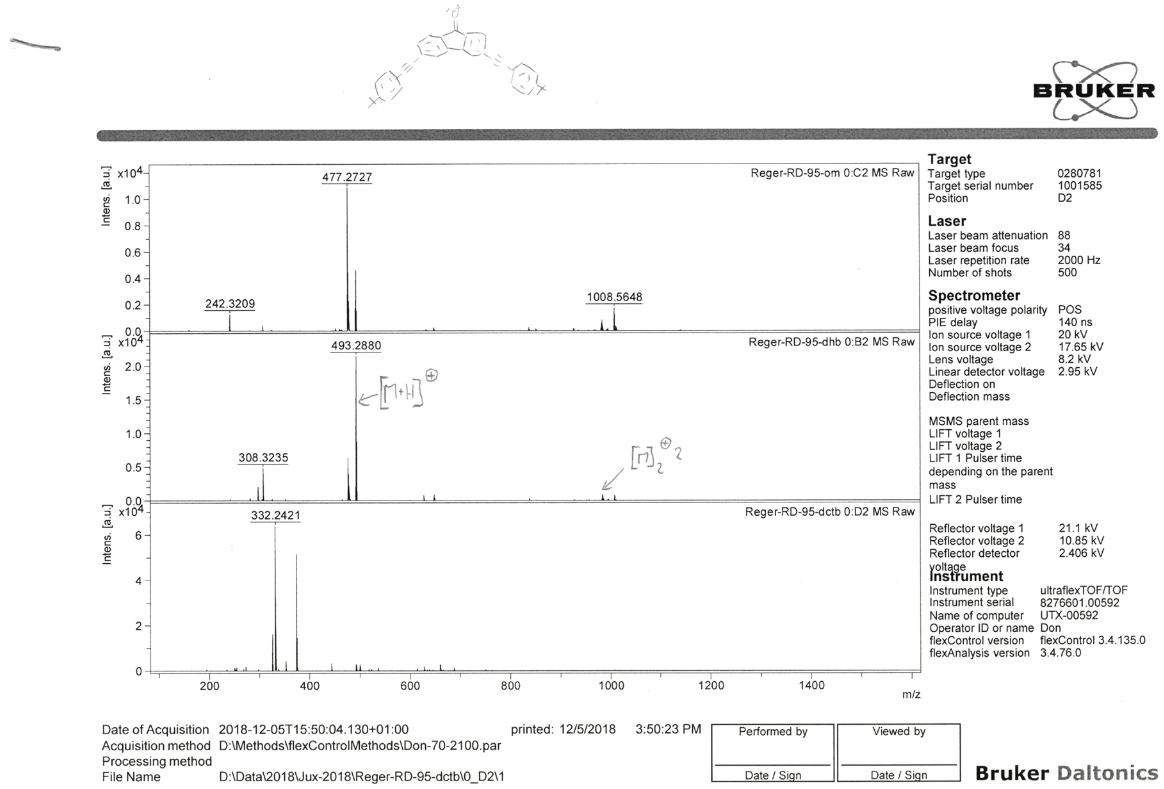

Figure S 79. MS-data (MALDI) of 6 (top: without matrix; middle: dhb; bottom: dctb).

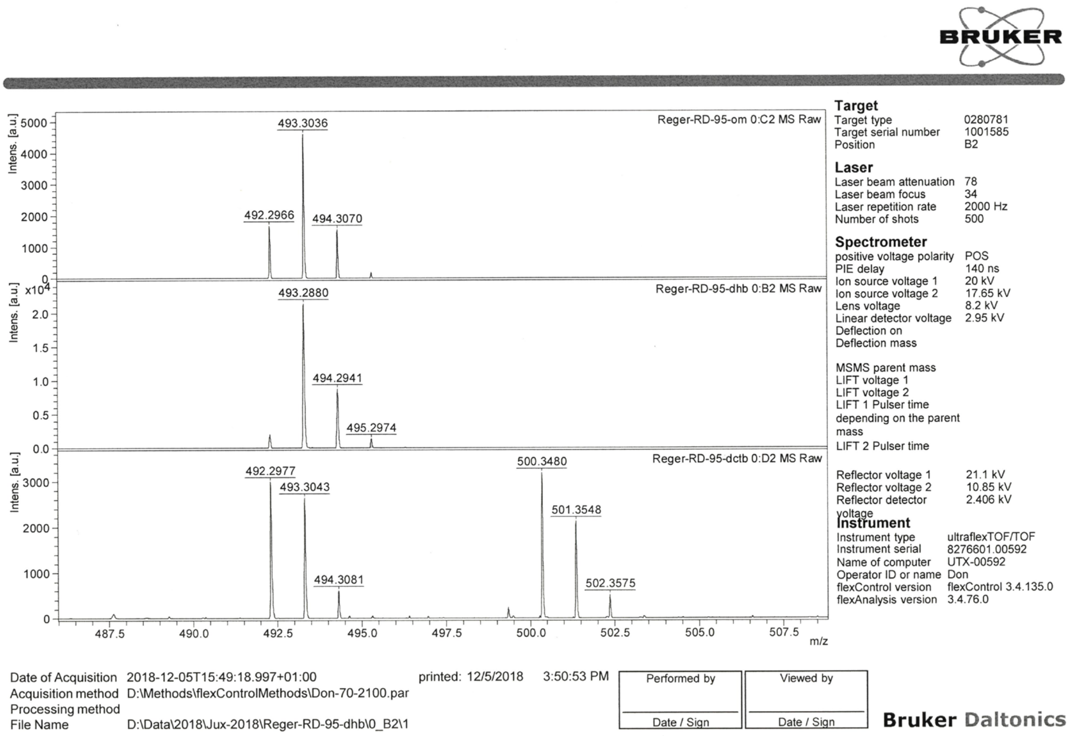

Figure S 80. MS-data (MALDI) of **6** zoom on product peak (top: without matrix; middle: dhb; bottom: dctb).

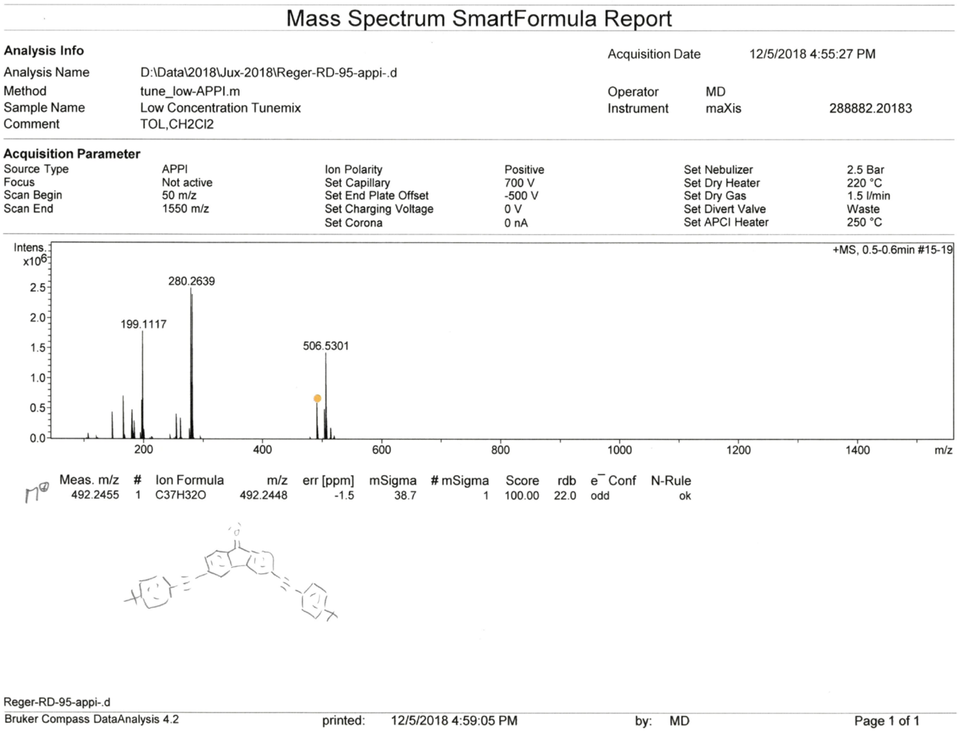

Figure S 81. HRMS data (APPI; toluene/CH_2Cl_2) of **6** (overview).

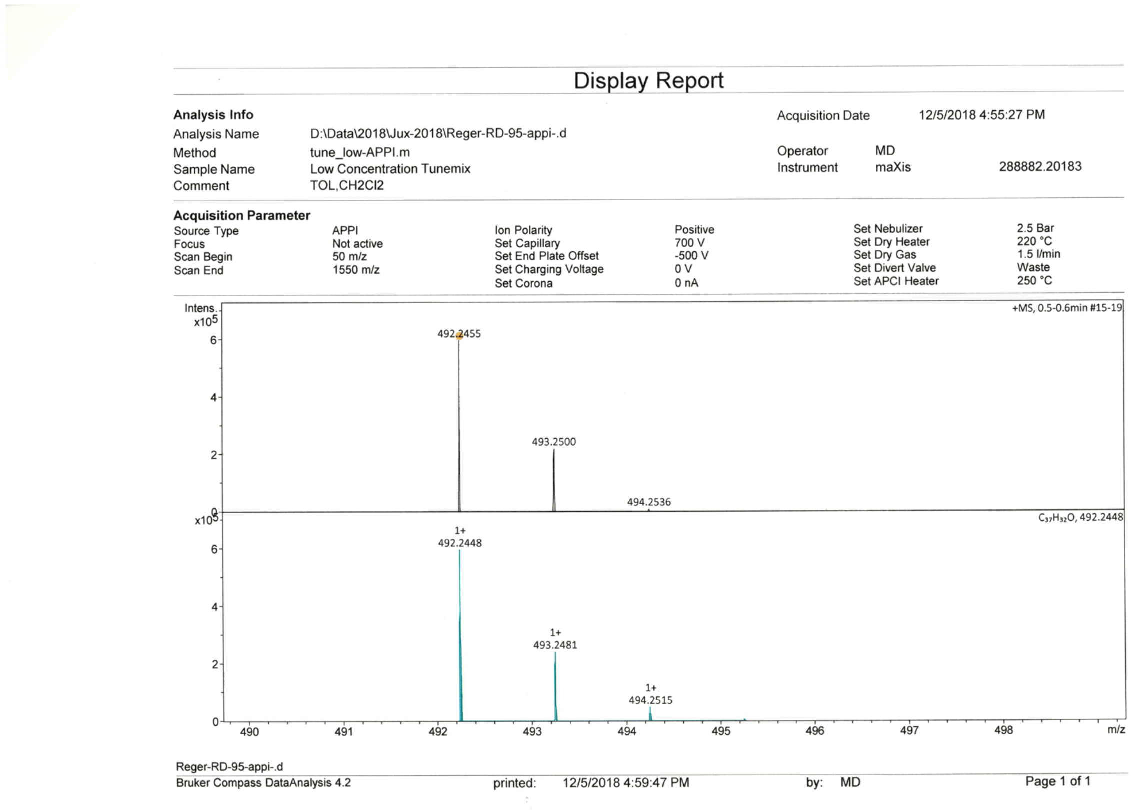

Figure S 82. HRMS data (APPI; toluene/CH_2Cl_2) of **6** (top: measured; bottom: calculated).

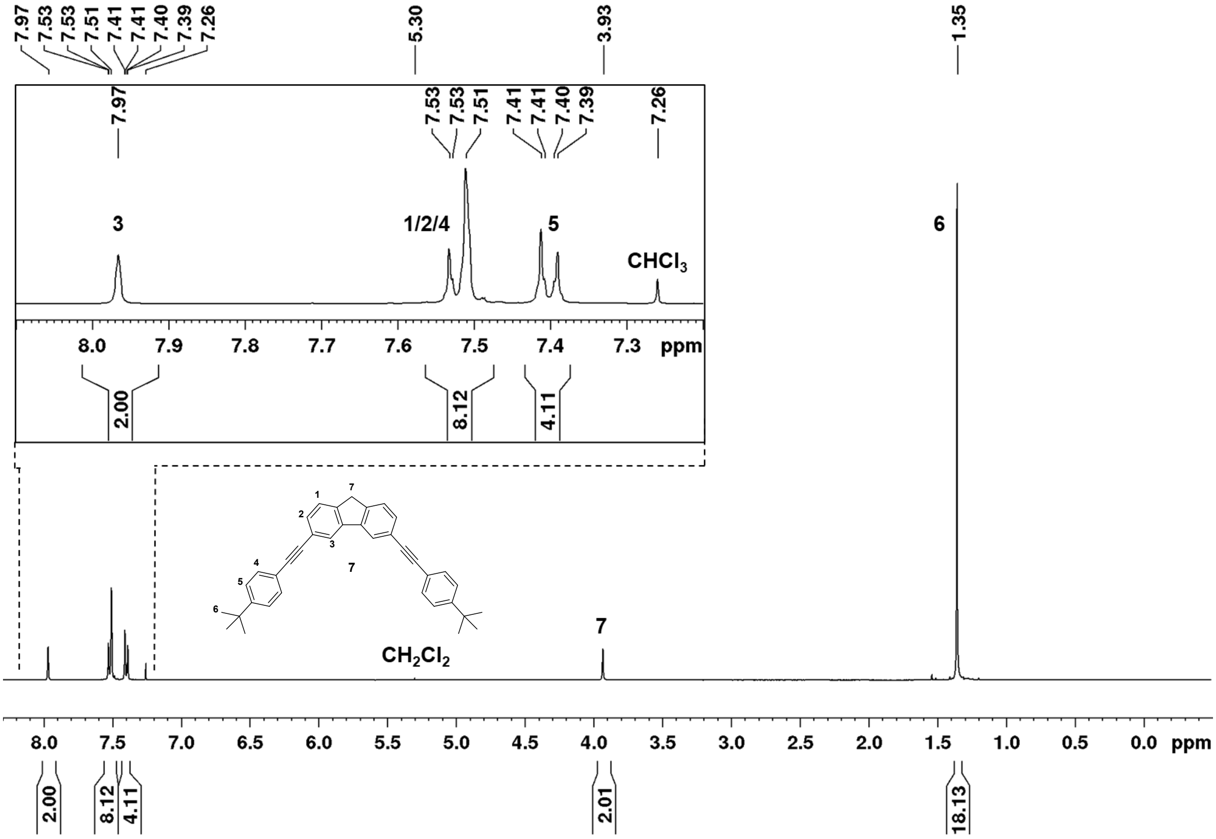

Figure S 83. ^{1}H NMR spectrum of **7** ($CDCl_3$, 400 MHz, rt.).

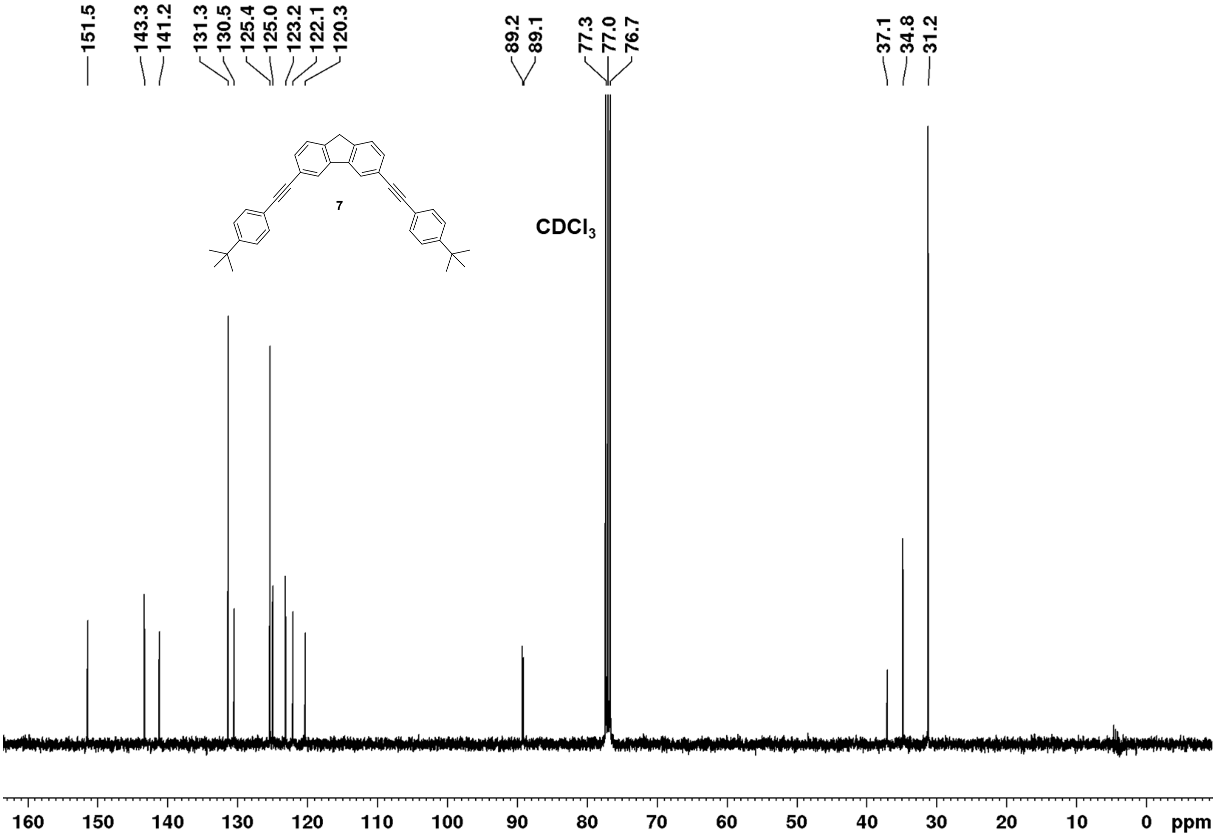

Figure S 84. ^{13}C NMR spectrum of **7** ($CDCl_3$, 100 MHz, rt.).

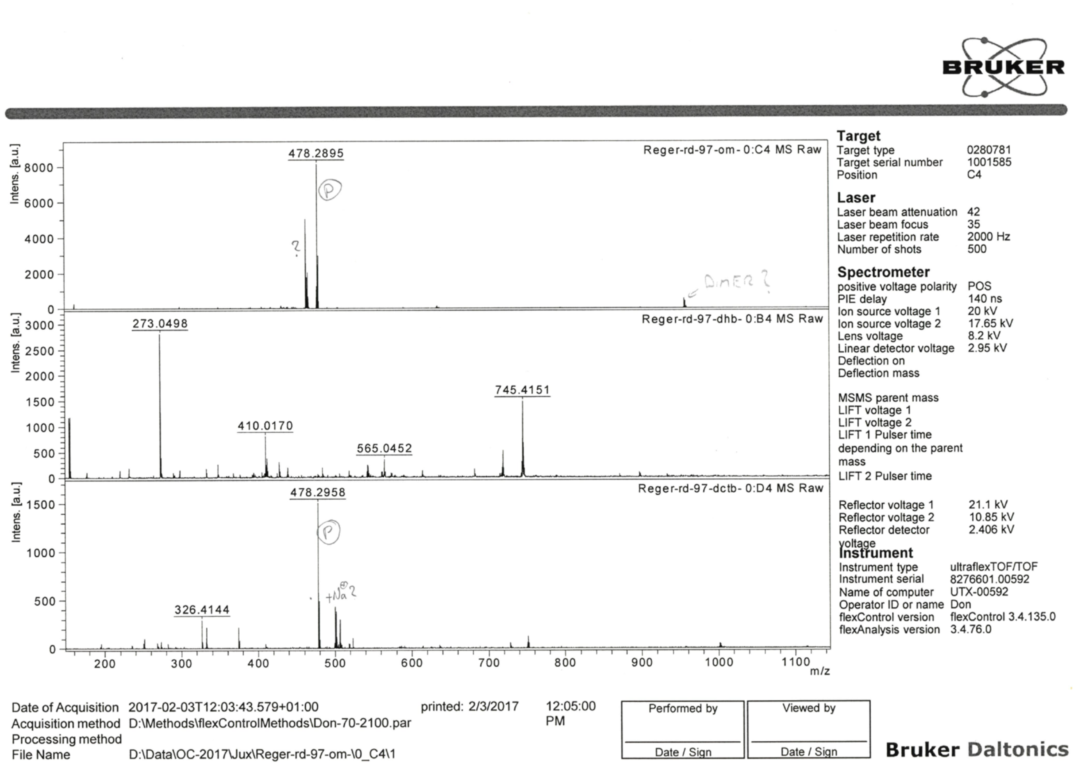

Figure S 85. MS-data (MALDI) of **7** (top: without matrix; middle: dhb; bottom: dctb).

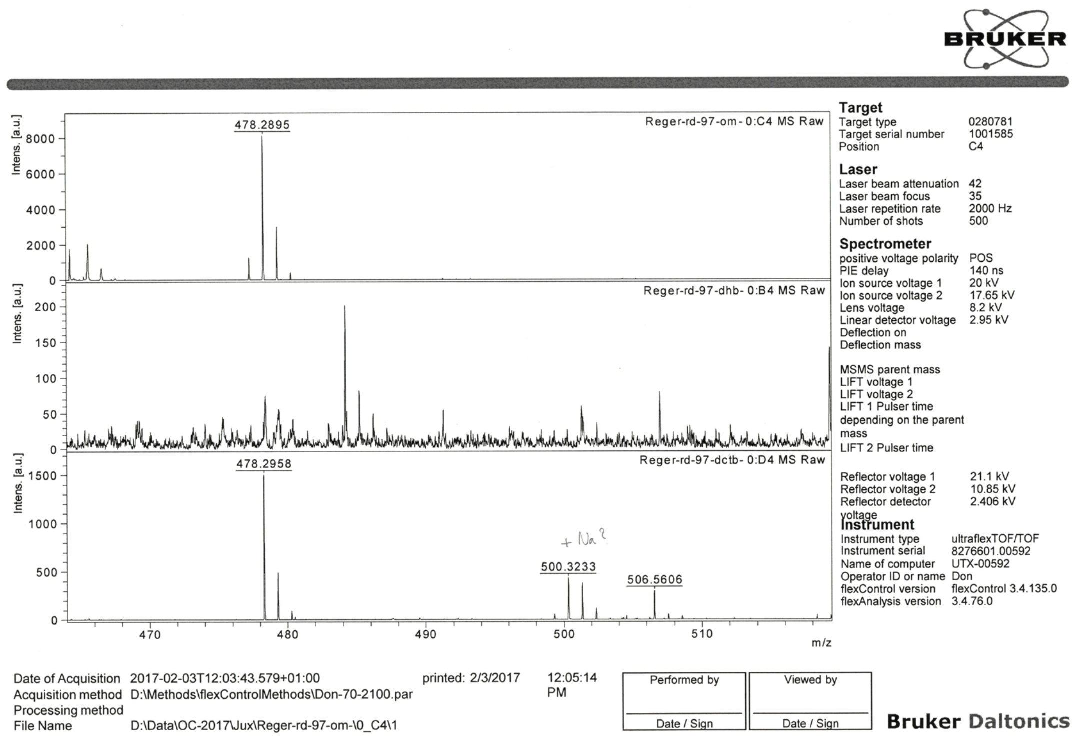

Figure S 86. MS-data (MALDI) of **7** zoom on product peak (top: without matrix; middle: dhb; bottom: dctb).

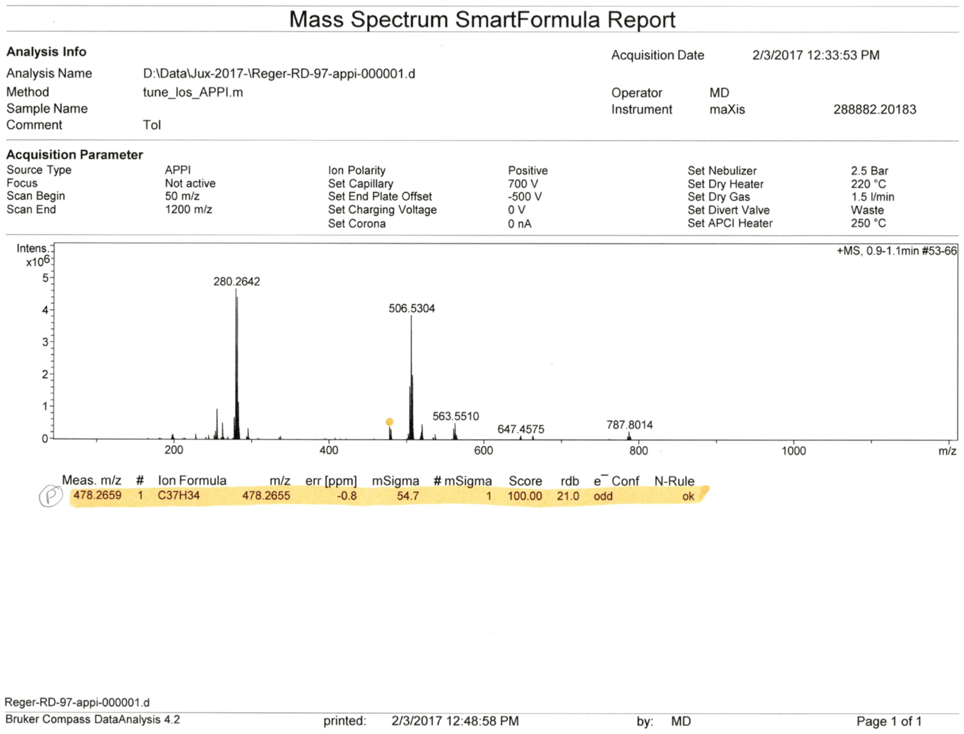

Figure S 87. HRMS data (APPI; toluene) of **7** (overview).

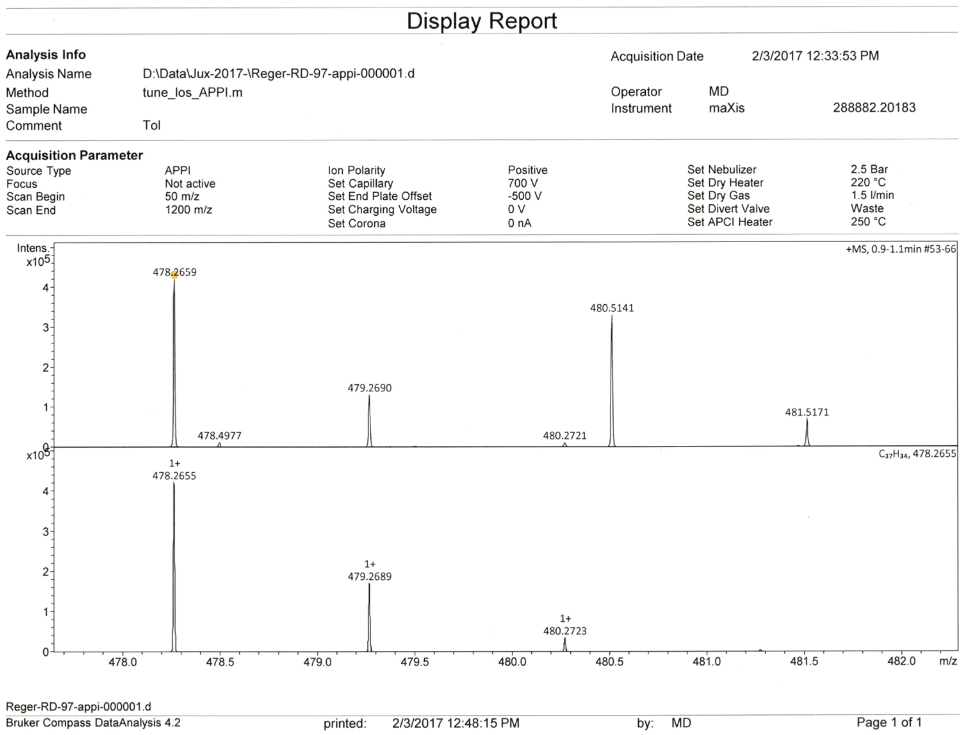

Figure S 88. HRMS data (APPI; toluene) of **7** (top: measured; bottom: calculated).

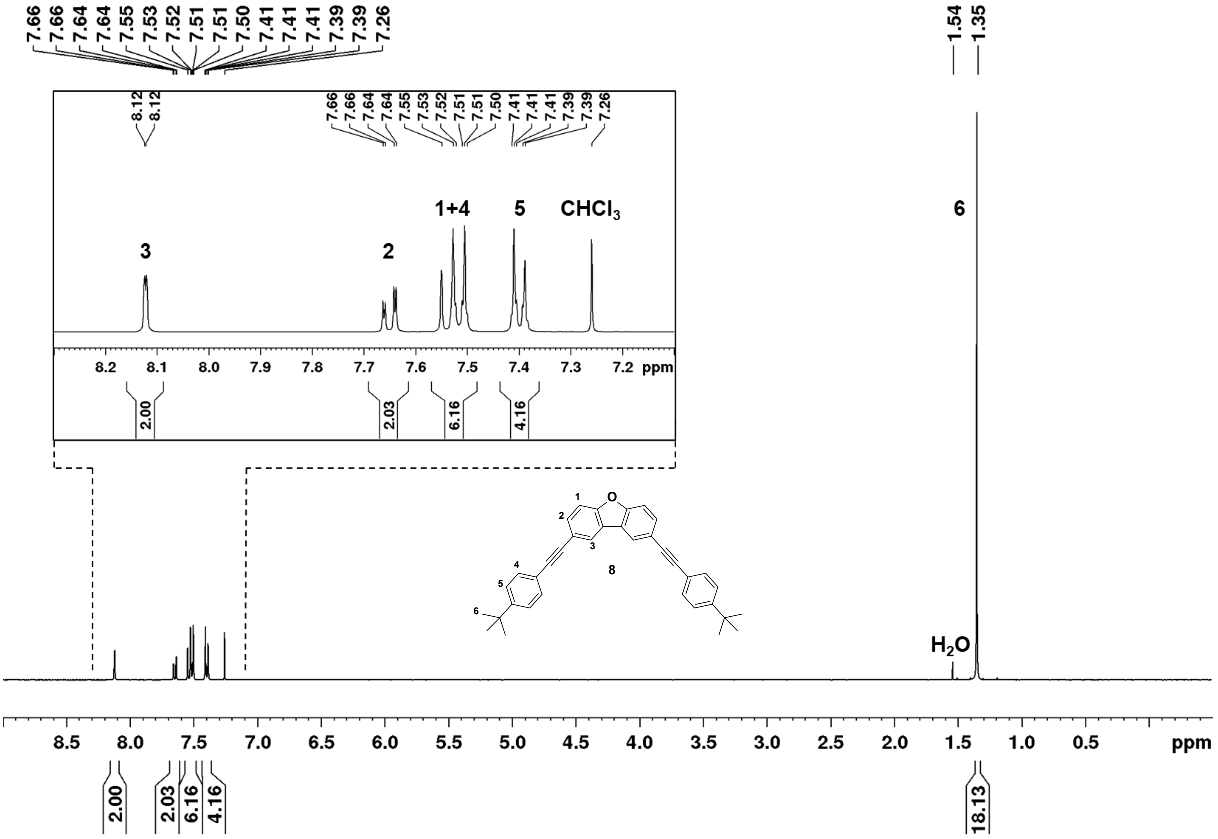

Figure S 89. ^{1}H NMR spectrum of **8** ($CDCl_3$, 400 MHz, rt.).

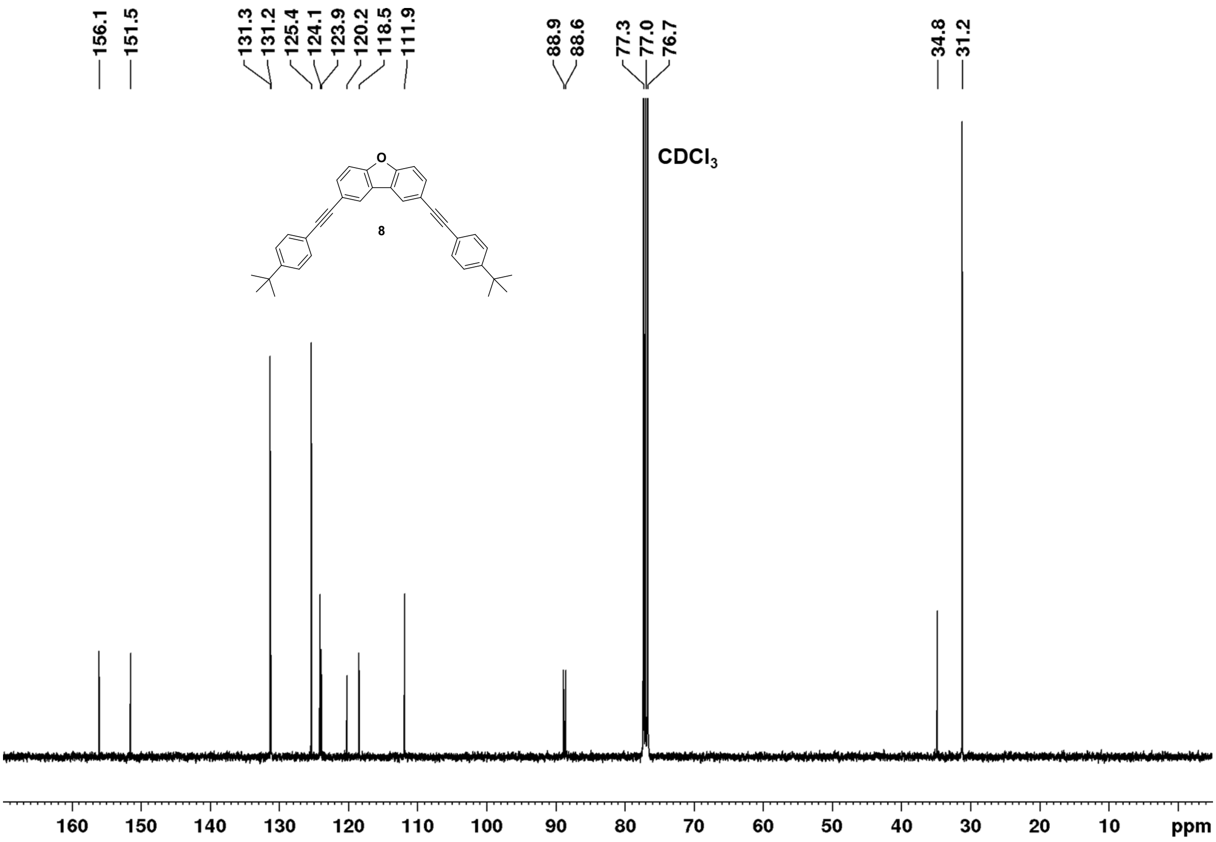

Figure S 90. ^{13}C NMR spectrum of **8** ($CDCl_3$, 100 MHz, rt.).

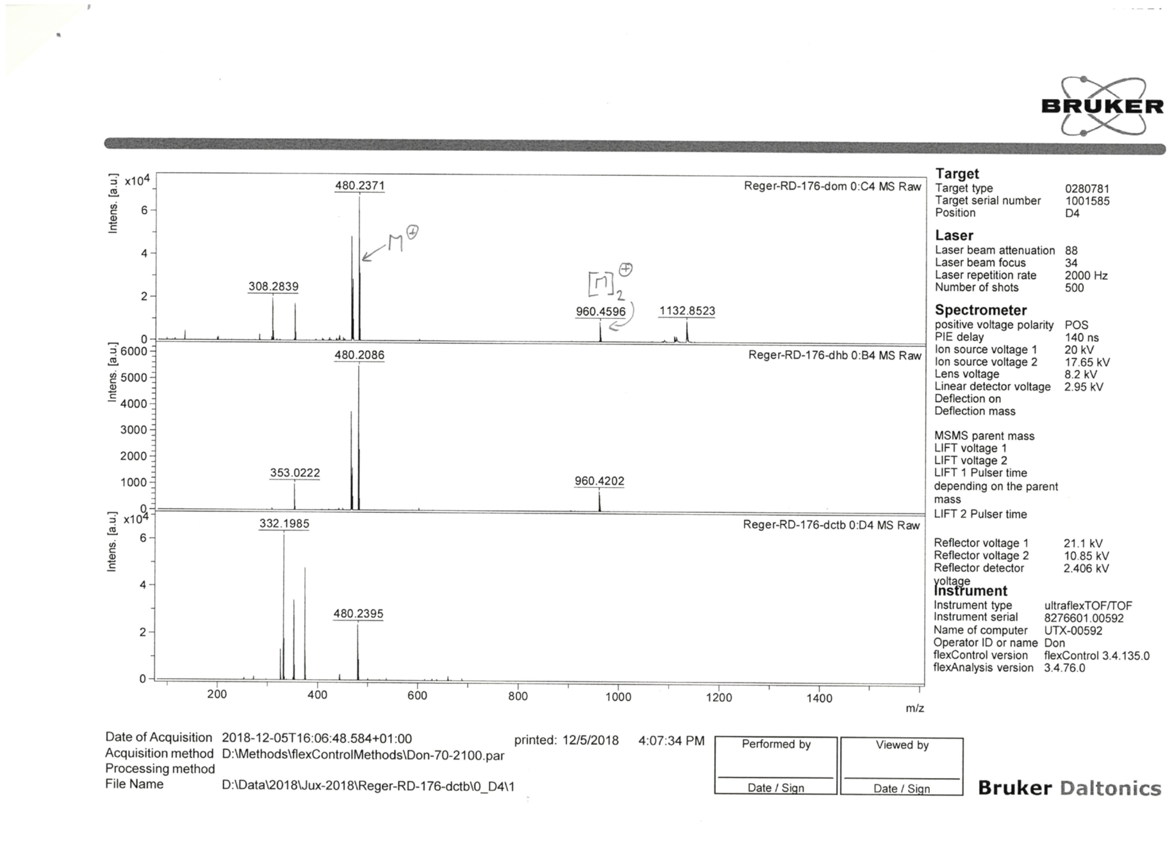

Figure S 91. MS-data (MALDI) of **8** (top: without matrix; middle: dhb; bottom: dctb).

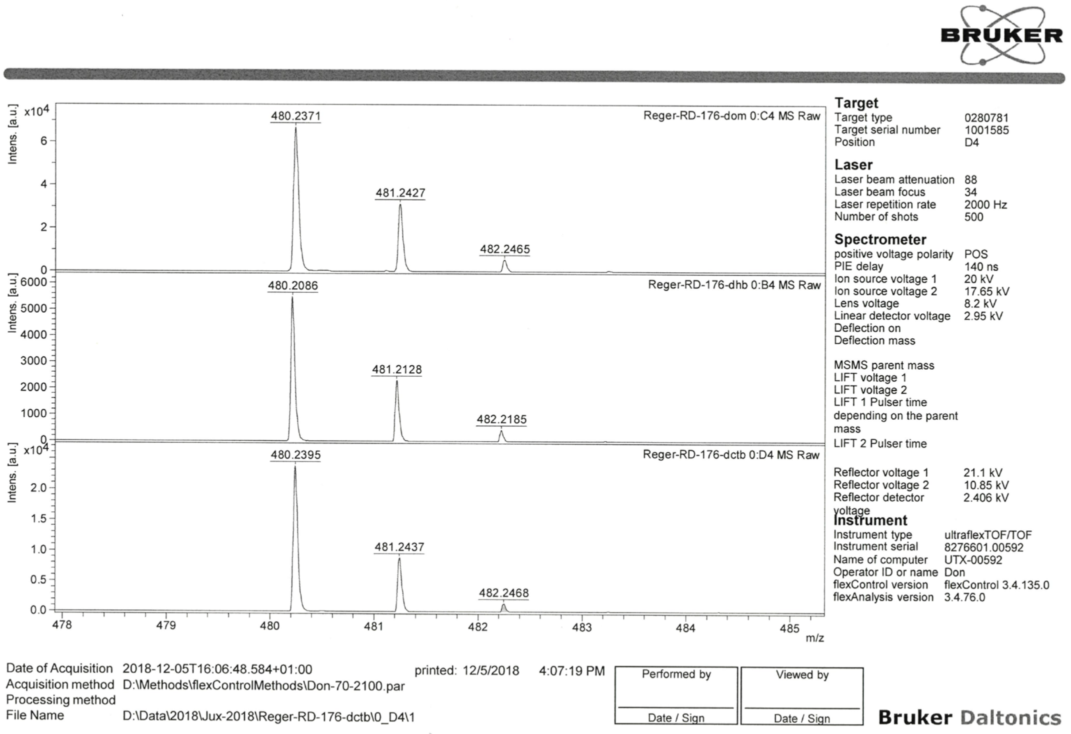

Figure S 92. MS-data (MALDI) of **8** zoom on product peak (top: without matrix; middle: dhb; bottom: dctb).

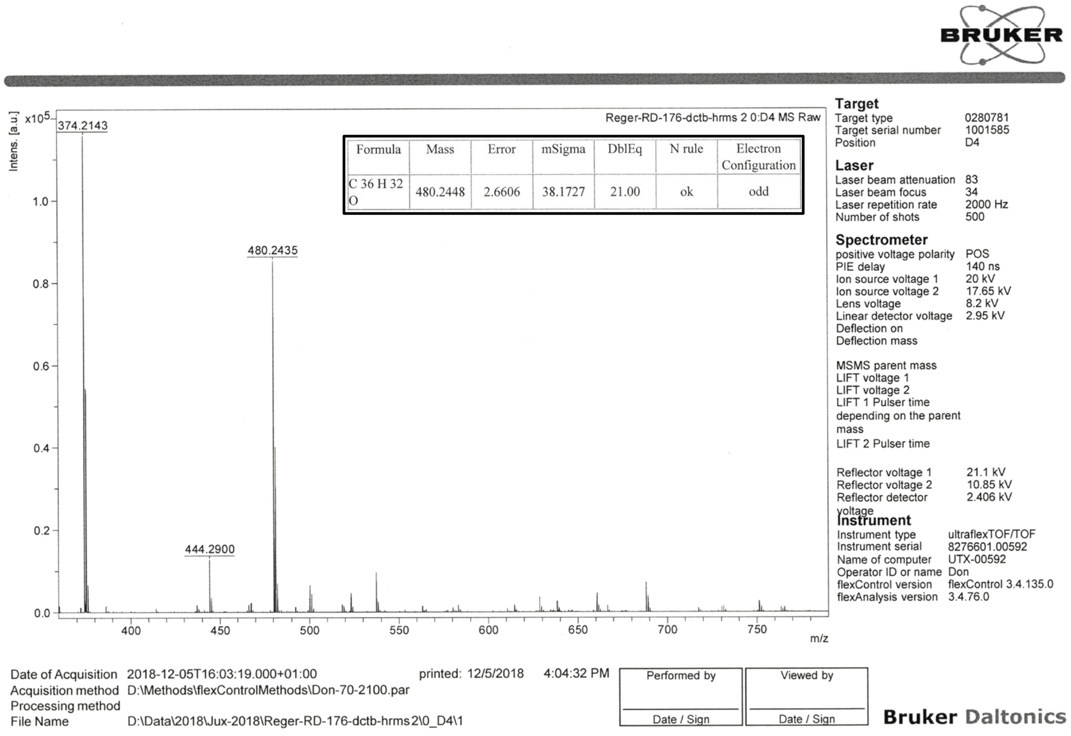

Figure S 93. HRMS data (MALDI; dctb) of **8** (overview). Insert: Calculated mass.

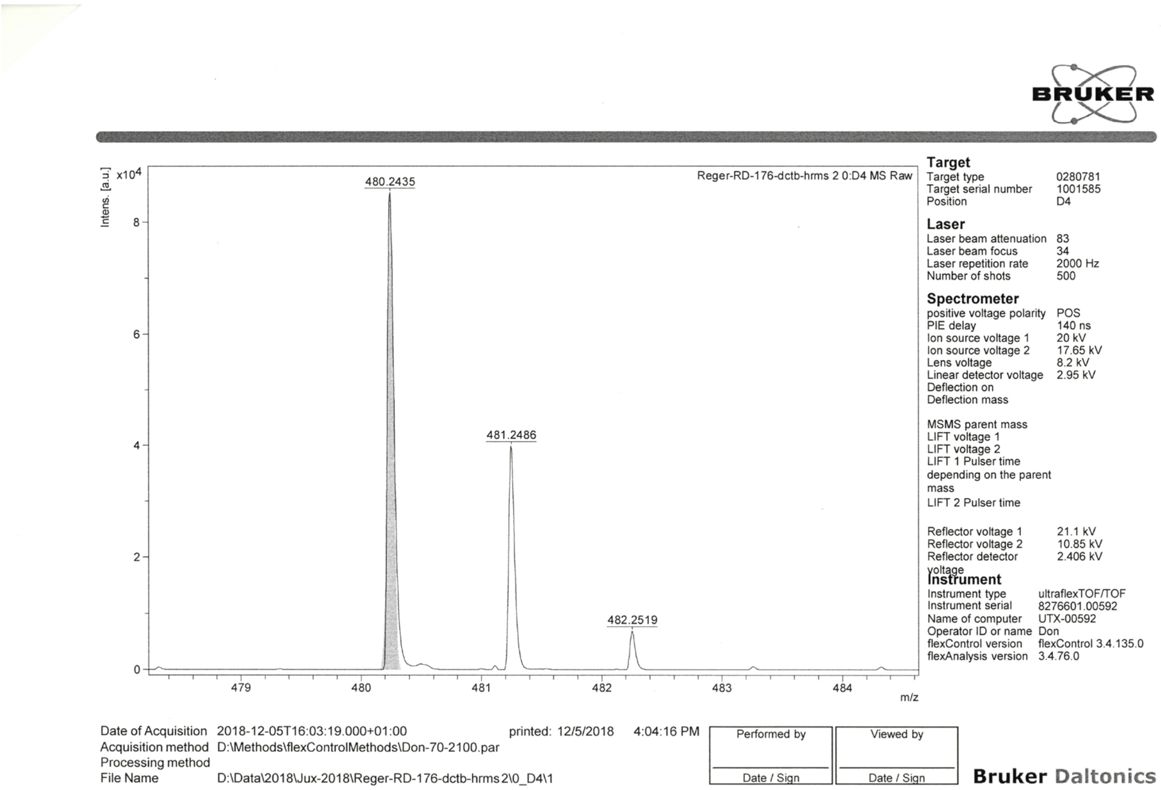

Figure S 94. HRMS data (MALDI; dctb) of **8** (grey background: simulated).

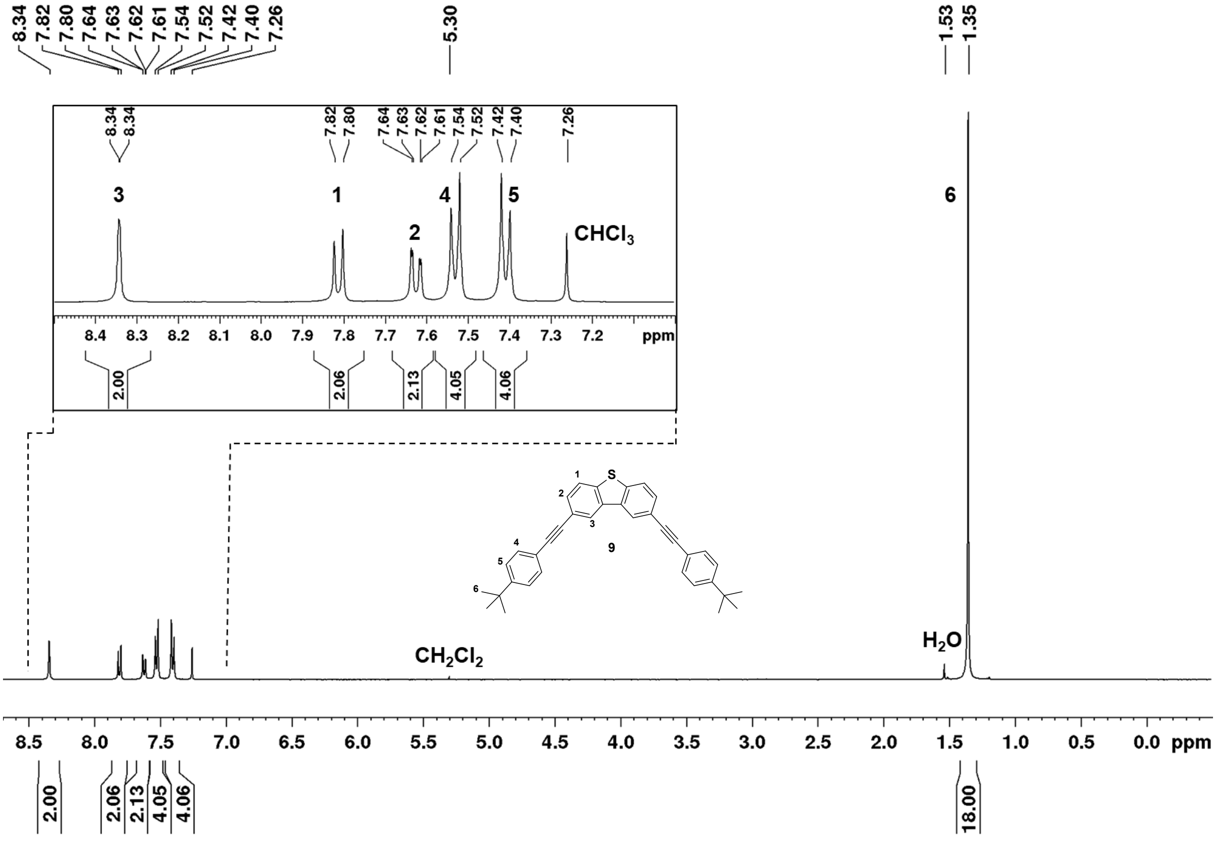

Figure S 95. ^{1}H NMR spectrum of **9** ($CDCl_3$, 400 MHz, rt.).

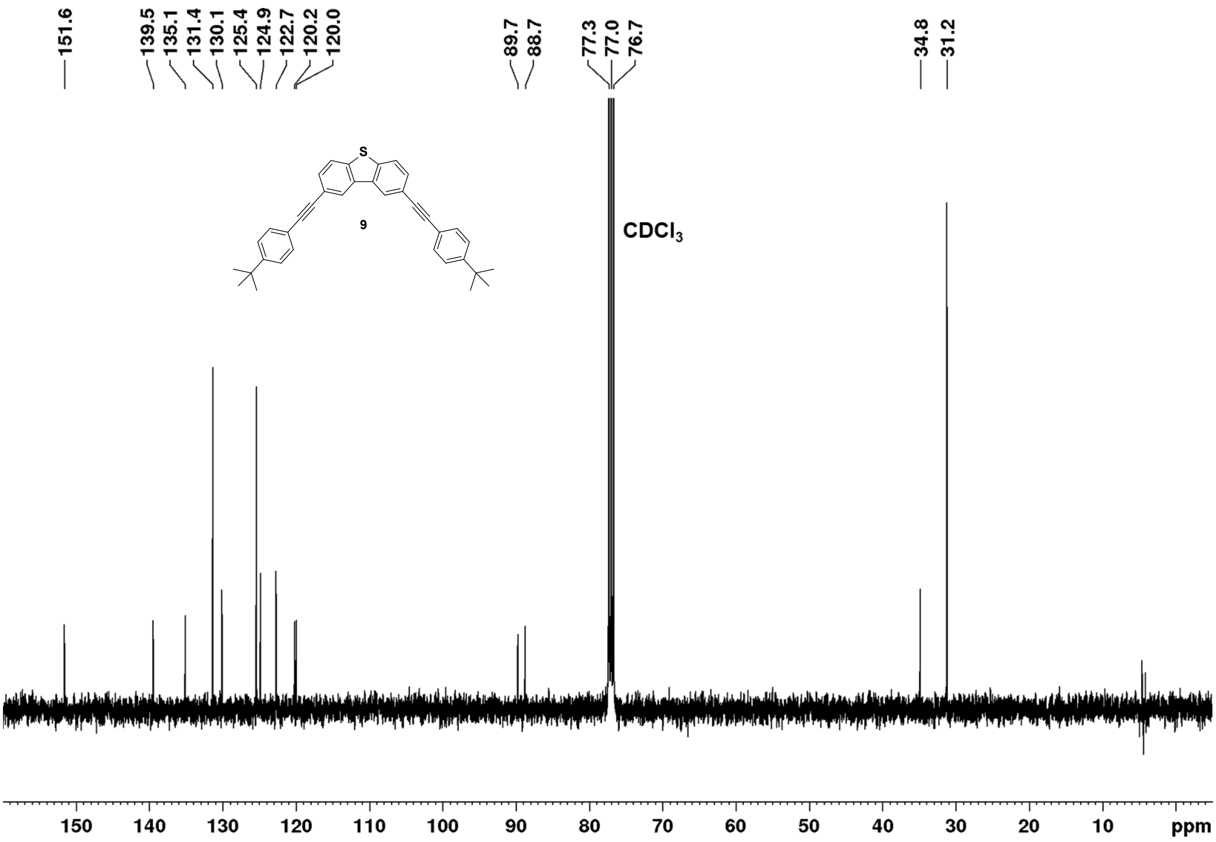

Figure S 96. ^{13}C NMR spectrum of **9** ($CDCl_3$, 100 MHz, rt.).

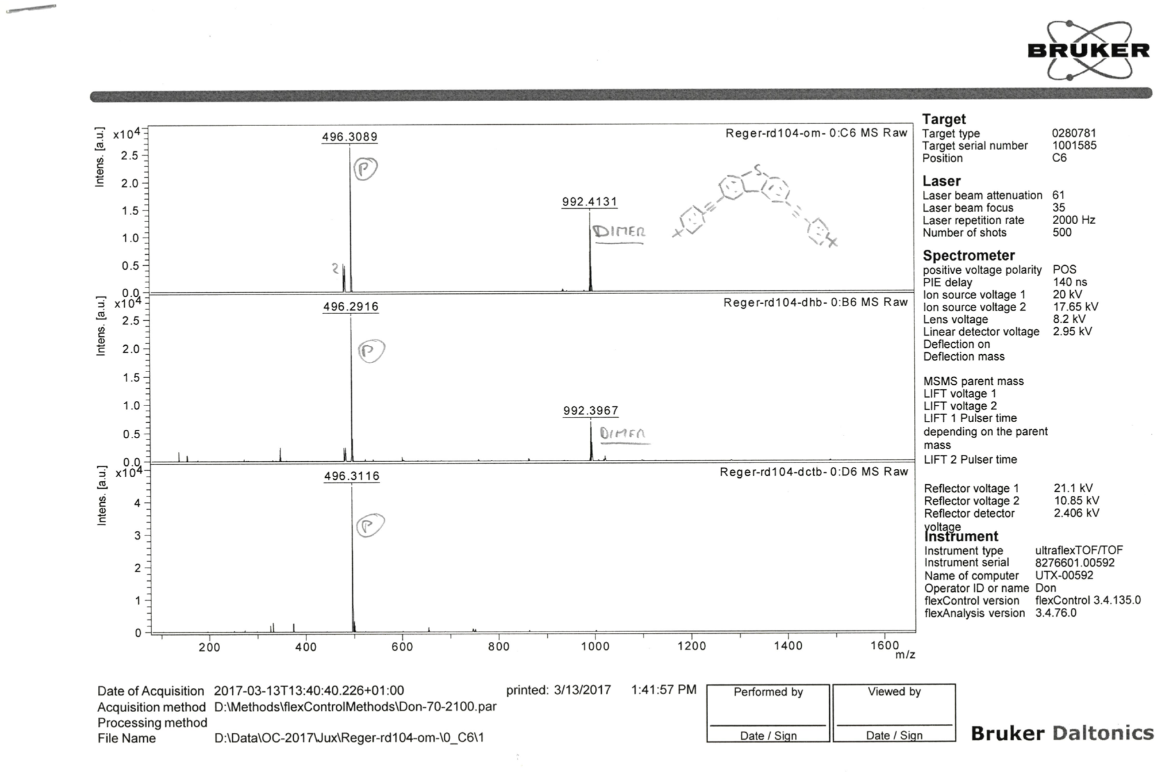

Figure S 97. MS-data (MALDI) of **9** (top: without matrix; middle: dhb; bottom: dctb).

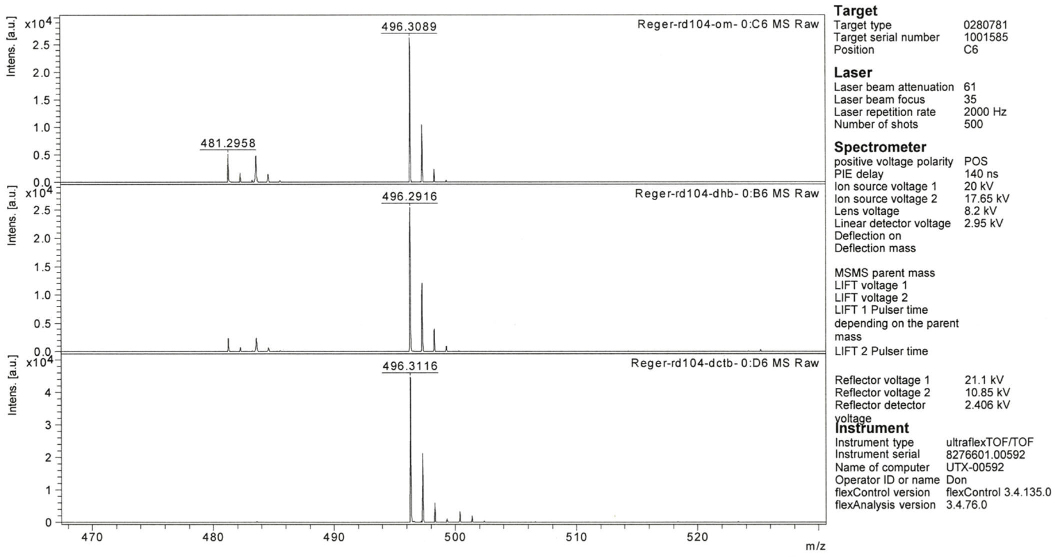

BRUKER

Target
Target type 0280781
Target serial number 1001585
Position C6

Laser
Laser beam attenuation 61
Laser beam focus 35
Laser repetition rate 2000 Hz
Number of shots 500

Spectrometer
positive voltage polarity POS
PIE delay 140 ns
Ion source voltage 1 20 kV
Ion source voltage 2 17.65 kV
Lens voltage 8.2 kV
Linear detector voltage 2.95 kV
Deflection on
Deflection mass

MSMS parent mass
LIFT voltage 1
LIFT voltage 2
LIFT 1 Pulser time depending on the parent mass
LIFT 2 Pulser time

Reflector voltage 1 21.1 kV
Reflector voltage 2 10.85 kV
Reflector detector voltage 2.406 kV

Instrument
Instrument type ultraflexTOF/TOF
Instrument serial 8276601.00592
Name of computer UTX-00592
Operator ID or name Don
flexControl version flexControl 3.4.135.0
flexAnalysis version 3.4.76.0

Date of Acquisition 2017-03-13T13:40:40.226+01:00 printed: 3/13/2017 1:42:09 PM
Acquisition method D:\Methods\flexControlMethods\Don-70-2100.par
Processing method
File Name D:\Data\OC-2017\Jux\Reger-rd104-om-\0_C6\1

Performed by — Date / Sign
Viewed by — Date / Sign

Bruker Daltonics

Figure S 98. MS-data (MALDI) of **9** zoom on product peak (top: without matrix; middle: dhb; bottom: dctb).

Mass Spectrum SmartFormula Report

Analysis Info

Analysis Name D:\Data\Jux-2017-\reger-RD-104-appi-000001.d
Method tune_los_APPI.m
Sample Name
Comment ACN

Acquisition Date 3/13/2017 1:56:30 PM
Operator MD
Instrument maXis 288882.20183

Acquisition Parameter

Source Type	APPI	Ion Polarity	Positive	Set Nebulizer	2.5 Bar
Focus	Not active	Set Capillary	700 V	Set Dry Heater	220 °C
Scan Begin	50 m/z	Set End Plate Offset	-500 V	Set Dry Gas	1.5 l/min
Scan End	1200 m/z	Set Charging Voltage	0 V	Set Divert Valve	Waste
		Set Corona	0 nA	Set APCI Heater	250 °C

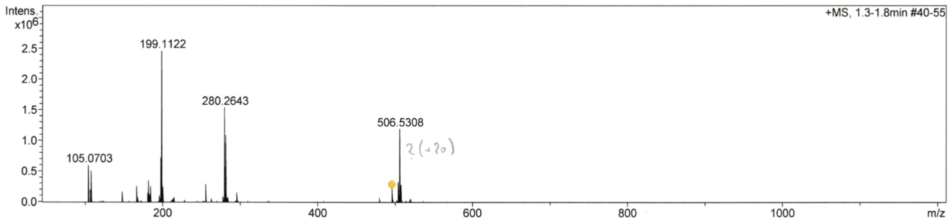

Meas. m/z	#	Ion Formula	m/z	err [ppm]	mSigma	# mSigma	Score	rdb	e⁻ Conf	N-Rule
496.2228	1	C30H30N3O4	496.2231	0.6	65.0	1	100.00	17.5	even	ok
	2	C36H32S	496.2219	-1.8	104.8	2	13.94	21.0	odd	ok

reger-RD-104-appi-000001.d
Bruker Compass DataAnalysis 4.2 printed: 3/13/2017 1:59:51 PM by: MD Page 1 of 1

Figure S 99. HRMS data (APPI; CH_3CN) of **9** (overview).

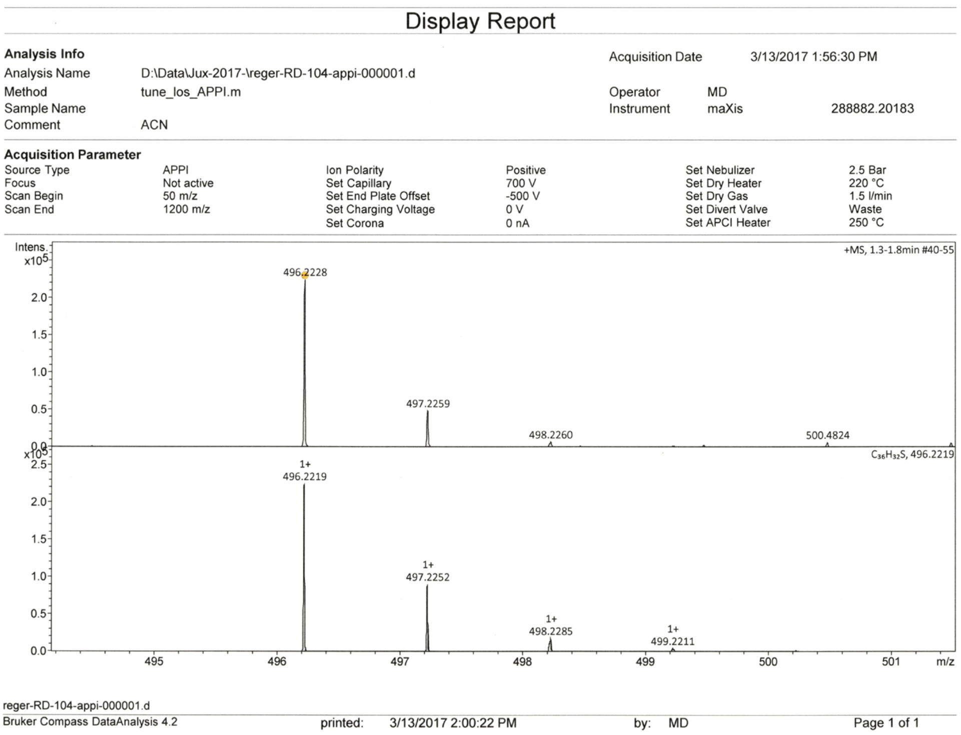

Figure S 100. HRMS data (APPI; CH_3CN) of **9** (top: measured; bottom: calculated).

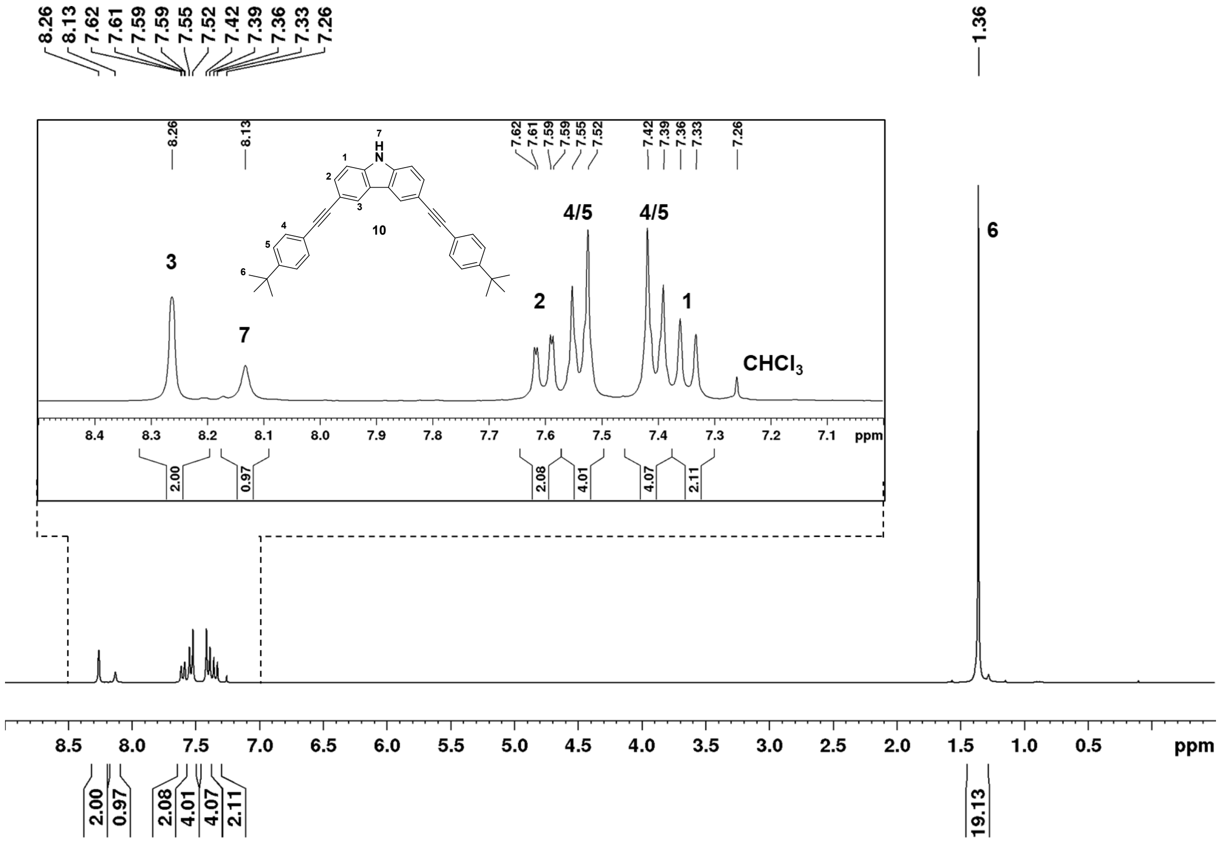

Figure S 101. ^{1}H NMR spectrum of **10** ($CDCl_3$, 300 MHz, rt.).

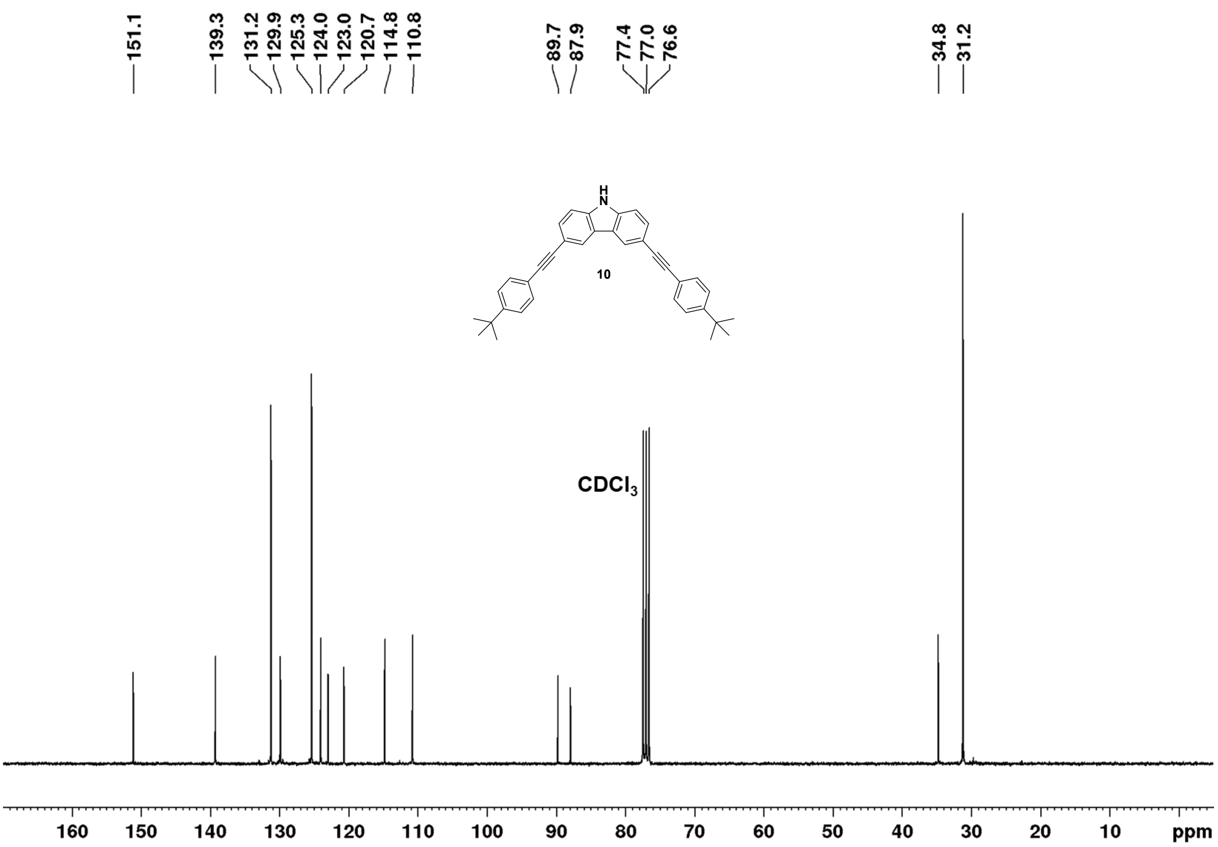

Figure S 102. ^{13}C NMR spectrum of **10** ($CDCl_3$, 75 MHz, rt.).

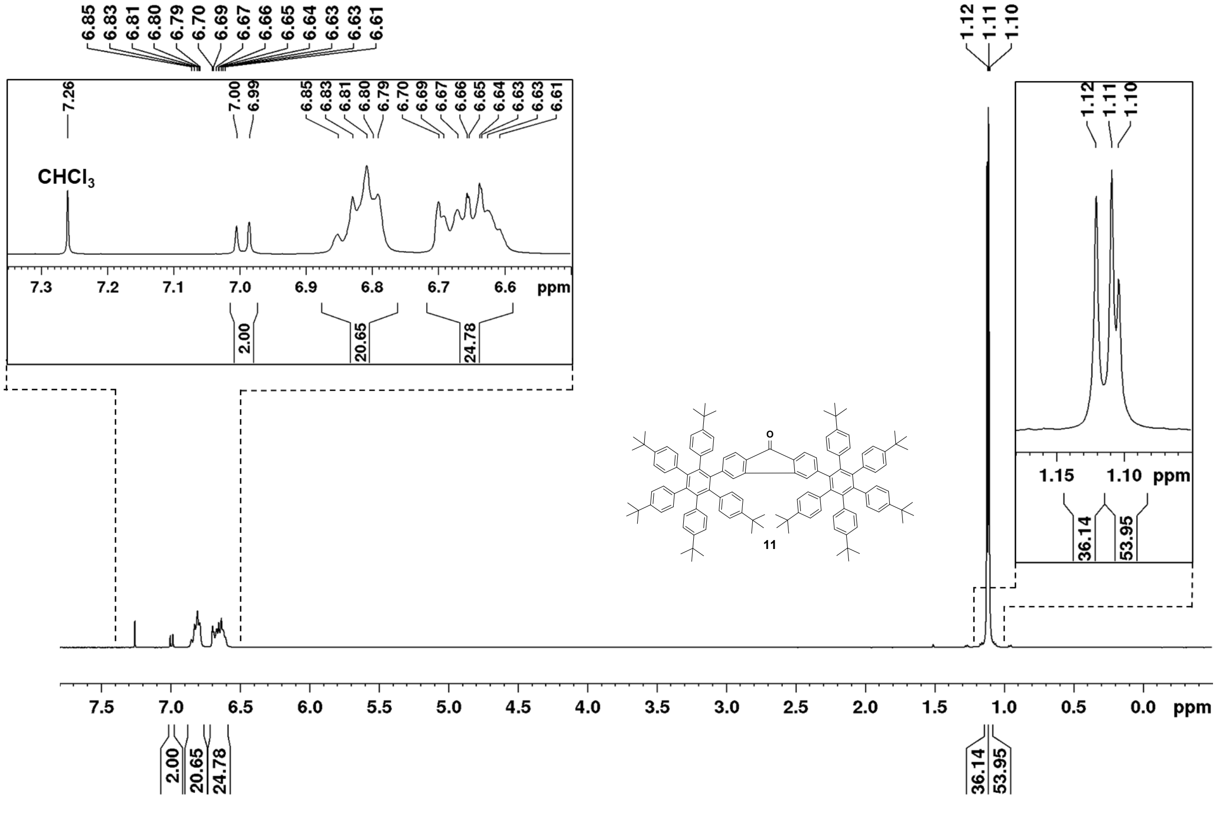

Figure S 103. ^{1}H NMR spectrum of **11** ($CDCl_3$, 400 MHz, rt.).

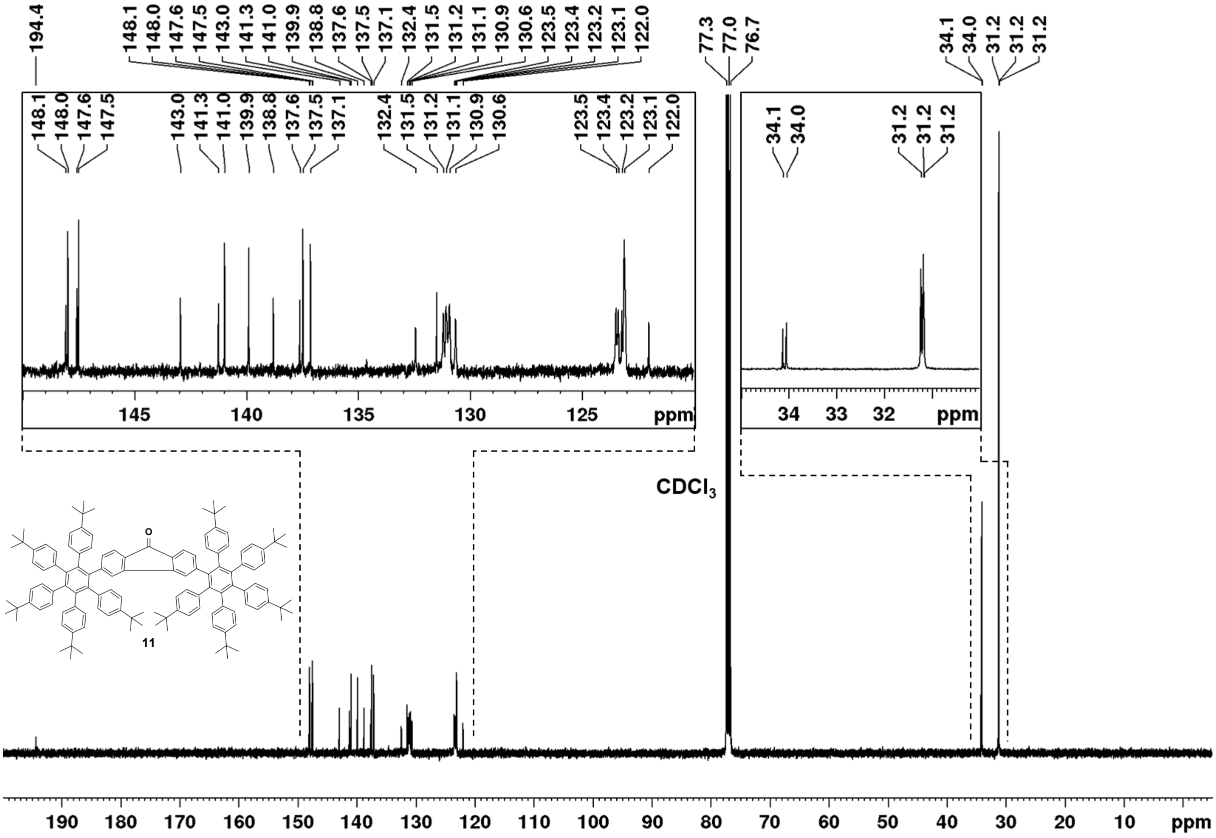

Figure S 104. ^{13}C NMR spectrum of **11** ($CDCl_3$, 100 MHz, rt.).

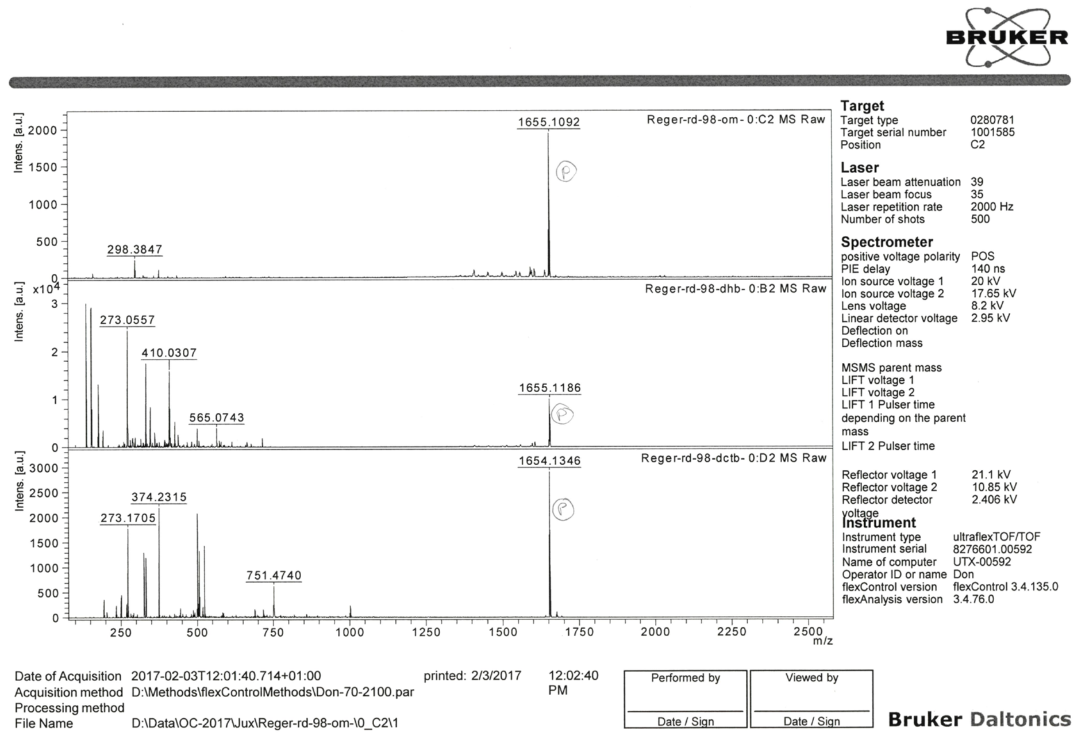

Figure S 105. MS-data (MALDI) of **11** (top: without matrix; middle: dhb; bottom: dctb).

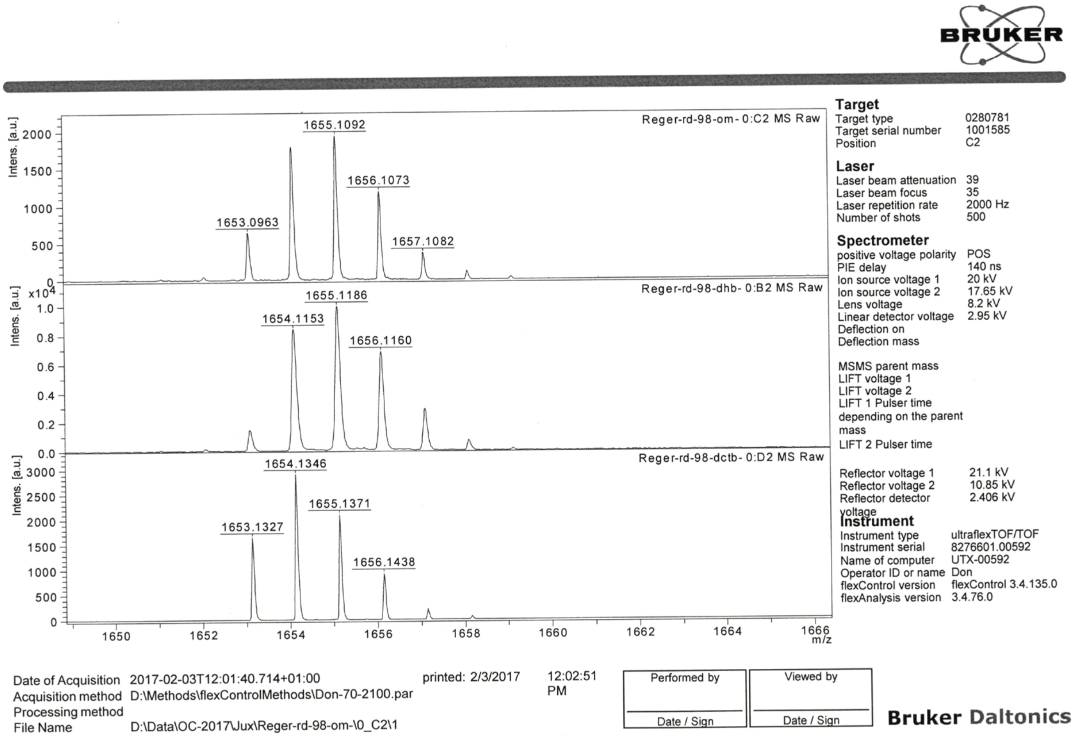

Figure S 106. MS-data (MALDI) of **11** zoom on product peak (top: without matrix; middle: dhb; bottom: dctb).

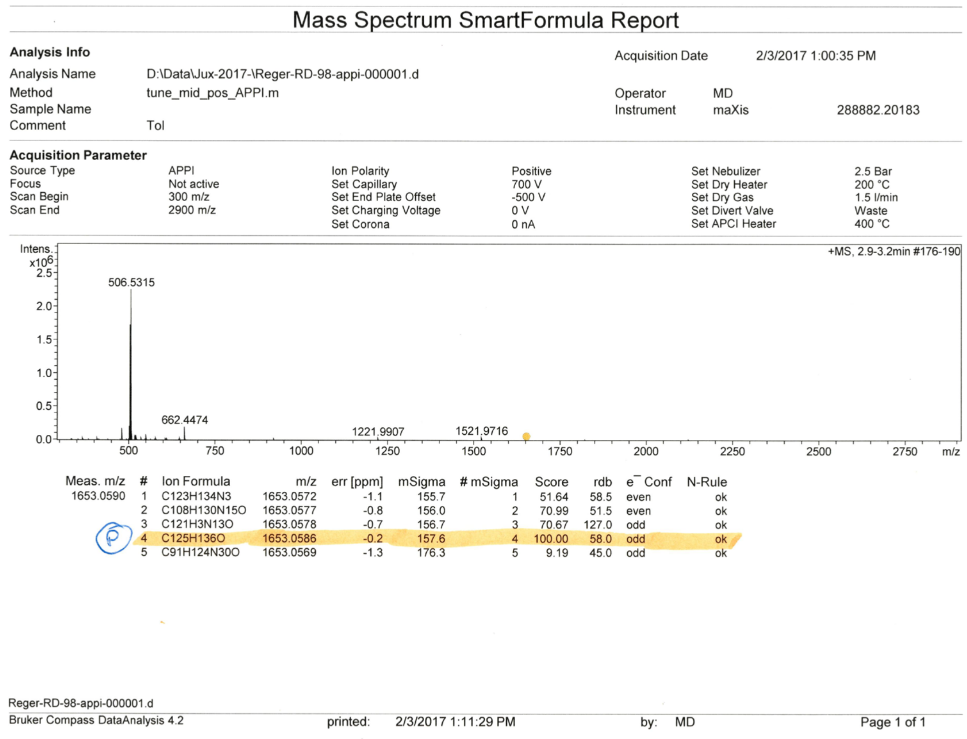

Figure S 107. HRMS data (APPI; toluene) of **11** (overview).

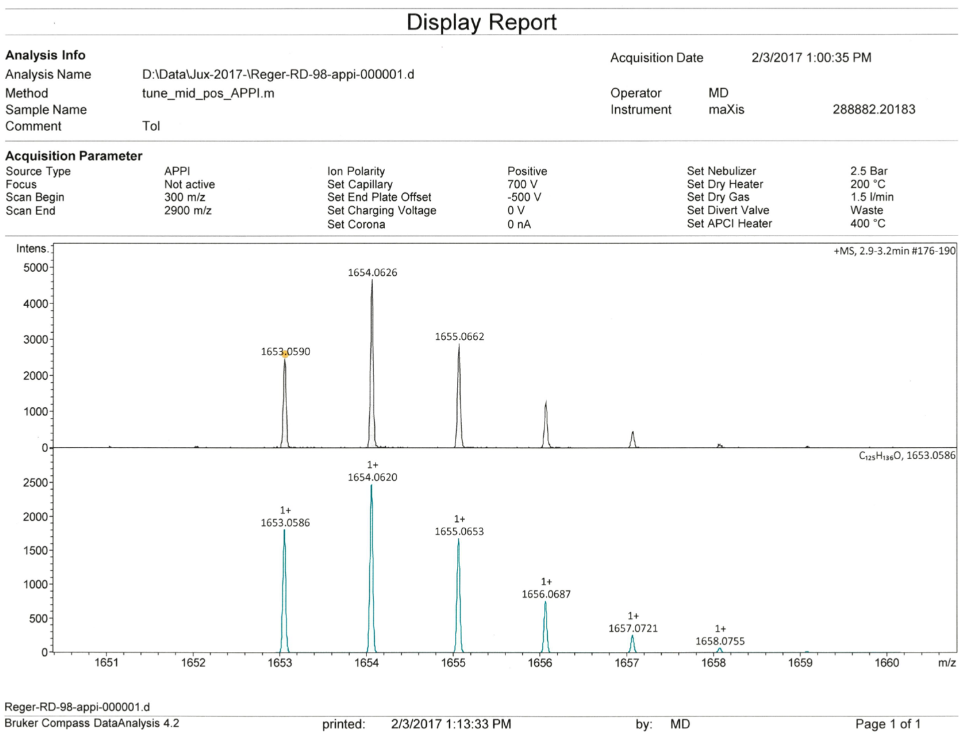

Figure S 108. HRMS data (APPI; toluene) of **11** (top: measured; bottom: calculated).

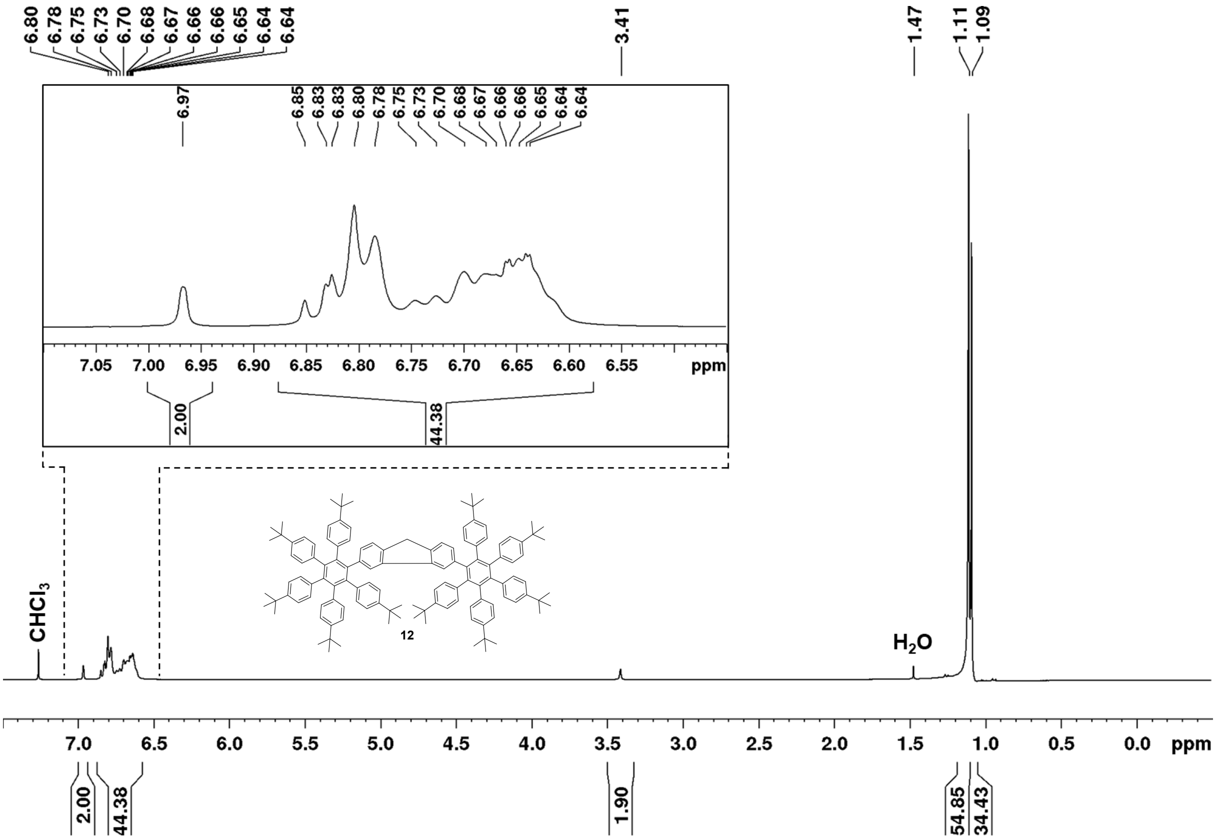

Figure S 109. ^{1}H NMR spectrum of **12** ($CDCl_3$, 400 MHz, rt.).

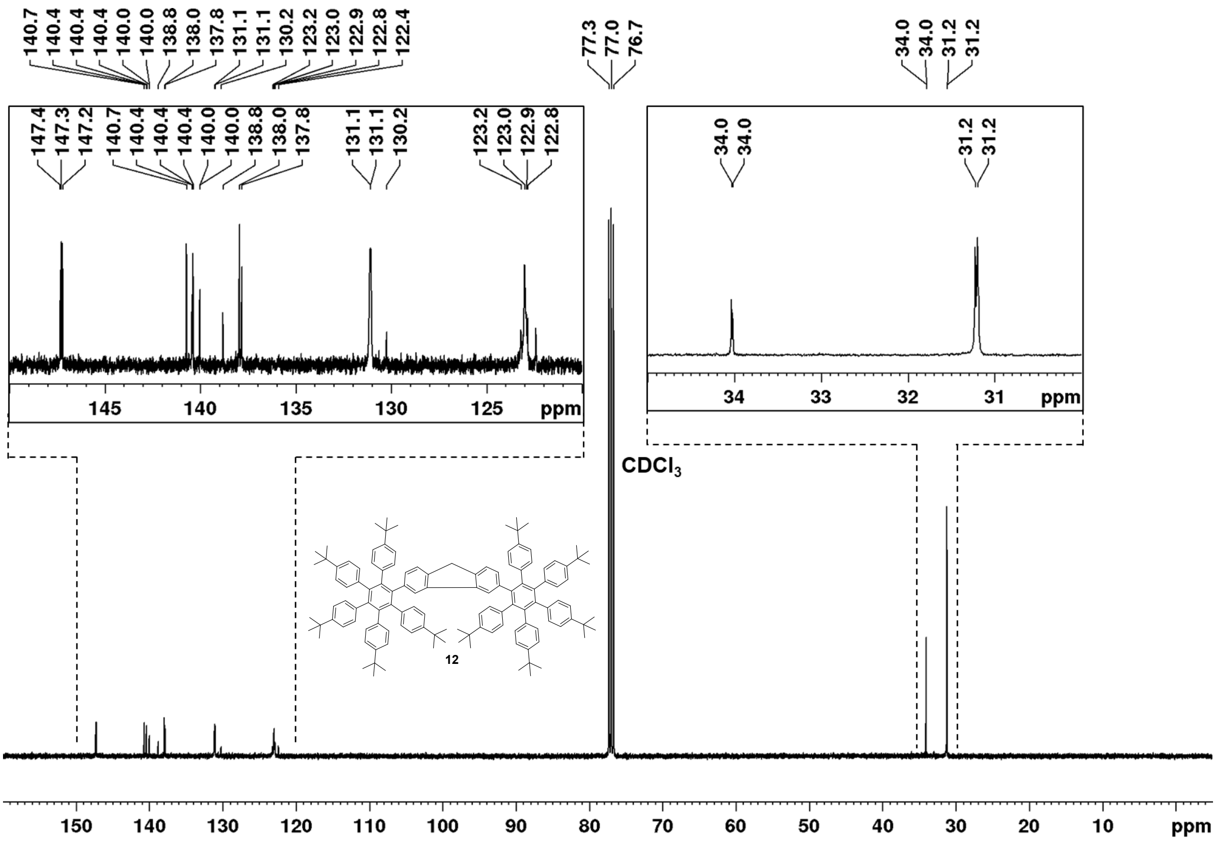

Figure S 110. ^{13}C NMR spectrum of **12** ($CDCl_3$, 100 MHz, rt.).

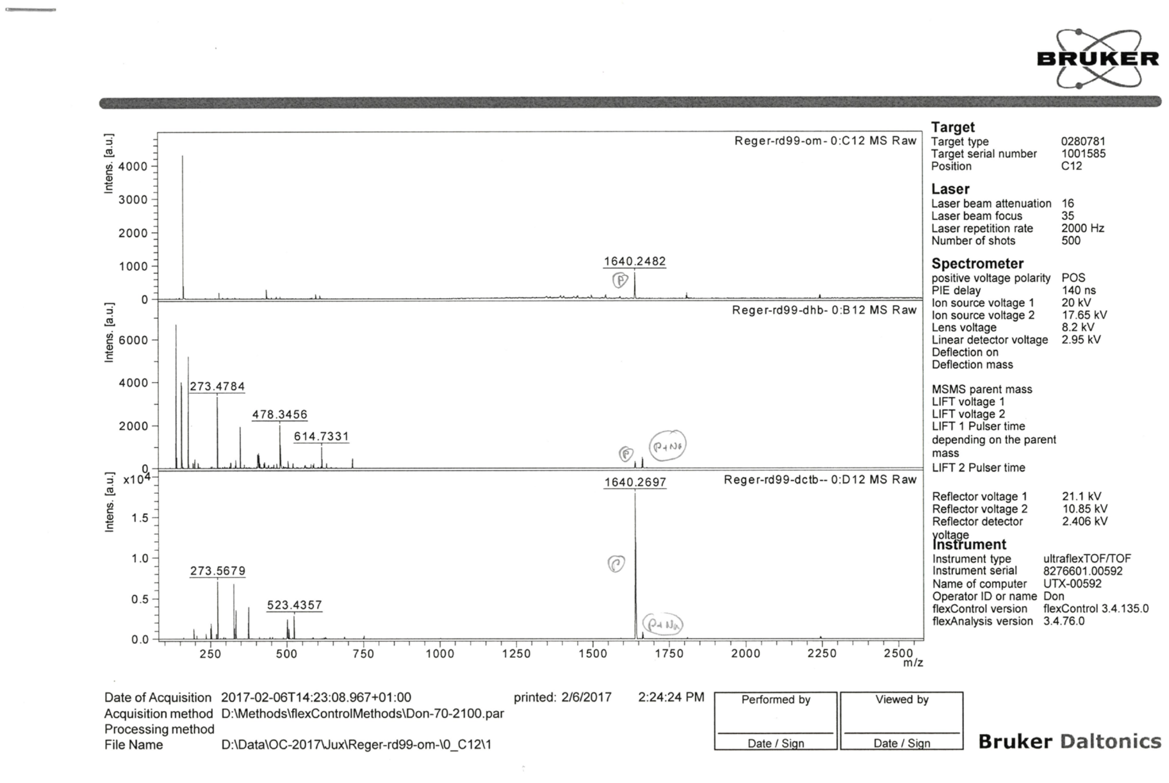

Figure S 111. MS-data (MALDI) of **12** (top: without matrix; middle: dhb; bottom: dctb).

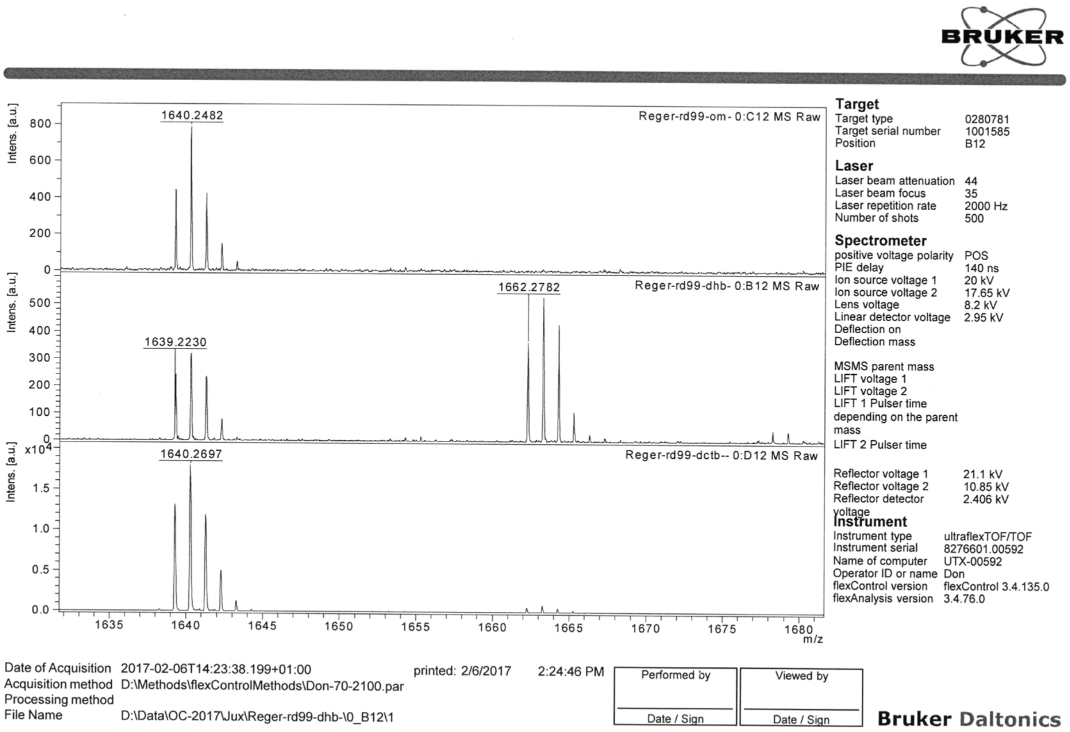

Figure S 112. MS-data (MALDI) of **12** zoom on product peak (top: without matrix; middle: dhb; bottom: dctb).

Mass Spectrum SmartFormula Report

Analysis Info

Analysis Name D:\Data\Jux-2017-\Reger-RD-99-appi-000001.d
Method tune_mid_pos_APPI.m
Sample Name
Comment Tol

Acquisition Date 2/6/2017 3:48:13 PM
Operator MD
Instrument maXis 288882.20183

Acquisition Parameter

Source Type	APPI	Ion Polarity	Positive	Set Nebulizer	2.5 Bar
Focus	Not active	Set Capillary	700 V	Set Dry Heater	200 °C
Scan Begin	300 m/z	Set End Plate Offset	-500 V	Set Dry Gas	1.5 l/min
Scan End	2900 m/z	Set Charging Voltage	0 V	Set Divert Valve	Waste
		Set Corona	0 nA	Set APCI Heater	400 °C

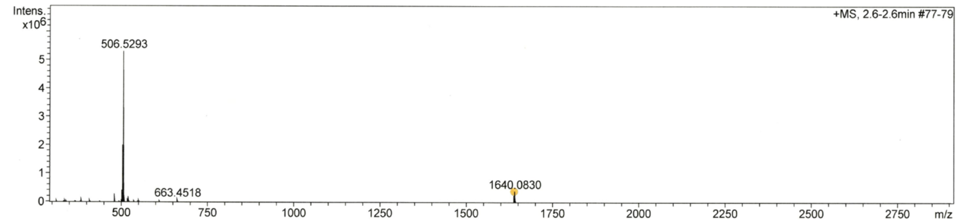

Meas. m/z	#	Ion Formula	m/z	err [ppm]	mSigma	# mSigma	Score	rdb	e⁻ Conf	N-Rule
1639.0783	1	C125H138	1639.0793	0.6	66.8	1	68.99	57.0	odd	ok
	2	C121H5N13	1639.0785	0.2	67.2	2	100.00	126.0	odd	ok
	3	C123H7N10O	1639.0799	1.0	67.5	3	46.86	125.5	even	ok
	4	C122H11N6O5	1639.0785	0.2	71.1	4	86.67	120.5	even	ok
	5	C120H9N9O4	1639.0772	-0.7	71.2	5	56.13	121.0	odd	ok

Reger-RD-99-appi-000001.d
Bruker Compass DataAnalysis 4.2 printed: 2/6/2017 3:53:48 PM by: MD Page 1 of 1

Figure S 113. HRMS data (APPI; toluene) of **12** (overview).

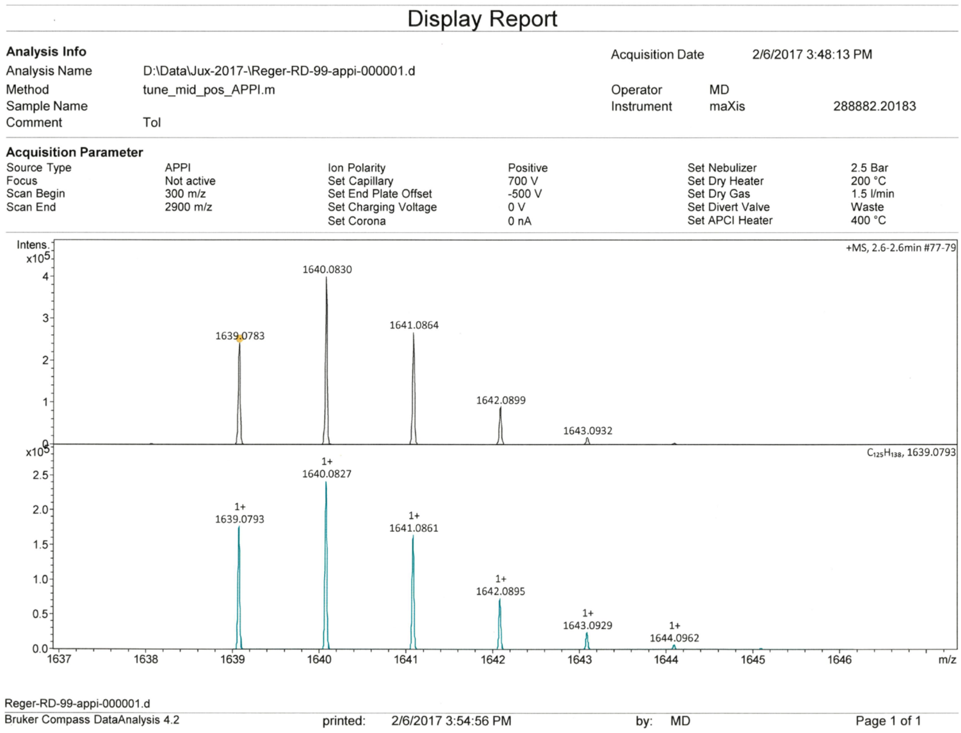

Figure S 114. HRMS data (APPI; toluene) of **12** (top: measured; bottom: calculated).

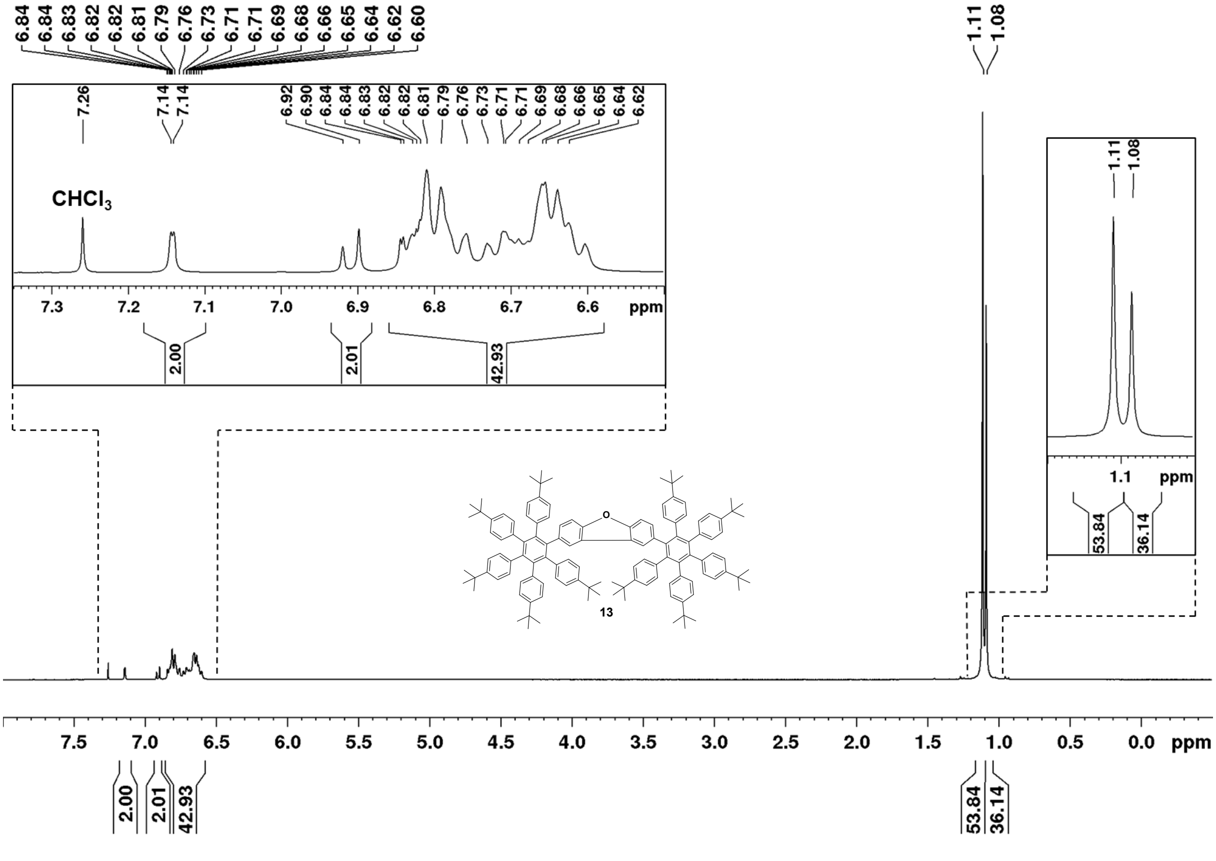

Figure S 115. ^{1}H NMR spectrum of **13** ($CDCl_3$, 400 MHz, rt.).

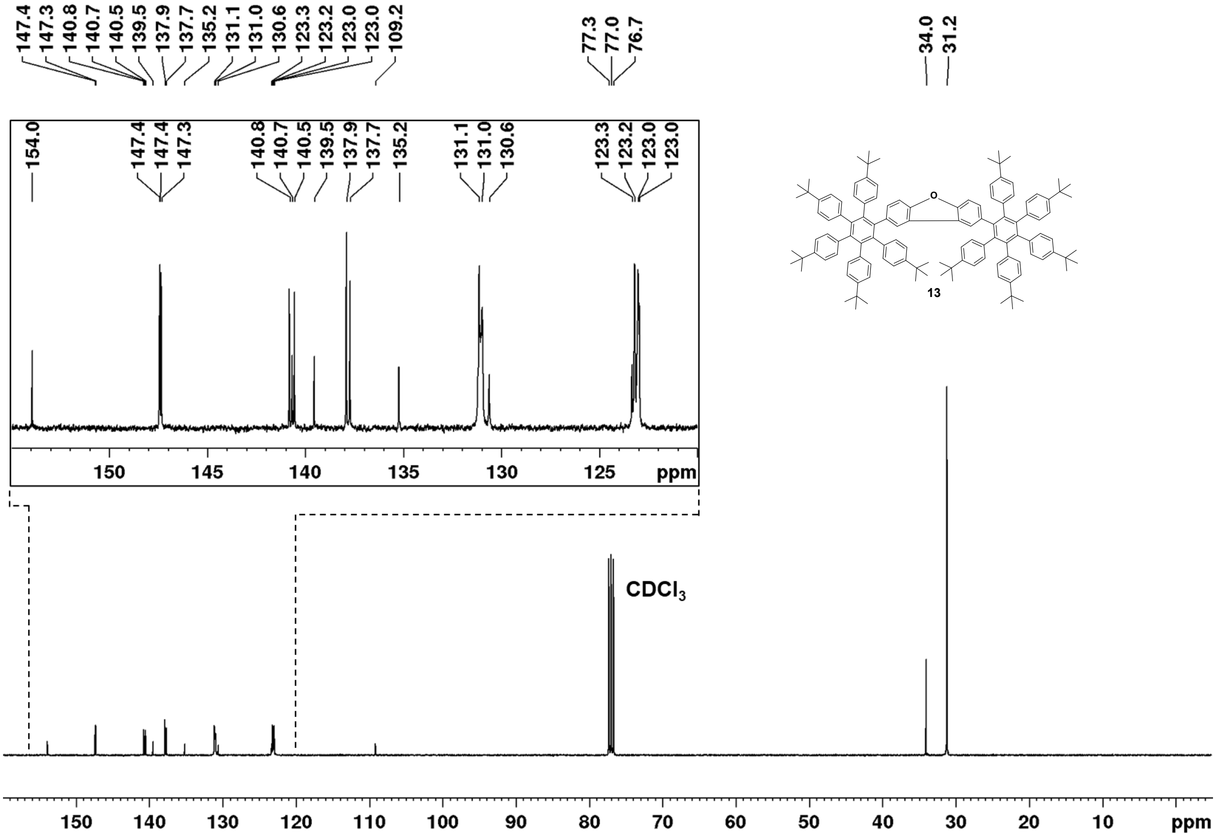

Figure S 116. ^{13}C NMR spectrum of **13** ($CDCl_3$, 100 MHz, rt.).

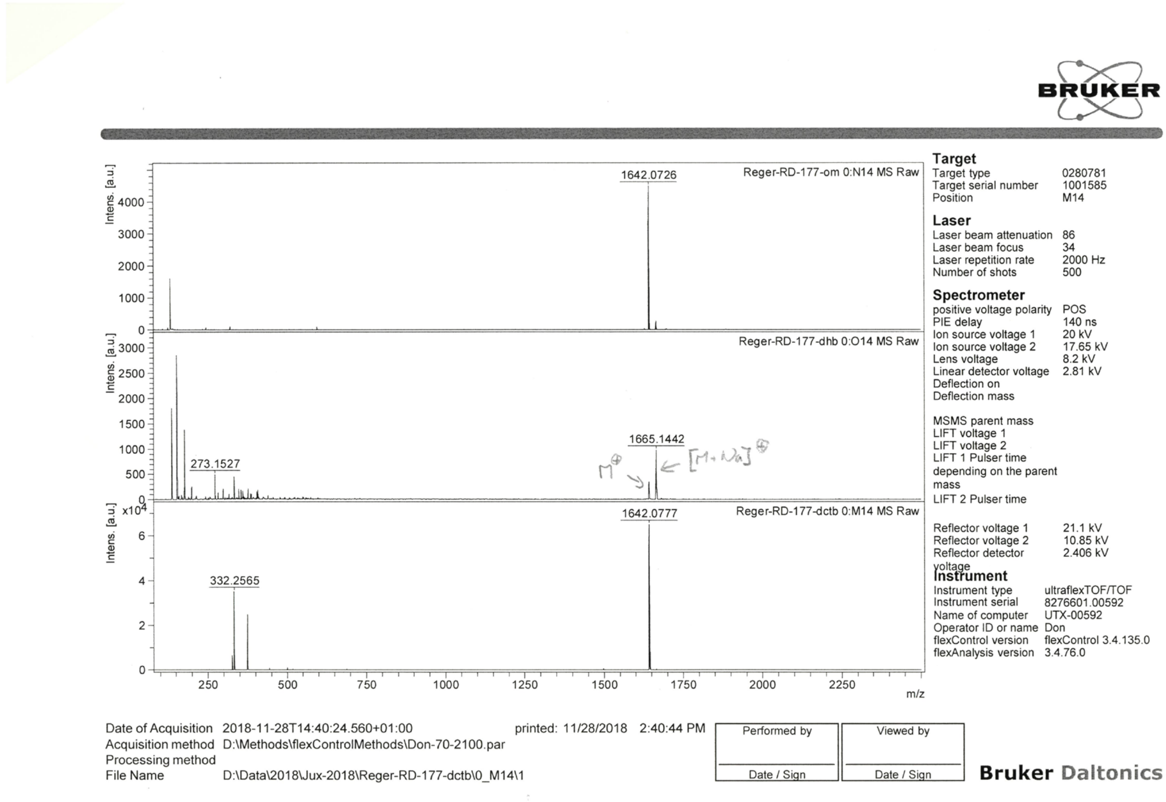

Figure S 117. MS-data (MALDI) of **13** (top: without matrix; middle: dhb; bottom: dctb).

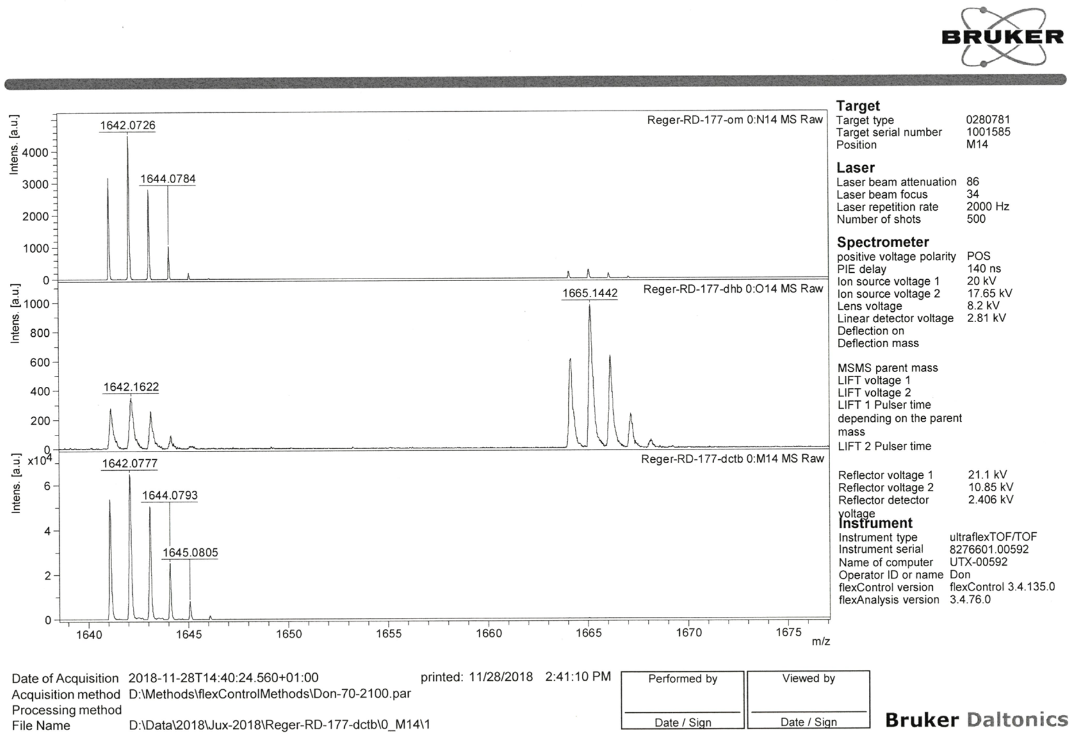

Figure S 118. MS-data (MALDI) of **13** zoom on product peak (top: without matrix; middle: dhb; bottom: dctb).

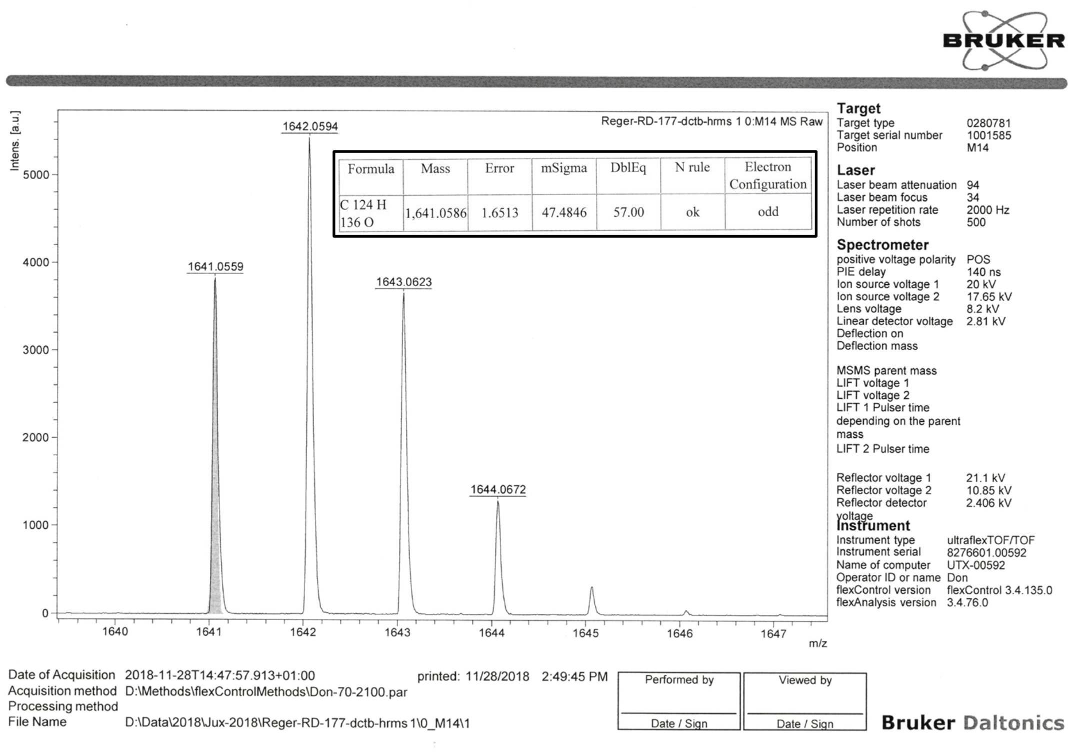

Figure S 119. HRMS data (MALDI; dctb) of **13** (grey background: simulated). Insert: Calculated mass.

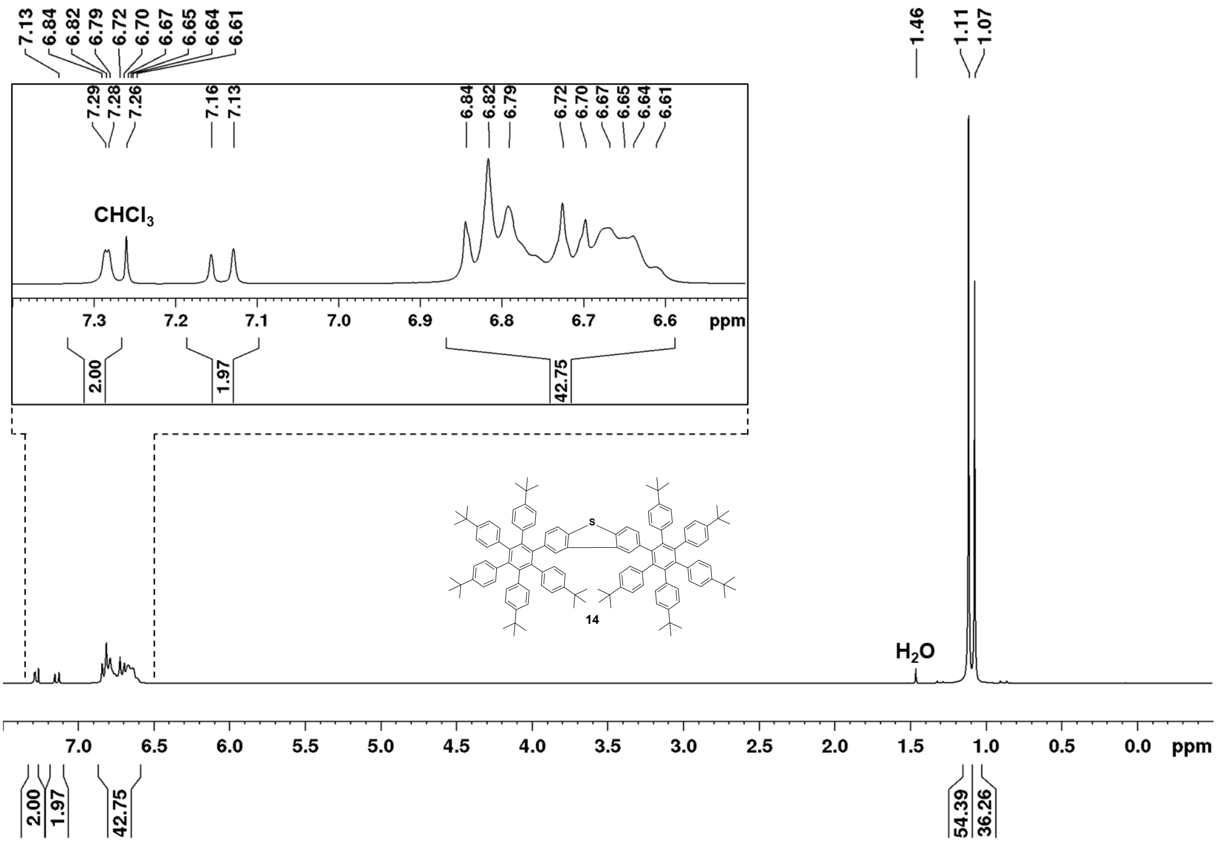

Figure S 120. ^{1}H NMR spectrum of **14** ($CDCl_3$, 300 MHz, rt.).

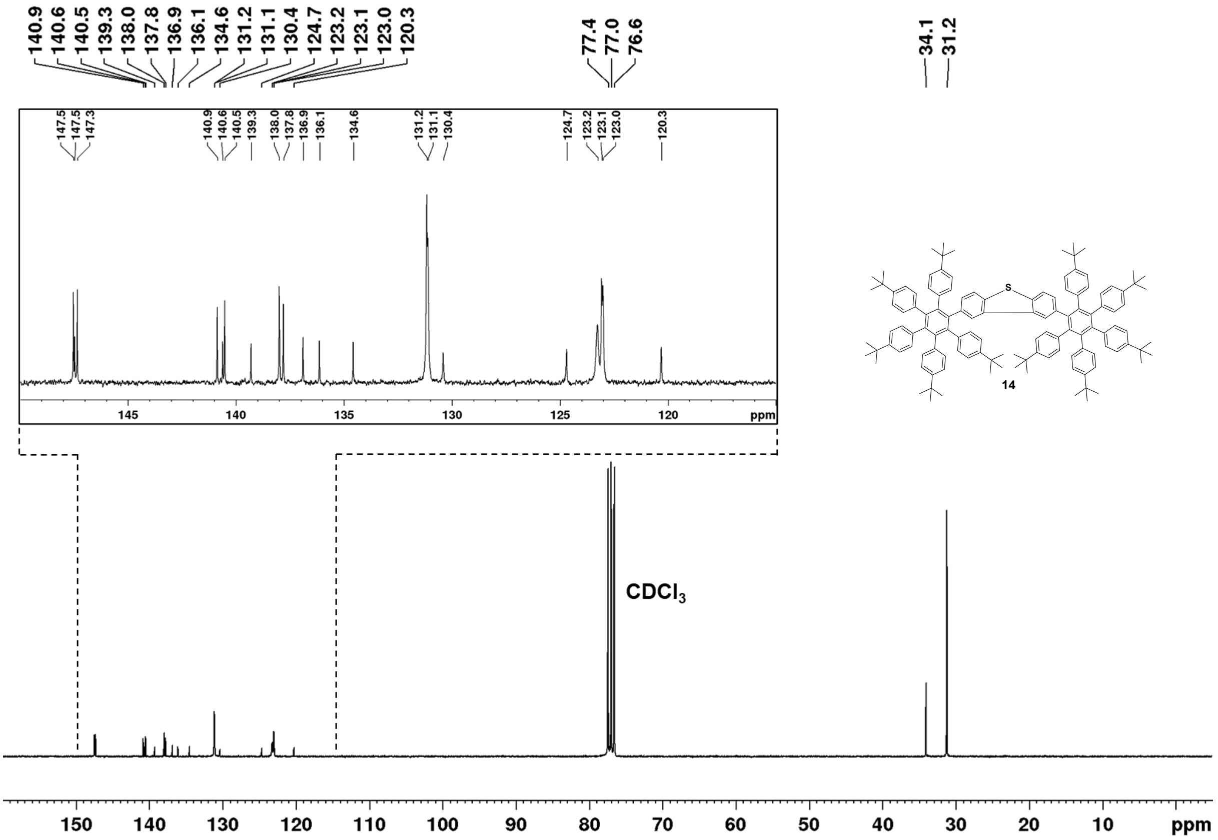

Figure S 121. ^{13}C NMR spectrum of **14** ($CDCl_3$, 75 MHz, rt.).

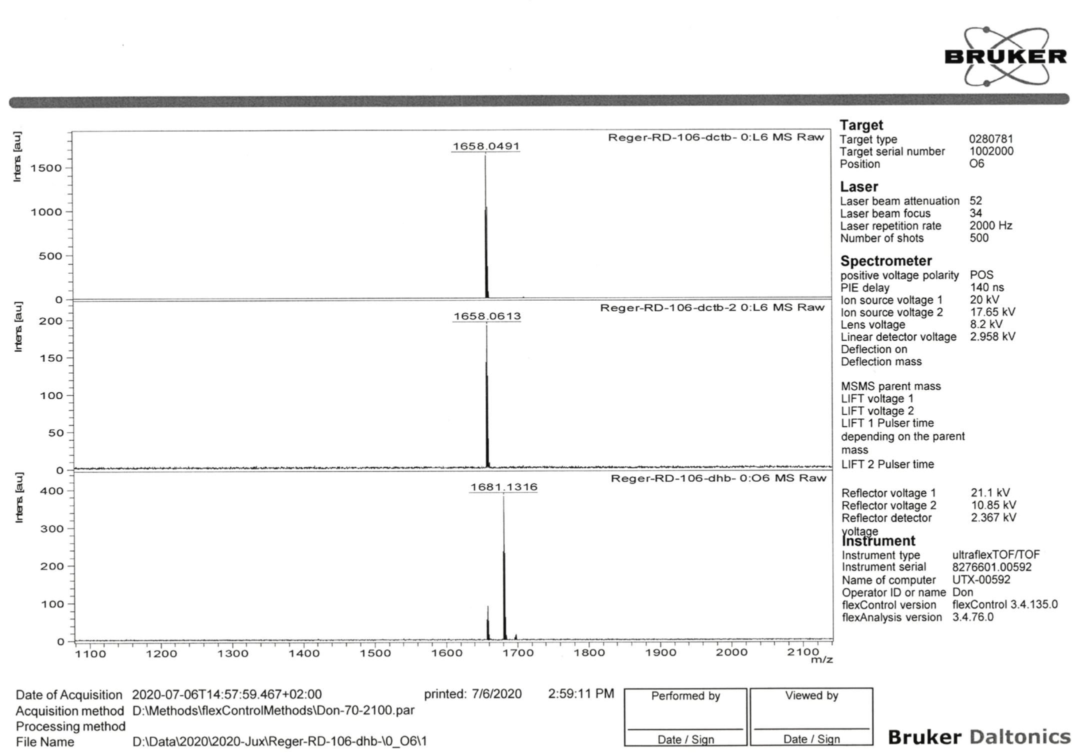

Figure S 122. MS-data (MALDI) of **14** (top/middle: dctb; bottom: dhb).

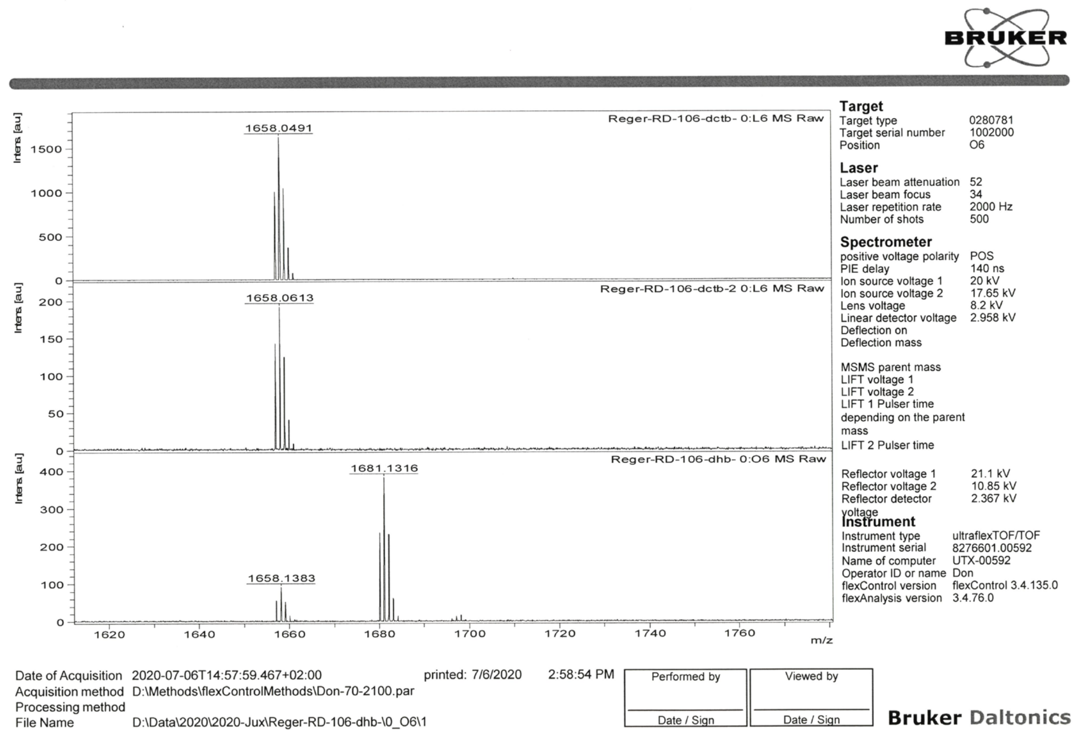

Figure S 123. MS-data (MALDI) of **14** zoom on product peak (top/middle: dctb; bottom: dhb).

Mass Spectrum SmartFormula Report

Analysis Info

Analysis Name	D:\Data\2020\Jux-2020\Reger-RD-106-appi-2.d	Acquisition Date	7/6/2020 3:43:05 PM
Method	APPI_pos_low_t3.d.m	Operator	MD
Sample Name		Instrument	maXis 288882.20183
Comment			

Acquisition Parameter

Source Type	APPI	Ion Polarity	Positive	Set Nebulizer	5.2 Bar
Focus	Not active	Set Capillary	700 V	Set Dry Heater	220 °C
Scan Begin	80 m/z	Set End Plate Offset	-500 V	Set Dry Gas	1.2 l/min
Scan End	1900 m/z	Set Charging Voltage	0 V	Set Divert Valve	Waste
		Set Corona	0 nA	Set APCI Heater	300 °C

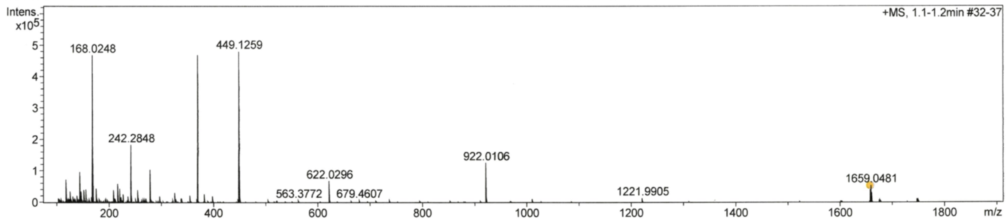

[M+H]⊕

Meas. m/z	#	Ion Formula	m/z	err [ppm]	mSigma	# mSigma	Score	rdb	e⁻ Conf	N-Rule
1658.0435	1	C124H137S	1658.0436	0.1	87.1	1	100.00	56.5	even	ok
	2	C124H8N7O2S	1658.0455	1.2	92.8	2	25.49	124.5	even	ok

Reger-RD-106-appi-2.d
Bruker Compass DataAnalysis 4.2 printed: 7/6/2020 3:46:08 PM by: MD Page 1 of 1

Figure S 124. HRMS data (APPI; MeOH) of **14** (overview).

Display Report

Analysis Info

Analysis Name	D:\Data\2020\Jux-2020\Reger-RD-106-appi-2.d	Acquisition Date	7/6/2020 3:43:05 PM
Method	APPI_pos_low_t3.d.m	Operator	MD
Sample Name		Instrument	maXis 288882.20183
Comment			

Acquisition Parameter

Source Type	APPI	Ion Polarity	Positive	Set Nebulizer	5.2 Bar
Focus	Not active	Set Capillary	700 V	Set Dry Heater	220 °C
Scan Begin	80 m/z	Set End Plate Offset	-500 V	Set Dry Gas	1.2 l/min
Scan End	1900 m/z	Set Charging Voltage	0 V	Set Divert Valve	Waste
		Set Corona	0 nA	Set APCI Heater	300 °C

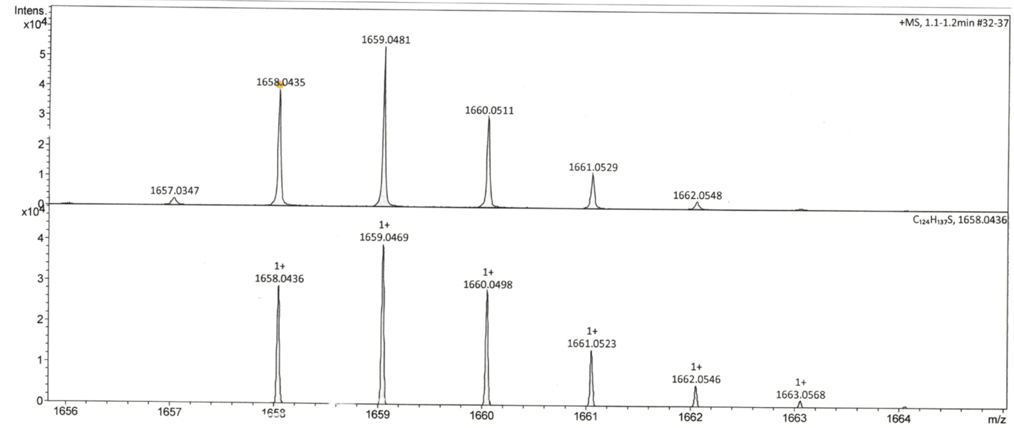

Reger-RD-106-appi-2.d
Bruker Compass DataAnalysis 4.2 printed: 7/6/2020 3:46:52 PM by: MD Page 1 of 1

Figure S 125. HRMS data (APPI; MeOH) of **14** (top: measured; bottom: calculated).

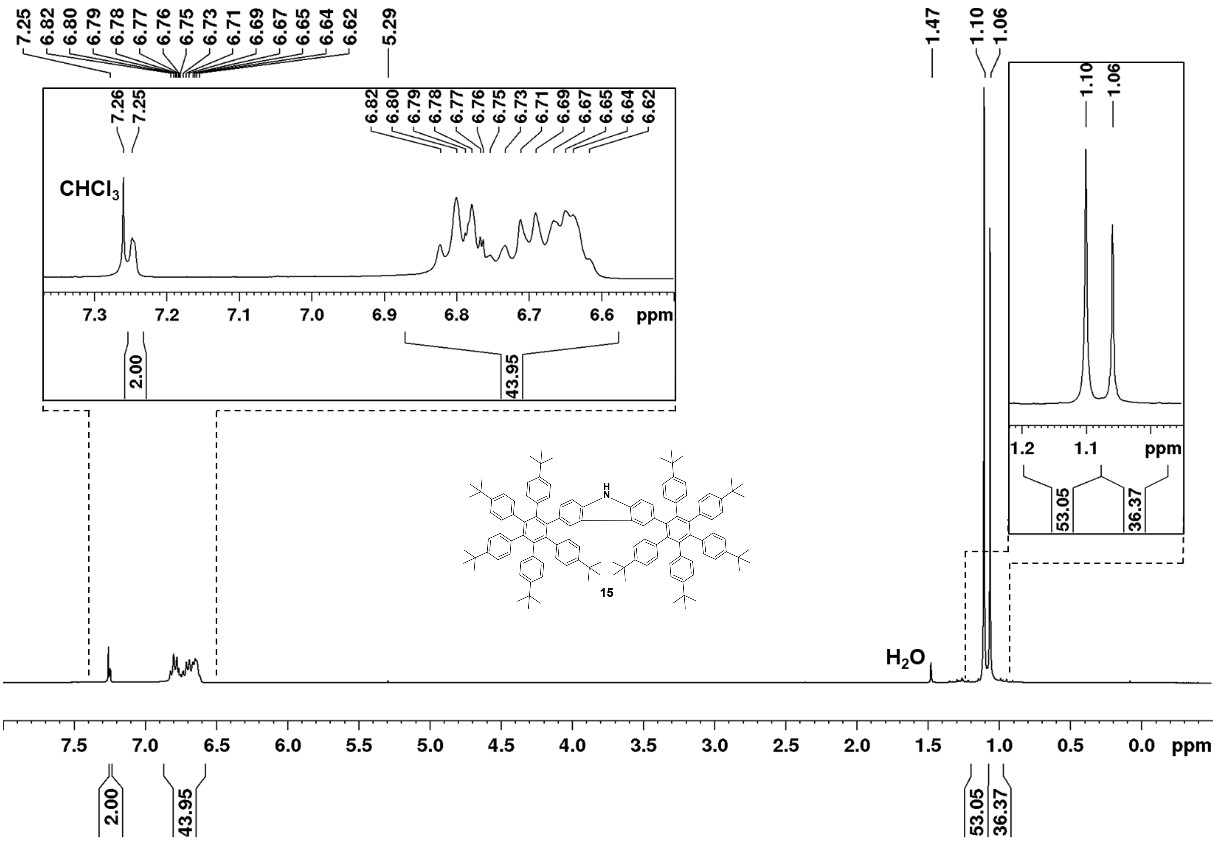

Figure S 126. ^{1}H NMR spectrum of **15** ($CDCl_3$, 400 MHz, rt.).

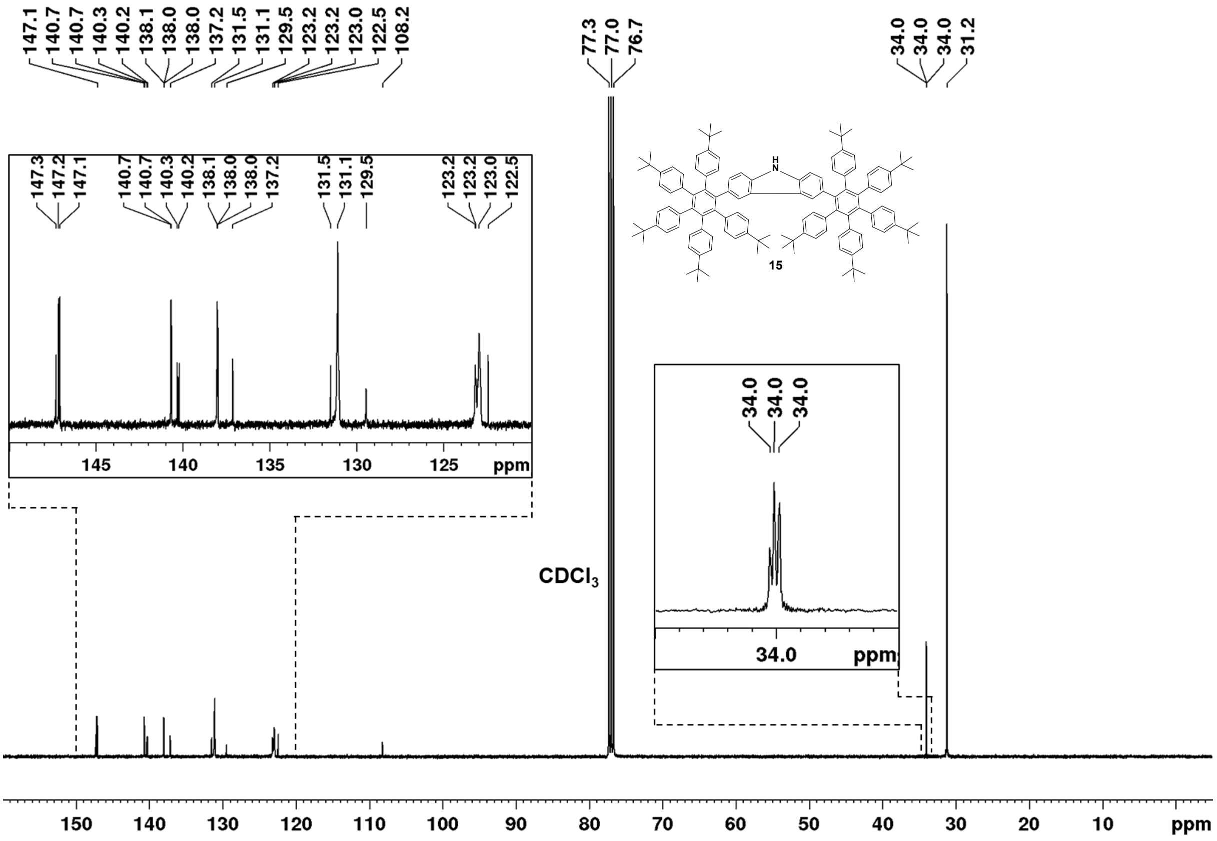

Figure S 127. ^{13}C NMR spectrum of **15** ($CDCl_3$, 100 MHz, rt.).

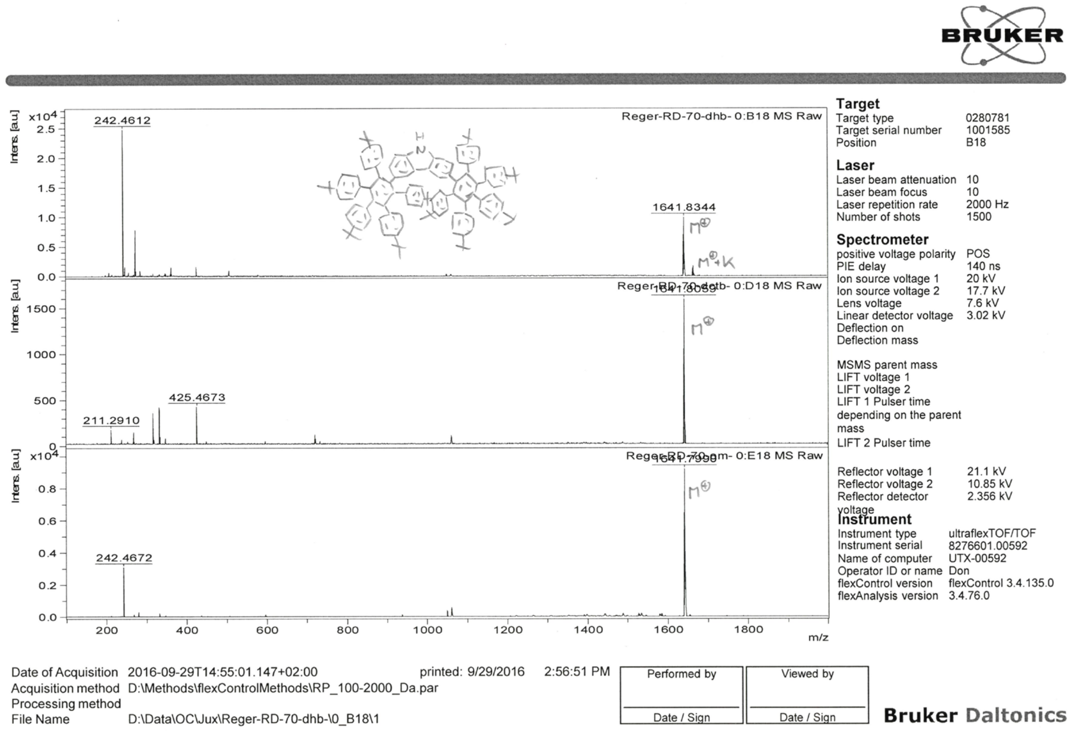

Figure S 128. MS-data (MALDI) of **15** (top: dhb; middle: dctb; bottom: without matrix).

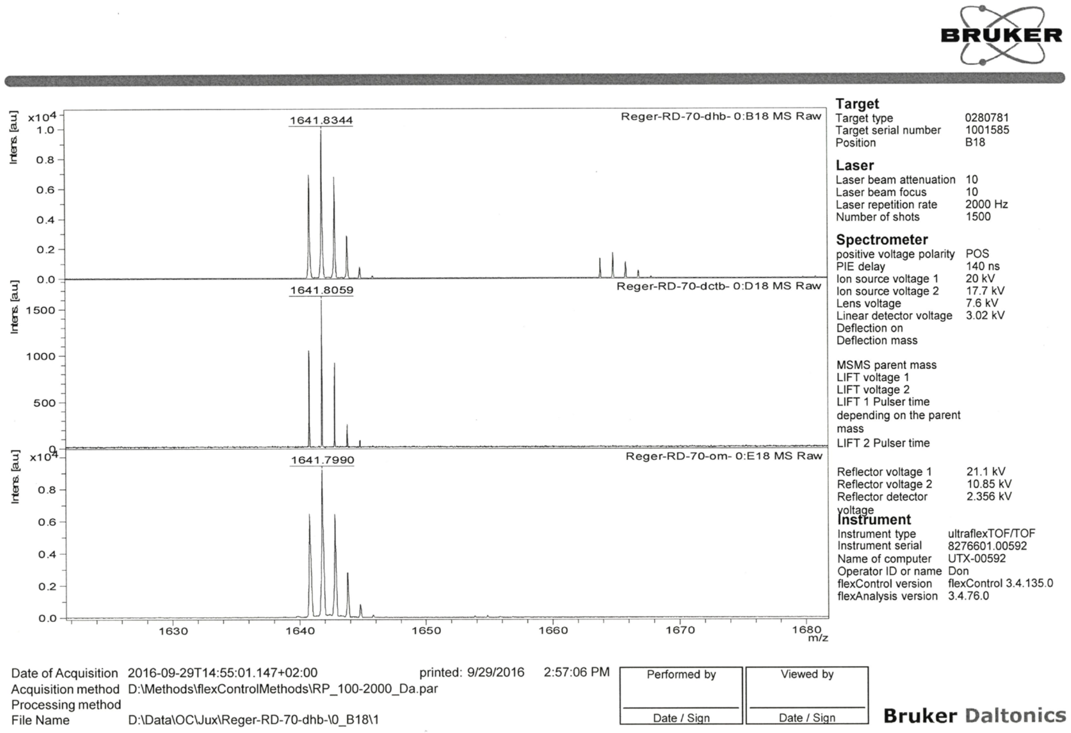

Figure S 129. MS-data (MALDI) of **15** zoom on product peak (top: dhb; middle: dctb; bottom: without matrix).

Mass Spectrum SmartFormula Report

**Analysis Info**		Acquisition Date	9/29/2016 3:18:23 PM
Analysis Name	D:\Data\Jux-2016-\Reger-RD-70-appi-000001.d		
Method	tune_mid_pos_APPI.m	Operator	MD
Sample Name		Instrument	maXis 288882.20183
Comment	Tol		

Acquisition Parameter

Source Type	APPI	Ion Polarity	Positive	Set Nebulizer	2.5 Bar
Focus	Not active	Set Capillary	700 V	Set Dry Heater	200 °C
Scan Begin	300 m/z	Set End Plate Offset	-500 V	Set Dry Gas	1.5 l/min
Scan End	2000 m/z	Set Charging Voltage	0 V	Set Divert Valve	Waste
		Set Corona	0 nA	Set APCI Heater	400 °C

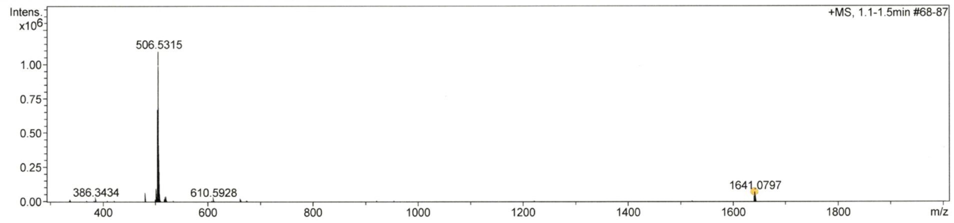

M⊕

Meas. m/z	#	Ion Formula	m/z	err [ppm]	mSigma	# mSigma	Score	rdb	e⁻ Conf	N-Rule
1640.0756	1	C124H137N	1640.0746	-0.6	58.1	1	100.00	57.0	odd	ok
	2	C125H14NO7	1640.0765	0.6	63.7	2	87.38	119.5	even	ok
	3	C122H16O10	1640.0738	-1.1	65.8	3	47.14	115.0	odd	ok
	4	C97H28O28	1640.0762	0.4	130.7	4	4.57	84.0	odd	ok
	5	C82H34NO38	1640.0753	-0.1	168.9	5	0.44	66.5	even	ok

Reger-RD-70-appi-000001.d
Bruker Compass DataAnalysis 4.2 printed: 9/29/2016 3:22:22 PM by: MD Page 1 of 1

Figure S 130. HRMS data (APPI; toluene) of **15** (overview).

Display Report

**Analysis Info**		Acquisition Date	9/29/2016 3:18:23 PM
Analysis Name	D:\Data\Jux-2016-\Reger-RD-70-appi-000001.d		
Method	tune_mid_pos_APPI.m	Operator	MD
Sample Name		Instrument	maXis 288882.20183
Comment	Tol		

Acquisition Parameter

Source Type	APPI	Ion Polarity	Positive	Set Nebulizer	2.5 Bar
Focus	Not active	Set Capillary	700 V	Set Dry Heater	200 °C
Scan Begin	300 m/z	Set End Plate Offset	-500 V	Set Dry Gas	1.5 l/min
Scan End	2000 m/z	Set Charging Voltage	0 V	Set Divert Valve	Waste
		Set Corona	0 nA	Set APCI Heater	400 °C

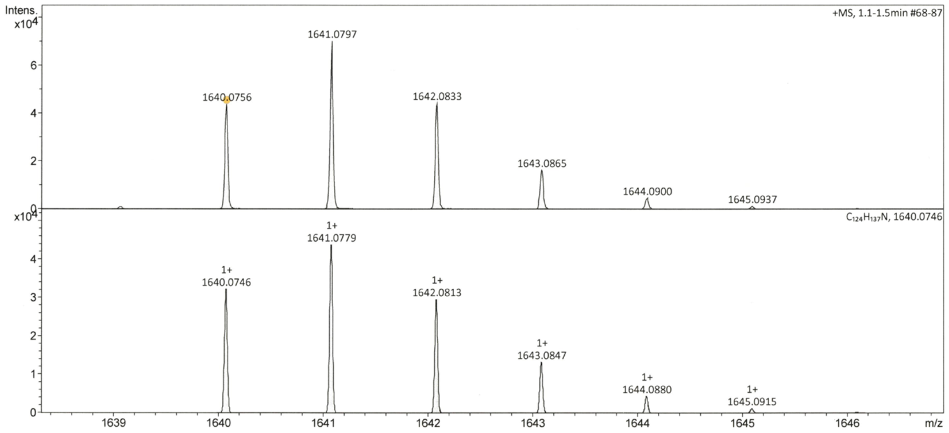

Reger-RD-70-appi-000001.d
Bruker Compass DataAnalysis 4.2 printed: 9/29/2016 3:22:42 PM by: MD Page 1 of 1

Figure S 131. HRMS data (APPI; toluene) of **15** (top: measured; bottom: calculated).

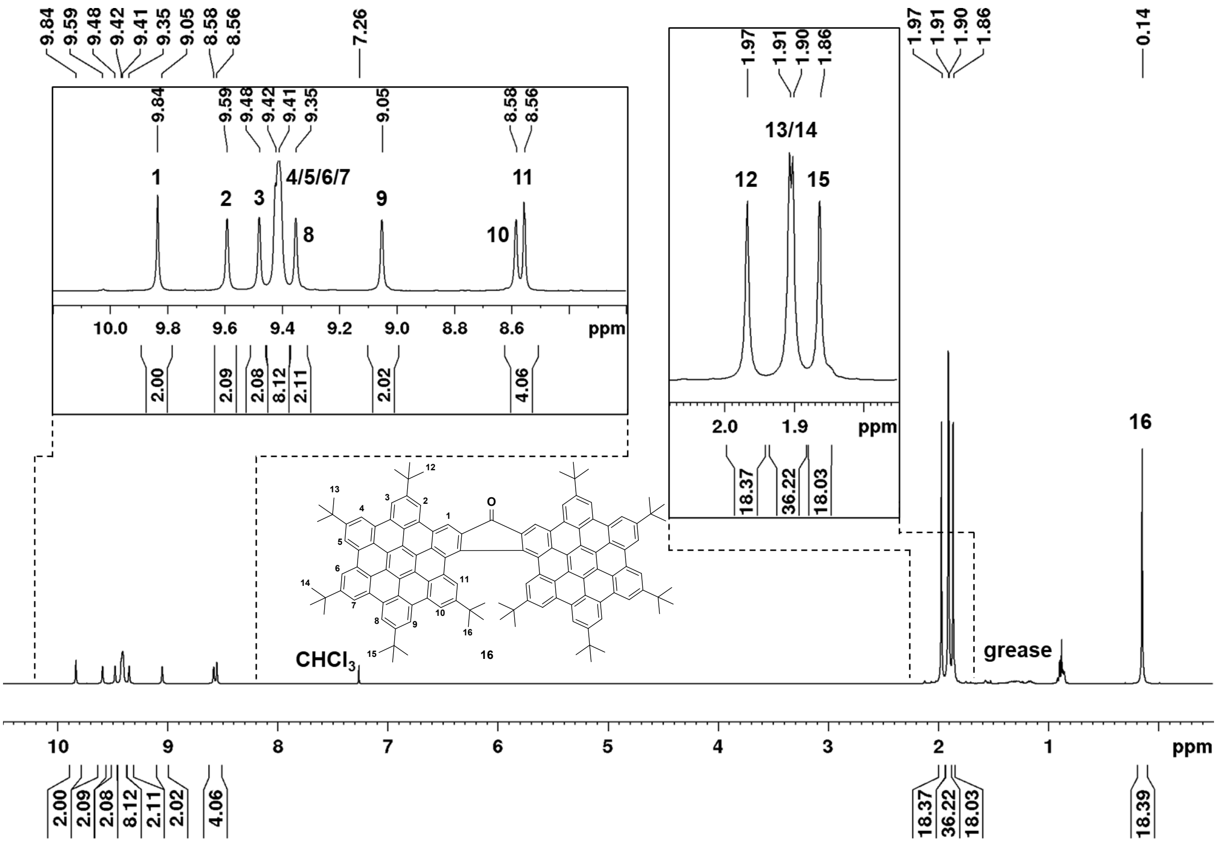

Figure S 132. ^{1}H NMR spectrum of **16** ($CDCl_3$, 400 MHz, rt.).

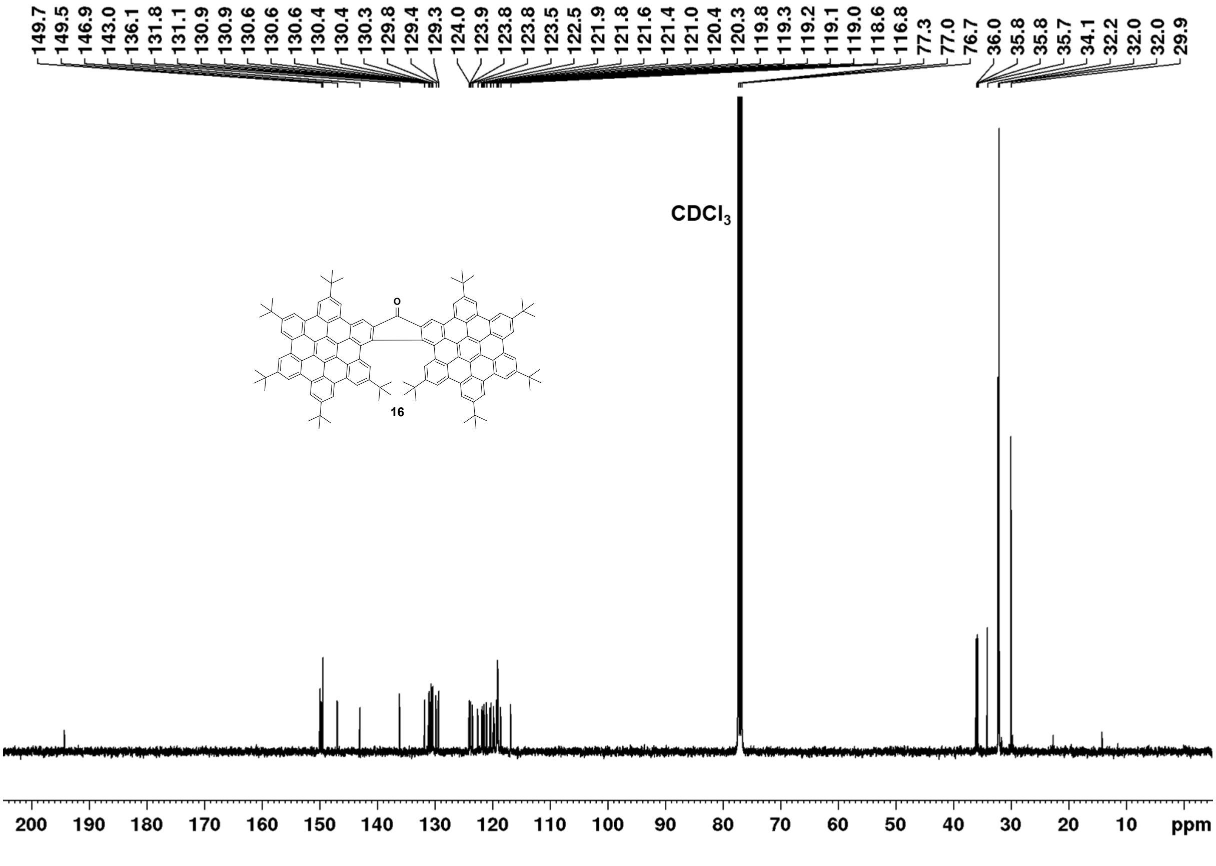

Figure S 133. Overview of the ^{13}C NMR spectrum of **16** ($CDCl_3$, 100 MHz, rt.).

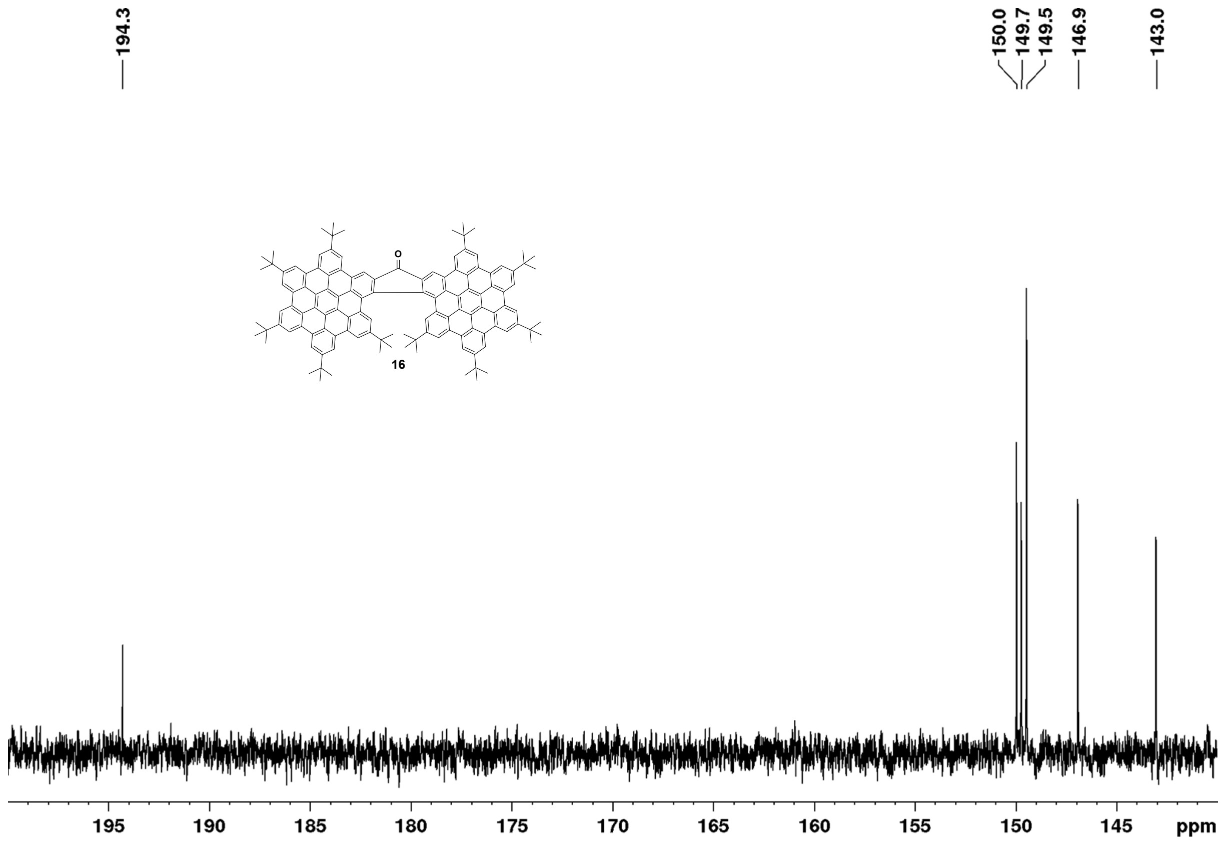

Figure S 134. Cutout (aromatic region) of the ^{13}C NMR spectrum of **16** ($CDCl_3$, 100 MHz, rt.).

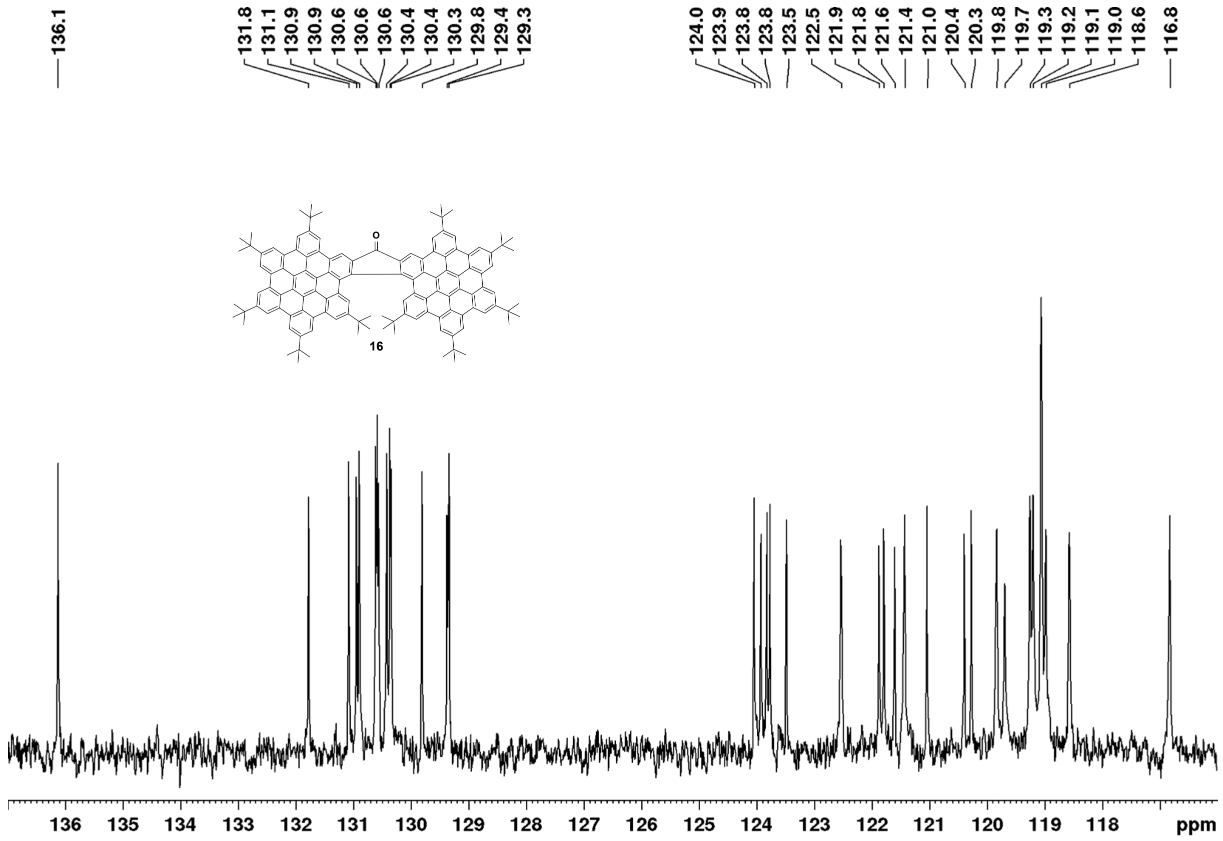

Figure S 135. Cutout (aromatic region) of the ^{13}C NMR spectrum of **16** ($CDCl_3$, 100 MHz, rt.).

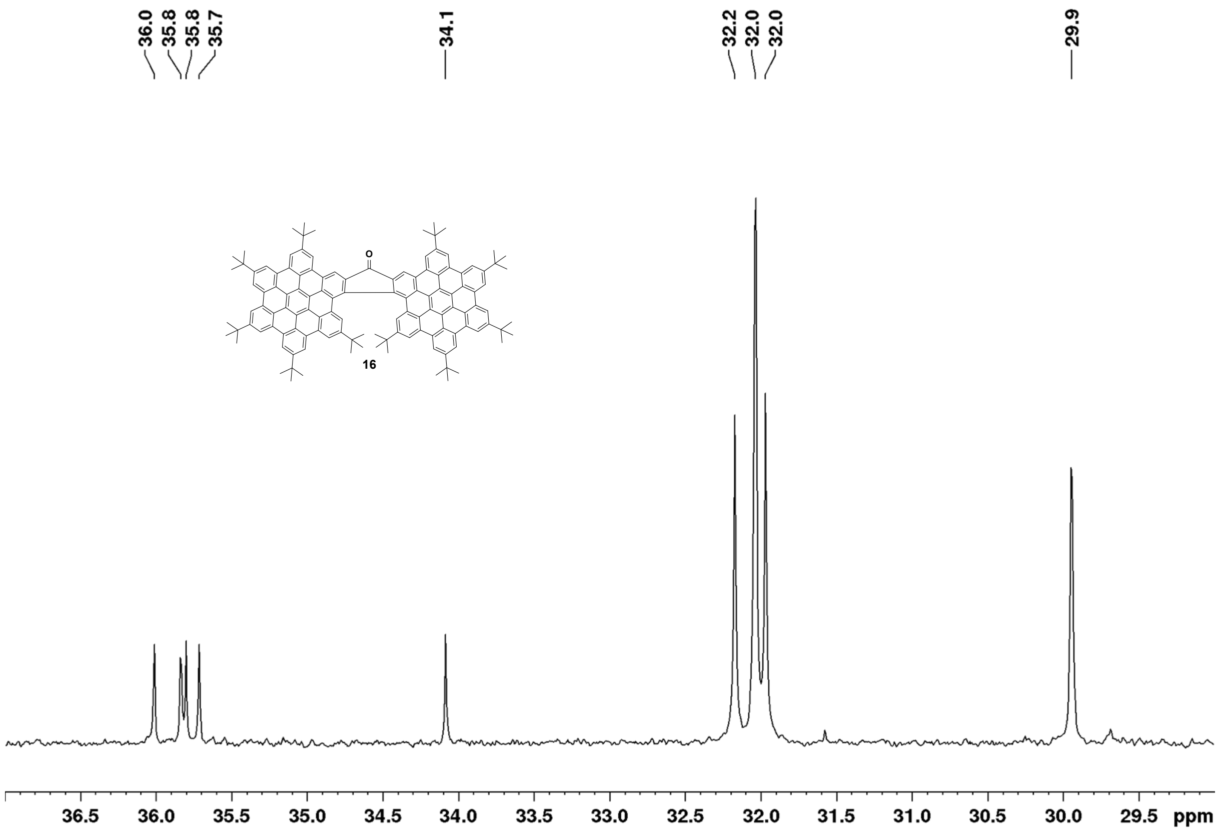

Figure S 136. Cutout (aliphatic region) of the ^{13}C NMR spectrum of **16** ($CDCl_3$, 100 MHz, rt.).

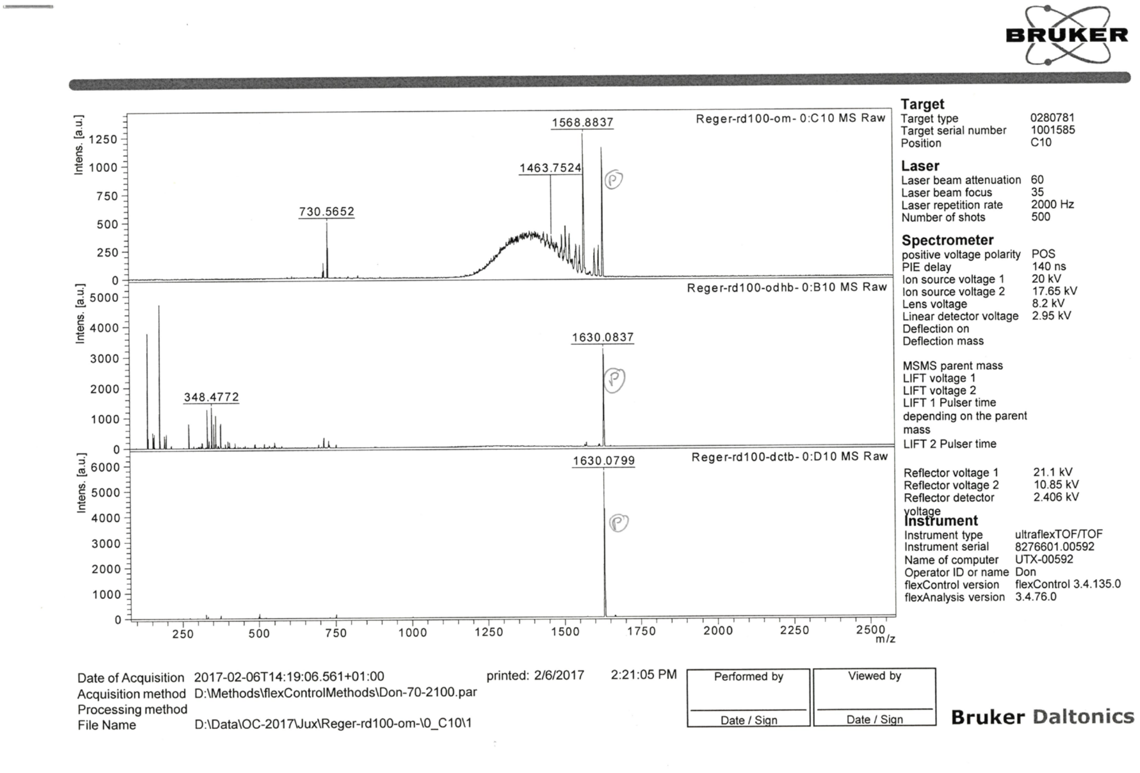

Figure S 137. MS-data (MALDI) of **16** (top: without matrix; middle: dhb; bottom: dctb).

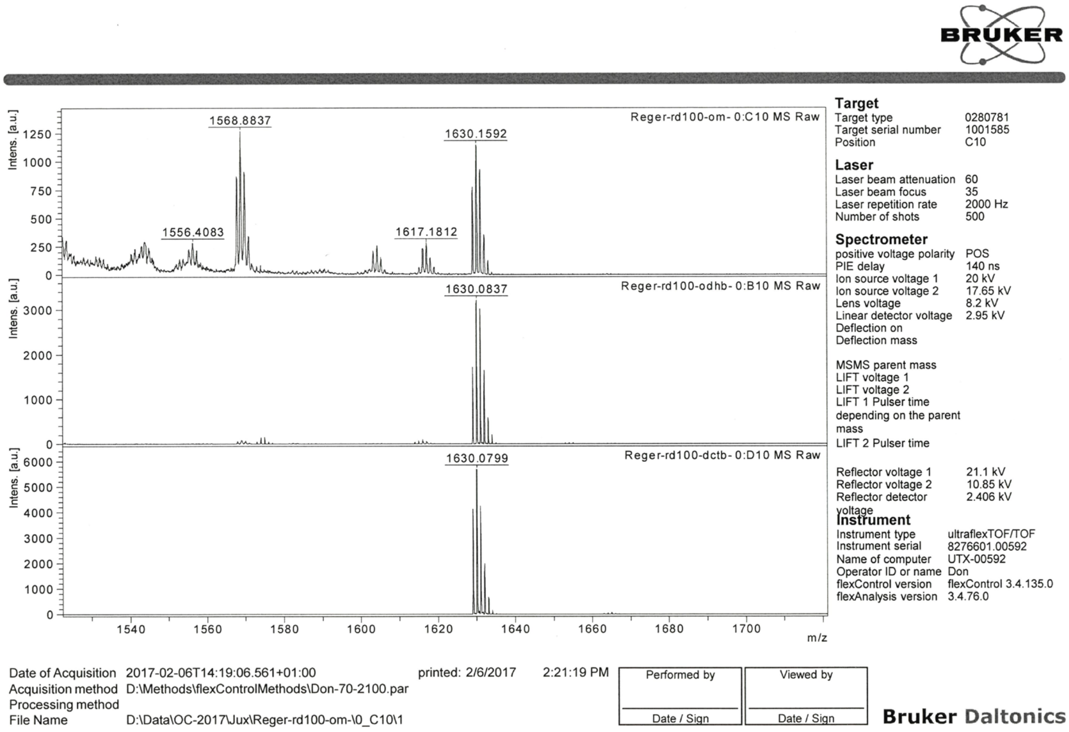

Figure S 138. MS-data (MALDI) of **16** zoom on product peak (top: without matrix; middle: dhb; bottom: dctb).

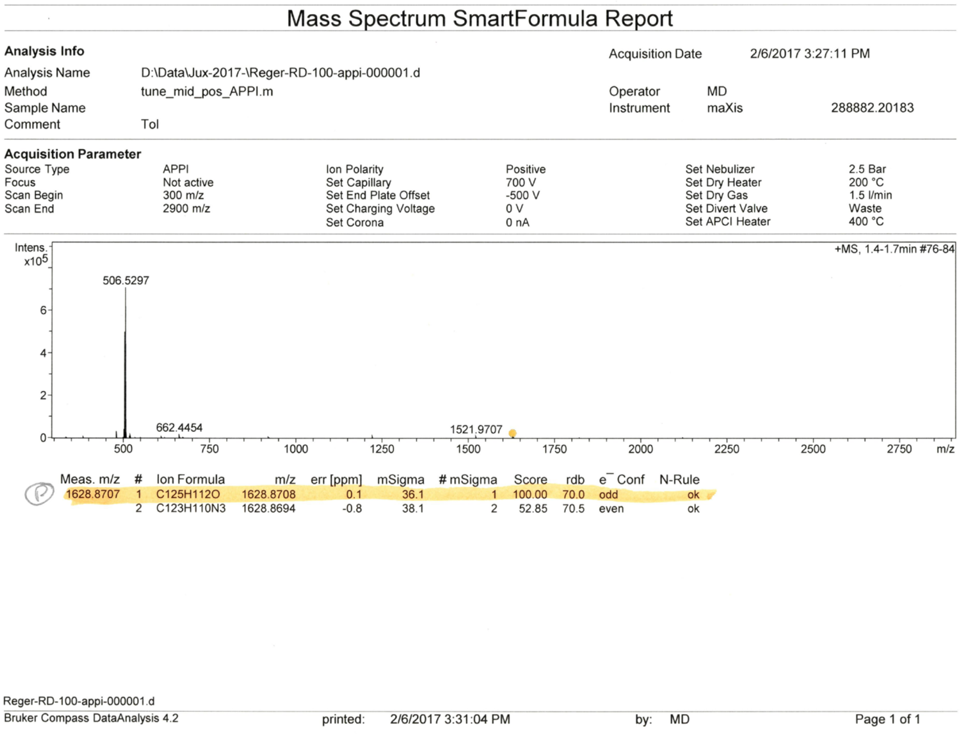

Figure S 139. HRMS data (APPI; toluene) of **16** (overview).

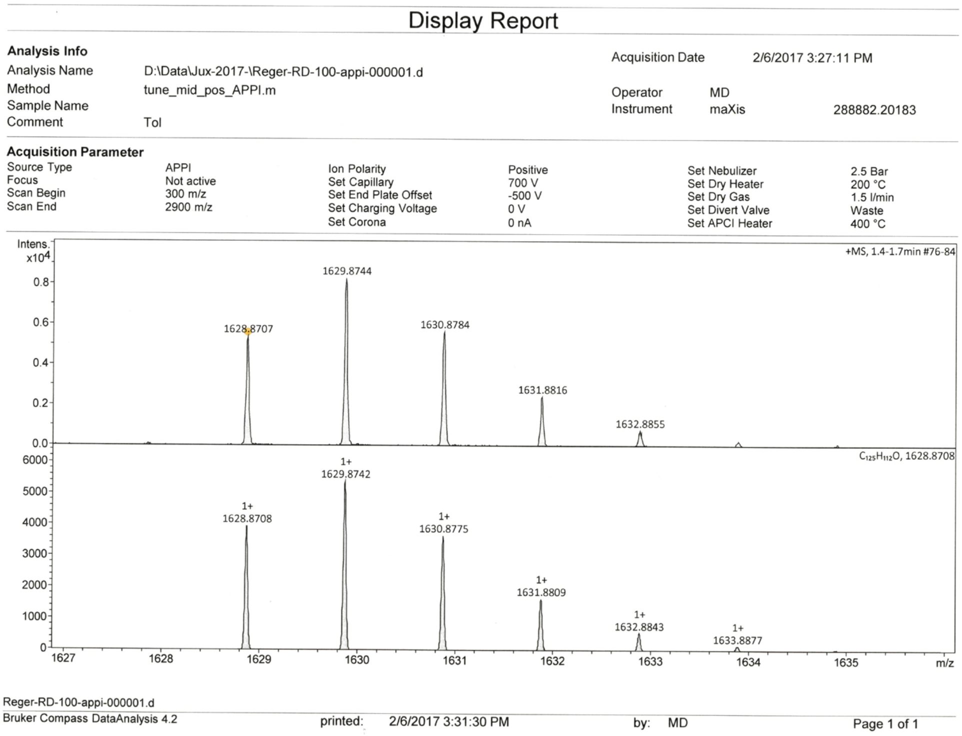

Figure S 140. HRMS data (APPI; toluene) of **16** (top: measured; bottom: calculated).

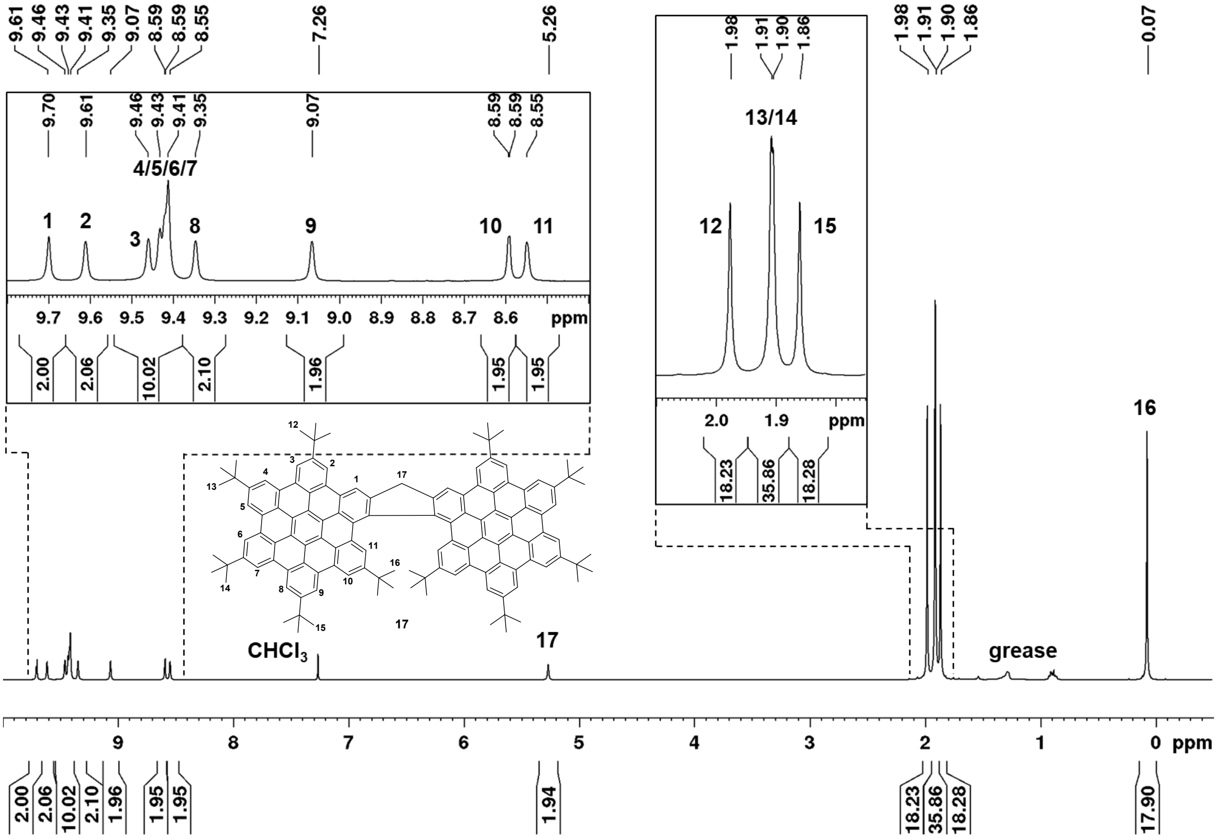

Figure S 141. ^{1}H NMR spectrum of **17** ($CDCl_3$, 400 MHz, rt.).

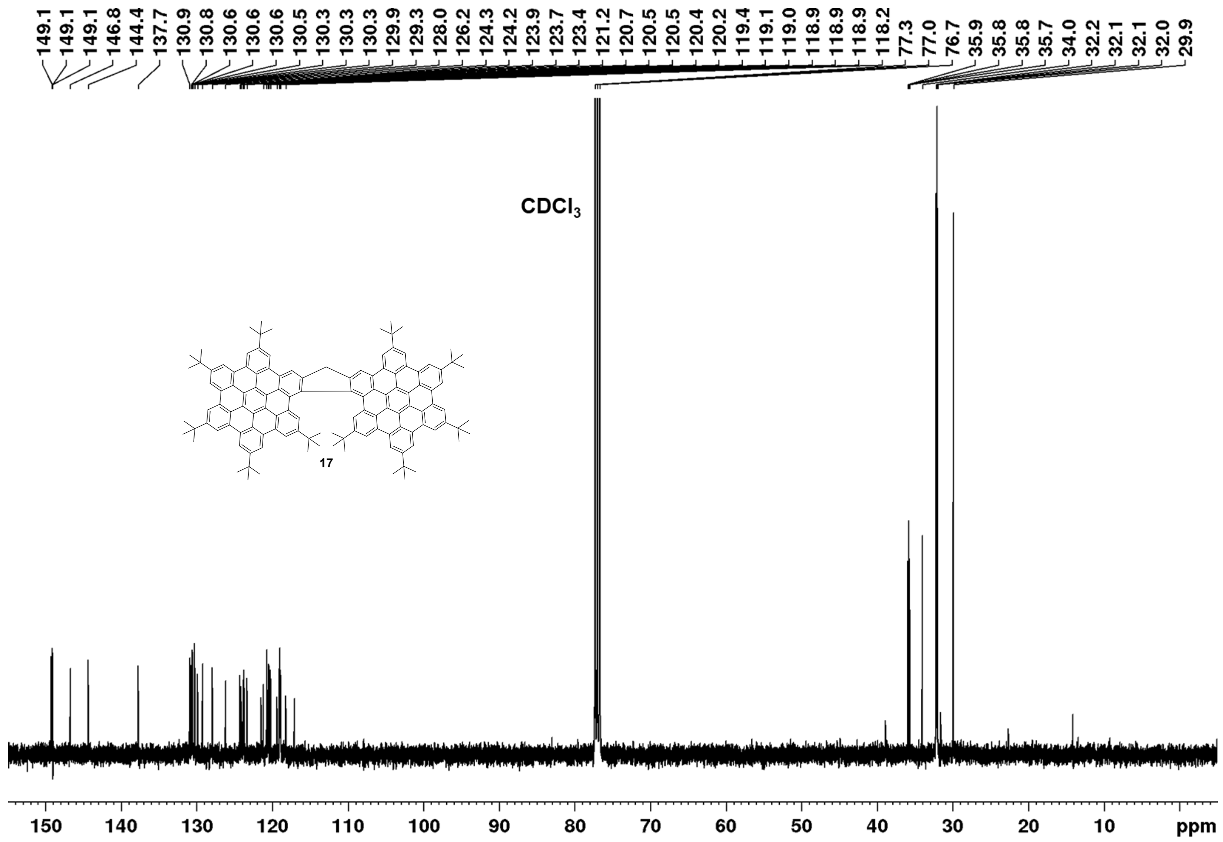

Figure S 142. Overview of the ^{13}C NMR spectrum of **17** ($CDCl_3$, 100 MHz, rt.).

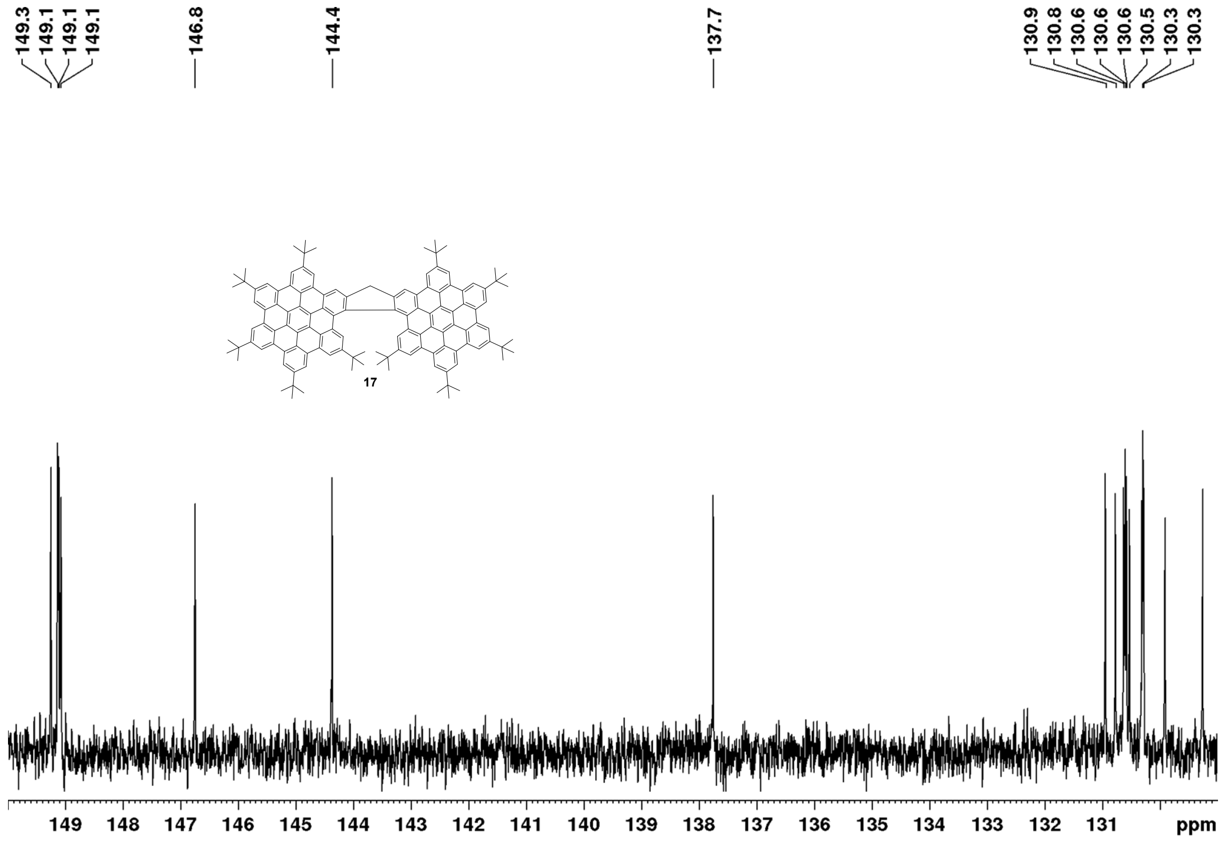

Figure S 143. Cutout (aromatic region) of the ^{13}C NMR spectrum of **17** ($CDCl_3$, 100 MHz, rt.).

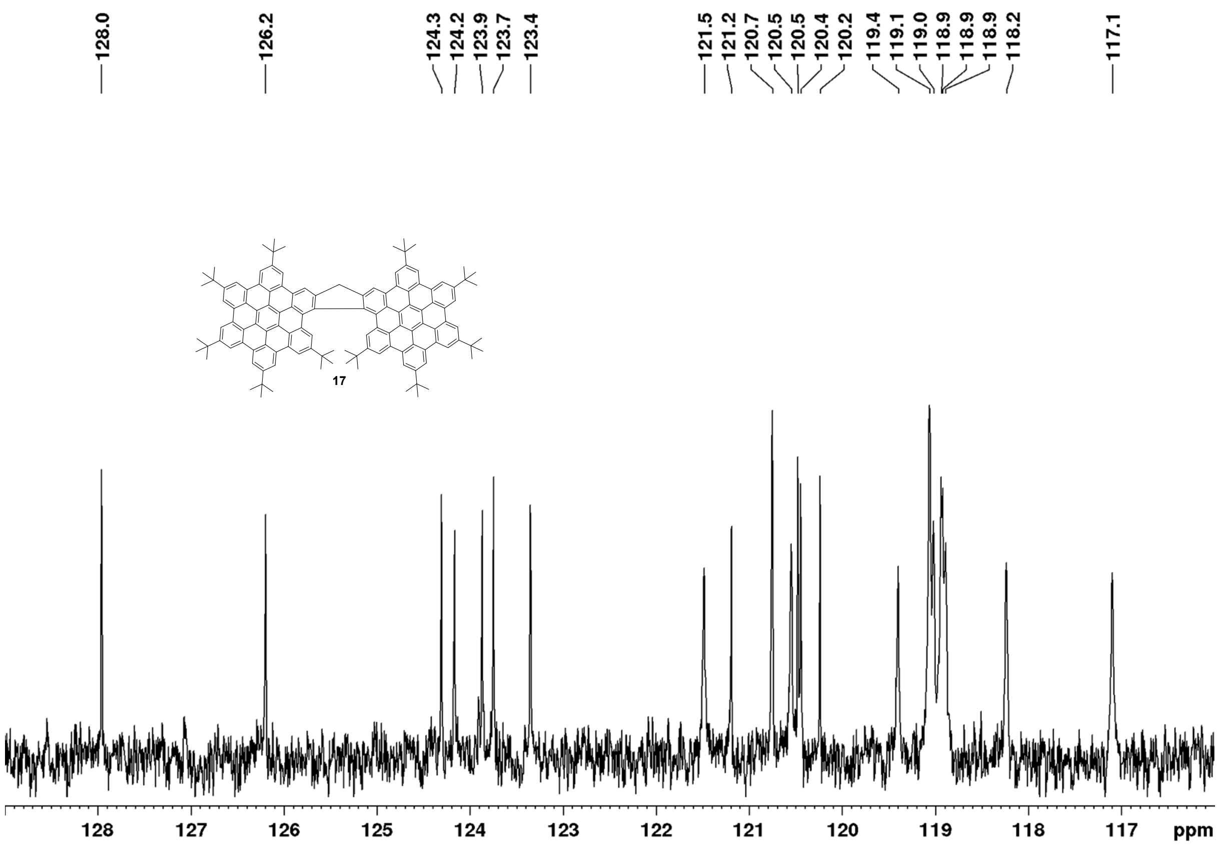

Figure S 144. Cutout (aromatic region) of the ^{13}C NMR spectrum of **17** ($CDCl_3$, 100 MHz, rt.).

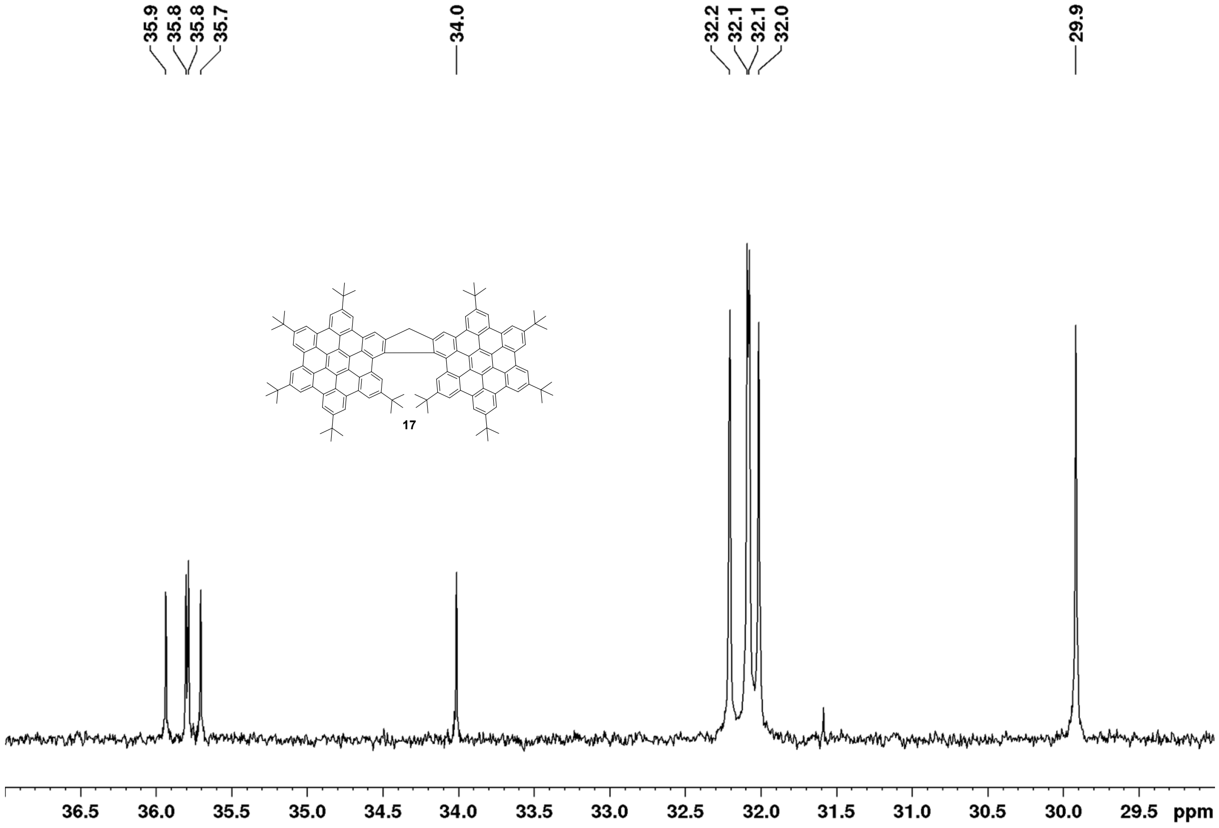

Figure S 145. Cutout (aliphatic region) of the ^{13}C NMR spectrum of **17** ($CDCl_3$, 100 MHz, rt.).

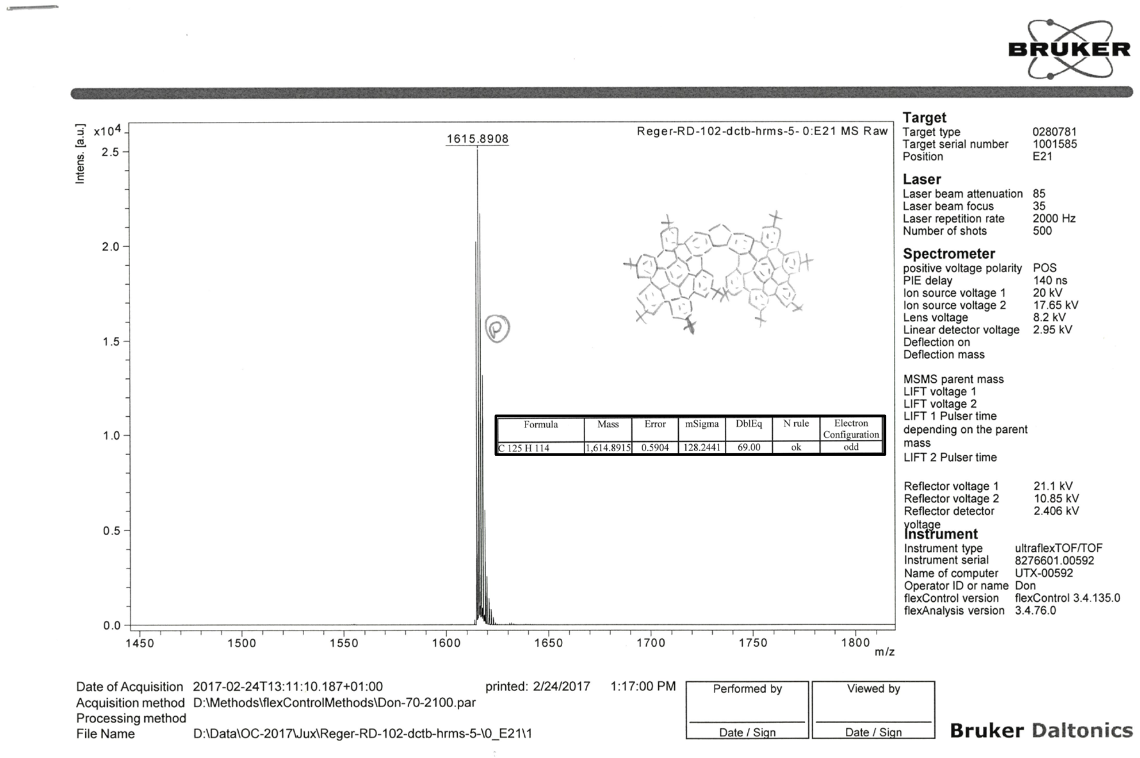

Figure S 146. HRMS-data (MALDI) of **17** (dctb). Insert: Calculated mass.

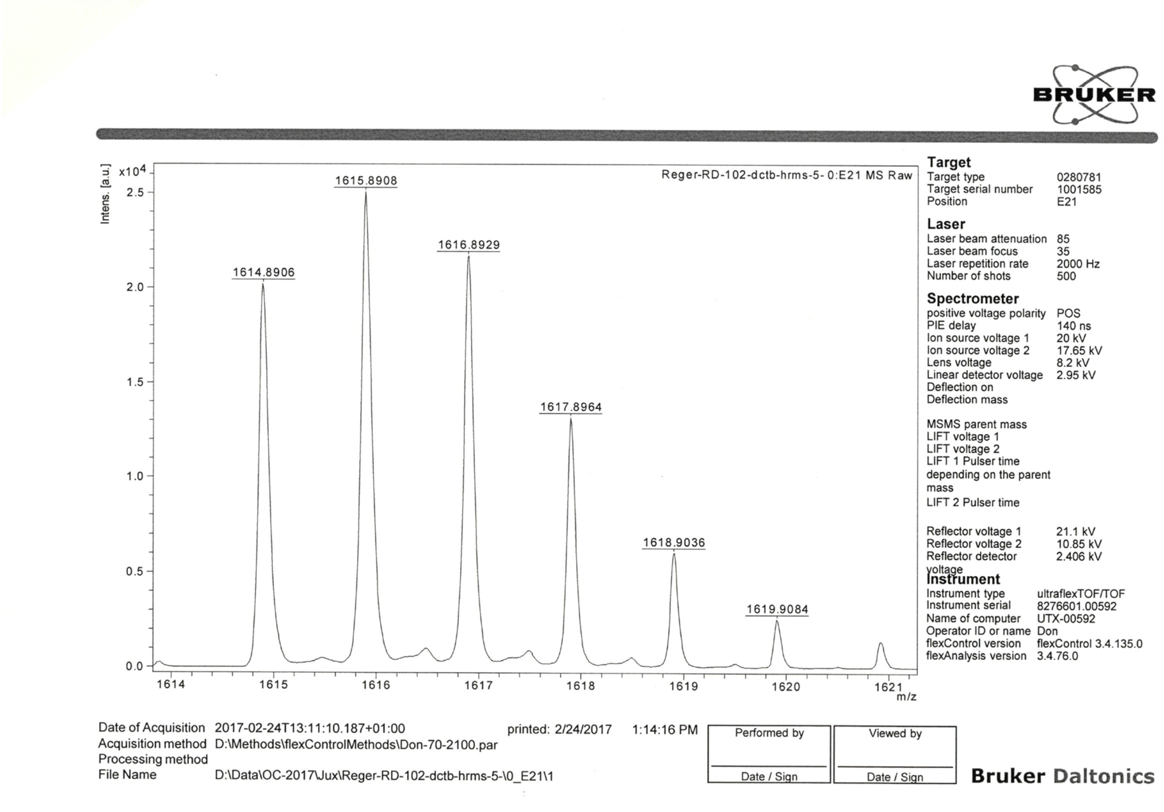

Figure S 147. HRMS-data (MALDI) of **17** zoom on product peak (dctb).

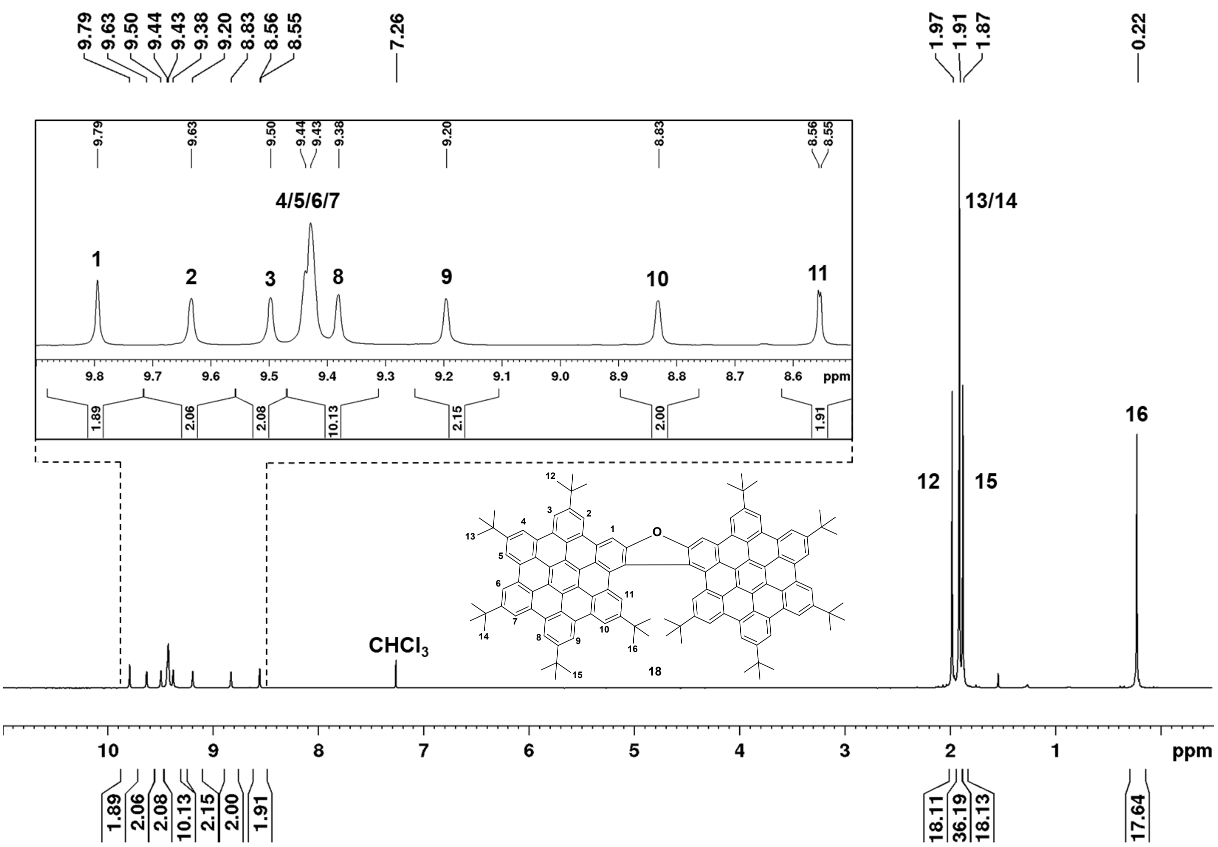

Figure S 148. ^{1}H NMR spectrum of **18** ($CDCl_3$, 400 MHz, rt.).

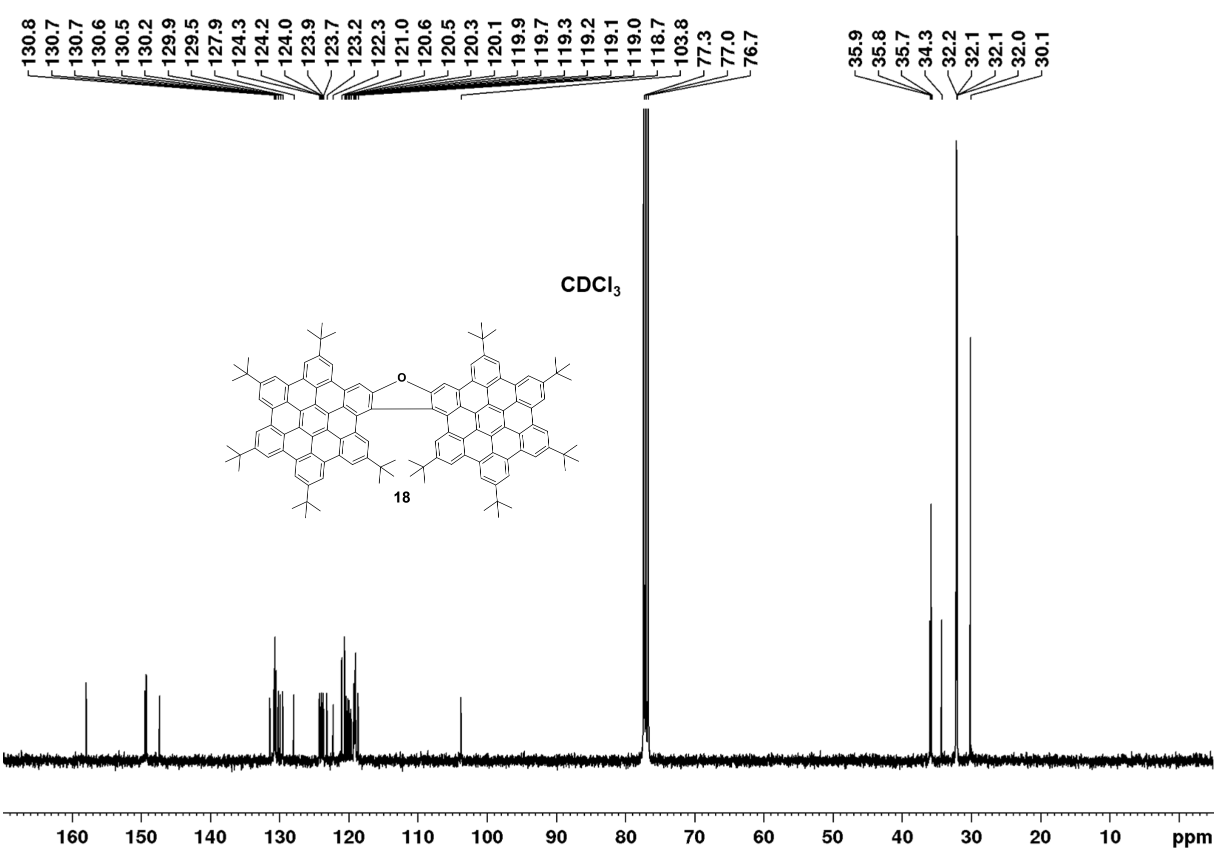

Figure S 149. Overview of the ^{13}C NMR spectrum of **18** ($CDCl_3$, 100 MHz, rt.).

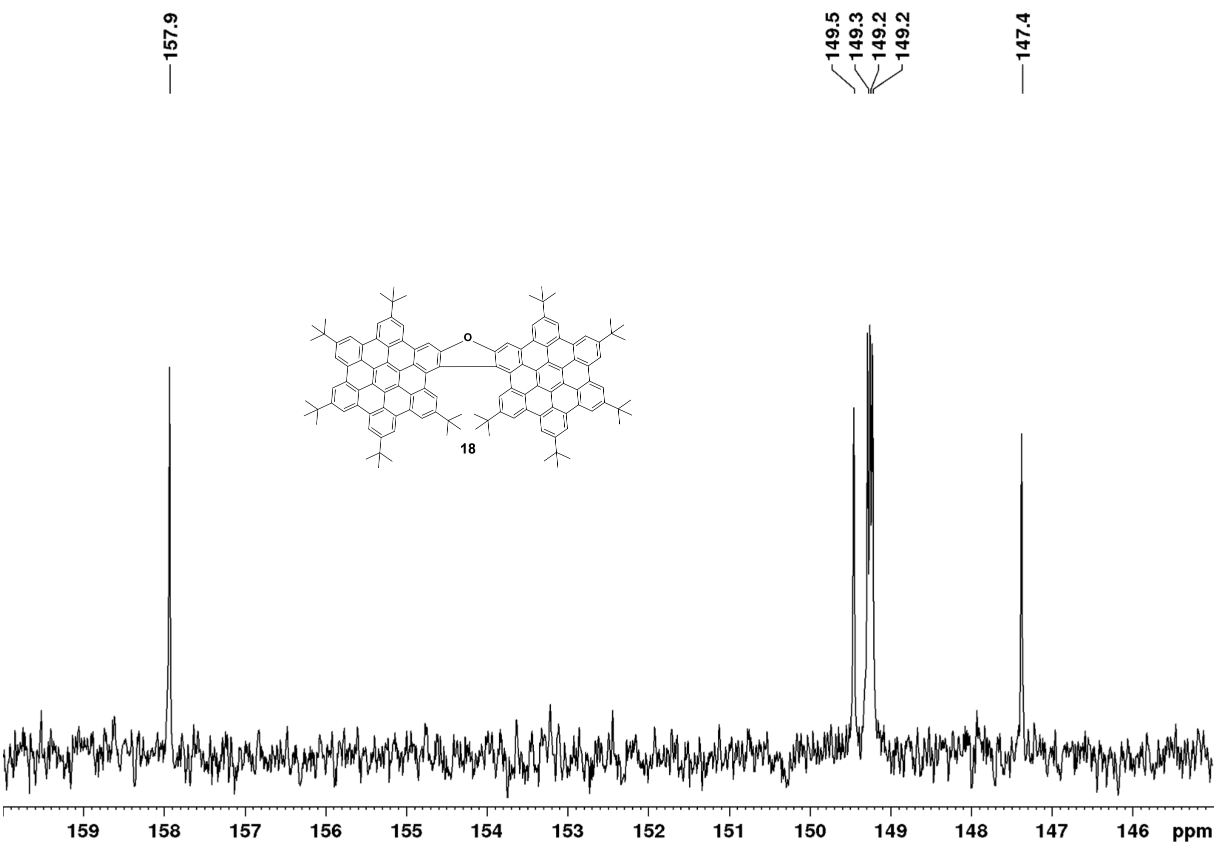

Figure S 150. Cutout (aromatic region) of the ^{13}C NMR spectrum of **18** ($CDCl_3$, 100 MHz, rt.).

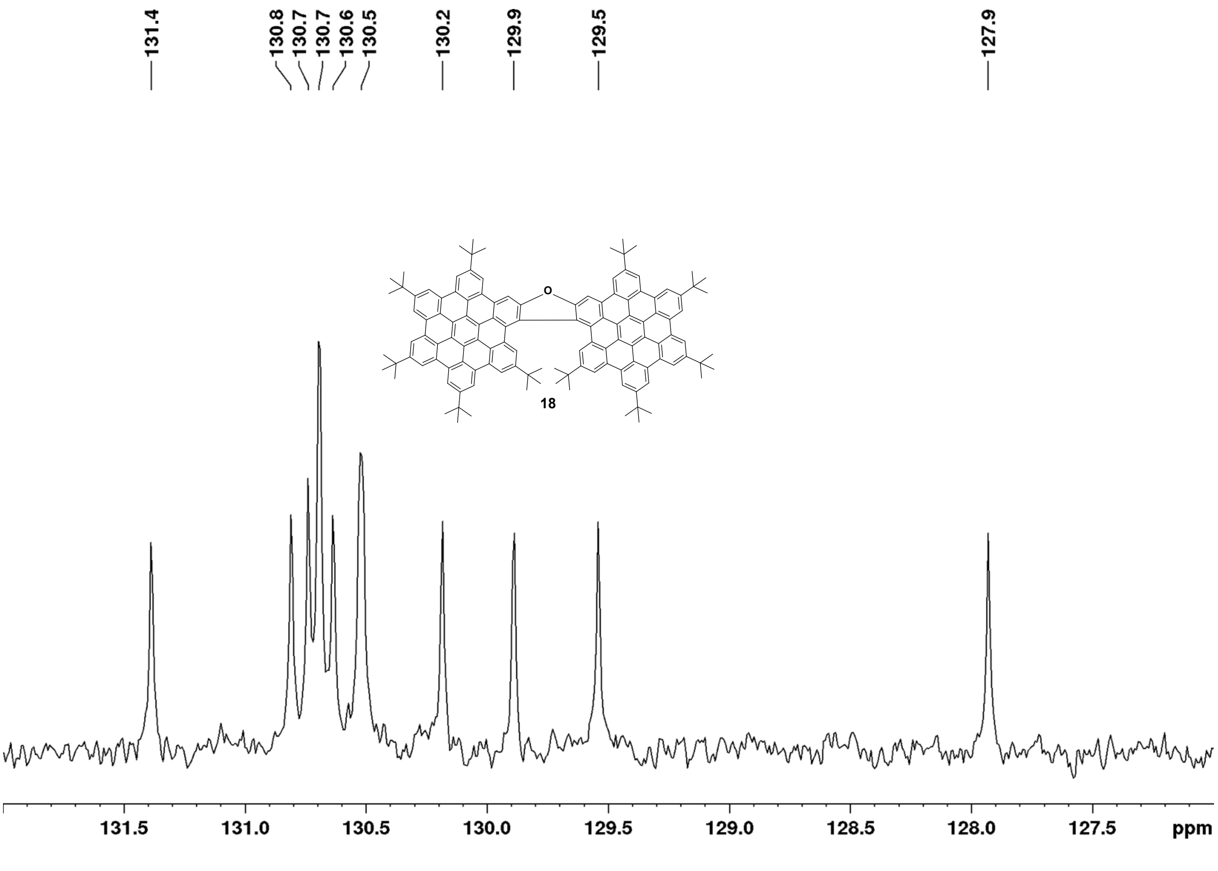

Figure S 151. Cutout (aromatic region) of the ^{13}C NMR spectrum of **18** ($CDCl_3$, 100 MHz, rt.).

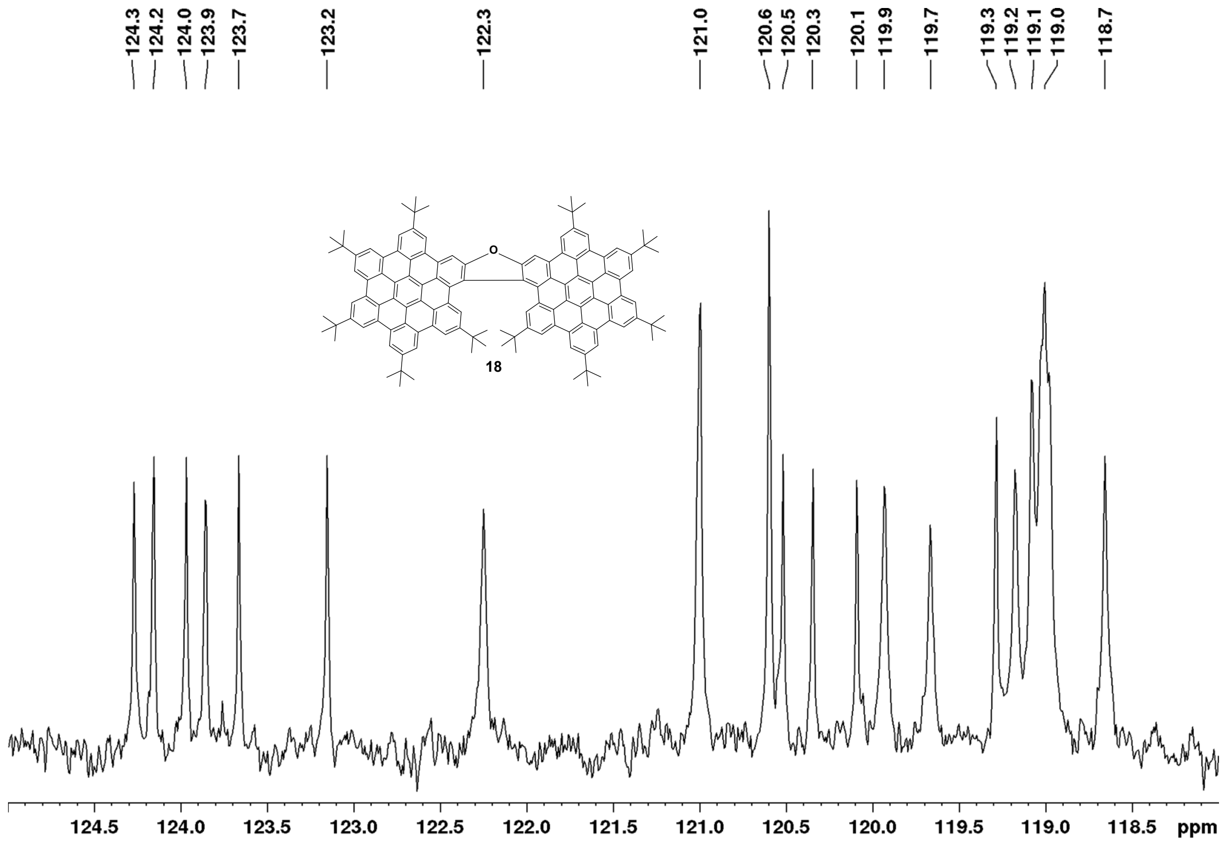

Figure S 152. Cutout (aromatic region) of the ^{13}C NMR spectrum of **18** ($CDCl_3$, 100 MHz, rt.).

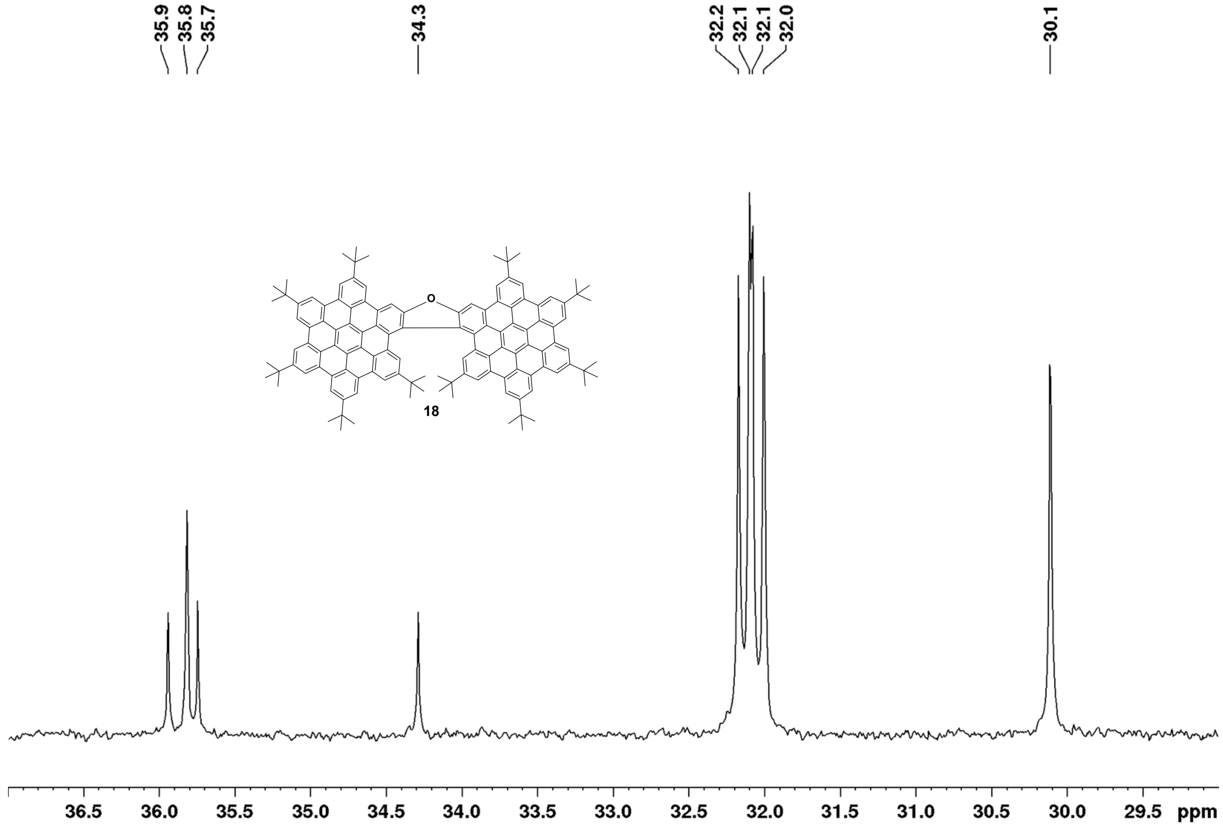

Figure S 153. Cutout (aliphatic region) of the ^{13}C NMR spectrum of **18** ($CDCl_3$, 100 MHz, rt.).

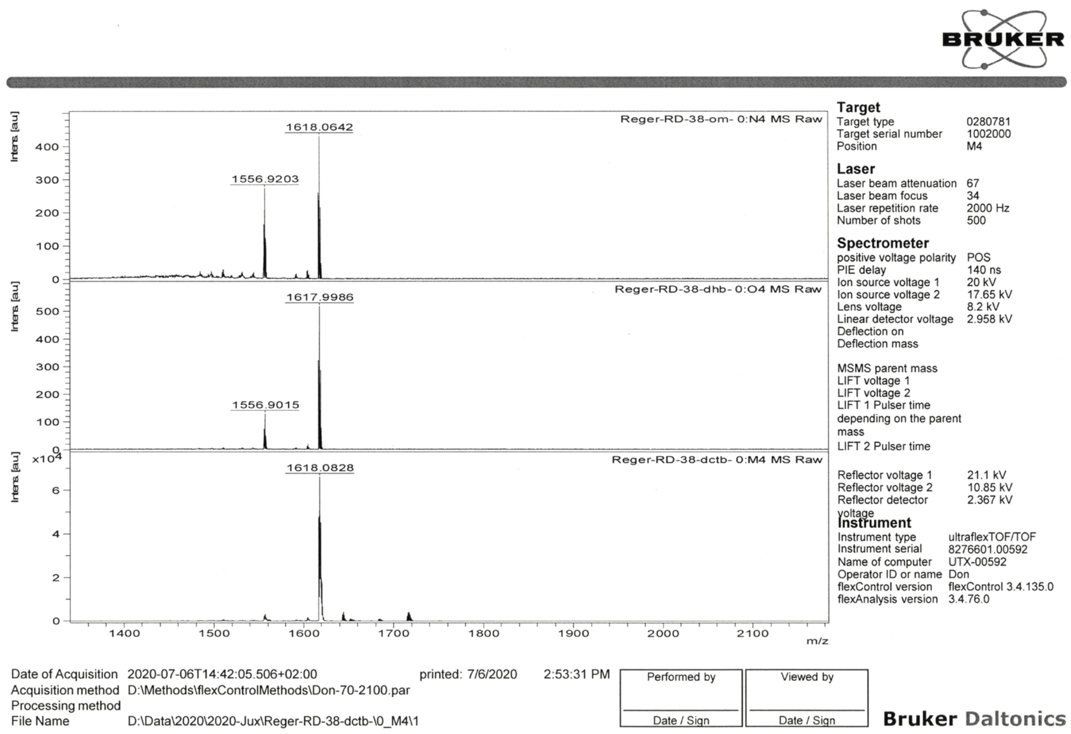

Figure S 154. MS-data (MALDI) of **18** (top: without matrix; middle: dhb; bottom: dctb).

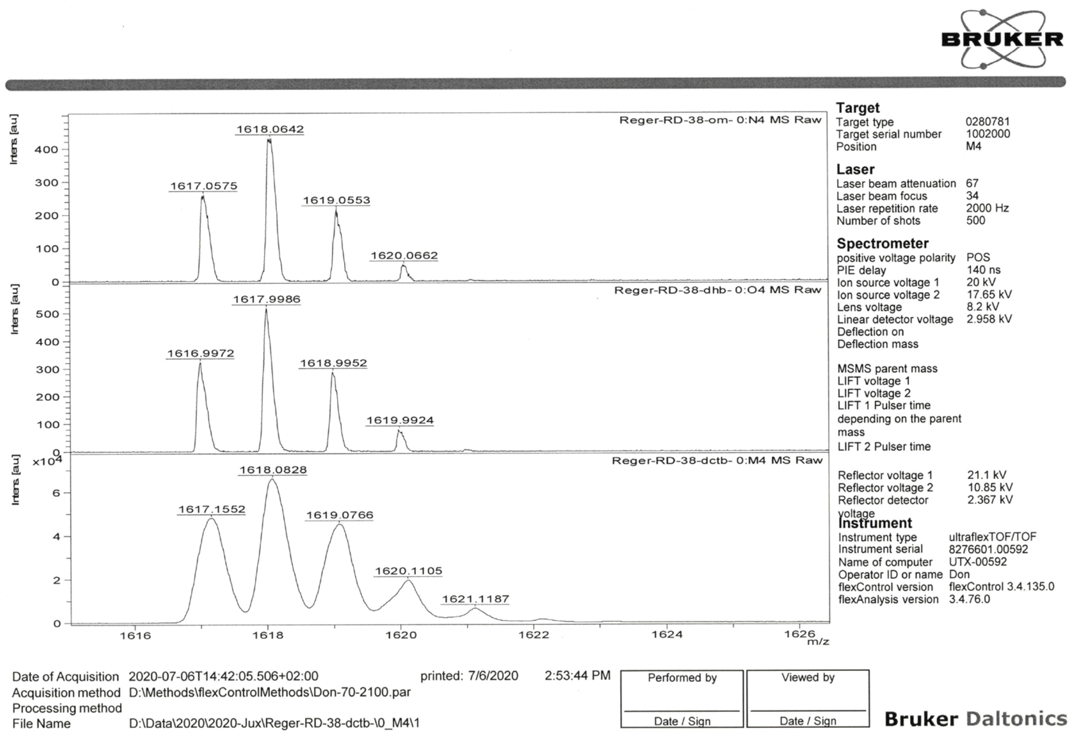

Figure S 155. MS-data (MALDI) of **18** zoom on product peak (top: without matrix; middle: dhb; bottom: dctb).

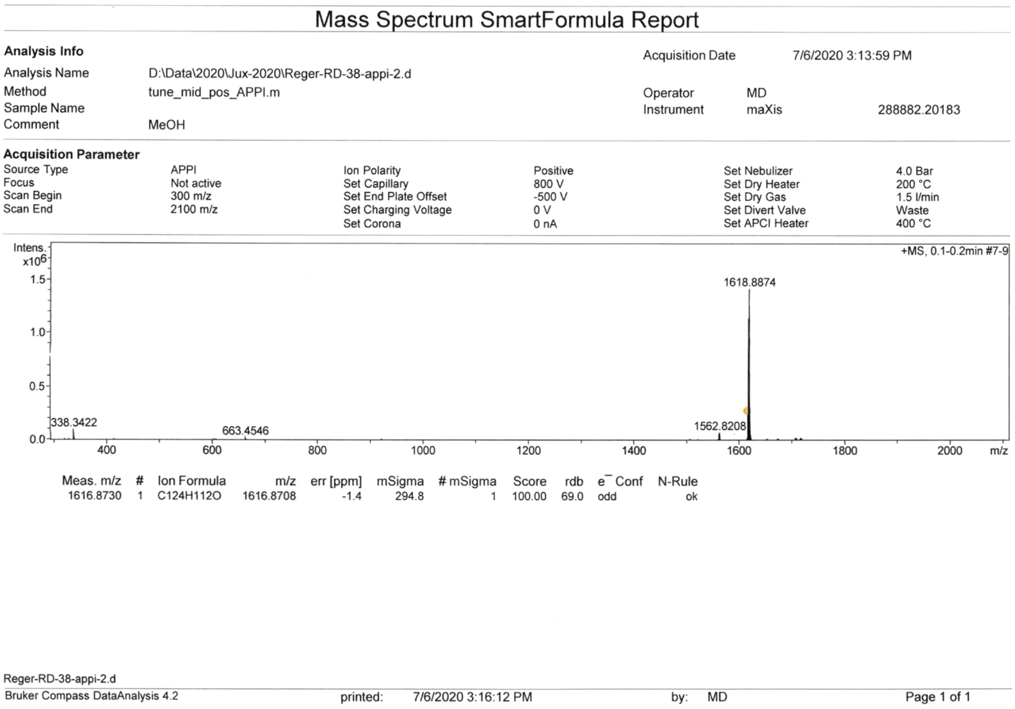

Figure S 156. HRMS data (APPI; toluene) of **18** (overview).

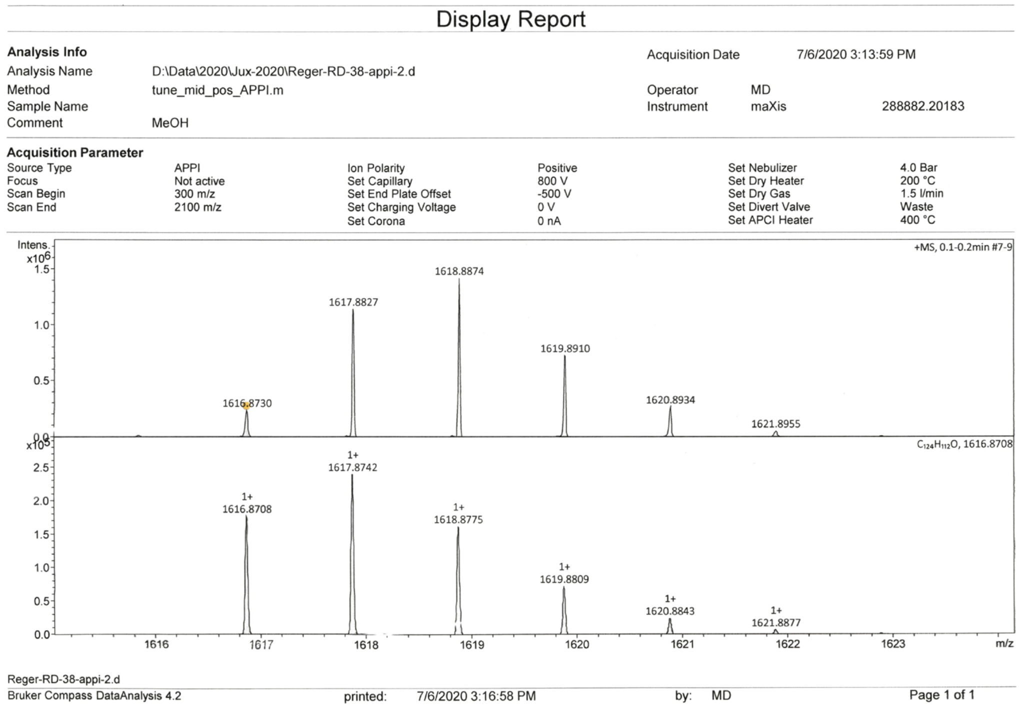

Figure S 157. HRMS data (APPI; toluene) of **18** (top: measured; bottom: calculated).

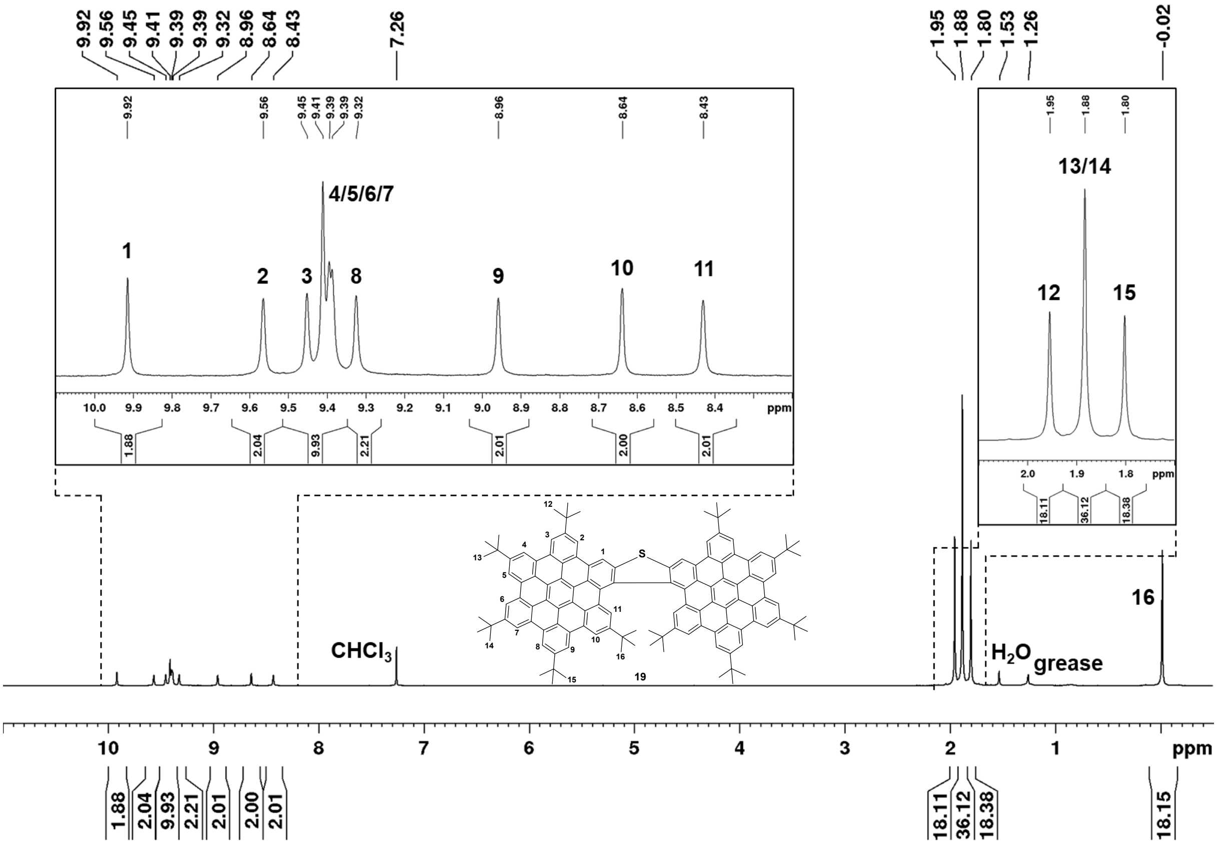

Figure S 158. ^{1}H NMR spectrum of **19** ($CDCl_3$, 400 MHz, rt.).

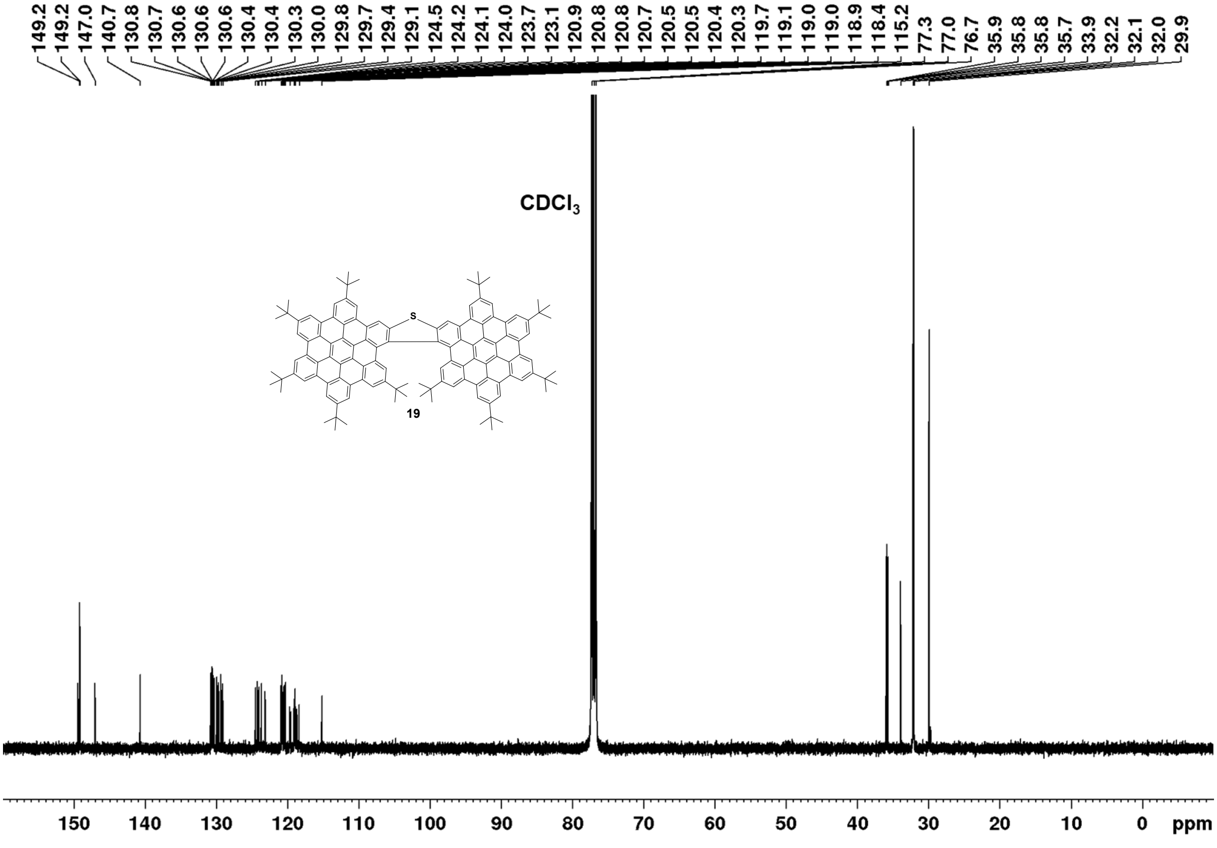

Figure S 159. Overview of the ^{13}C NMR spectrum of **19** ($CDCl_3$, 100 MHz, rt.).

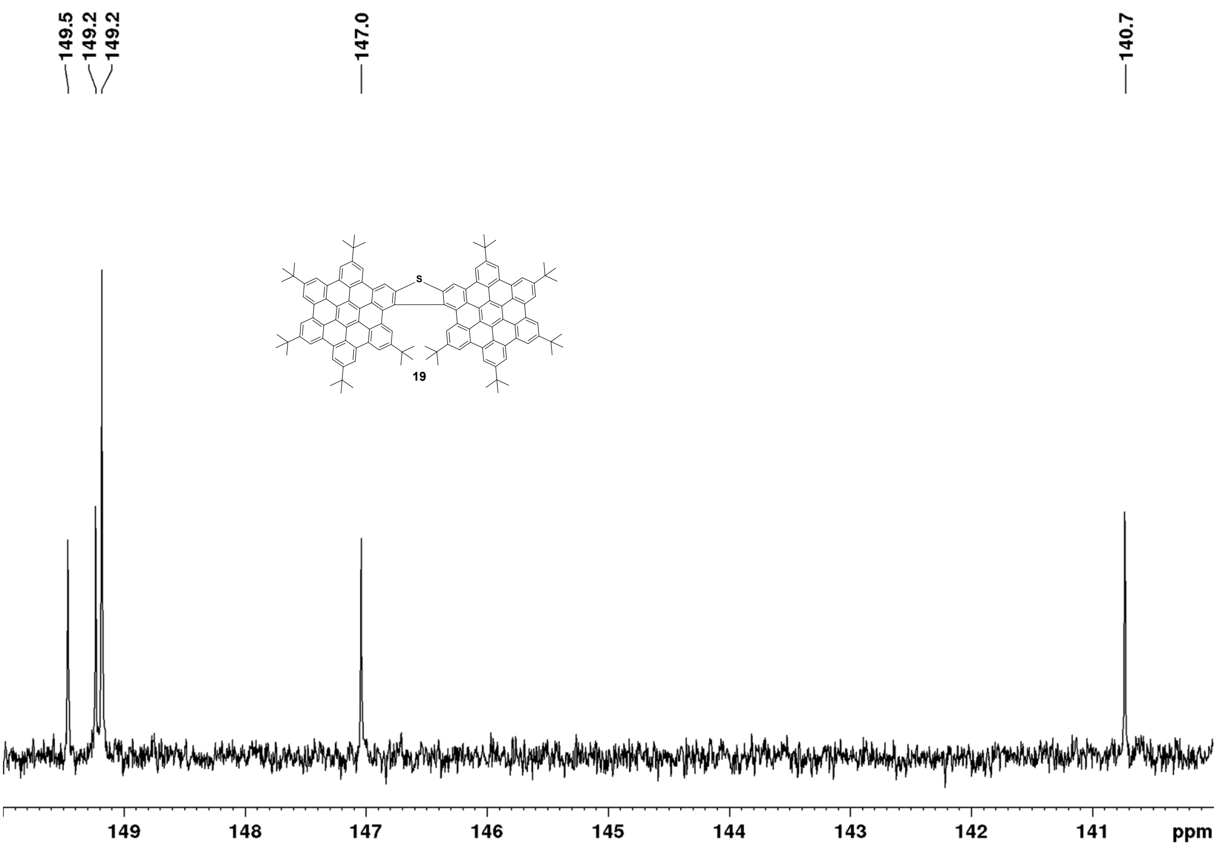

Figure S 160. Cutout (aromatic region) of the ^{13}C NMR spectrum of **19** ($CDCl_3$, 100 MHz, rt.).

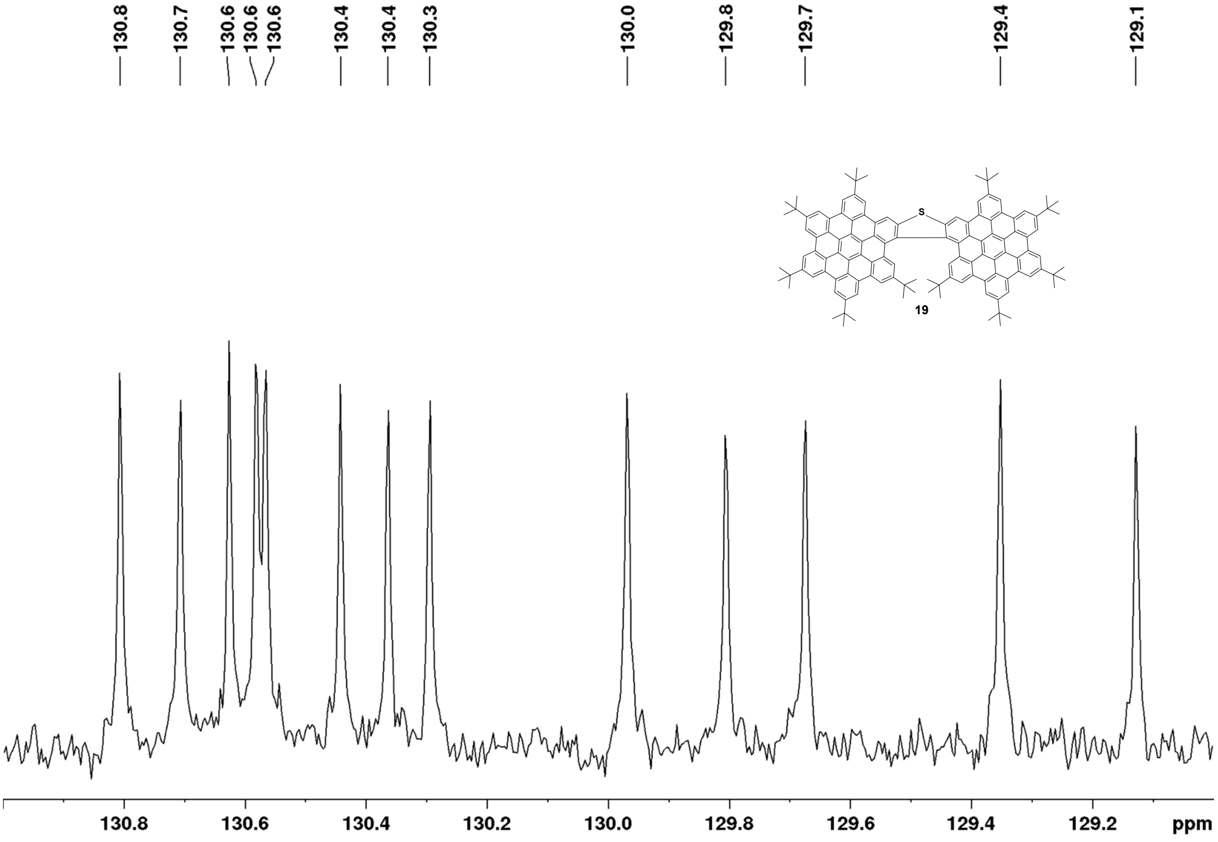

Figure S 161. Cutout (aromatic region) of the ^{13}C NMR spectrum of **19** ($CDCl_3$, 100 MHz, rt.).

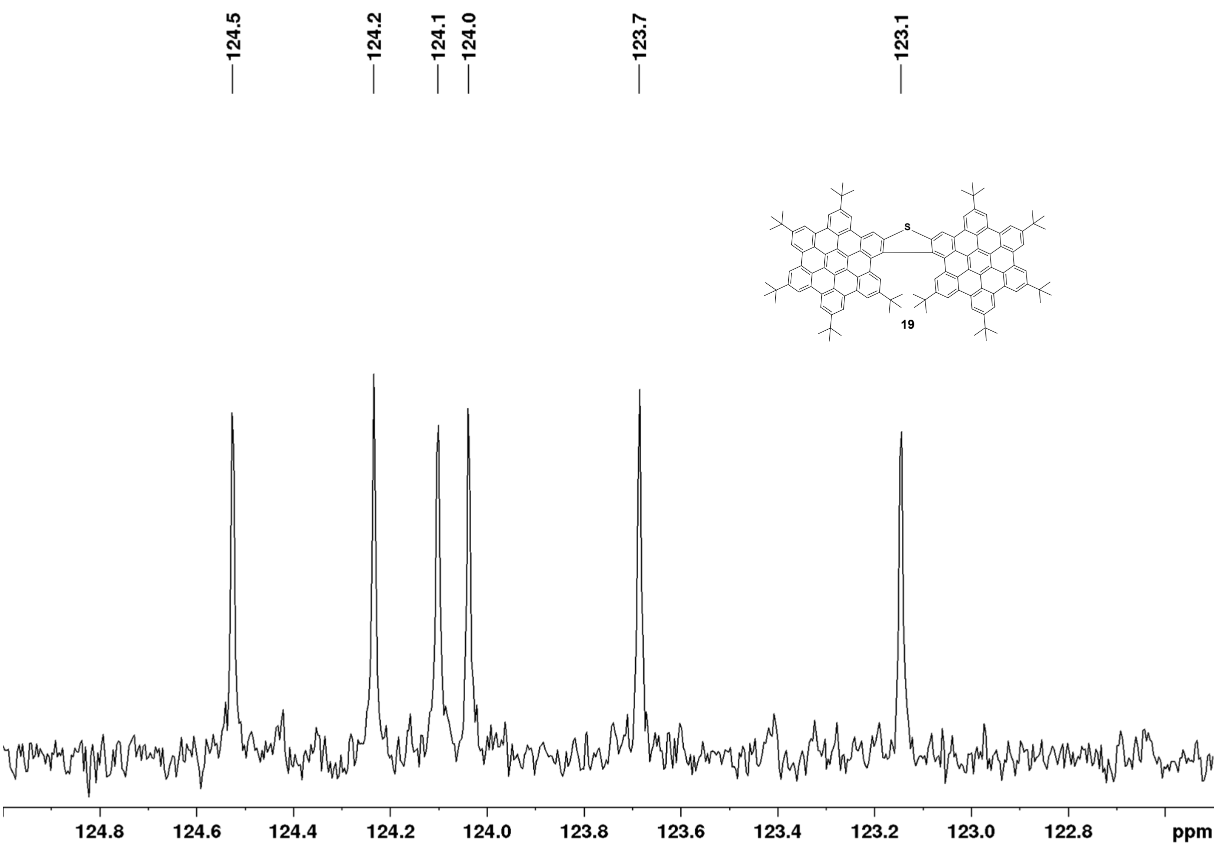

Figure S 162. Cutout (aromatic region) of the ^{13}C NMR spectrum of **19** ($CDCl_3$, 100 MHz, rt.).

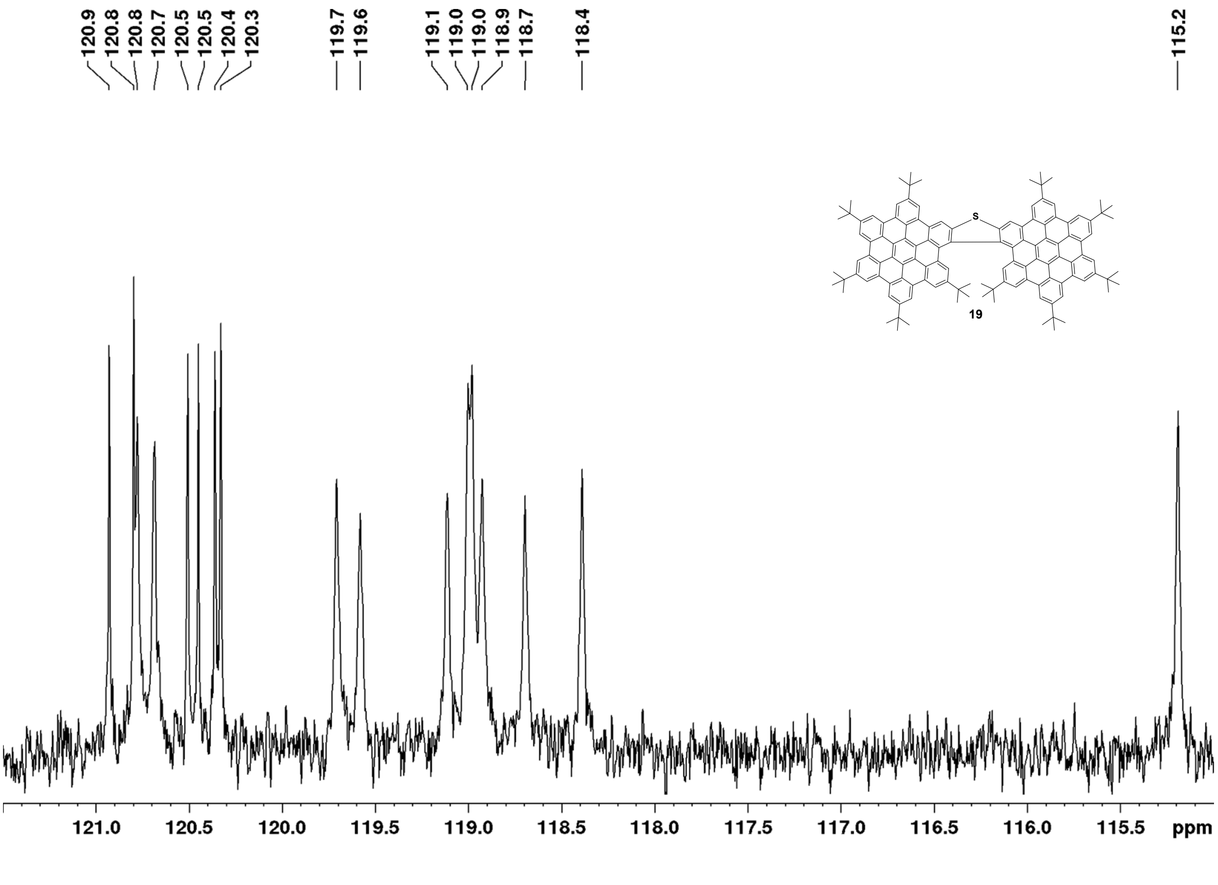

Figure S 163. Cutout (aromatic region) of the ^{13}C NMR spectrum of **19** ($CDCl_3$, 100 MHz, rt.).

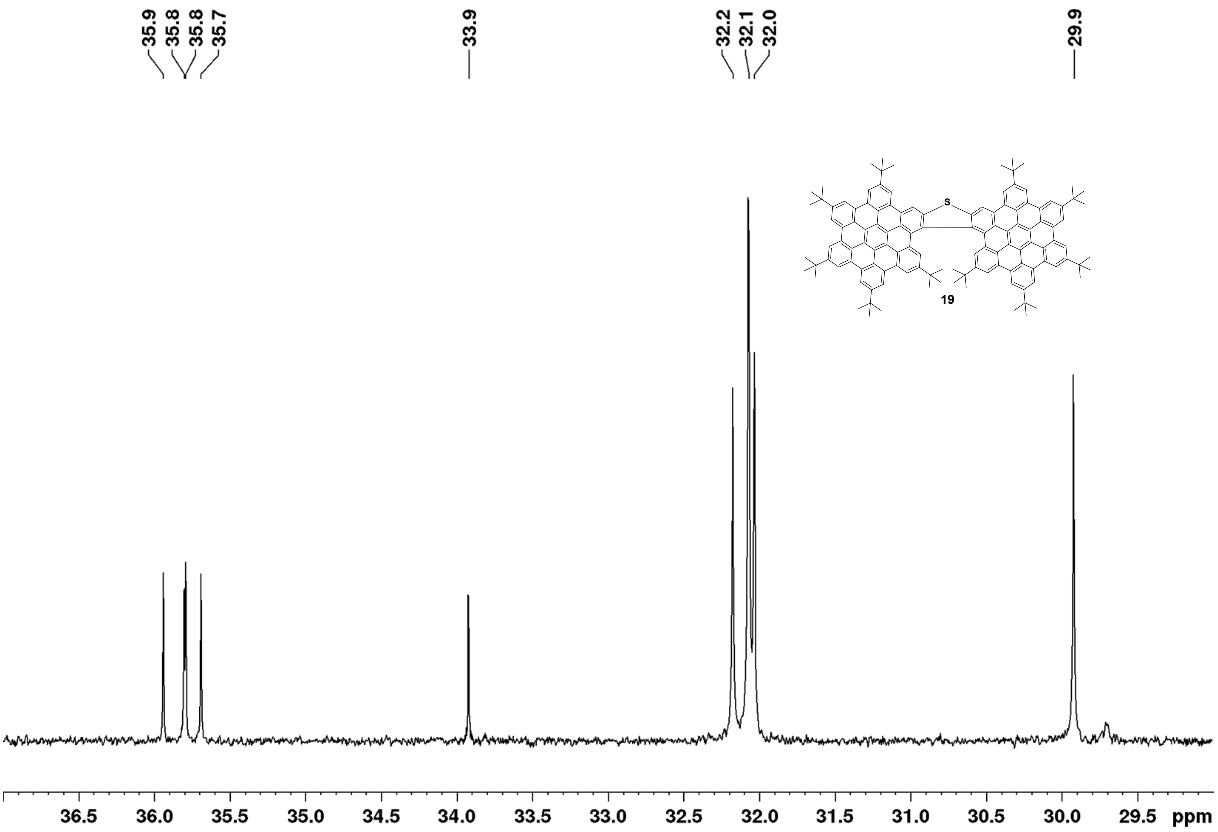

Figure S 164. Cutout (aliphatic region) of the ^{13}C NMR spectrum of **19** ($CDCl_3$, 100 MHz, rt.).

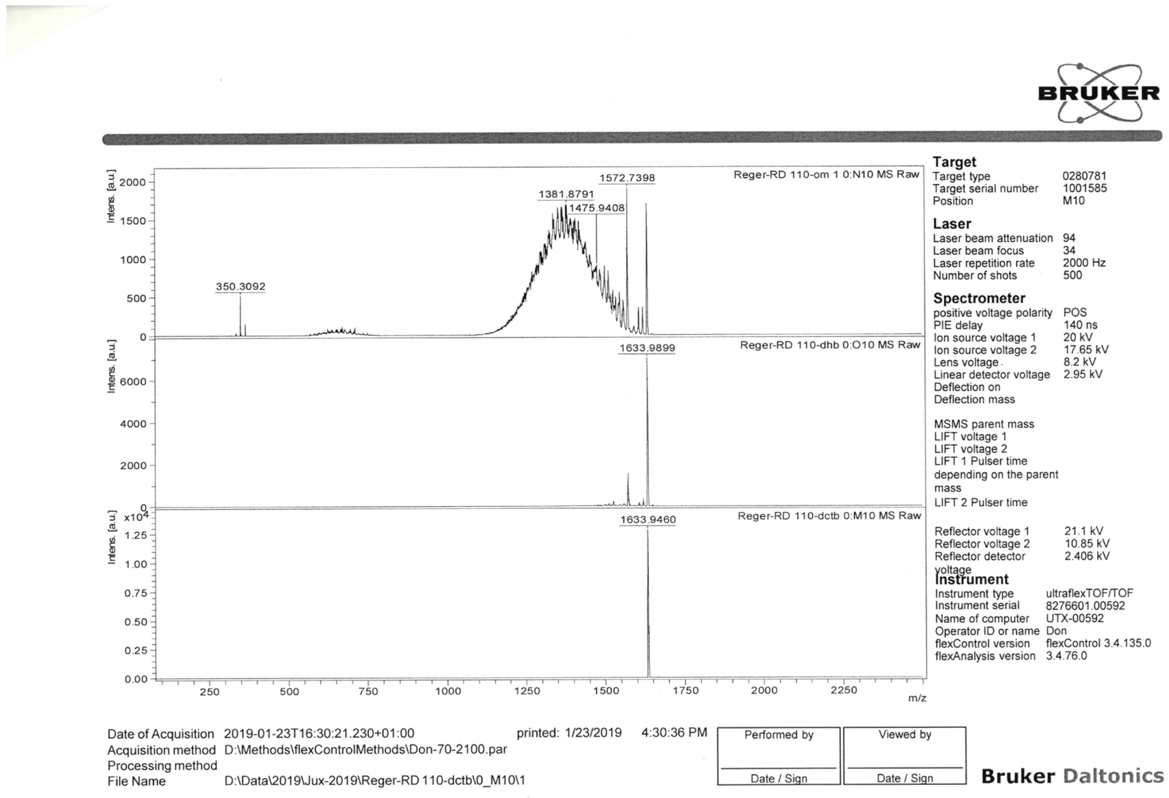

Figure S 165. MS-data (MALDI) of **19** (top: without matrix; middle: dhb; bottom: dctb).

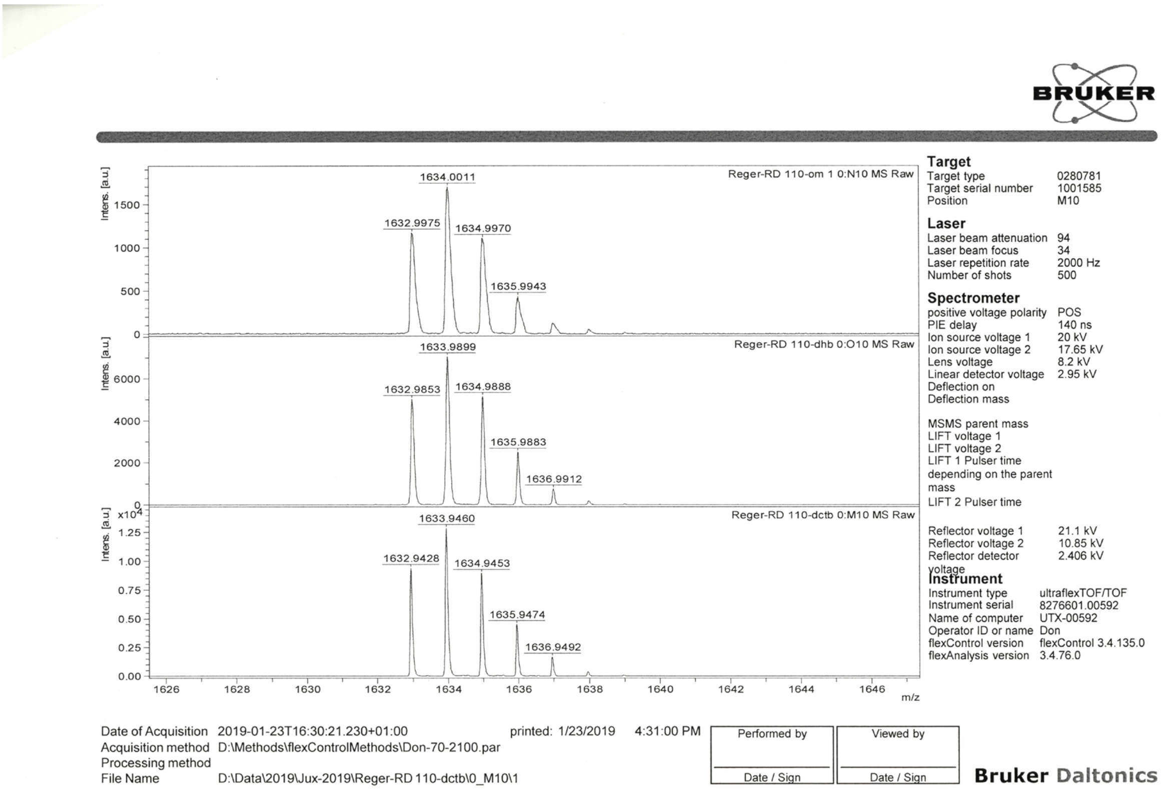

Figure S 166. MS-data (MALDI) of **19** zoom on product peak (top: without matrix; middle: dhb; bottom: dctb).

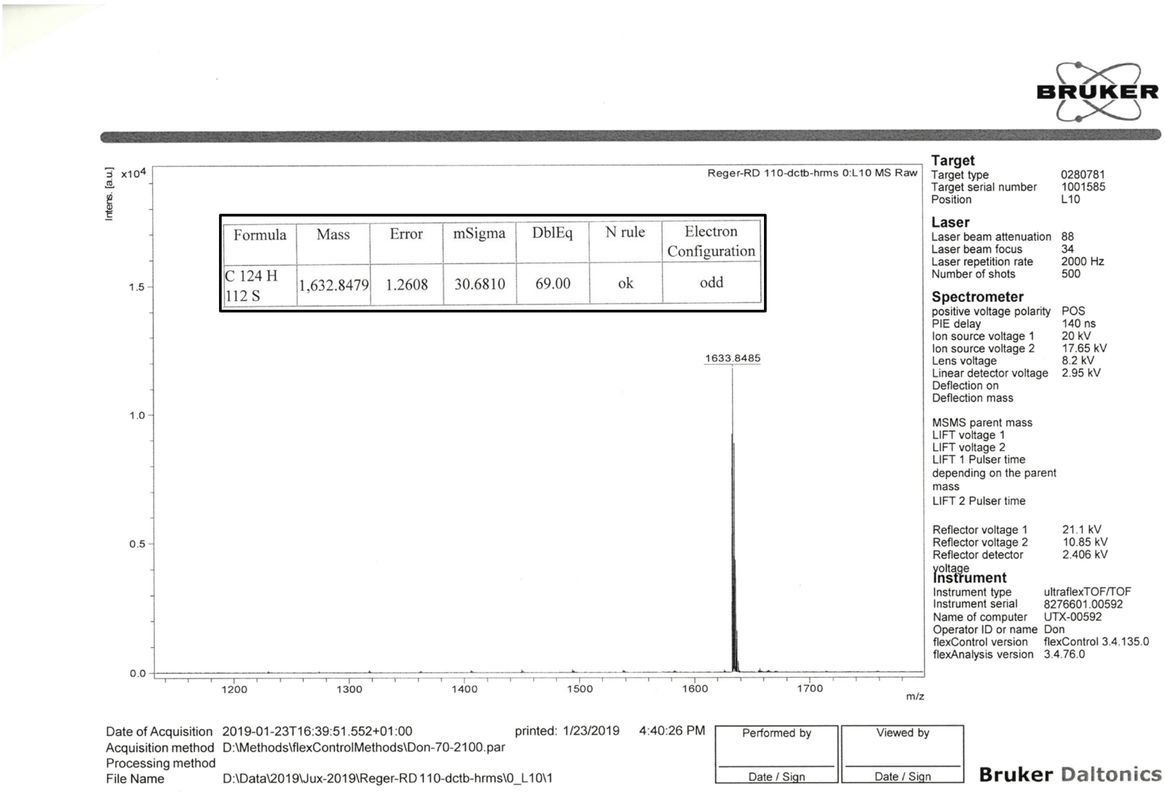

Figure S 167. HRMS-data (MALDI, dctb) of **19** (overview). Insert: Calculated mass.

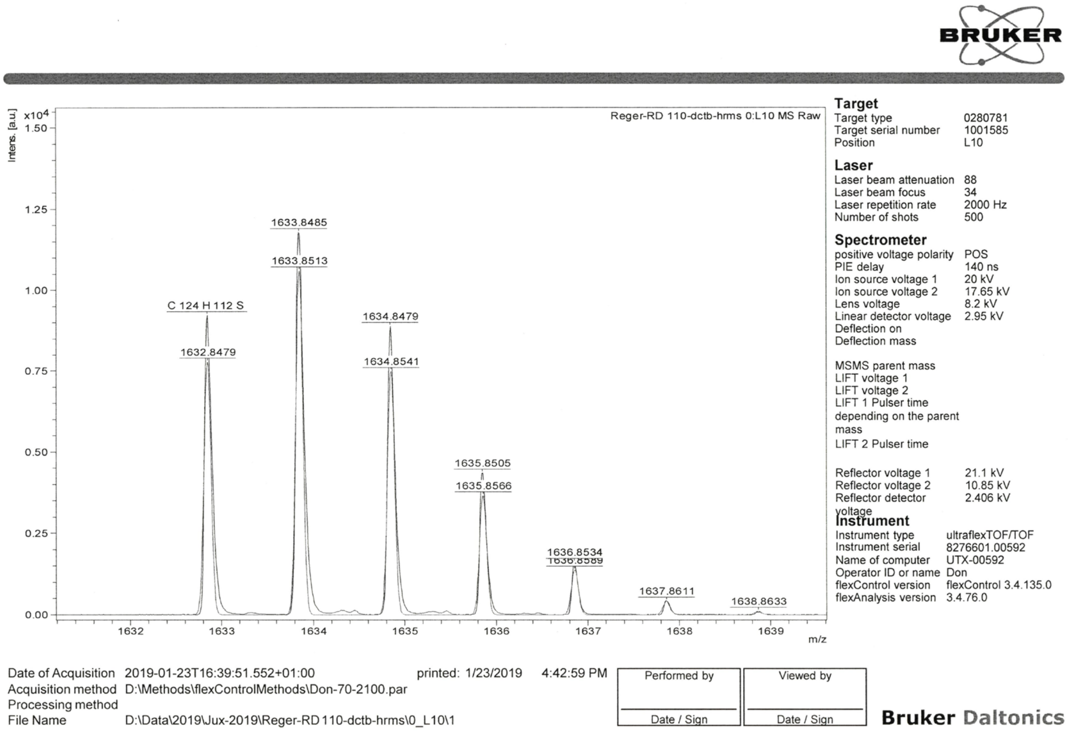

Figure S 168. HRMS-data (MALDI, dctb) of **19** (grey line: calculated; black line: measured).

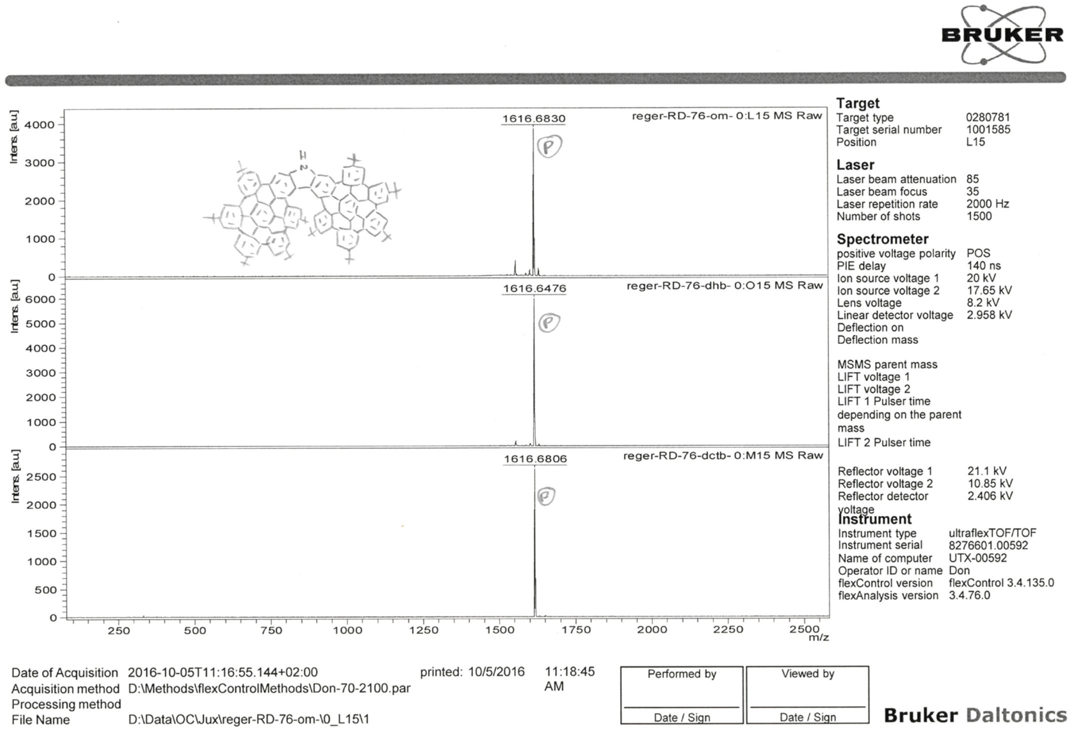

Figure S 169. MS-data (MALDI) of **20** (top: without matrix; middle: dhb; bottom: dctb).

BRUKER

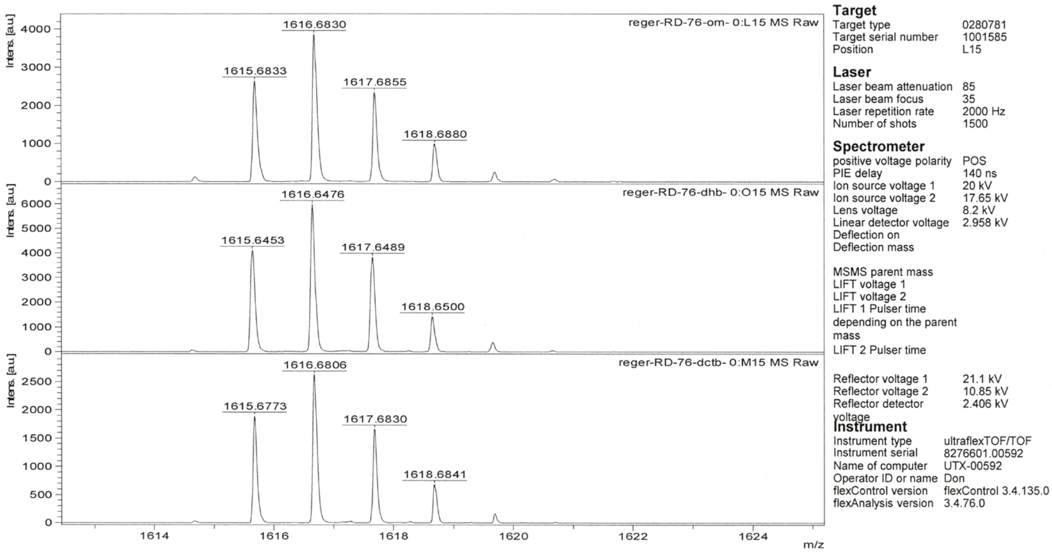

Target

Target type	0280781
Target serial number	1001585
Position	L15

Laser

Laser beam attenuation	85
Laser beam focus	35
Laser repetition rate	2000 Hz
Number of shots	1500

Spectrometer

positive voltage polarity	POS
PIE delay	140 ns
Ion source voltage 1	20 kV
Ion source voltage 2	17.65 kV
Lens voltage	8.2 kV
Linear detector voltage	2.958 kV
Deflection on	
Deflection mass	
MSMS parent mass	
LIFT voltage 1	
LIFT voltage 2	
LIFT 1 Pulser time depending on the parent mass	
LIFT 2 Pulser time	
Reflector voltage 1	21.1 kV
Reflector voltage 2	10.85 kV
Reflector detector voltage	2.406 kV

Instrument

Instrument type	ultraflexTOF/TOF
Instrument serial	8276601.00592
Name of computer	UTX-00592
Operator ID or name	Don
flexControl version	flexControl 3.4.135.0
flexAnalysis version	3.4.76.0

Date of Acquisition 2016-10-05T11:16:55.144+02:00 printed: 10/5/2016 11:18:57 AM
Acquisition method D:\Methods\flexControlMethods\Don-70-2100.par
Processing method
File Name D:\Data\OC\Jux\reger-RD-76-om-\0_L15\1

Performed by — Date / Sign | Viewed by — Date / Sign

Bruker Daltonics

Figure S 170. MS-data (MALDI) of **20** zoom on product peak (top: without matrix; middle: dhb; bottom: dctb).

Mass Spectrum SmartFormula Report

Analysis Info

Analysis Name	D:\Data\Tykwinski-2016-\Reger-RD-76-appi000001.d	Acquisition Date	10/5/2016 1:53:56 PM
Method	tune_mid_pos_APPI.m	Operator	MD
Sample Name		Instrument	maXis 288882.20183
Comment	Tol		

Acquisition Parameter

Source Type	APPI	Ion Polarity	Positive	Set Nebulizer	2.5 Bar
Focus	Not active	Set Capillary	700 V	Set Dry Heater	200 °C
Scan Begin	300 m/z	Set End Plate Offset	-500 V	Set Dry Gas	1.5 l/min
Scan End	2000 m/z	Set Charging Voltage	0 V	Set Divert Valve	Waste
		Set Corona	0 nA	Set APCI Heater	400 °C

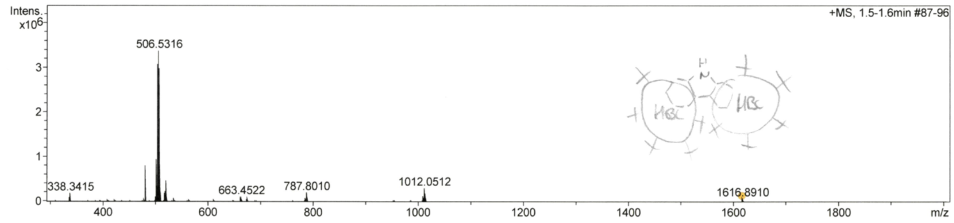

Meas. m/z	#	Ion Formula	m/z	err [ppm]	mSigma	# mSigma	Score	rdb	e⁻ Conf	N-Rule
1615.8851	1	C124H113N	1615.8868	1.0	114.6	1	100.00	69.0	odd	ok
	2	C121H115O3	1615.8841	-0.7	124.5	2	82.79	64.5	even	ok
	3	C104O23	1615.8825	-1.6	182.8	3	0.52	105.0	odd	ok
	4	C97H4O28	1615.8884	2.0	210.3	4	0.03	96.0	odd	ok
	5	C82H10NO38	1615.8875	1.5	256.4	5	0.00	78.5	even	ok

Reger-RD-76-appi000001.d
Bruker Compass DataAnalysis 4.2 printed: 10/5/2016 1:58:06 PM by: MD Page 1 of 1

Figure S 171. HRMS data (APPI; toluene) of **20** (overview).

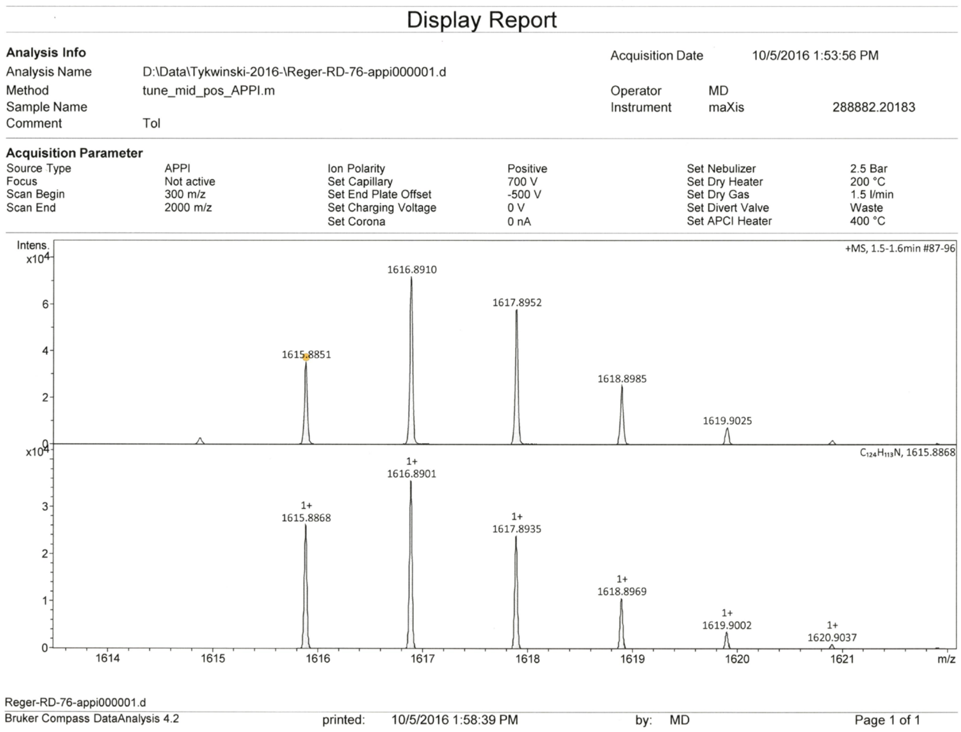

Figure S 172. HRMS data (APPI; toluene) of **20** (top: measured; bottom: calculated).

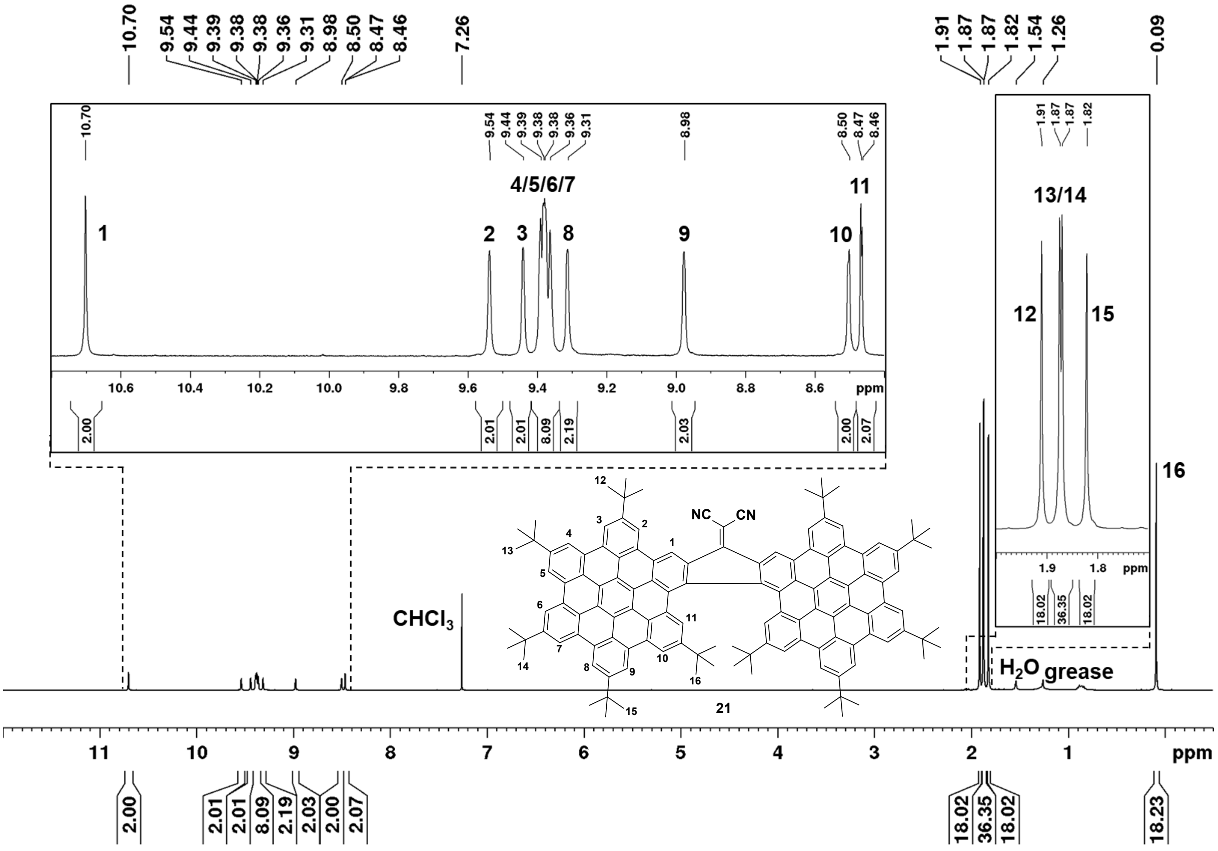

Figure S 173. ^{1}H NMR spectrum of **21** (CDCl₃, 400 MHz, rt.).

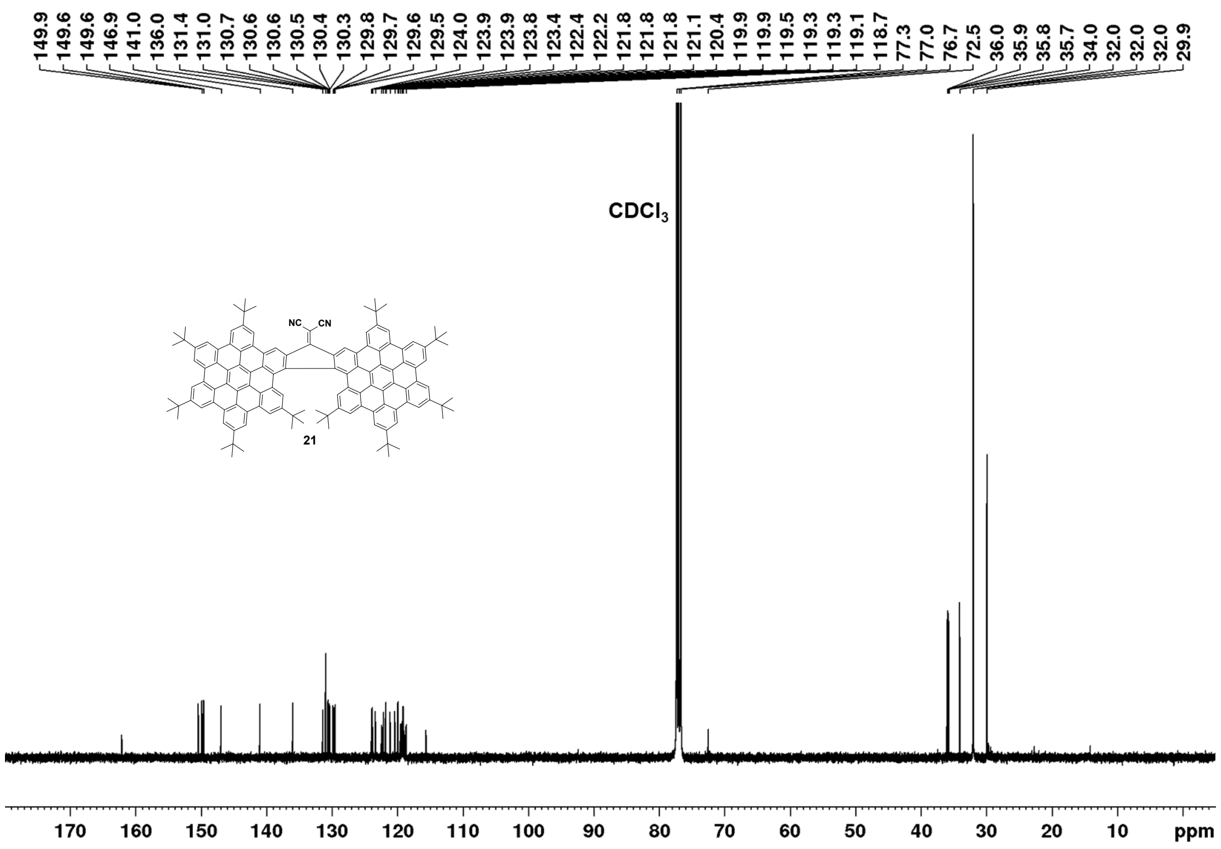

Figure S 174. Overview of the ^{13}C NMR spectrum of **21** ($CDCl_3$, 100 MHz, rt.).

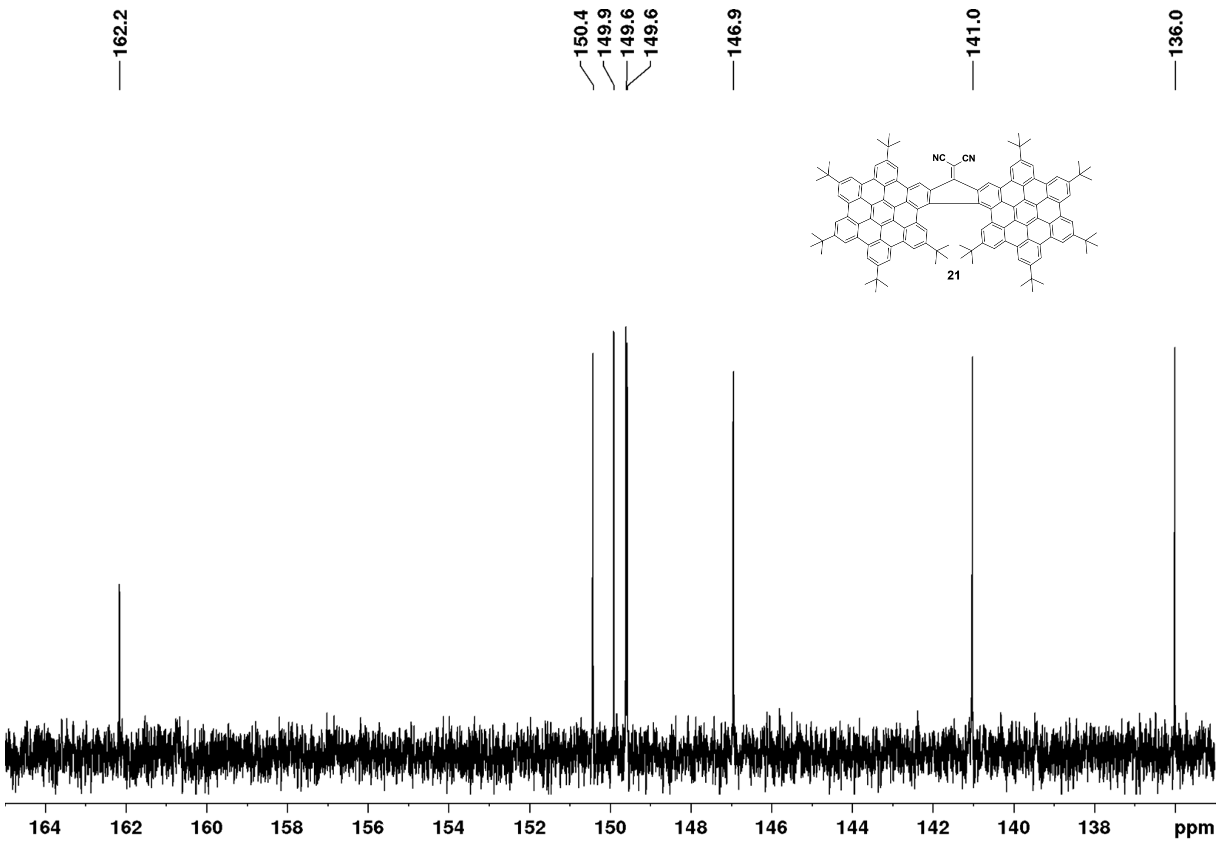

Figure S 175. Cutout (aromatic region) of the ^{13}C NMR spectrum of **21** ($CDCl_3$, 100 MHz, rt.).

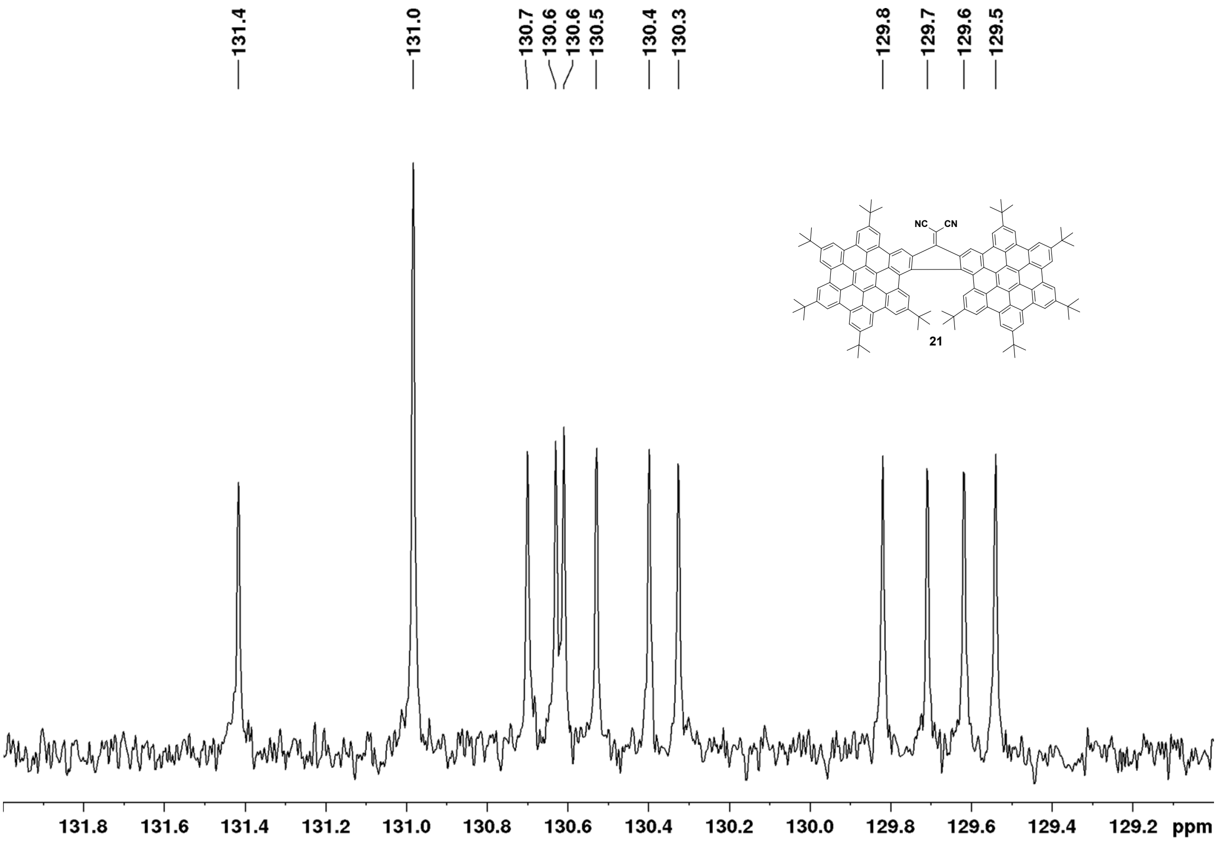

Figure S 176. Cutout (aromatic region) of the ^{13}C NMR spectrum of **21** ($CDCl_3$, 100 MHz, rt.).

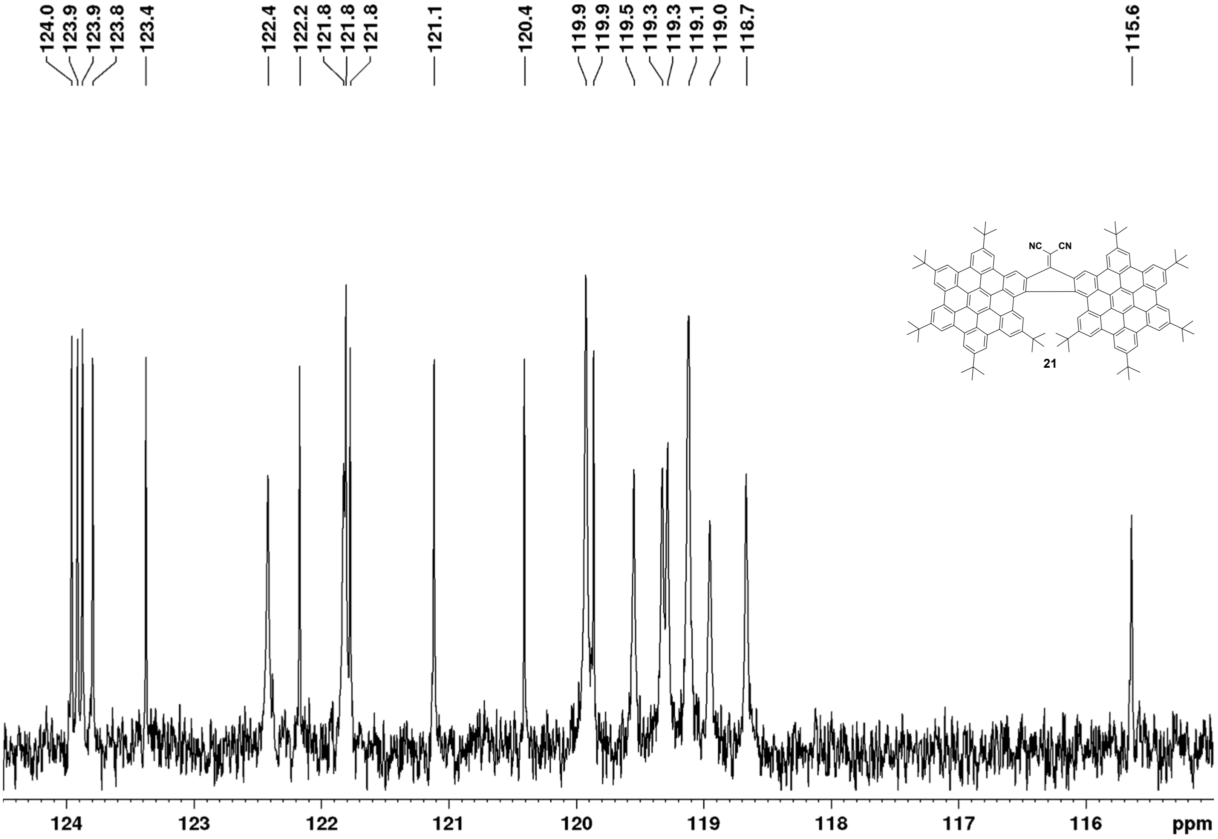

Figure S 177. Cutout (aromatic region) of the ^{13}C NMR spectrum of **21** ($CDCl_3$, 100 MHz, rt.).

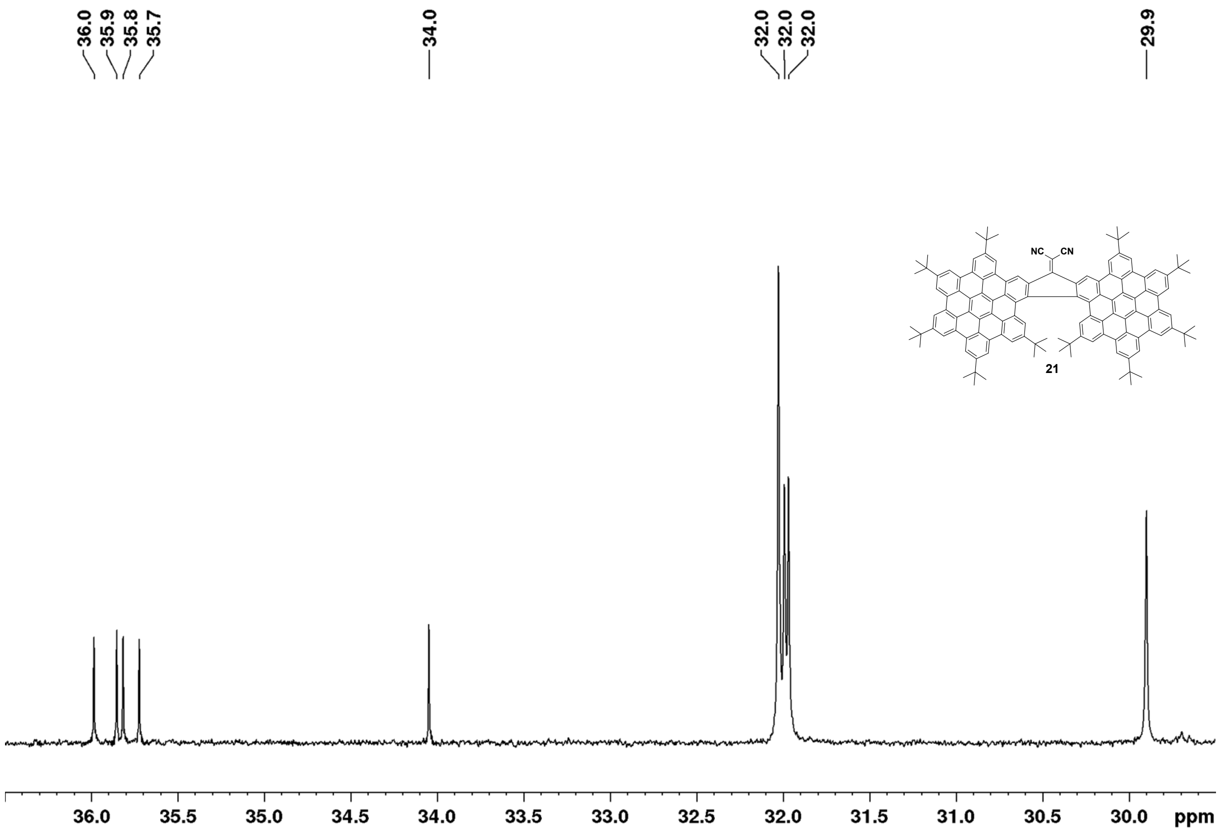

Figure S 178. Cutout (aliphatic region) of the ^{13}C NMR spectrum of **21** ($CDCl_3$, 100 MHz, rt.).

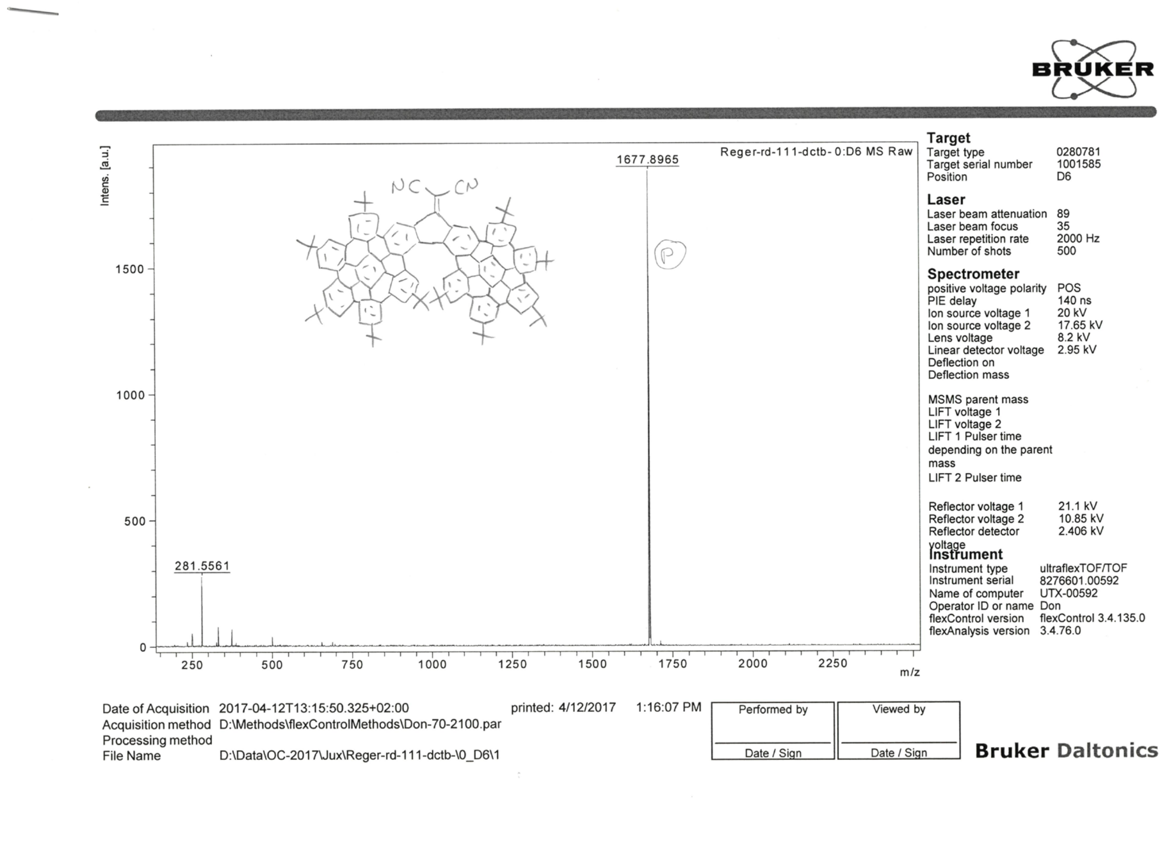

Figure S 179. MS-data (MALDI) of **21** (dctb).

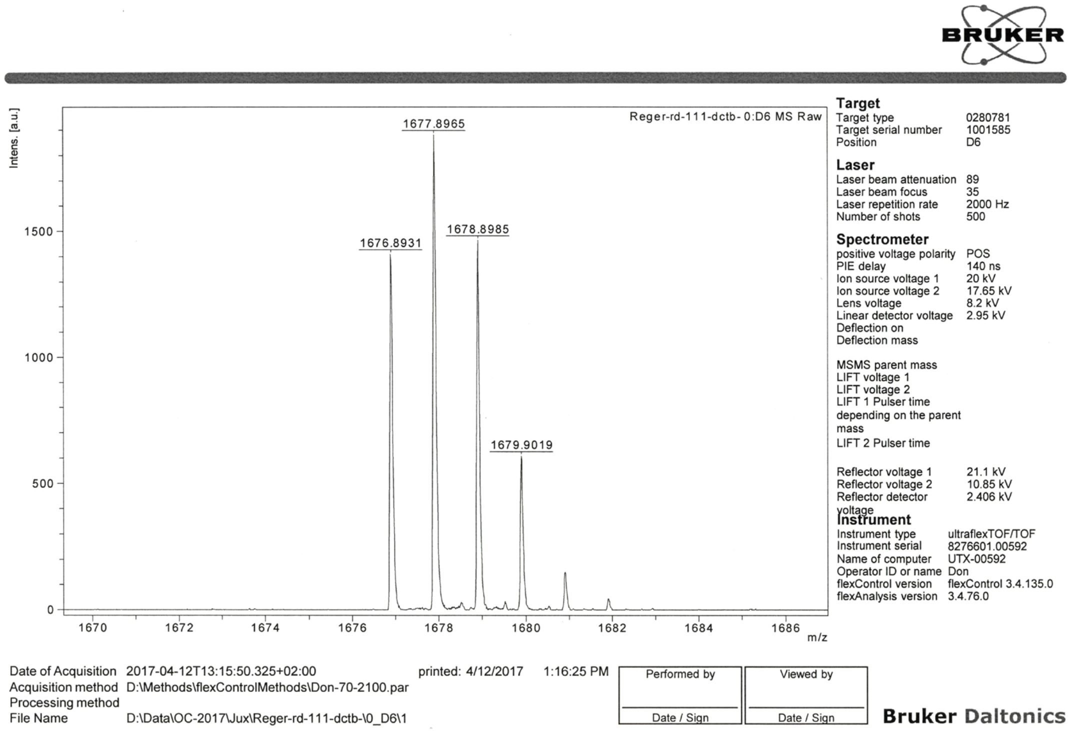

Figure S 180. MS-data (MALDI) of **21** zoom on product peak (dctb).

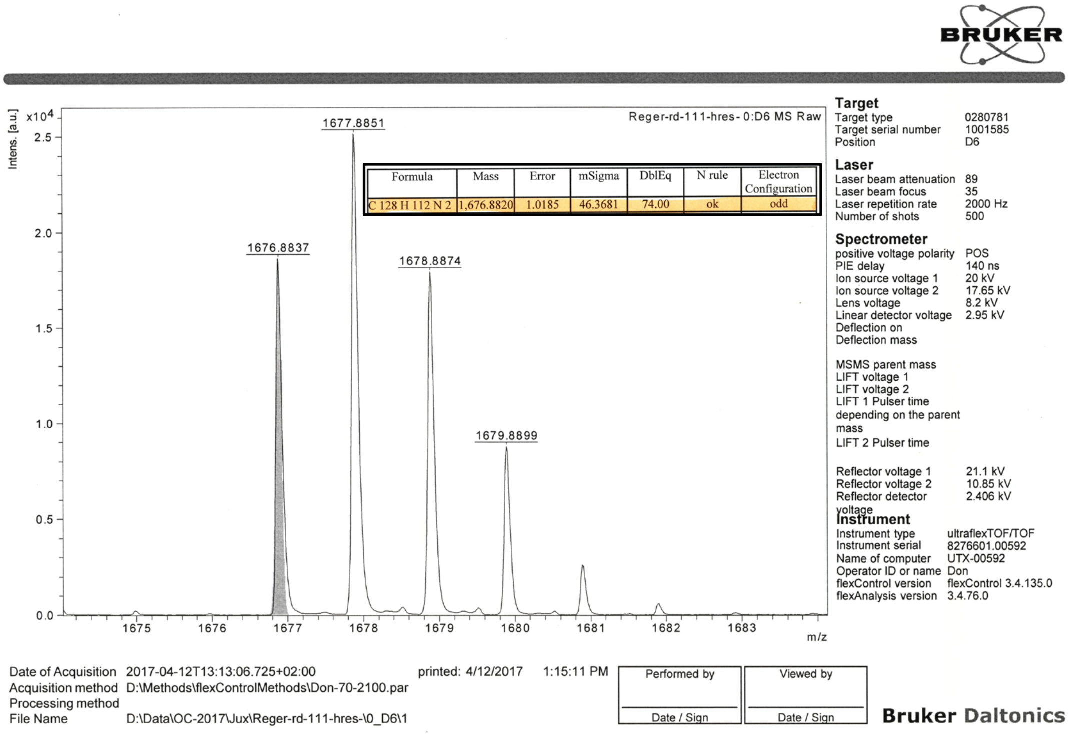

Figure S 181. HRMS-data (MALDI) of **21** (dctb). Insert: Calculated mass.

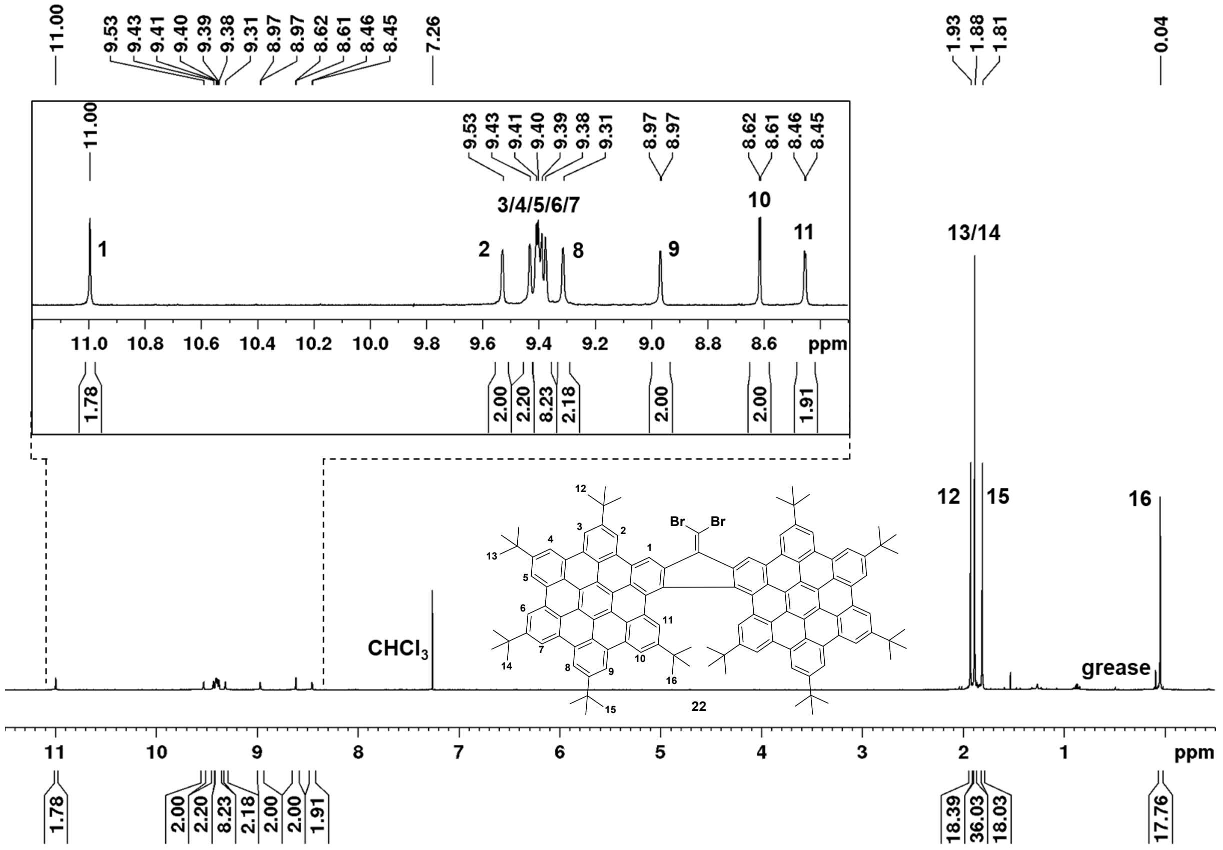

Figure S 182. ^{1}H NMR spectrum of **22** ($CDCl_3$, 400 MHz, rt.).

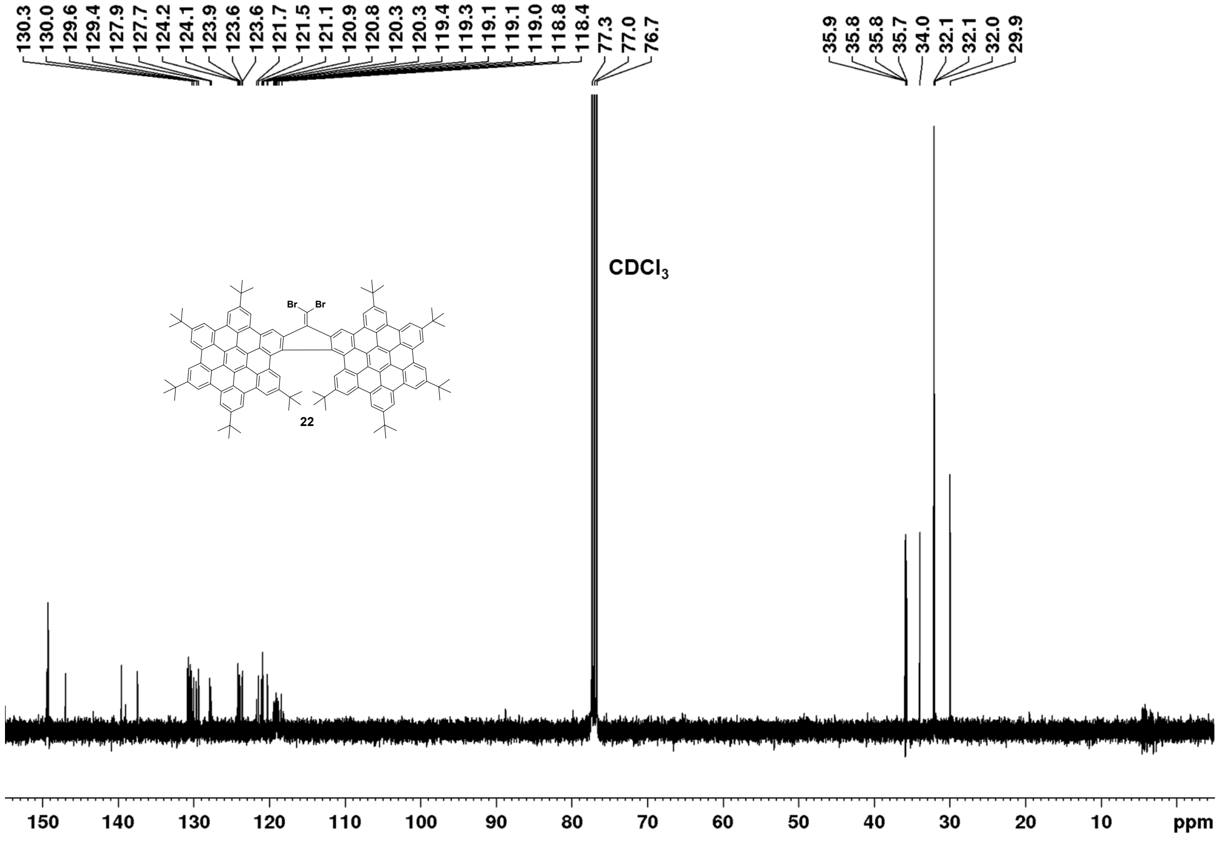

Figure S 183. Overview of the ^{13}C NMR spectrum of **22** ($CDCl_3$, 100 MHz, rt.).

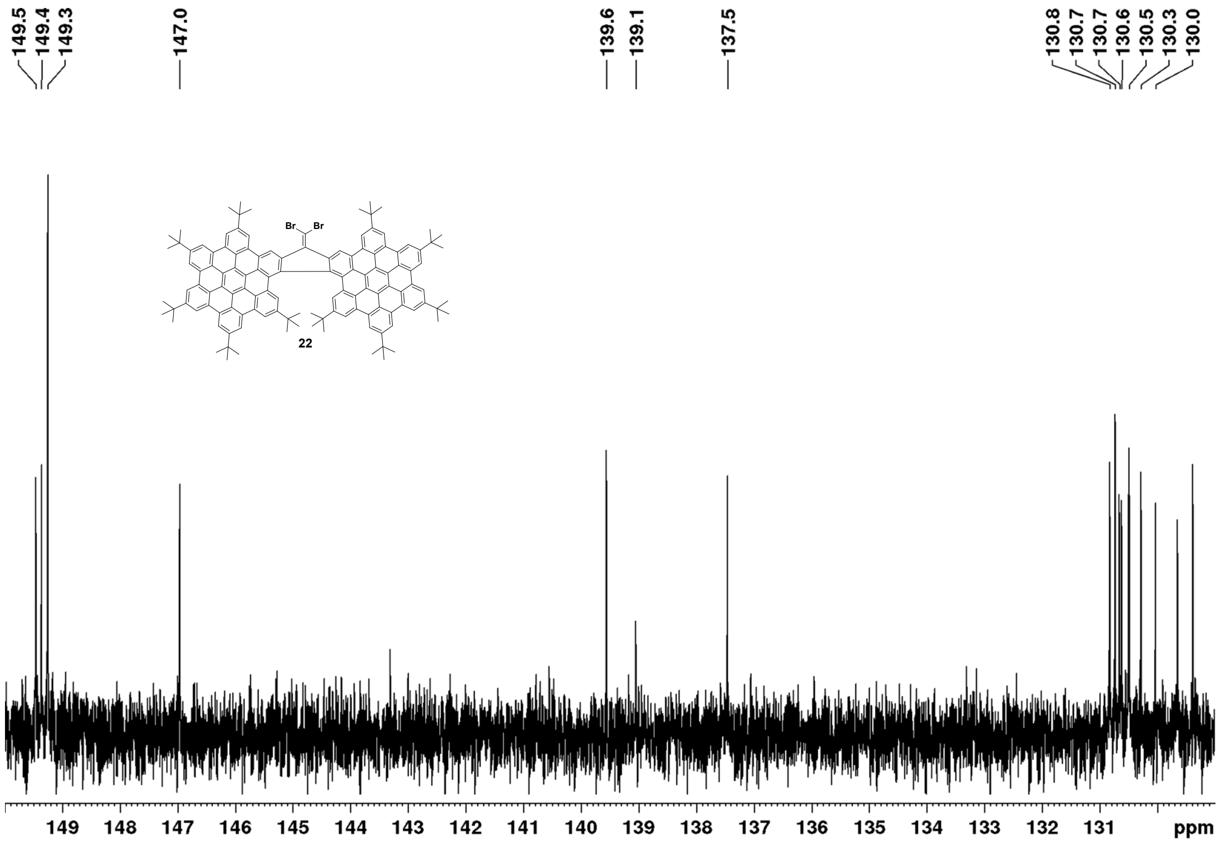

Figure S 184. Cutout (aromatic region) of the ^{13}C NMR spectrum of **22** ($CDCl_3$, 100 MHz, rt.).

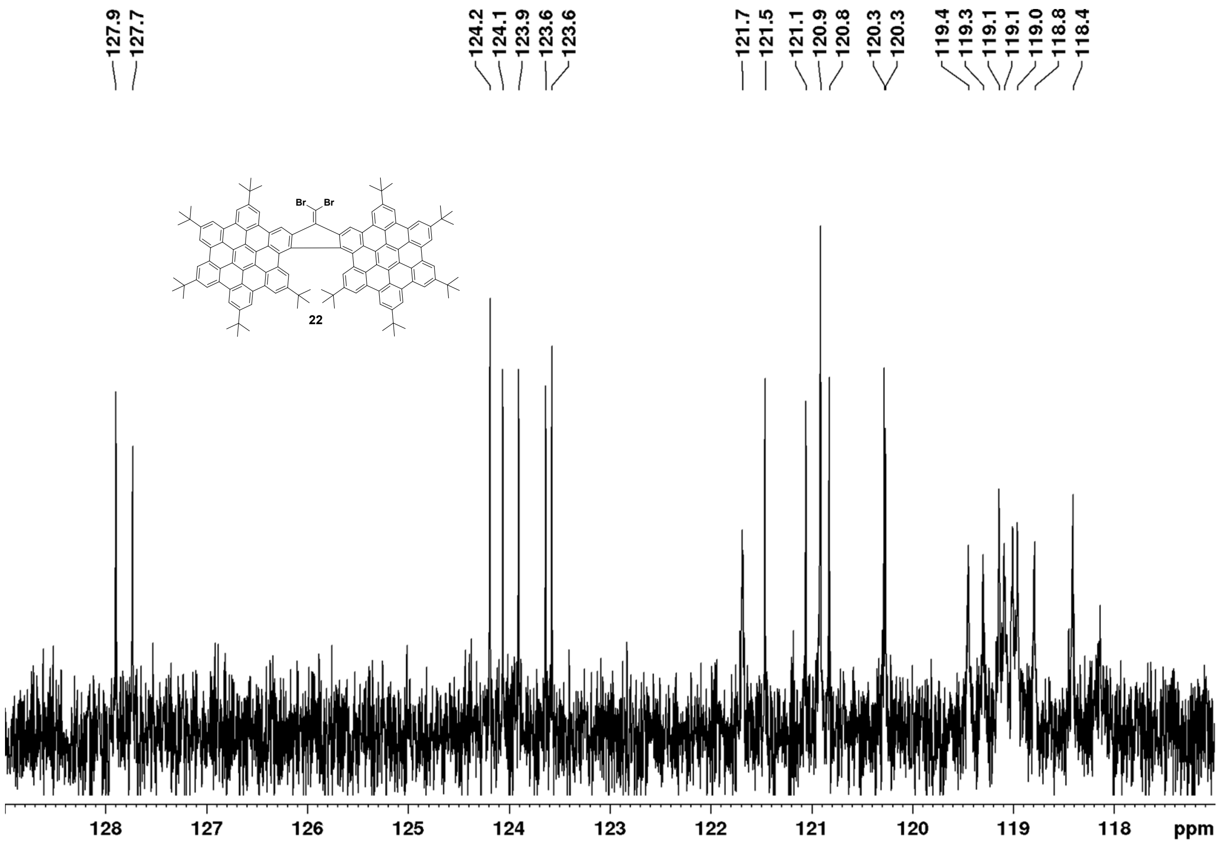

Figure S 185. Cutout (aromatic region) of the ^{13}C NMR spectrum of **22** ($CDCl_3$, 100 MHz, rt.).

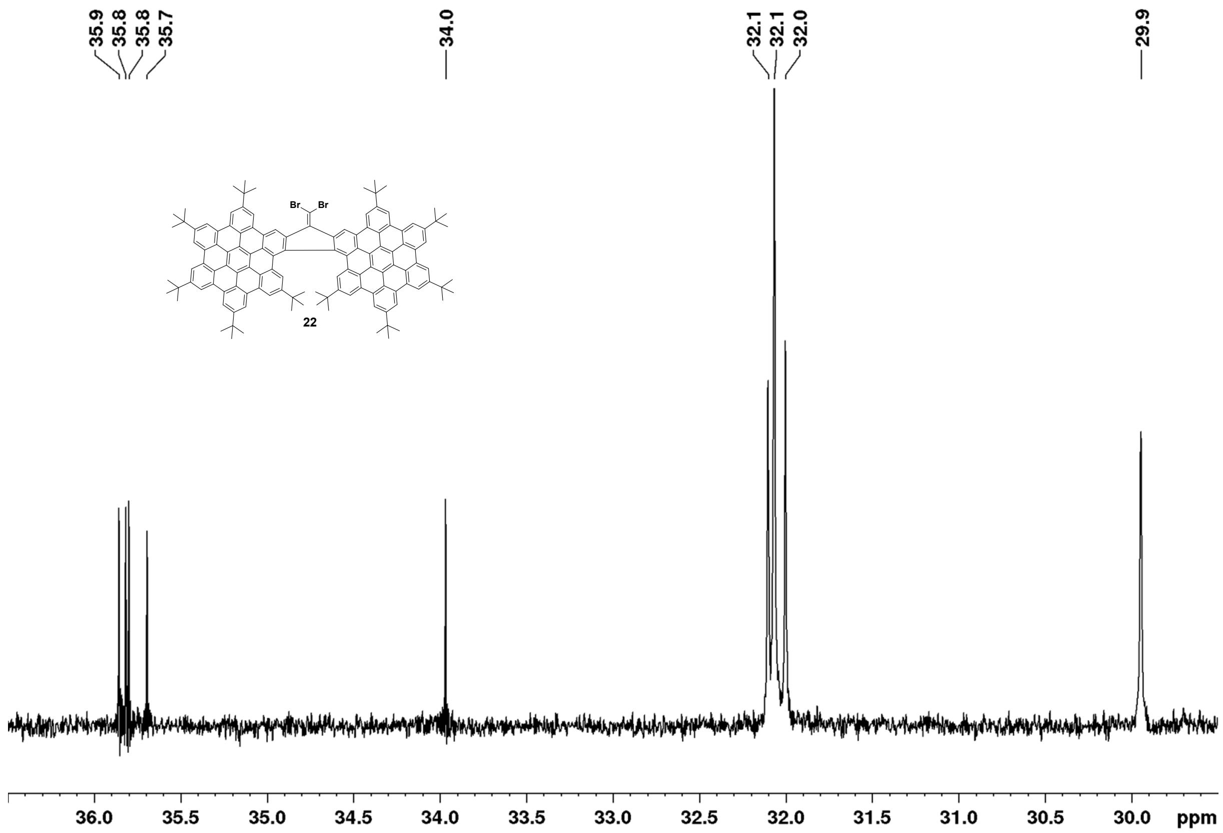

Figure S 186. Cutout (aliphatic region) of the ^{13}C NMR spectrum of **22** ($CDCl_3$, 100 MHz, rt.).

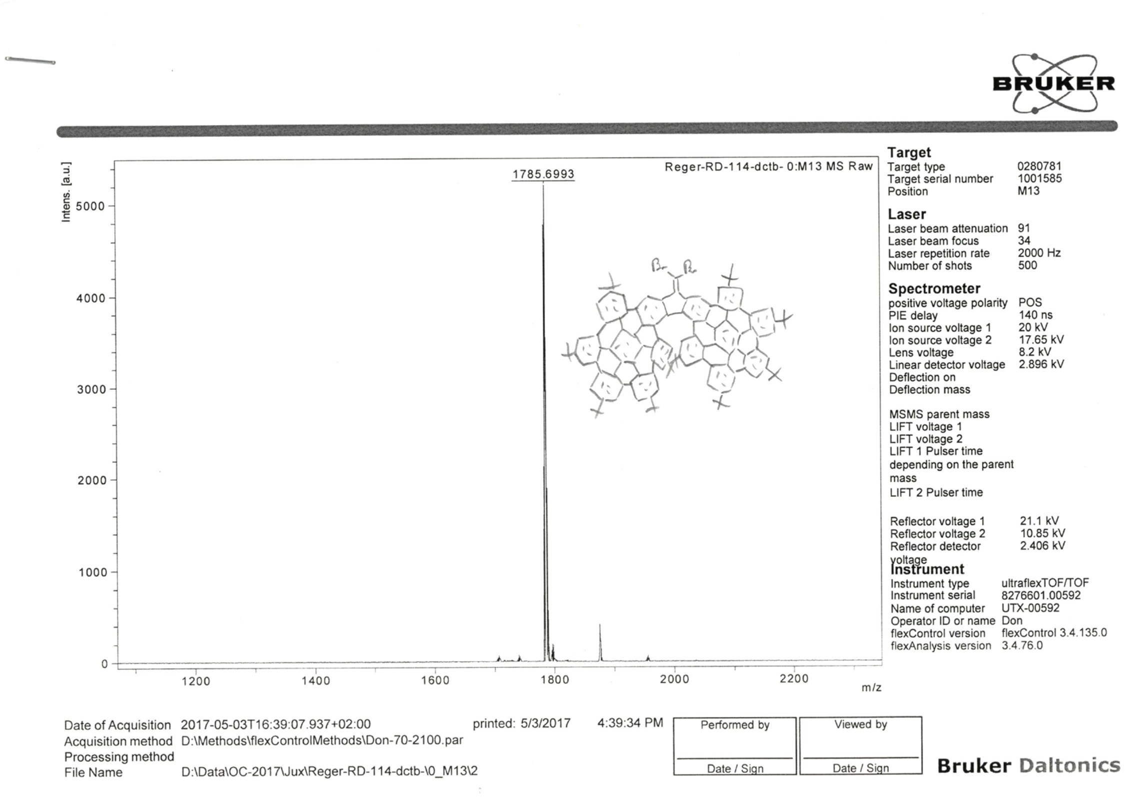

Figure S 187. MS-data (MALDI) of **22** (dctb).

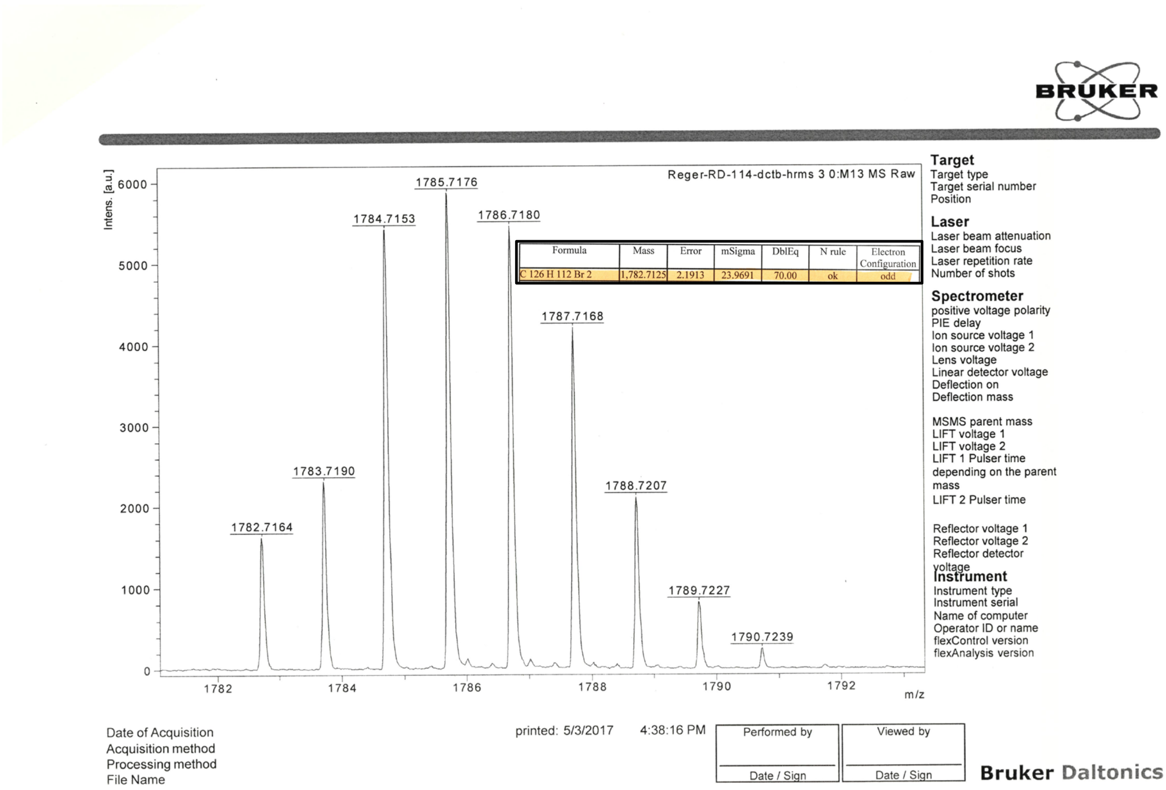

Figure S 188. HRMS-data (MALDI, dctb) of **22**. Insert: Calculated mass.

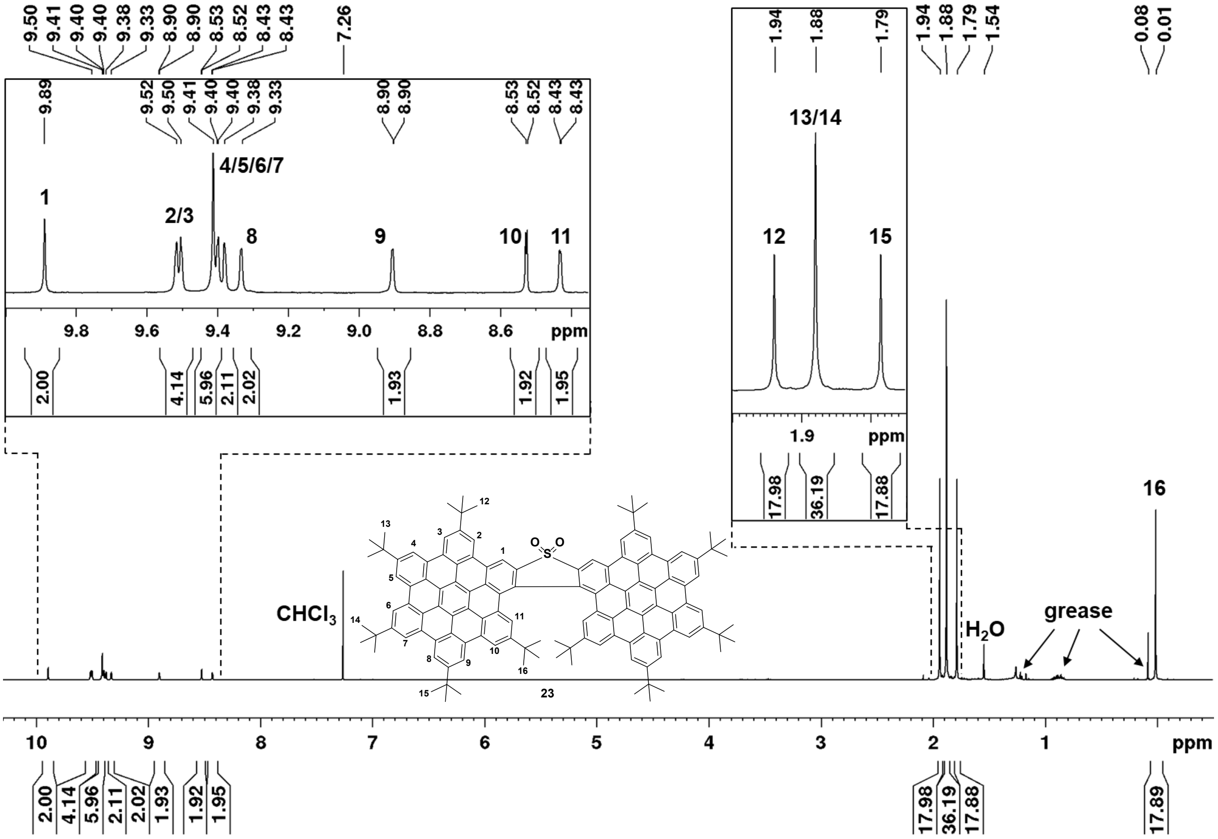

Figure S 189. ^{1}H NMR spectrum of **23** ($CDCl_3$, 400 MHz, rt.).

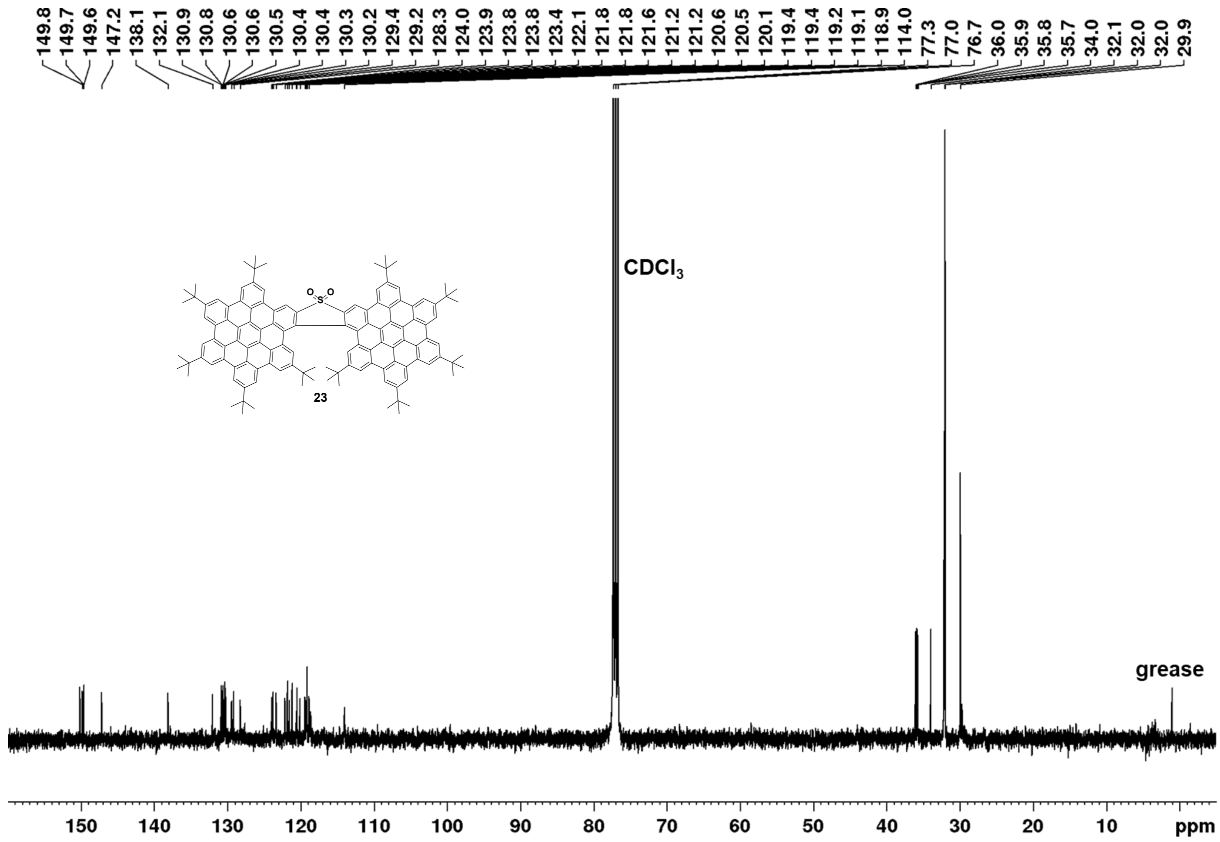

Figure S 190. Overview of the ^{13}C NMR spectrum of **23** ($CDCl_3$, 100 MHz, rt.).

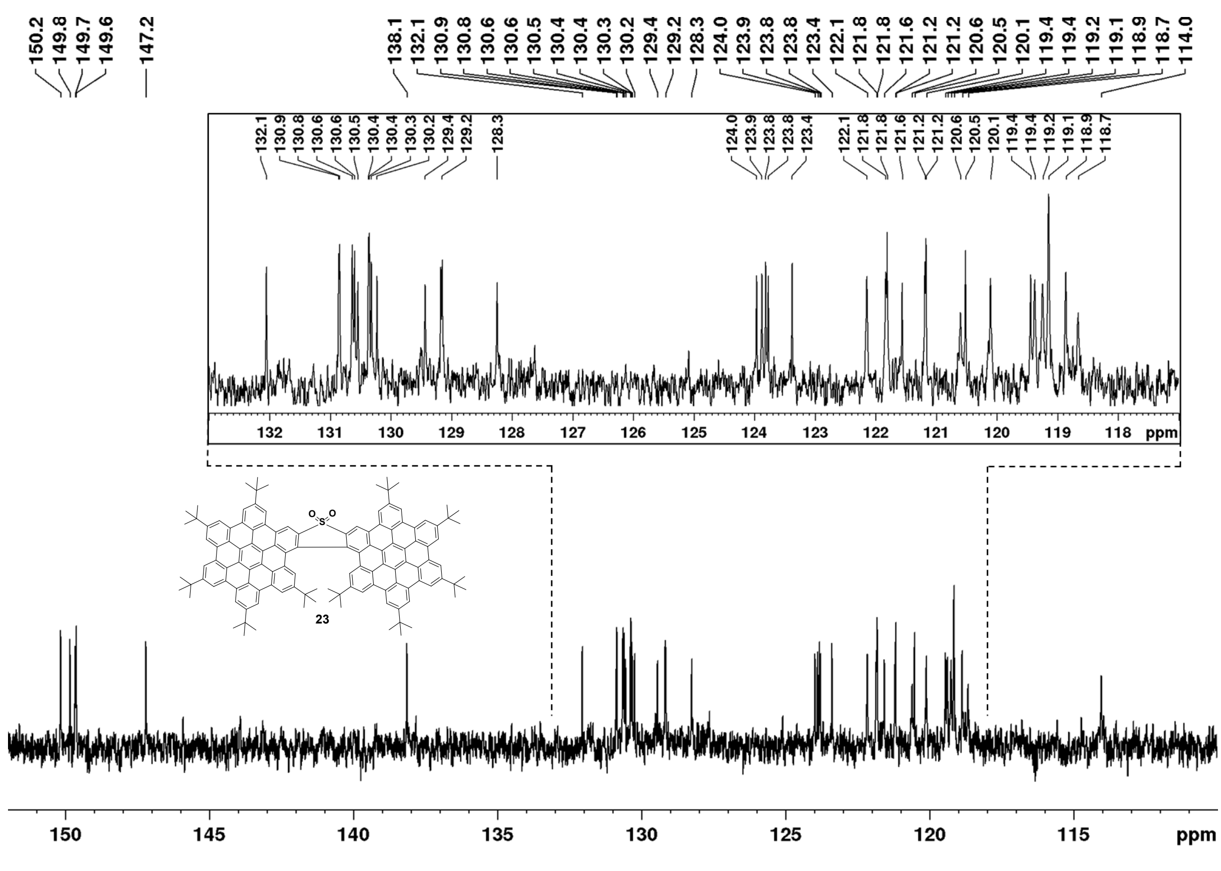

Figure S 191. Cutout (aromatic region) of the ^{13}C NMR spectrum of **23** ($CDCl_3$, 100 MHz, rt.).

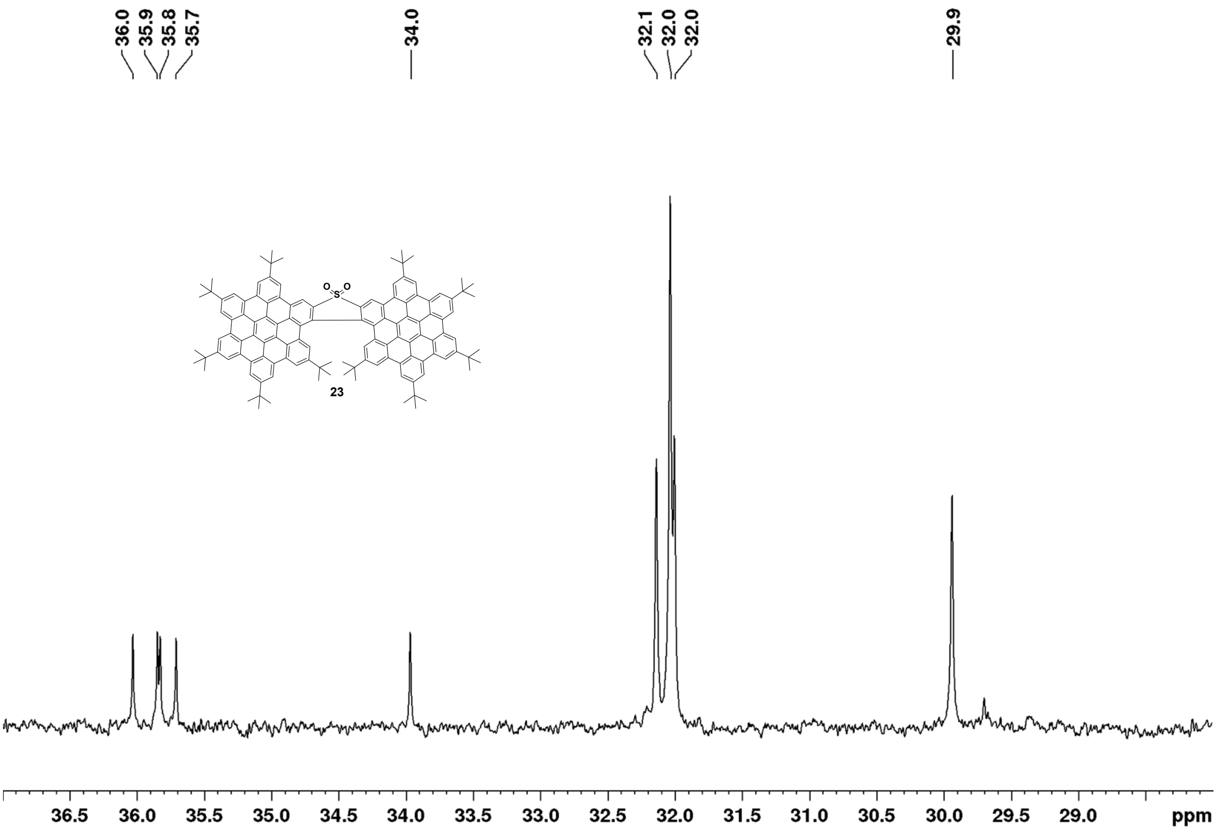

Figure S 192. Cutout (aliphatic region) of the ^{13}C NMR spectrum of **23** ($CDCl_3$, 100 MHz, rt.).

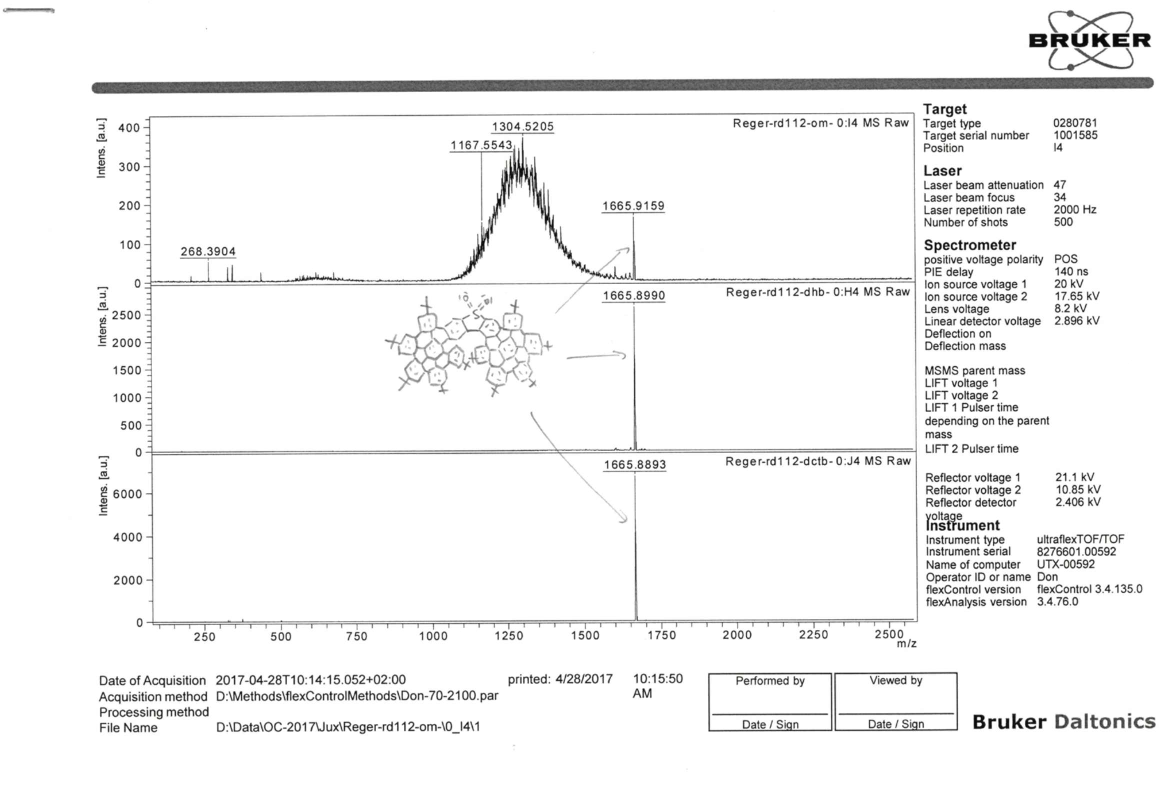

Figure S 193. MS-data (MALDI) of **23** (top: without matrix; middle: dhb; bottom: dctb).

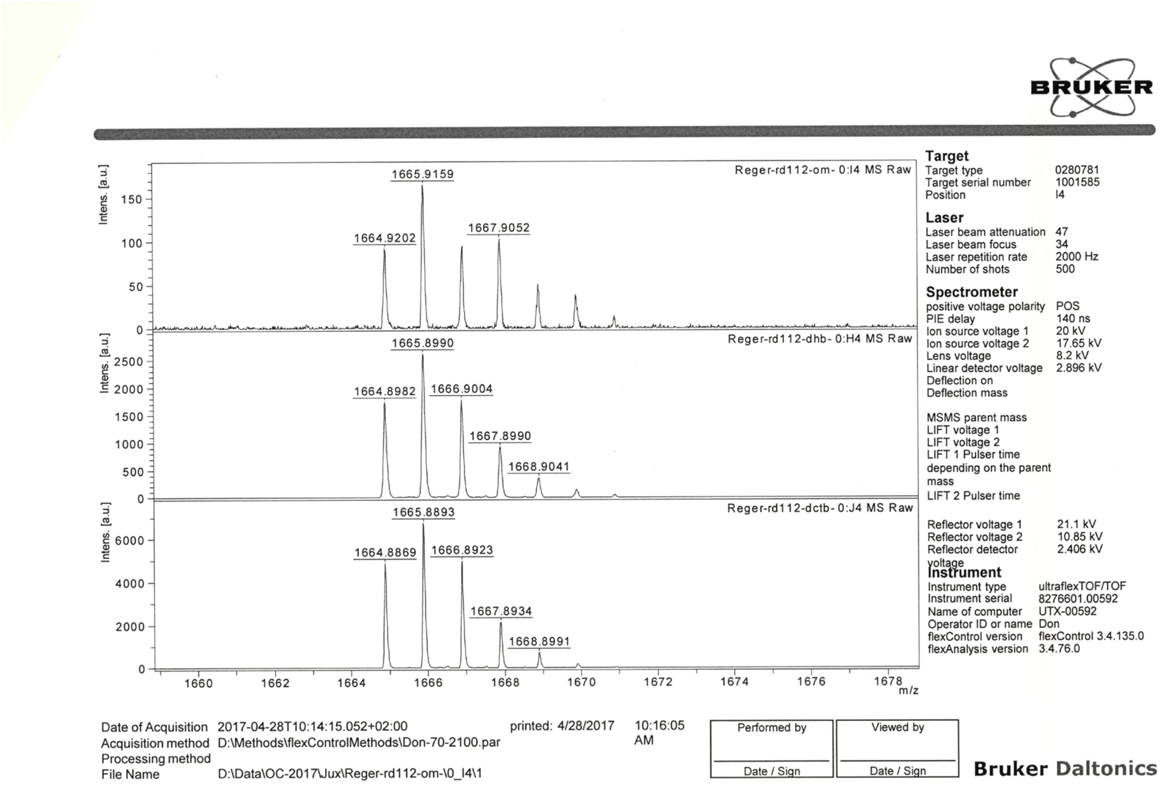

Figure S 194. MS-data (MALDI) of **23** zoom on product peak (top: without matrix; middle: dhb; bottom: dctb).

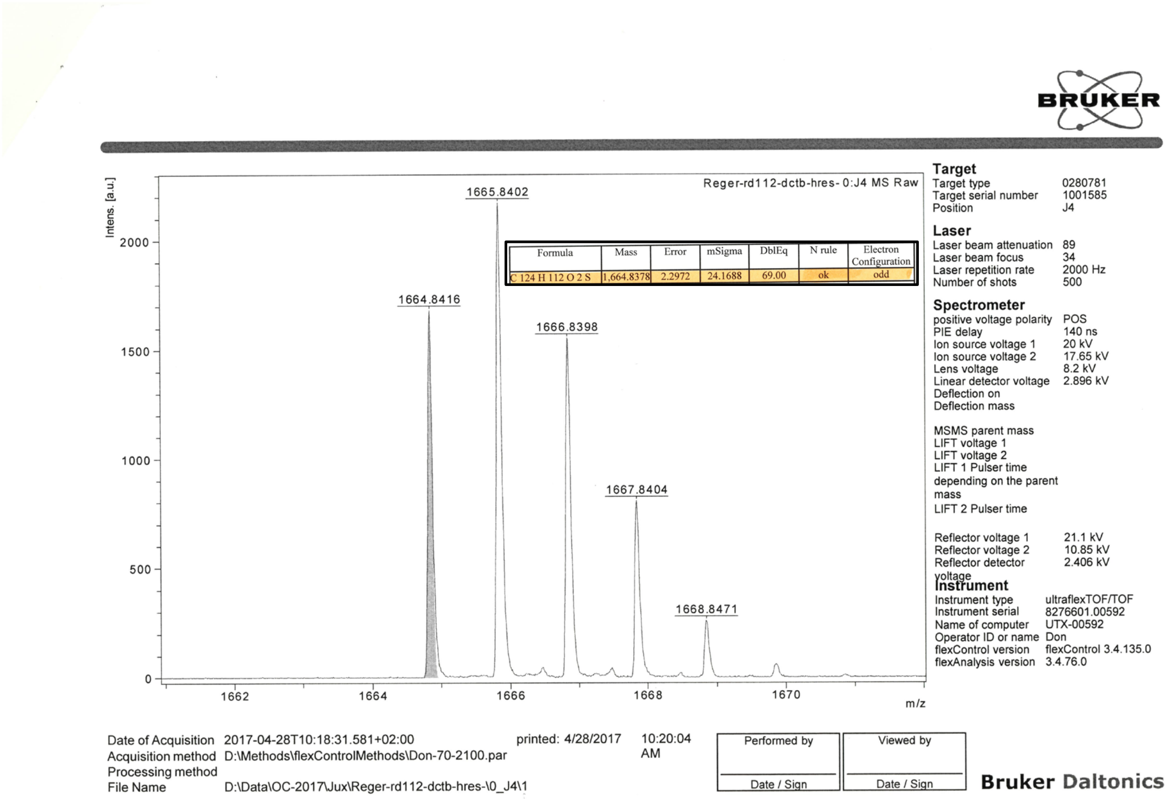

Figure S 195. HRMS-data (MALDI, dctb) of **23**. Grey background: Simulated. Insert: Calculated mass.

10 Literature

[S1] B. Kobin, L. Grubert, S. Blumstengel, F. Henneberger, S. Hecht, *J. Mater. Chem.* **2012**, *22*, 4383-4390.

[S2] K. Lin, Y. Zhao, S. Ming, H. Liu, S. Zhen, J. Xu, B. Lu, *J. Polym. Sci. Pol. Chem.* **2016**, *54*, 1468-1478.

[S3] S. Kumar, S. Patil, *New J. Chem.* **2015**, *39*, 6351-6357.

[S4] P. P. Bag, D. Wang, Z. Chen, R. Cao, *Chem. Commun.* **2016**, *52*, 3669-3672.

[S5] D. Reger, P. Haines, F. W. Heinemann, D. M. Guldi, N. Jux, *Angew. Chem. Int. Ed.* **2018**, *57*, 5938-5942.

[S6] D. Lungerich, J. F. Hitzenberger, M. Marcia, F. Hampel, T. Drewello, N. Jux, *Angew. Chem. Int. Ed.* **2014**, *53*, 12231-12235

[S7] U. T. Mueller-Westerhoff, M. Zhou, *Tetrahedron Lett.* **1993**, *34*, 571-574.

[S8] X. Dou, W. Pisula, J. Wu, G. J. Bodwell, K. Müllen, *Chem. Eur. J.* **2008**, *14*, 240-249.

[S9] O. V. Dolomanov, L. J. Bourhis, R. J. Gildea, J. A. K. Howard, H. Puschmann, *J. Appl. Cryst.* **2009**, *42*, 339-341.

[S10] G. M. Sheldrick, *Acta Cryst. A*, **2008**, *64*,112-122.

[S11] G. M. Sheldrick, *Acta Cryst. C*, **2015**, *71*, 3-8.

[S12] V. Coropceanu, J. Cornil, D. A. da Silva Filho, Y. Olivier, R. Silbey, J.-L. Brédas, *Chem. Rev.* **2007**, *107*, 926-952.

[S13] Y. Kim, J. Nelson, T. Zhang, S. Cook, J. R. Durrant, H. Kim, J. Park, M. Shin, S. Nam, M. Heeney, I. McCulloch, C.-S. Ha, D. D. C. Bradley, *ACS Nano*, **2009**, *3*, 2557-2562.

[S14] H. Oberhofer, K. Reuter, J. Blumberger, *Chem. Rev.* **2017**, *117*, 10319-10357.

[S15] H. Yang, F. Gajdos, J. Blumberger, *J. Phys. Chem. C*, **2017**, *121*, 7689-7697.

[S16] H. Bässler, A. Köhler, Top. Curr. Chem, **2012**, *312*, 1–66.

[S17] F. Steiner, S. Foster, A. Losquin, J. Labram, T. D. Anthopoulos, J. M. Frost, J. Nelson, *Mater. Horiz.* **2015**, *2*, 113-119.

[S18] D. Shi, X. Qin, Y. Li, Y. He, C. Zhong, J. Pan, H. Dong, W. Xu, T. Li, W. Hu, J.-L. Brédas, O. M. Bakr, *Science Advances*, **2016**, *2*, e1501491.

[S19] F. Musil, S. De, J. Yang, J. E. Campbell, G. M. Day, M. Ceriotti, *Chem. Sci.* **2018**, *9*, 1289-1300.

[S20] J. Wade, F. Steiner, D. Niedzialek, D. T. James, Y. Jung, D.-J. Yun, D. D. C. Bradley, J. Nelson, J.-S. Kim, *J. Mater. Chem. C*, **2014**, *2*, 10110-10115.

[S21] B. Rice, L. M. LeBlanc, A. Otero-de-la-Roza, M. J. Fuchter, E. R. Johnson, K. E. Jelfs, *Nanoscale*, **2018**, *10*, 1865-1876.

[S22] B. Rice, L. M. LeBlanc, A. Otero-de-la-Roza, M. J. Fuchter, E. R. Johnson, K. E. Jelfs, *Nanoscale*, **2018**, *10*, 9410-9410. (Correction)

[S23] Y. Yang, B. Rice, X. Shi, J. R. Brandt, R. Correa da Costa, G. J. Hedley, D.-M. Smilgies, J. M. Frost, I. D. W. Samuel, A. Otero-de-la-Roza, E. R. Johnson, K. E. Jelfs, J. Nelson, A. J. Campbell, A. J. Fuchter, *ACS Nano*, **2017**, *11*, 8329-8338.

[S24] F. Salerno, B. Rice, J. A. Schmidt, M. J. Fuchter, J. Nelson, K. E. Jelfs, *Phys. Chem. Chem. Phys.* **2019**, *21*, 5059-5067.

[S25] B. Aradi, B. Hourahine, T. Frauenheim, *J. Phys. Chem. A* **2007**, *111*, 5678-5684.

[S26] Gaussian 16, Revision C.01, M. J. Frisch, G. W. Trucks, H. B. Schlegel, G. E. Scuseria, M. A. Robb, J. R. Cheeseman, G. Scalmani, V. Barone, G. A. Petersson, H. Nakatsuji, X. Li, M. Caricato, A. V. Marenich, J. Bloino, B. G. Janesko, R. Gomperts, B. Mennucci, H. P. Hratchian, J. V. Ortiz, A. F. Izmaylov, J. L. Sonnenberg, D. Williams-Young, F. Ding, F. Lipparini, F. Egidi, J. Goings, B. Peng, A. Petrone, T. Henderson, D. Ranasinghe, V. G. Zakrzewski, J. Gao, N. Rega, G. Zheng, W. Liang, M. Hada, M. Ehara, K. Toyota, R. Fukuda, J. Hasegawa, M. Ishida, T. Nakajima, Y. Honda, O. Kitao, H. Nakai, T. Vreven, K. Throssell, J. A. Montgomery, Jr., J. E. Peralta, F. Ogliaro, M. J. Bearpark, J. J. Heyd, E. N. Brothers, K. N. Kudin, V. N. Staroverov, T. A. Keith, R. Kobayashi, J. Normand, K. Raghavachari, A. P. Rendell, J. C. Burant, S. S. Iyengar, J. Tomasi, M. Cossi, J. M. Millam, M. Klene, C. Adamo, R. Cammi, J. W. Ochterski, R.

L. Martin, K. Morokuma, O. Farkas, J. B. Foresman, and D. J. Fox, Gaussian, Inc., Wallingford CT, 2016.

[S27] J. Kirkpatrick, *Int. J. Quantum Chem.* **2008**, *108*, 51-56.

[S28] R. A. Marcus, *J. Chem. Phys.* **1956**, *24*, 966-978.

[S29] D. Andrienko, J. Kirkpatrick, V. Marcon, J. Nelson, K. Kremer, *Phys. Status Solidi. B* **2008**, *245*, 830-834.

[S30] J. Blumberger, G. Lamoureux, *Mol. Phys.* **2008**, *106*, 1597-1611.

[S31] K. Sakanoue, M. Motoda, M. Sugimoto, S. Sakaki, *J. Phys. Chem. A* **1999**, *103*, 5551-5556.

[S32] G. D'Avino, L. Muccioli, Y. Olivier, D. Beljonne, *J. Phys. Chem. Lett.* **2016**, *7*, 3, 536–540.

[S33] E. F. Oliveira, F. C. Lavarda, *Polymer*, **2016**, *99*, 105-111.

[S34] G. R. Hutchinson, M. A. Ratner, T. J. Marks, *J. Am. Chem. Soc.* **2005**, *127*, 2339-2350.

[S35] a) Turbomole 6.6 **2014**, a development of University of Karlsruhe and Forschungszentrum Karlsruhe GmbH, 1989, Turbomole GmbH, since 2007; available from www.turbomole.com. b) R. Ahlrichs, M. Baer, M. Haeser, H. Horn, C. Koelmel, *Chem. Phys. Lett.* **1989**, *162*, 165-169.

[S36] a) Orca version 4.2.1, b) F. Neese, *Wiley Interdiscip. Rev.: Comput. Mol. Sci.* **2012**, *2*, 73 c) F. Neese, *Wiley Interdiscip. Rev.: Comput. Mol. Sci.* **2017**, *8*, e1327.

[S37] J. P. Perdew, K. Burke, M. Ernzerhof, *Phys. Rev. Lett.* **1996**, *77*, 3865–3868.

[S38] F. Weigend, R. Ahlrichs, *Phys. Chem. Chem. Phys.* **2005**, *7*, 3297–3305.

[S39] a) K. Eichkorn, O. Treutler, H. Öhm, M. Häser, R. Ahlrichs, *Chem. Phys. Lett.* **1995**, *240*, 283-290; b) K. Eichkorn, O. Treutler, H. Öhm, M. Häser, R. Ahlrichs, *Chem. Phys. Lett.* **1995**, *242*, 652-660; c) K. Eichkorn, F. Weigend, O. Treutler, R. Ahlrichs, *Theor. Chem. Acc.* **1997**, *97*, 119-124.

[S40] F. Weigend, *Phys. Chem. Chem. Phys.* **2006**, *8*, 1057.

[S41] M. Sierka, A. Hogekamp, R. Ahlrichs, *J. Chem. Phys.* **2003**, *118*, 9136-9148.

[S42] S. Grimme, J. Antony, S. Ehrlich, H. Krieg, *J. Chem. Phys.* **2010**, *132*, 154104.

[S43] S. Grimme, S. Ehrlich, L. Goerigk, *J. Comput. Chem.* **2011**, *32*, 1456-1465.

[S44] a) P. Deglmann, F. Furche, *J. Chem. Phys.* **2002**, *117*, 9535-9538; b) P. Deglmann, F. Furche, R. Ahlrichs, *Chem. Phys. Lett.* **2002**, *362*, 511-518.

[S45] a) A. D. Becke, *J. Chem. Phys.* **1993**, *98*, 5648-5652, b) P. J. Stephens, F. J. Devlin, C. F. Chabalowski, M. J. Frisch, *J. Phys. Chem.* **1994**, *98*, 11623-11627.

[S46] a) J.-D. Chai., M. Head-Gordon, *J. Chem. Phys.* **2008**, *128*, 084106; b) Y.-S. Lin, G.-D. Li, S.-P. Mao, J.-D. Chai, *J. Chem. Theory Comput.* **2013**. *9*, 263-272.

[S47] a) S. Grimme, *J. Chem. Phys.* **2013**, *138*, 244104; b) T. Risthaus, A. Hansen, S. Grimme, *Phys. Chem. Chem. Phys.* **2014**, *16*, 14408-14419.

[S48] C. Bannwarth, S. Grimme, *Comp. Theor. Chem.* **2014**,*1040-1041*, 45-53.

[S49] a) M.E. Casida, D. Chong (Ed.), *Recent Advances in Density Functional Theory*, World Scientific, Singapore, **1995**, 155-192; b) R. Bauernschmitt, R. Ahlrichs, *Chem. Phys. Lett.* **1996**, *256*, 454, c) F. Furche, R. Ahlrichs, *J. Chem. Phys*. **2002**, *117*, 7433; Erratum: **2004**,*121*, 12772

[S50] T. Petrenko, S. Kossmann, F. Neese, *J. Chem. Phys.* **2011**,*134*, 054116.

[S51] F. Neese, F. Wennmohs, A. Hansen, U. Becker, *Chem. Phys.* **2009**, *356*, 98-109.

[S52] C. Wolfarth, Permittivity (Dielectric Constant) of Liquids" (Interactive Table) in CRC Handbook of Chemistry and Physics, 101th Edition (Internet Version), CRC Press/Taylor & Francis, Boca Raton, FL., **2020**.

[S53] “Physical Constants of Organic Compounds” in J. R. Rumble, ed., CRC Handbook of Chemistry and Physics, 101th Edition (Internet Version), CRC Press/Taylor & Francis, Boca Raton, FL., **2020**.

[S54] A. Klamt, G. Schüürmann, *J. Chem. Soc., Perkin Trans. 2*, **1993**, 799-805.

[S55] V. Barone, M. Cossi, *J. Phys. Chem. A* ***1998**, 102,* 1995-2001.
